# Development of Nurr1 agonists from amodiaquine by scaffold hopping and fragment growing

**DOI:** 10.1038/s42004-024-01224-0

**Published:** 2024-06-29

**Authors:** Minh Sai, Emily C. Hank, Hin-Man Tai, Till Kasch, Max Lewandowski, Michelle Vincendeau, Julian A. Marschner, Daniel Merk

**Affiliations:** 1 Ludwig-Maximilians-Universität München, Department of Pharmacy, 81377 Munich, Germany; 2 Helmholtz Munich, Institute of Virology, 85764 Munich, Germany; 3 Technical University of Munich, Institute of Virology, School of Medicine, 81675 Munich, Germany

**Keywords:** Lead optimization, Drug discovery and development, Target validation

## Abstract

The neuroprotective transcription factor nuclear receptor-related 1 (Nurr1) has shown great promise as a therapeutic target in Parkinson’s and Alzheimer’s disease as well as multiple sclerosis but high-quality chemical tools for pharmacological target validation of Nurr1 are rare. We have employed the weak Nurr1 modulator amodiaquine (AQ) and AQ-derived fragments as templates to design a new Nurr1 agonist chemotype by scaffold hopping and fragment growing strategies. Systematic structural optimization of this scaffold yielded Nurr1 agonists with nanomolar potency and binding affinity. Comprehensive in vitro profiling revealed efficient cellular target engagement and compliance with the highest probe criteria. In human midbrain organoids bearing a Parkinson-driving LRRK2 mutation, a novel Nurr1 agonist rescued tyrosine hydroxylase expression highlighting the potential of the new Nurr1 modulator chemotype as lead and as a chemical tool for biological studies.

## Abstract (web summary)

Nuclear receptor-related 1 (Nurr1) is a promising candidate target for neurodegenerative disease treatment, however, the validation of their therapeutic potential remains underexplored due to a lack of high-quality chemical tools. Here, the authors develop Nurr1 agonists from amodiaquine by scaffold hopping and fragment growing, exhibiting nanomolar potency and efficient cellular target engagement, showing their potential as lead and chemical tools.

## Introduction

Nuclear receptor related 1 (Nurr1, NR4A2) is a ligand-activated transcription factor mainly expressed in neurons and immune cells of the brain^1–3^. The receptor is critically involved in the regulation of (dopaminergic) neuron development and maintenance as well as inflammatory processes^3–6^. Observations of diminished Nurr1 levels in patients^7^ and animal models^8–10^ of Alzheimer’s (AD) and Parkinson’s diseases (PD) underline the therapeutic potential of Nurr1 activation in neurodegenerative diseases. Moreover, recent findings suggest a protective and anti-inflammatory role of Nurr1 in retinal pigment epithelial cells in the eye with possible therapeutic relevance in age-related macular degeneration^11^. Pharmacological activation of Nurr1 may therefore offer new therapeutic options in various degenerative diseases and potent Nurr1 agonists are needed^12^.

**Fig. 1:**
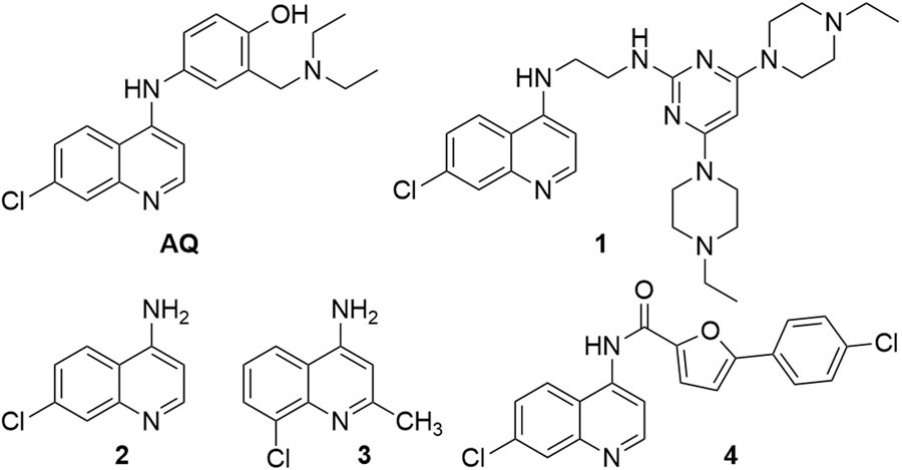
Nurr1 agonists. Amodiaquine (AQ)^24^, the optimized AQ derivative **1**^36^, the AQ-derived fragments **2** and **3**^28^, and the AQ derivative **4** from microscale combinatorial chemistry^37^.

Nurr1 acts as a monomer, homodimer, or heterodimer and has constitutive transcriptional activator activity also in the absence of ligands but can be modulated by agonists and inverse agonists in a bidirectional fashion^13–15^. The dopamine metabolite 5,6-dihydroxyindole (DHI)^16^, polyunsaturated fatty acids^17^ and the prostaglandins A and E^18^ have been identified as natural Nurr1 ligands. A few synthetic Nurr1 ligand chemotypes have been recently identified^19–23^ among which the antimalarial amodiaquine (AQ; Fig. 1; EC_50_~20 µM)^24^ was the first validated Nurr1 agonist and emerged as an early tool to study therapeutic effects of Nurr1 activation^24^. AQ treatment counteracted neuroinflammation and ameliorated behavioral deficits in a PD model^24^, and reduced neuronal loss and amyloid-beta deposition in an AD model^25^. However, while offering access to Nurr1 ligand discovery, AQ is unspecific^19,26–28^ and hepatotoxic^29^, and pharmacological effects with AQ cannot confidently be assigned to Nurr1 modulation. For example, AQ was found to inhibit autophagy, stabilize p53, block ribosome biogenesis, suppress PPARγ induced adipogenesis, and cause endoplasmic reticulum stress^30–33^ thus affecting multiple cellular processes. Moreover, AQ and many analogues contain pan-assay interference compounds (PAINS)^34,35^ elements further compromising their value as a chemical tool. Therefore, AQ descendants with improved potency and selectivity are needed to validate the promising observations on Nurr1-mediated therapeutic effects of this drug. So far, structural optimization efforts have yielded the AQ descendant **1** exhibiting improved Nurr1 agonist potency and protective effects in a PD model^36^, simplified 7-chloroquinolin-4-amine (**2**) and 8-chloro-2-methylquinolin-4-amine (**3**) fragments with enhanced Nurr1 agonism^28^, and a 5-(4-chlorophenyl)furan-2-carboxamide motif (**4**)^37^ as replacement for the unfavorable 4-aminophenol residue of AQ.

Here, we aimed to develop AQ-derived Nurr1 agonists to enable target validation studies on the promising effects of AQ in neurodegeneration. Systematic optimization, scaffold-hopping, and fusion of AQ substructures yielded a next-generation Nurr1 ligand scaffold offering high potency and selectivity. Comprehensive characterization and validation demonstrated high affinity binding to Nurr1, cellular target engagement, low toxicity, favorable permeability, and tunable metabolic stability. A potent and extensively validated Nurr1 agonist of the AQ-derived chemotype and a structurally matched negative control compound together provide a high-quality chemical tool for biological studies to validate Nurr1-dependent pharmacological effects of AQ and advance Nurr1 modulation as a therapeutic concept.

## Results & Discussion

### Design and structural optimization of Nurr1 agonists

Antimalarial activity of AQ and related compounds is mainly ascribed to the common chloroquinoline scaffold present in several antimalarials^38^ while the 4-hydroxyaniline is considered mainly responsible for cyto- and hepatotoxicity after metabolic activation to a reactive quinoneimine^29,32^. Additionally, cellular AQ effects, e.g., on autophagy and p53, surpassed the activity of chloroquinone (CQ)^30,31^ which is structurally related but lacks the 4-aminophenol moiety indicating that this substructure also mediates off-target activities of AQ.

Optimization of the AQ scaffold for Nurr1 agonism hence demanded the replacement of both the chloroquinolineamine and 4-aminophenol motifs. As we have previously found that 7-chloroquinoline-4-amine (**2**) is sufficient for Nurr1 activation and can be tuned in Nurr1 agonism by variation of its substitution pattern^28^, we aimed for optimization of the chloroquinolineamine fragment by scaffold hopping and subsequent fragment extension. Thus, we commenced the development of AQ-derived Nurr1 modulators by probing replacement of the quinoline scaffold of the optimized AQ fragment **3** by alternative heterocycles (Table 1). The corresponding quinazoline **5** was inactive and 7-chloro-2-methyl-1*H*-indol-3-amine (**6**) was not stable. 4-Chloro-2-methyl-benzimidazole (**7**) also failed to activate Nurr1 suggesting that the amino substituent was required and could not be replaced by the ring NH.

**Table 1:** Optimization of the chloroquinoline fragment

ID	structure	EC_50_(Nurr1) (max. activation)^a^	K_d_ (Nurr1 LBD)^b^
**3**		17 ± 6 µM (1.7 ± 0.1-fold)	n.d.
**5**		inactive (100 µM)	n.d.
**6**		unstable	n.d.
**7**		inactive (100 µM)	n.d.
**8**		7 ± 1 µM (2.0 ± 0.1-fold)	2.7 µM
**9**		inactive (100 µM)	weak binding
**10**		inactive (100 µM)	weak binding
**11**		inactive (100 µM)	7.2 µM
**12**		< 1.2-fold activation	5.1 µM
**13**		inactive (100 µM)	weak binding

^a^Nurr1 modulation was determined in a Gal4-Nurr1 hybrid reporter gene assay. Max. activation refers to the maximum effect vs. 0.1% DMSO control. Data are the mean ± SD; *n* ≥ 3. ^b^K_d_ values were determined by isothermal titration calorimetry (cf. Fig. 2a Supplementary Fig. 1). n.d. - not determined; weak binding - titration of 15 µM Nurr1 with 100 µM ligand showed heat differences indicative of binding that could not be fitted, however.

**Fig. 2:**
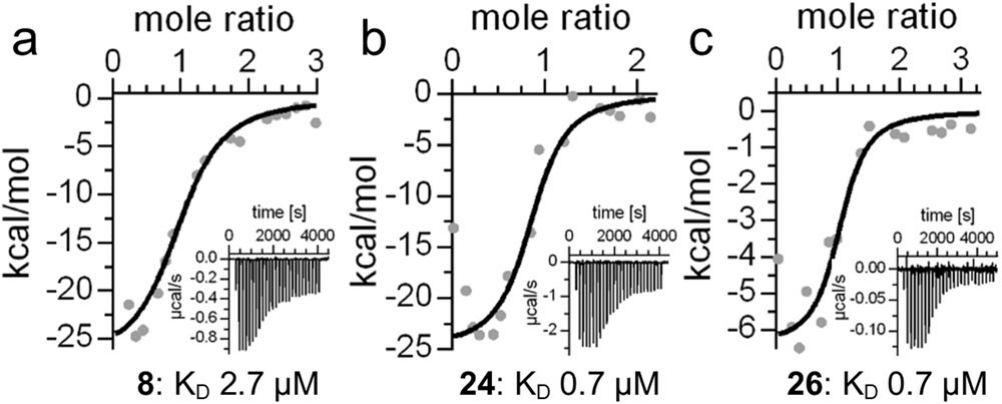
Orthogonal validation of ligand binding to Nurr1 by isothermal titration calorimetry (ITC). The fittings of the heat of binding are shown for **8** (**a**), **24** (**b**), and **26** (**c**) and the isotherms at 25 °C are shown as insets.

The alternative imidazo[1,2-*a*]pyridine scaffold (**8**), in contrast, retained Nurr1 agonism and achieved a notable improvement in potency and efficacy compared to **4**. The smaller skeleton was not compatible with the original regiochemistry of the chloro substituent (**9**) and systematic deconstruction of **8** indicated a tight SAR with the importance of all substituents (**10**-**13**). Despite weak to no Nurr1 activation by **9**-**13**, isothermal titration calorimetry (ITC, Table 1, Supplementary Fig. 1) demonstrated that **11** and **12** containing the 8-chloro substituent still bound with low micromolar affinity to the Nurr1 LBD while **10** and **13** lacking the chlorine atom exhibited weaker binding. These results thus highlighted the 8-chloro substituent as a key factor driving affinity and the amino motif as relevant for Nurr1 activation. With single-digit micromolar potency (EC_50_ 7 µM) and affinity (K_d_ 2.7 µM, Fig. 2a), 8-chloro-2-methylimidazo[1,2-*a*]pyridine (**8**) emerged as an improved fragment-like Nurr1 agonist which was also evident from enhanced ligand efficiency (LE), lipophilic ligand efficiency (LLE), and size-independent ligand efficiency (SILE) compared to AQ and the lead fragment **3** (Table 2).

**Table 2:** Ligand efficiency metrics of 1, 4, 8, 24, 26 and 36.^a^

	1	4	8	24	26	36
pEC_50_	4.7	4.8	5.2	6.4	5.8	7.0
LE	0.26	0.50	0.59	0.46	0.31	0.34
LLE	-0.48	1.99	3.28	1.86	0.34	2.18
SILE	1.79	2.21	2.45	2.64	2.18	2.59

^a^Ligand efficiency (LE), lipophilic ligand efficiency (LLE), and size-independent ligand efficiency (SILE) were computed according to ref. ^62^.

Imidazopyridine-based NR4A modulators have been reported previously^39–41^, but independent evaluation by Munoz-Tello et al. ^19^ provided no evidence for binding of a selected example of this chemotype (SR24237)^40^ to Nurr1. For direct comparison, we profiled the previously reported imidazopyridine-based Nurr1 agonist SA00025 (EC_50_(NBRE) 0.217 µM^39^) in our test systems (refer to Supplementary Fig. 2 for assay setups; chemical structure and data for SA00025 in Supplementary Fig. 3). No activation of Nurr1 (or the related NR4A receptors Nur77 and NOR1) by SA00025 was detectable in the Gal4-hybrid reporter gene assay in the concentration range from 10 nM to 3 µM (Supplementary Fig. 3b). At higher concentration, SA00025 was considerably cytotoxic. As literature^39^ reported activation of full-length human Nurr1 by SA00025 on the NBRE response element, we also employed reporters for the human Nurr1 response elements (NBRE, NurRE, DR5)^14^ but detected no effect of SA00025 (Supplementary Fig. 3c). Orthogonal evaluation of SA00025 in cell-free setting by ITC indicated potential weak binding, but insufficient affinity to determine a K_d_ value with this technique (Supplementary Fig. 3d). In accordance with the findings of Munoz-Tello et al. ^19^, these results do not support potent Nurr1 agonism of SA00025 and related compounds and align with the lack of activity of **9** indicating that 8-substituted imidazopyridines like **8** are favored over analogues with substituents in 7-position.

Aiming to enhance potency by fragment growing, we next evaluated a potential optimization of the 2-methyl substituent of **8** (Table 3). Extension in this region caused a general reduction in Nurr1 activation efficacy. In a rough exploration of aliphatic (**14**) and aromatic (**15,** **16**) extensions, only the phenyl substituent (**15**) retained sufficient efficacy and provided a slight improvement over **8** in terms of potency (EC_50_ 4 µM). Chloro substituents in 4- (**17**) and 3- (**18**) positions of the phenyl motif enhanced activation efficacy but diminished potency. 2-Chloro substitution (**19**) disrupted activity on Nurr1. Replacement of the chloro substituents (**17,** **18**) by a methyl group was tolerated in 4-position (**20**) but not in 3-position (**21**), and trifluoromethyl substituents (**22,** **23**) also caused a marked drop in efficacy. Double 3,4-chloro substitution (**24**), in contrast, was additive and resulted in enhanced potency (EC_50_ 0.4 µM) and efficacy (1.8-fold activation). The corresponding dimethyl analogue **25** was less active.

**Table 3:** Extension of the imidazo[1,2-a]pyridine 8

ID	R^1^ =	EC_50_(Nurr1) (max. activation)^a^
**8**		7 ± 1 µM (2.0 ± 0.1-fold)
**14**		< 1.2-fold act.
**15**		4 ± 2 µM (1.4 ± 0.1-fold)
**16**		< 1.2-fold act.
**17**		15 ± 3 µM (1.7 ± 0.2-fold)
**18**		9 ± 3 µM (1.7 ± 0.1-fold)
**19**		inactive (100 µM)
**20**		12 ± 2 µM (2.0 ± 0.2-fold)
**21**		< 1.2-fold act.
**22**		< 1.2-fold act.
**23**		< 1.2-fold act.
**24**		0.4 ± 0.2 µM (1.8 ± 0.1-fold)
**25**		14 ± 2 µM (1.6 ± 0.1-fold)

^a^Nurr1 modulation was determined in a Gal4-Nurr1 hybrid reporter gene assay. Max. activation refers to the maximum effect vs. 0.1% DMSO control. Data are the mean ± SD; *n* ≥ 3.

Structural extension of the 2-methyl substituent in **8** thus provided no major gain in Nurr1 agonist activity. Only the 3,4-dichlorophenyl derivative **24** achieved a notable increase in potency over **8** which was in line with results from ITC confirming enhanced affinity of **24** (K_d_ 0.7 µM, Fig. 2b). In terms of ligand efficiency (Table 2), the structural extension from **8** to **24** represented no significant improvement but the 2-(3,4-dichlorophenyl) substituent of **24** may still be a valuable potency driving extension in fused derivatives.

The optimized Nurr1 agonist **8** obtained by fragment hopping from the chloroquinoline motif of AQ also appeared suitable for fragment growing by fusion with N-substituents. Our previous studies^37^ have revealed a 5-(4-chlorophenyl)furan-2-carboxamide residue as alternative motif to replace the aminophenol of AQ. Transfer of this SAR knowledge to the new imidazo[1,2-*a*]pyridine scaffold of **8** in the fused 5-(4-chlorophenyl)furan-2-carboxamide derivative **26** provided an improvement in Nurr1 agonist potency (Table 4) but decreased ligand efficiency (Table 2). Simplification of **26** by removal of the chloro substituent (**27**) was tolerated but among alternative central aromatic systems, only thiophene (**28**) retained Nurr1 agonism. Replacement of furan (**27**) by pyrrole (**29**) or benzene (**30**) disrupted activity thus suggesting **26** as lead for further optimization which was also supported by improved binding affinity of **26** (K_d_ 0.7 µM, Fig. 2c). Further optimization potential seemed to rest in the 4-chloro substituent as its removal (**27**) hardly diminished potency. This indicated that space to accommodate substituents was available in this region and we thus focused our attention on alternative motifs to replace the chlorine atom as 4-substituent of the phenylfuran-2-carboxamide residue (Table 5).

**Table 4:** Fusion of 8 with N-substituents

ID	R^2^ =	EC_50_(Nurr1) (max. activation)^a^
**8**		7 ± 1 µM (2.0 ± 0.1-fold)
**26**		1.6 ± 0.5 µM (1.8 ± 0.1-fold)
**27**		4 ± 1 µM (2.1 ± 0.1-fold)
**28**		3.2 ± 0.4 µM (2.1 ± 0.1-fold)
**29**		inactive (10 µM^b^)
**30**		inactive (10 µM^b^)

^a^Nurr1 modulation was determined in a Gal4-Nurr1 hybrid reporter gene assay. Max. activation refers to the maximum effect vs. 0.1% DMSO control. Data are the mean ± SD; *n* ≥ 3. ^b^Highest non-toxic concentration.

**Table 5:** Optimization of the fused derivative 26

ID	R^3^ =	EC_50_(Nurr1) (max. activation)^a^
**27**		4 ± 1 µM (2.1 ± 0.1-fold)
**26**		1.6 ± 0.5 µM (1.8 ± 0.1-fold)
**31**		1.1 ± 0.3 µM (1.8 ± 0.1-fold)
**32**		0.13 ± 0.02 µM (2.0 ± 0.2-fold)
**33**		0.8 ± 0.2 µM (2.0 ± 0.1-fold)
**34**		0.16 ± 0.06 µM (1.7 ± 0.1-fold)
**35**		0.30 ± 0.02 µM (1.8 ± 0.1-fold)
**36**		0.090 ± 0.005 µM (2.1 ± 0.1-fold)

^a^Nurr1 modulation was determined in a Gal4-Nurr1 hybrid reporter gene assay. Max. activation refers to the maximum effect vs. 0.1% DMSO control. Data are the mean ± SD; *n* ≥ 3.

The 4-trifluoromethyl derivative **31** exhibited similar activity as **26** while the corresponding 4-methyl analogue **32** was substantially more potent. A similar trend was observed for the 4-trifluoromethoxy (**33**) and 4-methoxy (**34**) pair, which indicated the potential relevance of inductive effects. Like 4-methyl (**32**) and 4-methoxy (**34**), a 4-methylamino substituent (**35**) was highly favored and enhanced Nurr1 agonist potency to a sub-micromolar range. A 4-dimethylamino group (**36**) provided a further improvement to a double-digit nanomolar EC_50_ value.

**Fig. 3:**
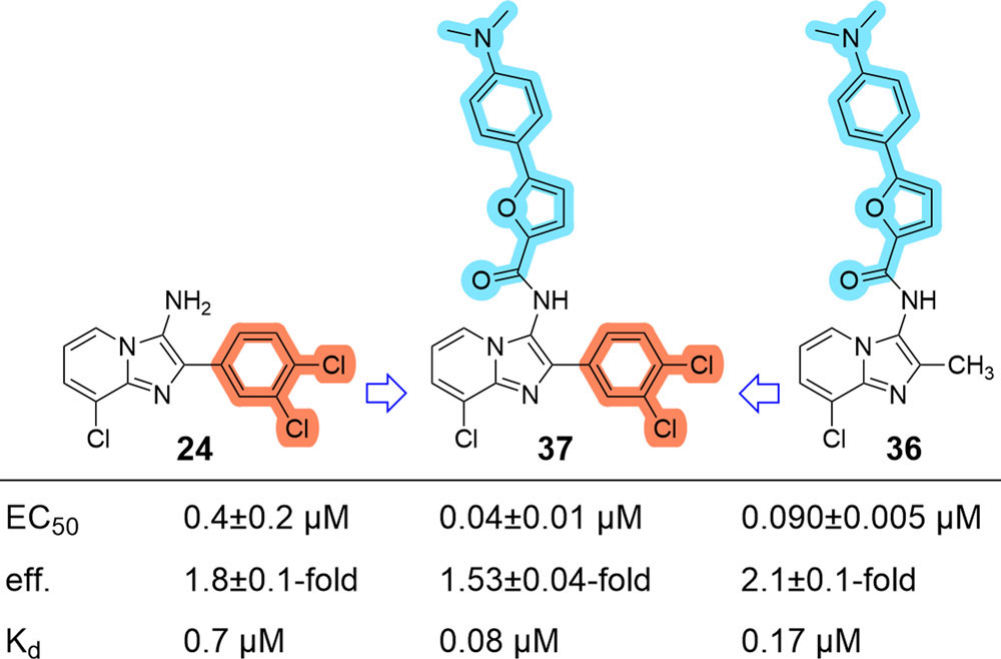
Structural fusion of 24 and 36 in 37 enhanced Nurr1 agonist potency and binding affinity. Nurr1 modulation was determined in a Gal4-Nurr1 hybrid reporter gene assay. Max. activation refers to the maximum effect vs. 0.1% DMSO control. Data are the mean ± SD; *n* ≥ 3. Binding affinity was determined by ITC.

The SAR evaluation had revealed favorable contributions to Nurr1 agonist potency for a 3,4-dichlorophenyl substituent in 2-position of the imidazopyridine scaffold (**24**) and for the 5-(4-(dimethylamino)phenyl)furan-2-carboxamide motif in 3-position (**36**). Structural fusion of these modifications in the combined derivative **37** (Fig. 3) generated a high-affinity Nurr1 modulator (K_d_ 0.08 µM, Supplementary Fig. 4) with strong agonist potency (EC_50_ = 0.04 ± 0.01 µM), but the fused compound **37** did not substantially outmatch **36** and was significantly more lipophilic (**37**: SlogP 7.54).

### Biological profiling of the Nurr1 agonist 36

**Fig. 4:**
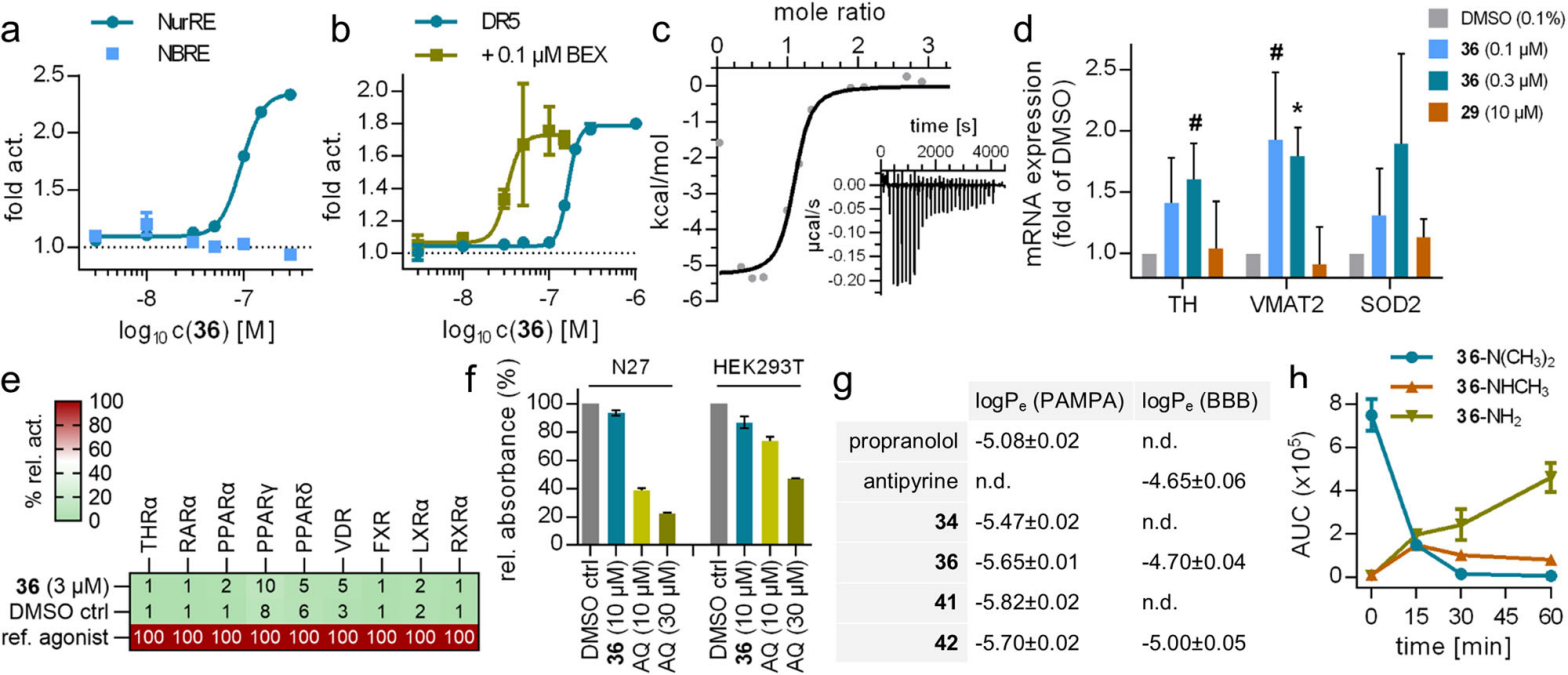
Orthogonal validation and profiling of 36. **a** Effects of **36** on the human full-length Nurr1 homodimer (NurRE) and monomer (NBRE). Data are the mean±S.E.M. fold activation vs. DMSO ctrl; *n* ≥ 3. **b** Effects of **36** on the Nurr1-RXR heterodimer (DR5) in the absence and presence of bexarotene (0.1 µM). Data are the mean±S.E.M. fold activation vs. DMSO ctrl or vs. 0.1 µM BEX; *n* ≥ 3. **c** Binding of **36** to the Nurr1 LBD (K_d_ 0.17 µM, *n* = 1.0) determined by ITC. The fitting of the heat of binding is shown and the isotherm at 25 °C is shown as inset. **d** Effects of **36** and **29** on the expression of the Nurr1-regulated tyrosine hydroxylase (TH), vesicular amino acid transporter 2 (VMAT2), and superoxide dismutase 2 (SOD2) in astrocytes (T98G). Data are the mean±S.E.M. fold mRNA induction vs. DMSO control; *n* = 3; ^#^*p* < 0.1, *p < 0.05 (t-test vs. DMSO ctrl). **e** Selectivity screening of **36** on nuclear receptors. Heatmap shows the mean relative activation compared to reference ligands (listed in the methods section); *n* = 3. (**f**) **36** (10 µM) had no toxic effect in a WST-8 assay in N27 rat neurons and HEK293T cells. AQ (**1**, 10 and 30 µM) was toxic. Data are the mean±S.E.M. rel. absorbance (450 nm); *n* ≥ 3. **g** Permeability of Nurr1 agonists in a parallel artificial membrane permeability assay (PAMPA) and in a cellular model of the blood-brain-barrier (BBB). Propranolol and the brain-penetrant reference antipyrine for comparison. Data are the mean ± SD; *n* = 6. **h** Metabolism of **36** by rat liver microsomes resulted in demethylation of the dimethylamino group. Data are the mean ± SD; *n* = 4.

These results highlighted **36** as preferred Nurr1 agonist from the scaffold hopping and fragment growing approach. Potent Nurr1 agonism of **36** was also evident in reporter gene assays to observe the activity of the full-length human Nurr1 (Fig. 4a, b) which better reflects the physiological setting than the hybrid reporter gene assay (cf. Supplementary Fig. 2). Nurr1 can act as a monomer, homodimer, and RXR heterodimer on different response elements^14^. **36** robustly activated the Nurr1 homodimer (NurRE, EC_50_ 0.094 µM; Fig. 4a) and the Nurr1-RXR heterodimer (DR5, EC_50_ 0.165 µM; Fig. 4b) but interestingly was inactive on the Nurr1 monomer (NBRE; Fig. 4a) indicating an unprecedented Nurr1 dimer preference. The RXR agonist bexarotene caused generally reduced activity of DR5 (Supplementary Fig. 5) but enhanced the potency of **36** by a factor of >5 (EC_50_ 0.032 µM (DR5 w. 0.1 µM BEX)) suggesting potentially cooperative binding^42^ to and activation of the Nurr1-RXR heterodimer.

**36** exhibited high-affinity binding (K_d_ 0.17 µM) to the Nurr1 LBD in ITC (Fig. 4c) orthogonally validating its potent Nurr1 agonism. In astrocytes (T98G), **36** induced expression of the Nurr1-regulated genes tyrosine hydroxylase (TH), vesicular amino acid transporter 2 (VMAT2), and superoxide dismutase 2 (SOD2) at low concentrations (0.1 µM, 0.3 µM) supporting cellular target engagement (Fig. 4d). Selectivity profiling revealed a preference of **36** for Nurr1 over Nur77 (Table 6) and no activity outside the NR4A family at 3 µM corresponding to >30-fold selectivity (Fig. 4e). Moreover, **36** was non-toxic in N27 rat neurons, which can be used for neurodegeneration models^36,43^, and in HEK293T cells at 10 µM which is two orders of magnitude above its EC_50_ value (Fig. 4f). AQ, in contrast, exhibited considerable toxicity with only ~20% remaining N27 metabolic activity at a concentration (30 µM) that may be considered necessary for confident target engagement given its low potency. With its favorable profile fulfilling the highest quality criteria for chemical tools^44,45^, **36** thus emerges as a next-generation tool to study the effects of Nurr1 modulation by AQ-type ligands.

**Table 6:** Characterization of NR4A agonist 36 and negative control 29 demonstrating high chemical tool quality^44,45^

		
	**36**	**29**
EC_50_(Nurr1)	0.090 ± 0.005 µM	no activation (10 µM)
EC_50_(Nur77)	0.33 ± 0.04 µM	no activation (10 µM)
EC_50_(NOR-1)	0.11 ± 0.03 µM	no activation (10 µM)
EC_50_(NBRE)	no activation (1 µM)	no activation (10 µM)
EC_50_(NurRE)	0.094 ± 0.003 µM	no activation (10 µM)
EC_50_(DR5) [+0.1 µM BEX]	0.165 ± 0.004 µM [0.032 ± 0.007 µM]	no activation (10 µM)
K_d_(Nurr1 LBD)	0.17 µM	no binding^b^
NR selectivity^c^	inactive (3 µM)	n.d.
toxicity^d^	inactive (10 µM)	inactive (10 µM)
aq. solubility	6.8 mg/L	4.1 mg/L
SlogP^e^	4.87	4.54

^a^Nurr1 modulation was determined in a Gal4-Nurr1 hybrid reporter gene assay. Max. activation refers to the maximum effect vs. 0.1% DMSO control. Data are the mean ± SD; *n* ≥ 3. ^b^No binding observable in ITC with 100 µM **29** and 30 µM protein (Supplementary Fig. 6). ^c^Nuclear receptor selectivity was determined in Gal4-hybrid reporter gene assays for THRα, RARα, PPARα/γ/δ, VDR, FXR, LXRα and RXRα (Fig. 4e). ^d^Cytotoxicity was evaluated in N27 (**36**) and HEK293T (**29,** **36**) cells using a WST-8 assay (Fig. 4f). ^e^SlogP was computed with RDKit^63^ software.

To boost the value of **36** as a chemical tool, we aimed to complement it with a structurally matched negative control compound for which **29** appeared suitable. **29** strongly resembles **36** in its chemical structure and physicochemical characteristics but exhibited no Nurr1 agonism in a cellular setting at 10 µM and revealed no detectable binding to Nurr1 in ITC (Table 6). Further profiling of **29** revealed no effect on Nur77 and NOR-1, no activation of full-length Nurr1, and no induction of Nurr1-regulated gene expression (Fig. 4d). Thus, **29** is at least 100-fold less active than **36** as Nurr1 modulator and suitable as negative control.

To further explore the potential of **36** as a chemical tool for in vivo applications, we evaluated its pharmacokinetic parameters. **36** exhibited favorable permeability in a parallel artificial membrane permeability assay (PAMPA) and a cellular model of the blood-brain barrier using the human brain endothelial cell-line HBEC-5i on trans-well plates^46^ (Fig. 4g) as indicators of good absorption and brain penetration. The stability of **36** against degradation by rat liver microsomes was low, however, pointing to limited metabolic stability (Fig. 4h). Closer inspection suggested that **36** was almost exclusively degraded by demethylation of the dimethylamino motif. The initially formed monomethylamine (**35**) retained slightly reduced Nurr1 agonism (Table 5) while the eventually dominant fully demethylated amine **38** was less active (Table 7). We aimed to overcome the metabolic liability of **36** and obtain an analogue suitable for in vivo applications and hence probed replacement of the labile motif (Table 7). The introduction of cyclic amino substituents (**39,** **40**) was not productive as Nurr1 agonism was lost with these bulkier residues. Therefore, we moved our attention to the methoxy analogue **34**, which exhibited similar Nurr1 agonist potency as the labile dimethylamine **36** and threefold higher stability against microsomal degradation. Extension of the methoxy group of **34** to an isopropyloxy (**41**) or cyclopropyloxy (**42**) motif was well tolerated in terms of Nurr1 agonism and significantly improved metabolic stability while mostly retaining membrane and BBB permeability (Fig. 4g). These results, therefore, demonstrate that the pharmacokinetic profile of the new Nurr1 agonist scaffold can be tuned to obtain candidates for in vivo application.

**Table 7:** Optimization of 36 for microsomal stability

ID	R^3^ =	EC_50_(Nurr1) (max. act.)^a^	microsomal half-life^b^
**36**		0.090 ± 0.005 µM (2.1 ± 0.1-fold)	6.3 ± 0.3 min.
**38**		<1.2-fold activation	n.d.
**39**		inactive (10 µM^c^)	n.d.
**40**		inactive (10 µM^d^)	n.d.
**34**		0.16 ± 0.06 µM (1.7 ± 0.1-fold)	18 ± 2 min.
**41**		0.12 ± 0.03 µM (2.1 ± 0.2-fold)	42 ± 5 min.
**42**		0.12 ± 0.06 µM (1.5 ± 0.2-fold)	78 ± 7 min.

^a^Nurr1 modulation was determined in a Gal4-Nurr1 hybrid reporter gene assay. Max. activation refers to the maximum effect vs. 0.1% DMSO control. Data are the mean ± SD; *n* ≥ 3. ^b^Stability against degradation by rat liver microsomes was determined by LCMS. n.d. - not determined. ^c^Highest non-toxic concentration.

### The Nurr1 agonist 36 rescued tyrosine hydroxylase expression in LRRK2 mutant midbrain organoids

**Fig. 5:**
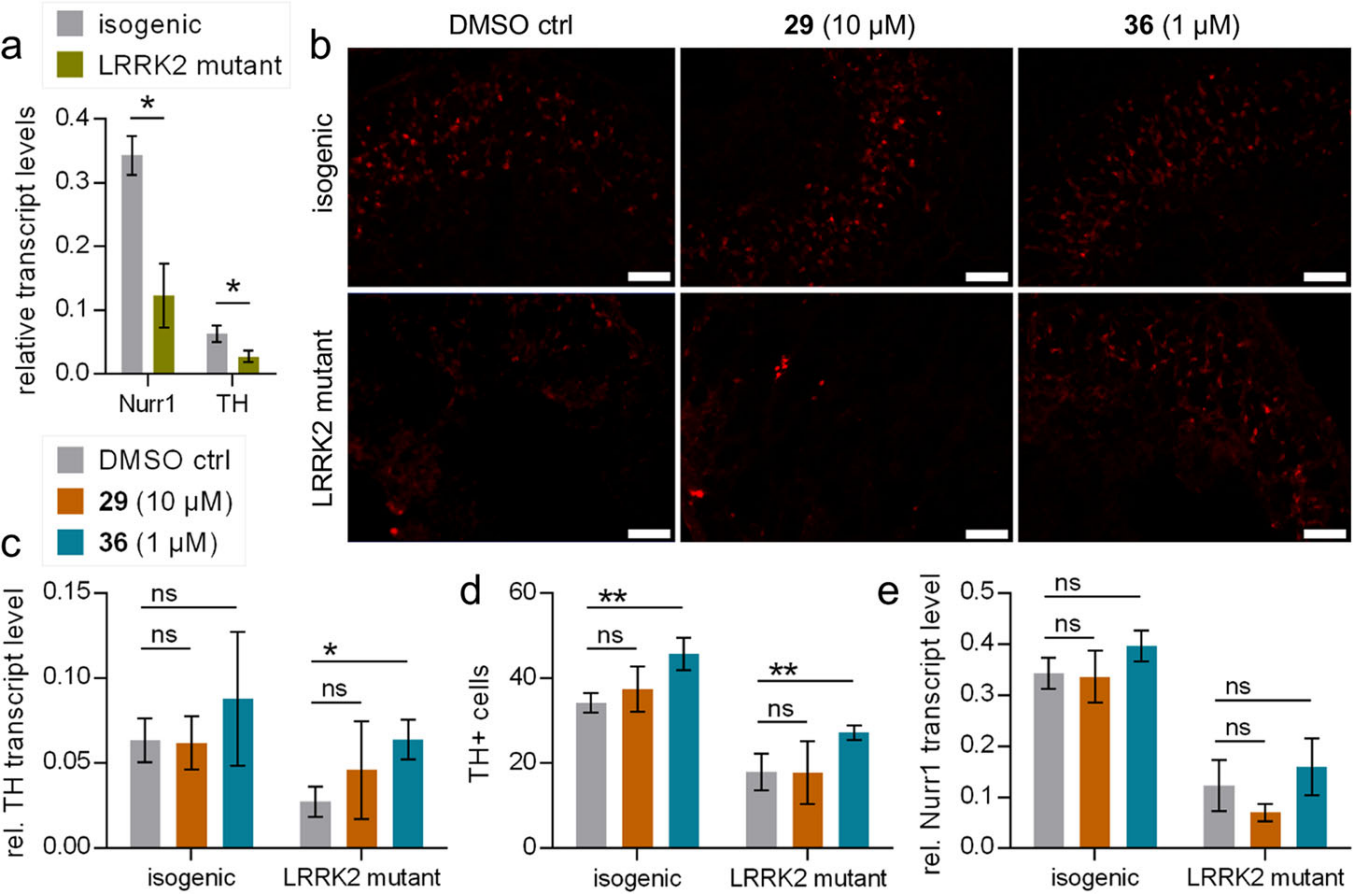
Treatment of midbrain organoids with the Nurr1 agonist 36 increased the number of TH positive cells. **a** Midbrain organoids generated from human iPSC bearing a gain-of-function LRRK2 mutation (G2019S) displayed diminished Nurr1 and TH transcript levels compared to isogenic controls after 45 days. **b** Midbrain organoid sections stained for TH expressing cells by immunofluorescence. Scale bars represent 100 µm. **c** The Nurr1 agonist **36** but not the negative control **29** induced TH mRNA expression in LRRK2 mutant organoids and tended to enhance TH mRNA expression in isogenic controls. **d** The Nurr1 agonist **36** but not the negative control **29** enhanced the number of TH positive cells in LRRK2 mutant organoids and in isogenic controls. **e** Compound treatment did not affect Nurr1 levels. All data are the mean ± SD; *n* = 3. *p < 0.05, **p < 0.01 (unpaired, two-tailed Student’s *t*-test).

To evaluate the biological effects of the new Nurr1 agonist scaffold in a more (patho-)physiological setting and capture its full potential as a chemical tool and lead, we employed a PD model in a three-dimensional organoid system^47^ derived from induced pluripotent stem cells (iPSC). Human midbrain organoids were generated from wildtype iPSC (isogenic control) and from iPSC bearing a G2019S mutation in the leucine-rich repeat kinase 2 (LRRK2) gene. LRRK2 mutation is among the most common genetic causes of PD^48^ and the G2019S mutation enhancing the kinase activity of LRRK2 has been correlated with increased α-synuclein accumulation, mitochondrial dysfunction, impaired dopamine signaling, and ultimately progressive dopamine neuron loss in the human brain^48,49^. Using this in vitro disease model, we evaluated the impact of **36** and **29** on the dopamine neuron marker TH in 44 days old organoids (Fig. 5). Compared to the isogenic control, LRRK2 mutant organoids exhibited significantly diminished Nurr1 and TH mRNA expression (Fig. 5a, b). Treatment with the Nurr1 agonist **36** rescued the TH levels in LRRK2 mutant organoids as evident in mRNA transcript level (Fig. 5c) and histologically by staining for TH (Fig. 5b, d). TH transcript levels of mutant organoids treated with **36** reached the level of isogenic control organoids further supporting the therapeutic potential of Nurr1 activation in PD. A weaker efficacy of **36** on TH levels in wild-type organoids may indicate that Nurr1 activity in these organoids was sufficient and that therapeutic Nurr1 activation may unfold stronger when Nurr1 activity is pathologically diminished. Importantly, Nurr1 levels were not altered by treatment with **36** (Fig. 5e) underscoring that its effects on TH expression were mediated by direct Nurr1 activation. Compound **29** did not affect TH and Nurr1 levels in organoids supporting its suitability as negative control.

## Conclusion

Nurr1 is attracting remarkable interest as a candidate target for neurodegenerative disease treatment^12^. However, the therapeutic potential of the nuclear receptor is mainly supported by knockout studies and observations from patients^12^, and target validation with high-quality chemical tools is pending. AQ was discovered as a direct Nurr1 modulator and used in several pharmacological studies^24,25^ but is only a weak Nurr1 agonist and exhibits unspecific effects^19^ disqualifying the antimalarial as a chemical tool. Despite recent progress with an optimized Nurr1 agonist providing further evidence for the therapeutic value of Nurr1 activation in PD models^36^, the promising effects observed with AQ require validation. Here, we have developed potent Nurr1 agonists from AQ with extensively validated activity and lacking the structural elements of AQ mediating unspecific toxicity to enable robust biological studies on Nurr1 modulation with AQ-derived agonists. Scaffold-hopping, fragment optimization, and replacement of unfavorable motifs generated potent Nurr1 agonists with preferable chemical features and experimentally confirmed high-affinity binding to the Nurr1 LBD. **36** and the structurally matched negative control **29** fulfil the highest quality criteria^44^ as a next-generation chemical tool for biological studies on Nurr1 to advance Nurr1 modulation as a therapeutic strategy in neurodegeneration and beyond.

## Chemistry

**Fig. 6:**
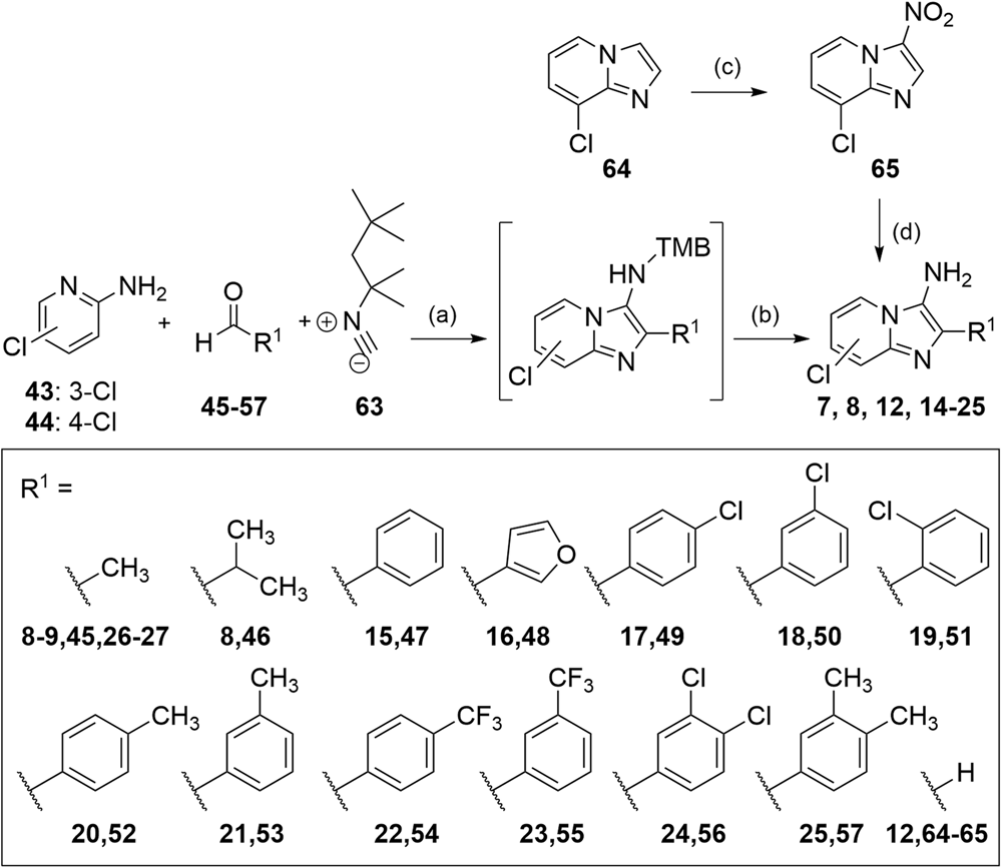
Synthesis of 7, 8, 12, 14-24, 26 and 27. Reagents & Conditions: **a** AcOH, MeOH, rt, 24-48 h; **b** TFA/DCM, rt, 30 min to overnight, 3-30% over two steps; **c** nitric acid, sulfuric acid, 0 °C – rt, 1 h, 93%; **d** iron, ammonium chloride, MeOH/water, reflux, overnight, 57%.

Compounds **7,** **8,** **12** and **14**-**24** were prepared by Groebke-Blackburn reaction according to Fig. 6. 3-Chloropyridine-2-amine (**43**) and 4-chloropyridine-2-amine (**44**) were cyclized with the aldehydes **45**-**57** and 1,1,3,3-tetramethylbutylisocyanide followed by acid-mediated cleavage to **7,** **8** and **14-24** using 4 N HCl in dioxane or TFA/CH_2_Cl_2_ (1:1). 1,1,3,3-Tetramethylbutylisocyanide (**63**) was commercially available. 3-Amino-8-chloroimidazo[1,2-*a*]pyridine (**12**) was prepared by nitration of 8-chloroimidazo[1,2-*a*]pyridine (**64**) followed by reduction with iron.

**Fig. 7:**
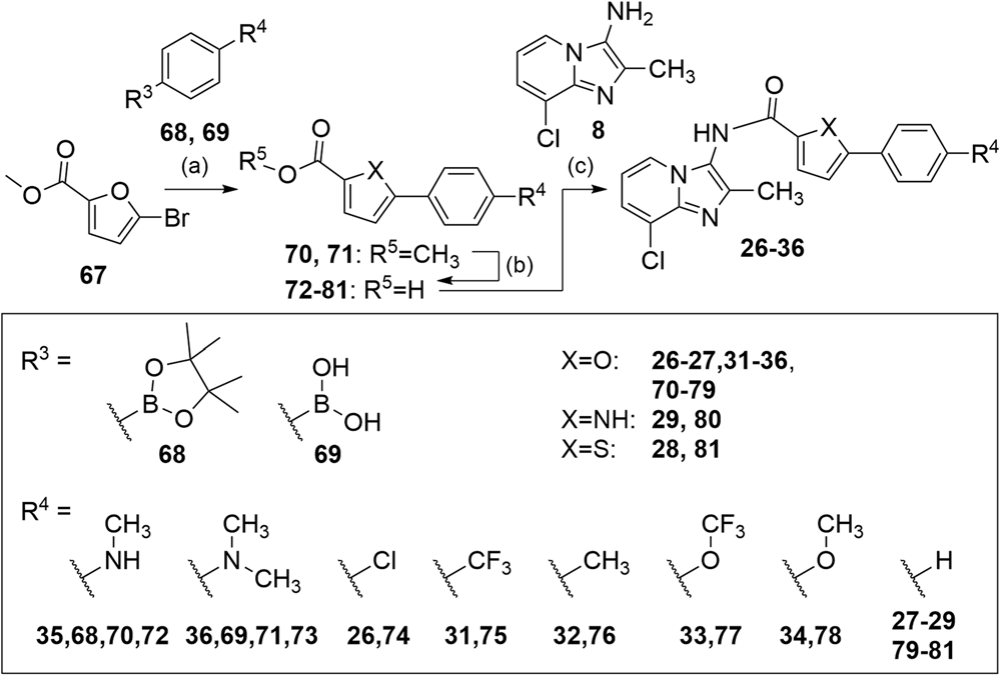
Synthesis of 26-36. Reagents & Conditions: **a** XPhos Pd G2, K_3_PO_4_, water/dioxane, reflux, overnight, 86-97%; **b** LiOH, water/THF, rt, overnight, 67–73%; **c** HATU, DIPEA, DMF, rt, overnight, 33-100% or SOCl_2_, CHCl_3_ reflux, 3 h, 11%.

Compounds **26-36** were obtained by amide coupling of 3-amino-8-chloro-2-methylimidazo[1,2-*a*]pyridine (**8**) and the carboxylic acids **72**-**81** using HATU or thionyl chloride (Fig. 7). The carboxylic acids **72** and **73** were prepared by Suzuki coupling of aryl bromide **67** with the boronates **68,** **69** to **70,** **71** followed by alkaline ester hydrolysis. **74**-**81** were commercially available.

**Fig. 8:**
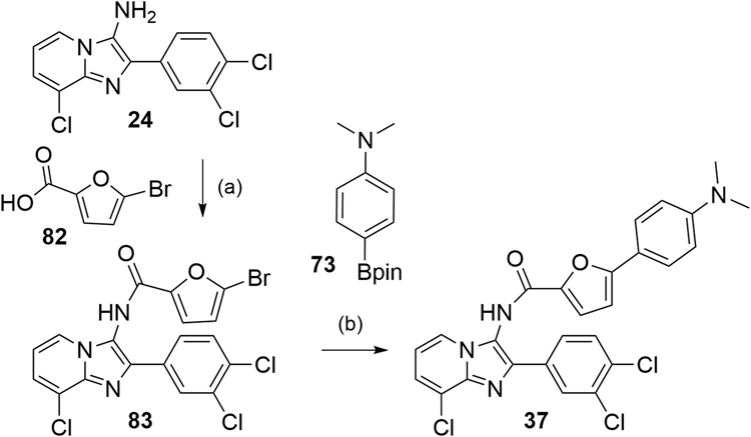
Synthesis of 37. Reagents & Conditions: **a** oxalyl chloride, pyridine, toluene, 0 °C - rt, 24 h, 64%; **b** tetrakis(triphenylphosphane)palladium(0), Na_2_CO_3_, water/dioxane, reflux, overnight, 68%.

**Fig. 9:**
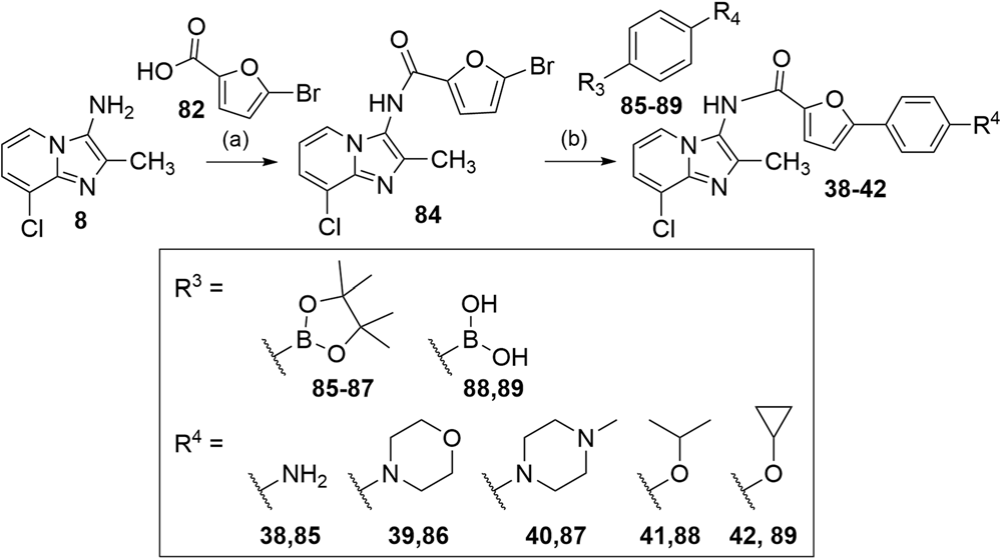
Synthesis of 38-42. Reagents & Conditions: **a** HATU, DIPEA, DMF, rt, overnight, 81%; **b** tetrakis(triphenylphosphane)palladium(0), Na_2_CO_3_, water/dioxane, reflux, overnight, 40–68%.

The fused derivative **37** was obtained from **24** via amide coupling with 5-bromofuran-2-carbonyl chloride (**82**) and subsequent Suzuki-Miyaura reaction with **73** according to Fig. 8. Similarly, **38-42** were synthesized from 3-amino**-**8-chloro-2-methylimidazo[1,2-*a*]pyridine (**8**) by amide coupling with 5-bromofuran-2-carboxylic acid (**81**) to **84** and Suzuki-Miyaura coupling with the boronates **85-89** (Fig. 9).

## Experimental procedures

### Chemistry

#### General

All chemicals were of reagent grade, purchased from commercial sources (e.g., Sigma-Aldrich, TCI, BLDpharm), and used without further purification unless otherwise specified. All reactions were conducted under a nitrogen or argon atmosphere and in absolute solvents purchased from Sigma-Aldrich. Other solvents, especially for work-up procedures, were of reagent grade or purified by distillation (*iso*-hexane, cyclohexane, ethyl acetate, EtOH). Reactions were monitored by thin layer chromatography (TLC) on TLC Silica gel 60 F254 coated aluminum sheets by Merck and visualized under ultraviolet light (254 nm) or by using ninhydrin or Ehrlichs reagent stains. Purification by column chromatography was performed on a puriFlash® XS520Plus system (Advion, Ithaca, NY, USA) using high-performance spherical silica columns (SIHP, 50 µm) by Interchim and a gradient of *iso*-hexane or cyclohexane to ethyl acetate, Reversed-phase column chromatography was performed on a puriFlash® 5.250 system (Advion) using C18HP columns (SIHP, 15 µm) by Interchim and a gradient of H_2_O with 10% MeCN to 100% MeCN (HPLC gradient grade). Mass spectra were obtained on a puriFlash®-CMS system (Advion) using atmospheric pressure chemical ionization (APCI). HRMS were obtained with a Thermo Finnigan LTQ FT instrument for electron impact ionization (EI) or electrospray ionization (ESI). NMR spectra were recorded on Bruker Avance III HD 400 MHz or 500 MHz spectrometers equipped with a CryoProbeTM Prodigy broadband probe (Bruker). Chemical shifts are reported in δ values (ppm) relative to residual protium signals in the NMR solvent (^1^H-NMR: acetone-*d*_*6*_: δ = 2.04 ppm; DMSO-*d*_*6*_: δ = 2.50 ppm; MeOD-*d*_*4*_: δ = 3.31 ppm, ^13^C-NMR: acetone-*d*_*6*_: δ = 206.26, 29.84 ppm; DMSO-*d*_*6*_: δ = 39.52 ppm; MeOD-*d*_*4*_:δ = 49.0 ppm), coupling constants (*J*) in hertz (Hz). The purity of the compounds was determined by ^1^H NMR (qHNMR) according to the method described by Pauli et al.^50^ with internal calibration. To ensure accurate determination of peak area ratio, the qHNMR measurements were conducted under conditions allowing for complete relaxation. Ethyl 4-(dimethylamino)benzoate (LOT#BCCC6657, purity 99.63%), dimethyl terephthalate (LOT#BCBT9974, purity 99.95%) and maleic acid (LOT#BCBM8127V, purity 99.94%) were used as internal standards in MeOD-*d*_4_, DMSO-*d*_6_, or acetone-*d*_6_. All compounds for biological testing had a purity >95% according to quantitative ^1^H NMR (qHNMR).

#### General procedure A for Groebke-Blackburn-Bienaymé reaction and hydrolysis

2-Amino-3-chloropyridine (**43**, 1.0 eq.) or 2-amino-4-chloropyridine (**44**, 1.0 eq.), the respective aldehyde (**45**-**57**, 1.0–1.1 eq.) and glacial acetic acid (1.5 eq.) were dissolved in dry methanol (0.7 M) under nitrogen atmosphere. The mixture was stirred for 30–45 min. at room temperature (rt) for imine formation. 1,1,3,3-Tetramethylbutylisocyanid (**63**, 1.5 eq.) was subsequently added, and the mixture was stirred at rt for 17–48 h. When TLC monitoring indicated completion, the isocyanide was quenched by the addition of 2N aqueous HCl (2 mL) and further stirring for 30 min. 2 N aqueous NaOH solution and ethyl acetate (10 mL) were then added, and the phases were separated. The aqueous layer was extracted with ethyl acetate (3x). The combined organic layers were dried over MgSO_4_ and the solvent was evaporated under reduced pressure. The crude product was dissolved in a mixture of CH_2_Cl_2_ and trifluoroacetic acid (10 mL, 1:1) or 4 N HCl in dioxane (10 mL) and the mixture was stirred for 30–60 min. at rt. When TLC monitoring indicated completion, 2 N aqueous NaOH solution was added, phases were separated, and the aqueous layer was extracted with ethyl acetate (3x). The combined organic layers were dried over MgSO_4_ and the solvents were evaporated under reduced pressure. The crude product was purified by flash column chromatography using a gradient of *iso*-hexane or cyclohexane/ethyl acetate as mobile phase, and potentially by reverse phase chromatography using a gradient of H_2_O with 10% MeCN to 100% MeCN (HPLC gradient grade).

#### General procedure B for amide coupling with HATU

The respective carboxylic acid (**72-80**, 1.2 eq.) and 1-[bis(dimethylamino)methylene]-1*H*-1,2,3-triazolo[4,5-*b*]pyridinium 3-oxide hexafluorophosphate (HATU, 1.2 eq.) were dissolved in DMF (0.13 M). *N-*Ethyldiisopropylamine (DIPEA, 0.13 M, 1.2 eq) was added and the mixture was stirred at rt for 40 min. 3-Aminoimidazo[1,2-*a*]pyridine (**8**, 1.0 eq.) was dissolved in DMF (0.11 M) and added to the activated carboxylic acid. The mixture was stirred at rt overnight. When TLC monitoring indicated completion, the solvent was removed under reduced pressure, the residue was dissolved in ethyl acetate and treated with 5% HCl (0.13 M). Phases were separated and the aqueous layer was extracted with ethyl acetate (3x). The combined organic layers were washed with 1 N aqueous NaOH solution and dried over MgSO_4_. The solvent was evaporated under reduced pressure and the crude product was purified by flash column chromatography using a gradient of *iso*-hexane / ethyl acetate as mobile phase, and potentially by reverse phase chromatography using a gradient of H_2_O with 10% MeCN to 100% MeCN (HPLC gradient grade).

#### 8-Chloro-2-methylimidazo[1,2-*a*]pyridine-3-amine (**8**)

Preparation according to general procedure A using 2-amino-3-chloropyridine (**43**, 821 mg, 6.39 mmol, 1.00 eq) and acetaldehyde (**45**, 39 µL, 7.03 mmol, 1.10 eq). **8** was obtained as a colorless solid (344 mg, 30%). ^1^H-NMR (400 MHz, acetone-*d*_6_): δ = 8.05 (dd, *J* = 6.8, 1.1 Hz, 1H), 7.11 (dd, *J* = 7.3, 1.0 Hz, 1H), 6.76 (t, *J* = 7.0 Hz, 1H), 4.17 (s, 2H), 2.33 (s, 3H). ^13^C-NMR (101 MHz, acetone-*d*_6_): δ = 136.1, 130.1, 126.3, 121.5, 121.0, 119.8, 110.0, 11.9. MS (APCI+): *m/z* 181.9 ([M + H]^+^). HRMS (EI+): *m/z* calculated 181.0407 for C_8_H_8_ClN_3_, found 181.0400 ([M + H]^+^). qHNMR (400 MHz, acetone-*d*_6_, ethyl-4(dimethylamino)benzoate as reference): purity = 97.5%.

#### 8-Chloro-2-(3,4-dichlorophenyl)imidazo[1,2-*a*]pyridine-3-amine (**24**)

Preparation according to general procedure A using 2-amino-4-chlorpyridine (**43**, 1.29 g, 10.0 mmol, 1.00 eq) and 3,4-dichlorobenzaldehyde (**56**, 1.93 g, 11.0 mmol, 1.01 eq). **24** was obtained as a yellow solid (197 mg, 6%). ^1^H-NMR (400 MHz, acetone-*d*_6_): δ = 8.41–8.35 (m, 1H), 8.30 – 8.23 (m, 1H), 8.20 – 8.12 (m, 1H), 7.630–7.56 (m, 1H), 7.27 (dd, *J* = 7.3, 1.1 Hz, 1H), 6.88 (t, *J* = 7.1 Hz, 1H), 4.83 (s, 2H).^13^C-NMR (101 MHz, MeOD-*d*_4_): δ = 137.2, 134.4, 132.1, 130.1, 130.0, 128.6, 128.0, 127.5, 126.3, 122.6, 121.7, 121.3, 111.3. MS (APCI+): *m/z* 311.9 ([M + H]^+^). HRMS (EI+): *m/z* calculated 310.9784 for C_13_H_8_Cl_3_N_3_, found 310.9778 ([M]•^+^). qHNMR (400 MHz, acetone-*d*_6_, ethyl-4-(dimethylamino)benzoate as reference) purity = 95.7%.

#### *N*-(8-Chloro-2-methylimidazo[1,2-*a*]pyridin-3-yl)-5-(4-(dimethylamino)phenyl)furan-2-carboxamide (**36**)

Preparation according to general procedure B using 8-chloro-2-methylimidazo[1,2-*a*]pyridine-3-amine (**8**, 50 mg, 0.28 mmol, 1.00 eq) and 5-(4-(dimethylamino)phenyl)furan-2-carboxylic acid (**73**, 76 mg, 0.33 mmol, 1.20 eq). **36** was obtained as a yellow solid (69 mg, 64%). ^1^H-NMR (400 MHz, acetone-*d*_6_): δ = 9.68 (s, 1H), 8.11 (d, *J* = 1.0 Hz, 1H), 7.77 (d, *J* = 8.5 Hz, 2H), 7.40 – 7.27 (m, 2H), 6.94 – 6.73 (m, 4H), 3.00 (s, 6H), 2.36 (s, 3H). ^13^C-NMR (101 MHz, acetone-*d*_6_): δ = 157.7, 157.2, 151.0, 145.1, 139.1, 138.7, 125.9, 122.8, 122.6, 121.8, 118.0, 117.6, 117.0, 111.99, 111.0, 104.3, 39.4, 12.4. MS (APCI+): *m/z* 394.7 ([M + H]^+^). HRMS (EI+): *m/z* calculated 394.1197 for C_21_H_19_ClN_4_O_2_, found 394.1194 ([M]•^+^). qHNMR (400 MHz, acetone-*d*_6_, maleic acid as reference) purity = 95.1%.

#### *N*-(8-Chloro-2-methylimidazo[1,2-*a*]pyridin-3-yl)-5-phenyl-1*H*-pyrrole-2-carboxamide (**29**)

5-Phenyl-1*H*-pyrrole-2-carboxylic acid (**80**, 74.0 mg, 396 µmol, 1.20 eq) was refluxed in thionyl chloride (4 mL) for 3 h. After cooling to rt, remaining thionyl chloride was removed under reduced pressure. 8-Chloro-2-methylimidazo[1,2-*a*]pyridine-3-amine (**8**, 60.0 mg, 330 µmol, 1.00 eq) dissolved in chloroform (5 mL) was added to the crude acyl chloride at 0 °C and the mixture was stirred at rt overnight. The solvent was removed under reduced pressure, the residue was dissolved in ethyl acetate and treated with 5% HCl (5 mL). Phases were separated, the aqueous layer was extracted with ethyl acetate (3x), and the combined organic layers were washed with 1 N aqueous NaOH solution and dried over MgSO_4_. The solvent was removed under reduced pressure and the crude product was purified by flash column chromatography and reverse column chromatography giving compound **29** as a colorless solid (13 mg, 11%). ^1^H-NMR (400 MHz, acetone-*d*_*6*_): δ = 11.85 (s, 1H), 10.51 (s, 1H), 8.31 (d, *J* = 6.7 Hz, 1H), 7.98 (d, *J* = 7.5 Hz, 2H), 7.63 (d, *J* = 7.4 Hz, 1H), 7.41 (t, *J* = 7.7 Hz, 2H), 7.34–7.23 (m, 1H), 7.21–7.06 (m, 2H), 6.75–6.63 (m, 1H), 2.44 (s, 3H). ^13^C-NMR (101 MHz, acetone-*d*_*6*_): δ = 159.6, 154.3, 154.1, 137.4, 136.7, 131.9, 128.7, 127.2, 126.2, 126.0, 125.0, 123.7, 118.9, 114.9, 113.1, 107.3, 11.2. qHNMR (400 MHz, acetone-*d*_*6*_, dimethyl terephthalate as reference): purity = 97.1%. MS (APCI+): *m/z* 350.3 ([M]^+^). HRMS (ESI+): *m/z* calculated 351.0007 for C_19_H_16_ClN_4_O^+^, found 351.10048 ([M + H]^+^).

#### *N*-(8-Chloro-2-(3,4-dichlorophenyl)imidazo[1,2-*a*]pyridin-3-yl)-5-(4-(dimethylamino)phenyl)furan-2-carboxamidearboxamide (**37**)

*N,N-*Dimethyl-4-(4,4,5,5-tetramethyl-1,3,2-dioxaborolan-2-yl)aniline (**69**, 40.8 mg, 0.165 mmol, 1.00 eq), 5-bromo-*N*-(8-chloro-2-(3,4-dichlorophenyl)imidazo[1,2-*a*]pyridin-3-yl)furan-2-carboxamide (**84**, 80.0 mg, 0.165 mmol, 1.00 eq) and sodium carbonate (52.5 mg, 3.00 mol, 3.00 eq) were dissolved in dioxane/H_2_O (10 mL, 9:1). The solution was degassed by freeze-pump-thaw cycles (3x). Pd(PPh_3_)_4_ (9.53 mg, 8.25 µmol, 0.05 eq) was added and the mixture was refluxed for 3 h under argon atmosphere. The resulting suspension was filtered, and the precipitate was washed with 2 N aqueous NaOH solution, EtOH, methylene chloride, and brine giving **37** as a colorless solid (59 mg, 68%). ^1^H-NMR (400 MHz, DMSO-*d*_*6*_): δ = 10.86 (s, 1H), 8.27–8.17 (m, 2H), 8.02–7.94 (m, 1H), 7.85–7.72 (m, 3H), 7.60 (d, *J* = 7.3 Hz, 1H), 7.57–7.50 (m, 1H), 7.02–6.92 (m, 2H), 6.80 (d, *J* = 8.8 Hz, 2H), 2.98 (s, 6H). ^13^C-NMR (126 MHz, DMSO-*d*_*6*_): δ = 158.0, 151.1, 144.5, 139.7, 136.5, 136.0, 134.1, 132.0, 131.6, 128.6, 127.1, 126.4, 125.6, 124.1, 122.0, 119.4, 117.3, 115.3, 113.0, 112.4, 110.4, 105.2, 31.2. qHNMR (400 MHz, DMSO-*d*_*6*_, maleic acid as reference): purity = 96.1%. MS (APCI+): *m/z* 524.2 ([M + H]•^+^). HRMS (ESI+): *m/z* calculated 525.0646 for C_26_H_20_Cl_3_N_4_O_2_^+^, found 525.0641 ([M + H]^+^).

#### *N*-(8-Chloro-2-methylimidazo[1,2-*a*]pyridin-3-yl)-5-(4-cyclopropoxyphenyl)furan-2-carboxamide (**42**)

4-Cyclopropyloxyphenylboronic acid (**89**, 30.1 mg, 169 mmol, 1.00 eq), 5-bromo-*N*-(8-chloro-2-methylimidazo[1,2-*a*]pyridin-3-yl)furan-2-carboxamide (**84**, 60.0 mg, 0.169 mmol, 1.00 eq) and sodium carbonate (53.7 mg, 3.00 mol, 3.00 eq) were dissolved in dioxane/ H_2_O (10 mL, 9:1). The solution was degassed by freeze-pump-thaw cycles (3x). Pd(PPh_3_)_4_ (9.76 mg, 8.45 µmol, 0.05 eq) was added and the mixture was refluxed for 3 h under argon atmosphere. The resulting suspension was filtered through Celite and the solvent was removed under reduced pressure. The residue was dissolved in 2 N aqueous NaOH solution and was extracted with ethyl acetate (3x). The combined organic layers were dried over MgSO_4_ and the solvent was removed under reduced pressure. The crude product was purified by flash column chromatography and reverse column chromatography giving **42** as a colorless solid (45 mg, 65%). ^1^H-NMR (400 MHz, acetone-*d*_*6*_): δ = 9.76 (s, 1H), 8.13 (d, *J* = 1.0 Hz, 1H), 7.89 (d, *J* = 8.5 Hz, 2H), 7.41–7.29 (m, 2H), 7.22–7.09 (m, 2H), 6.98 (d, *J* = 3.6 Hz, 1H), 6.88 (t, *J* = 6.8 Hz, 1H), 3.93–3.85 (m, 1H), 2.36 (s, 3H), 0.89–0.67 (m, 4H). ^13^C-NMR (126 MHz, acetone-*d*_*6*_): δ = 159.8, 157.1, 156.5, 145.9, 139.1, 138.7, 126.1, 122.9, 122.8, 122.6, 121.9, 117.8, 116.9, 115.4, 111.0, 106.0, 50.8, 12.4, 5.7. qHNMR (400 MHz, acetone-*d*_*6*_, ethyl-4(dimethylamino)benzoate as reference): purity = 97.5%. MS (APCI + ): *m/z* 407.7 ([M + H]^+^). HRMS (ESI+): *m/z* calculated 408.1109 for C_22_H_19_ClN_3_O_3_^+^, found 408.1103 ([M + H]^+^).

#### 5-Bromo-*N*-(8-chloro-2-(3,4-dichlorophenyl)imidazo[1,2-*a*]pyridin-3-yl)furan-2-carboxamide (**83**)

5-Bromofuran-2-carboxylic acid (**82**, 345 mg, 1.81 mmol, 3.14 eq) was dissolved in methylene chloride (2 mL) under Ar atmosphere. Oxalyl chloride (310 µL, 3.62 mmol, 6.3 eq) was added dropwise to the solution at 0 °C. After 3 h the solvent was removed under reduced pressure and 8-chloro-2-(3,4-dichlorophenyl)imidazo[1,2-*a*]pyridine-3-amine (**24**, 180 mg, 576 mmol, 1.00 eq) dissolved in a mixture of pyridine (1 mL) and toluene (4 mL) was added and the mixture was stirred at rt overnight. 2 N aqueous NaOH solution (10 mL) was added, phases were separated, and the aqueous layer was extracted with ethyl acetate (3x). The combined organic layers were dried over MgSO_4_ and the solvent was removed under reduced pressure. The crude product was purified by flash column chromatography using a gradient of cyclohexane/ ethyl acetate as mobile phase giving **83** as a brown solid (180 mg, 64%). ^1^H-NMR (400 MHz, DMSO-*d*_*6*_): δ = 10.87 (s, 1H), 8.29–8.24 (m, 1H), 8.15 (d, *J* = 2.0 Hz, 1H), 7.92 (dd, *J* = 8.4, 2.1 Hz, 1H), 7.76 (d, *J* = 8.4 Hz, 1H), 7.62–7.57 (m, 1H), 7.50 (d, *J* = 3.6 Hz, 1H), 7.02–6.92 (m, 2H). ^13^C-NMR (101 MHz, DMSO-*d*_*6*_): δ = 157.0, 148.8, 139.8, 136.5, 133.9, 132.0, 131.7, 131.1, 128.6, 127.1, 127.1, 125.7, 124.1, 122.0, 119.2, 117.2, 115.2, 113.1. MS (APCI+): *m/z* 483.3 ([M + H]^+^).

#### 5-Bromo-*N*-(8-chloro-2-methylimidazo[1,2-*a*]pyridin-3-yl)furan-2-carboxamide (**84**)

Preparation according to general procedure B using 8-chloro-2-methylimidazol[1,2-*a*]pyridine-3-amine (**8**, 340 mg, 1.87 mmol, 1.00 eq) and 5-bromofuran-2-carboxylic acid (**82**, 429 mg, 2.24 mmol, 1.20 eq) yielded compound **84** as a colorless solid (536 mg, 81%). ^1^H-NMR (400 MHz, MeOD-*d*_*4*_): δ = 8.00 (d, *J* = 1.0 Hz, 1H), 7.43 (d, *J* = 1.0 Hz, 1H), 7.33 (d, *J* = 3.6 Hz, 1H), 6.91 (t, *J* = 7.1 Hz, 1H), 6.73 (d, *J* = 3.7 Hz, 1H), 2.39 (s, 3H). ^13^C-NMR (101 MHz, MeOD-*d*_*4*_): δ = 157.5, 148.5, 139.5, 138.1, 126.6, 124.2, 122.2, 121.4, 118.5, 116.3, 114.4, 111.9, 11.2. MS (APCI+): *m/z* 355.4 ([M + H]^+^).

Synthetic procedures and analytical data for **8**-**9,** **12,** **14**-**42** are provided as Supplementary Methods in the Supplementary Information (pdf). NMR spectra (^1^H, ^13^C and qH) and HRMS of **8**-**9,** **12,** **14**-**42** are provided in Supplementary Data 1.

### In vitro Characterization

#### Hybrid reporter gene assays

Nurr1 modulation was determined in a Gal4 hybrid reporter gene assay in HEK293T cells (German Collection of Microorganisms and Cell Culture GmbH, DSMZ) using pFR-Luc (Stratagene, La Jolla, CA, USA; reporter), pRL-SV40 (Promega, Madison, WI, USA; internal control) and pFA-CMV-hNurr1-LBD^14^, coding for the hinge region and ligand binding domain of the canonical isoform of human Nurr1. Cells were cultured in Dulbecco’s modified Eagle’s medium (DMEM), high glucose supplemented with 10% fetal calf serum (FCS), sodium pyruvate (1 mM), penicillin (100 U/mL), and streptomycin (100 μg/mL) at 37 °C and 5% CO_2_ and seeded in 96-well plates (3 × 10^4^ cells/well). After 24 h, the medium was changed to Opti-MEM without supplements, and cells were transiently transfected using Lipofectamine LTX reagent (Invitrogen, Carlsbad, CA, USA) according to the manufacturer’s protocol. Five hours after transfection, cells were incubated with the test compounds in Opti-MEM supplemented with penicillin (100 U/mL), streptomycin (100 μg/mL), and 0.1% DMSO for 16 h before luciferase activity was measured using the Dual-Glo Luciferase Assay System (Promega) according to the manufacturer’s protocol on a Tecan Spark luminometer (Tecan Deutschland GmbH, Crailsheim, Germany). Firefly luminescence was divided by Renilla luminescence and multiplied by 1000 resulting in relative light units (RLU) to normalize for transfection efficiency and cell growth. Fold activation was obtained by dividing the mean RLU of the test compound by the mean RLU of the untreated control. All samples were tested in at least three biologically independent experiments in duplicates. For dose-response curve fitting and calculation of EC_50_ values, the equation “[Agonist] vs. response -- Variable slope (four parameters)” was used in GraphPad Prism (version 7.00, GraphPad Software, La Jolla, CA, USA). Selectivity profiling was performed with identical procedures using pFA-CMV-Nur77-LBD^14^, pFA-CMV-NOR-1-LBD^14^, pFA-CMV-THRα-LBD^51^, pFA-CMV-RARα-LBD^52^, pFA-CMV-PPARα-LBD^53^, pFA-CMV-PPARγ-LBD^53^, pFA-CMV-PPARδ-LBD^53^, pFA-CMV-LXRα-LBD^54^, pFA-CMV-FXR-LBD^55^ and pFA-CMV-hRXRα-LBD^56^.

#### Full-length Nurr1 reporter gene assays

Activation of full-length human Nurr1 was studied in transiently transfected HEK293T cells using the reporter plasmids pFR-Luc-NBRE^14^, pFR-Luc-POMC^14^ or pFR-Luc-DR5^14^ each containing one copy of the respective human Nurr1 response element NBRE Nl3, NurRE, or DR5. The full-length human nuclear receptor Nurr1 (pcDNA3.1-hNurr1-NE; Addgene plasmid #102363) and, for DR5, RXRα (pSG5-hRXR)^57^ were overexpressed. pRL-SV40 (Promega) was used for the normalization of transfection efficacy and to observe test compound toxicity. Cells were cultured in Dulbecco’s modified Eagle’s medium (DMEM), high glucose supplemented with 10% fetal calf serum (FCS), sodium pyruvate (1 mM), penicillin (100 U/mL), and streptomycin (100 μg/mL) at 37 °C and 5% CO_2_ and seeded in 96-well plates (3 × 10^4^ cells/well). After 24 h, the medium was changed to Opti-MEM without supplements, and cells were transiently transfected using Lipofectamine LTX reagent (Invitrogen) according to the manufacturer’s protocol. Five hours after transfection, cells were incubated with the test compounds in Opti-MEM supplemented with penicillin (100 U/mL), streptomycin (100 μg/mL) and 0.1% DMSO for 16 h before luciferase activity was measured using the Dual-Glo Luciferase Assay System (Promega) according to the manufacturer’s protocol on a Tecan Spark luminometer (Tecan Deutschland GmbH). Firefly luminescence was divided by Renilla luminescence and multiplied by 1000 resulting in relative light units (RLU) to normalize for transfection efficiency and cell growth. Fold activation was obtained by dividing the mean RLU of the test compound by the mean RLU of the untreated control. All samples were tested in at least three biologically independent experiments in duplicates. For dose-response curve fitting and calculation of EC_50_ values, the equation “[Agonist] vs. response -- Variable slope (four parameters)” was used in GraphPad Prism (version 7.00, GraphPad Software).

#### Isothermal Titration Calorimetry (ITC)

ITC experiments were conducted on an Affinity ITC instrument (TA Instruments, New Castle, DE) at 25 °C with a stirring rate of 75 rpm. Nurr1 LBD protein (5–30 µM, expressed as described previously^21^) in buffer (20 mM Tris pH 7.5, 100 mM NaCl, 5% glycerol) containing 1–4% DMSO was titrated with the test compounds (30–150 μM in the same buffer containing 1-4% DMSO) in 26 injections (1 × 1 µL, 25 × 3-4 μL) with an injection interval of 120-150 s. As control experiments, the test compounds were titrated to the buffer, and the buffer was titrated to the Nurr1 LBD protein under otherwise identical conditions. Results were analyzed using NanoAnalyze software (version 3.11.0, TA Instruments, New Castle, DE) with independent binding models.

#### Evaluation of Nurr1-regulated VMAT2 expression in T98G cells

T98G (ATCC, CRL-1690) were grown in DMEM, high glucose supplemented with 10% FCS, sodium pyruvate (1 mM), penicillin (100 U/mL), and streptomycin (100 μg/mL) at 37 °C and 5% CO_2_ and seeded at a density of 250,000 cells per well in 12-well plates. After 24 h, medium was changed to DMEM, high glucose supplemented with 0.2% fetal calf serum (FCS), penicillin (100 U/mL), and streptomycin (100 µg/mL) and the cells were incubated for another 24 h before stimulation with the test compounds (**36** (0.3 µM), **29** (10 µM)) solubilized with 0.1% DMSO or with 0.1% DMSO as a negative control. After 16 h of incubation, the medium was removed, cells were washed with phosphate-buffered saline (PBS) and after full aspiration of residual liquids, they were immediately frozen at −80 °C until further processing. Total RNA was isolated using the E.Z.N.A.® Total RNA Kit I (Omega Bio-tek, Norcross, USA) following the manufacturer’s instructions. RNA concentration and purity were assessed using a NanoDrop™ One UV/VIS spectrophotometer (Thermo Fisher Scientific, Waltham, USA) at 260/280 nm. Right before reverse transcription (RT), RNA was linearized at 65 °C for 10 min and then immediately incubated on ice for at least 1 min. Reverse transcription was performed using 2 µg total RNA, 20 U Recombinant RNasin® Ribonuclease Inhibitor (Promega, Mannheim, Germany), 100 U SuperScript ® IV Reverse Transcriptase including 5x First Strand Buffer and 0.1 M dithiothreitol (Thermo Fisher Scientific, Waltham, USA), 3.75 ng linear acrylamide, 625 ng random hexamere primers (#11277081001, Merck, Darmstadt, Germany) and 11.25 nmol deoxynucleoside triphosphate mix (2.8 nmol each ATP, TTP, CTP, GTP; #R0186, Thermo Fisher Scientific, Waltham, USA) at a volume of 22.45 µL at 50 °C for 10 min and 80 °C for 10 min using a Thermal cycler XT^96^ (VWR International, Darmstadt, Germany). Quantitative polymerase chain reaction (qPCR) was conducted using an Applied Biosystems™ QuantStudio 1 (Waltham, USA) and a SYBR green-based detection method. Appropriately diluted cDNA was added to 6 pmol of forward and reverse primer, respectively, 0.8 U Taq DNA Polymerase (#M0267, New England Biolabs, Ipswich, USA), 40 ppm SYBR® Green I (#S9430, Sigma Aldrich, St. Louis, USA), 15 nmol deoxynucleoside triphosphate mix (as indicated above), 60 nmol MgCl_2_, 4 µg bovine serum albumin (#B14, Thermo Fisher Scientific, Waltham, USA), 20% BioStab PCR Optimizer II (#53833, Merck, Darmstadt, Germany), and 10% Taq buffer without detergents (#B55, Thermo Fisher Scientific, Waltham, USA) topped up at a final volume of 20 µL with ddH_2_O. Samples underwent 40 cycles of 15 s denaturation at 95 °C, 15 s of primer annealing at 59.4 or 62.4 °C (depending on the primer), and 20 s of elongation at 68 °C. PCR product specificity was evaluated using a melting curve analysis ranging from 65 to 95 °C. VMAT2, TH and SOD2 mRNA expression was normalized to GAPDH mRNA expression per each sample using the ΔCt-method. The following primers for the human genes were used. hVMAT2 (SLC18A2): 5’-GCT ATG CCT TCC TGC TGA TTG C-3’ (fw) and 5’-CCA AGG CGA TTC CCA TGA CGT T-3’ (rev); hTH: 5′-GCT GGA CAA GTG TCA TCA CCT G-3′ (fw) and 5′-CCT GTA CTG GAA GGC GAT CTC A-3′ (rev); hSOD2: 5′-CCA AAG GGG AGT TGC TGG AA -3′ (fw) and 5′-GAA ACC AAG CCA ACC CCA AC -3′ (rev); hGAPDH: 5’-AGG TCG GAG TCA ACG GAT TT-3’ (fw) and 5’-TTC CCG TTC TCA GCC TTG AC-3’ (rev).

#### Determination of aqueous solubility

The aqueous solubility of **29** and **36** was assessed by mixing 1 mg of each test compound with an appropriate volume of water for a theoretical concentration of 4 mM to obtain an oversaturated mixture. The mixture was agitated in a VWR Thermal Shake lite (VWR International GmbH, Darmstadt, Germany) for 24 h at 600 rpm and a constant temperature of 25 °C. The supersaturated mixtures were subsequently centrifuged at 23.300 rpm for 15 min (25 °C). Part of the supernatant was taken off for quantification by UV absorbance at 312 nm with external calibration. The external calibration samples contained 1% DMSO and the test samples were spiked with DMSO to 1% concentration right before the measurement. Absorbance was measured with a Tecan Spark luminometer (Tecan Deutschland GmbH, Crailsheim, Germany). The solubility test was repeated in three independent experiments.

#### Cytotoxicity assays

HEK293T cells were cultured at 37 °C and 5% CO_2_ in DMEM high-glucose supplemented with sodium pyruvate (1 mM), penicillin (100 U/mL), streptomycin (100 µg/mL), and 10% fetal calf serum (FCS) and seeded at a density of 10,000 cells in 96-well plates pre-coated with a 10 µg/mL collagen G solution (Merck KGaA, L7213) at 37 °C for 30 min. N27 rat dopaminergic neural cells (SCC048, Sigma-Aldrich, Darmstadt, Germany) were grown in RPMI 1640 (Gibco, Thermo Fisher Scientific, Waltham) supplemented with 10% FCS, penicillin (100 U/mL), and streptomycin (100 μg/mL) at 37 °C and 5% CO_2_ and seeded at a density of 10,000 cells in 96-well plates. After 24 h, the cells were treated with the solubilized (0.1% DMSO) test compounds in Opti-MEM supplemented with penicillin (100 U/mL) and streptomycin (100 μg/mL) for HEK293T cells or in RPMI 1640 supplemented with penicillin (100 U/mL), streptomycin (100 μg/mL) and 0.2% FCS for N27 cells. Each sample was prepared in four biologically independent repeats. After incubation for 24 h, the medium was refreshed and 10% water-soluble tetrazolinum salt (Cell Counting Kit-8, MedChemExpress) was added to assess metabolic activity. After 4 h, absorbance was measured at 450 nm using a Tecan Spark Cyto instrument (Tecan).

#### Evaluation of microsomal stability

To determine microsomal stability, test compounds (10 μM) were incubated in 100 mM potassium phosphate buffer at pH 7.4 (total volume of 100 µL) containing 0.5 mg/mL male rat liver microsomes (Sprague-Dawley, no. M9066, Merck KGaA, Darmstadt) and 1 mM NADPH for 0, 15, 30, or 60 min. At the end of the incubation time, microsomal activity was terminated by the addition of 500 µL MeCN and subsequent centrifugation at 1700 g for 5 min. A reaction mixture containing heat-inactivated microsomes (95 °C, 10 min) was prepared as a control for each compound. 5 µL supernatant of each sample was analyzed and the remaining concentrations of the respective test compounds at each time point were determined by LC-MS/MS on an API-3200-QTrap (Sciex) with an Agilent Technologies 1100 series setup including a binary pump (G1311A), a degasser (G1322A), and a Shimadzu SIL 20 A HT autosampler under the control of Analyst 1.6 (Sciex). A XBridge BEH C18 (3.5 µm, 150 mm×3 mm, Waters, protected with a 0.5 µm frit) stationary phase was used in combination with a gradient method starting with 0.1% formic acid in water (A) and MeCN (B) as mobile phase (A:B = 80:20) for 6 min going to A: B = 50:50 after 8 min. 5 µL of supernatant diluted in mobile phase starting conditions was loaded onto the column, separated at a flow rate of 400 µL/min, and detected and quantified per Area of MRM (multiple reaction monitoring) with the following transitions: *m/z* 381.797/145.000 (**34**); *m/z* 395.125/158.200 (**36**); *m/z* 409.822/131.000 (**41**); *m/z* 407.838/171.100 (**42**).

#### Parallel artificial membrane permeability assay (PAMPA)

Passive lipid membrane diffusion of test compounds was determined using Merck Millipore MultiScreen Filter Plates (0.45 μm pore diameter, hydrophobic PVDF). The filter inserts were coated with 1% L-α-phosphatidylcholine (Fluka Analytical) in *n*-dodecan. The test compounds were then added to the donor compartment at a final concentration of 500 µM in a phosphate buffer pH 7.4 containing 5% DMSO with a total volume of 150 µL. The acceptor compartment was filled with 300 µL PBS containing 5% DMSO. Additionally, three equilibrium samples were prepared by directly adding the donor solution to the acceptor compartment for the calculation of log Pe values. The filter plates were incubated for 18 h before the test compound concentrations in the acceptor compartments and in the equilibrium, samples were determined by UV absorbance at 320 nm (**34**), 360 nm (**36**), 310 nm (**41**), or 315 nm (**42**) with external calibration in a 96 well quartz plate on a SpectraMax M2e microplate reader (Molecular Devices). logPe values were calculated according to the formula published by Sugano and colleagues^58^.

#### In vitro blood-brain-barrier model

Permeation of test compounds through a human brain endothelial cell barrier was evaluated using the Corning Costar 3470 Transwell Plate system (0.4 µm pore diameter with 6.5 mm inserts) and HBEC-5i cells (ATCC, CRL-3245). Filter inserts were coated one day before seeding with 50 µL 0.01% rat-tail collagen type I (C7661, Sigma-Aldrich) in PBS. 60,000 HBEC-5i cells in 100 µL HBEC-5i-Medium (DMEM/F12 with 10% FCS and 40 µg/mL ECGS) were seeded after aspiration of the coating-supernatant in each insert with 600 µL HBEC-5i-Medium in each receiver well. After 24 h incubation at 37 °C with 5% CO_2_, the medium was exchanged to 600 µL T98G cell supernatant in receiver wells and 100 µL fresh HBEC-5i-Medium in inserts. The medium exchange was repeated every second day. On day 7 after seeding, the medium in the receiver wells was replaced by 600 µL Hank’s balanced salt solution (HBSS) containing 10 mM HEPES and 0.1% DMSO and 100 µL test compound mixtures (antipyrine and test compound; each at 10 µM) in HBSS containing 10 mM HEPES and 0.1% DMSO were added to inserts. After 60 min incubation, 100 µL samples were taken from the receiver wells and diluted in 400 µL (75%/25% A/B; A=Formic acid 0.1% in water; B = Acetonitrile). Test compound concentrations in the samples were determined by LC-MS/MS on an API-3200-QTrap (Sciex) equipped with an Agilent Technologies 1100 series setup including a binary pump (G1311A), a degasser (G1322A), and a Shimadzu SIL 20 A HT autosampler under the control of Analyst 1.6 (Sciex). An XBridge BEH C18 (3.5 µm, 150 mm × 3 mm, Waters, protected with a 0.5 µm frit) served as stationary phase in combination with a gradient method starting with 0.1% formic acid in water (A) and MeCN (B) as mobile phase (80%:20% = A: B) for 6 min going to (50%:50%) after 8 min. 5 µL of aspirated supernatant diluted in mobile phase starting conditions was loaded onto the column, separated at a flow rate of 400 µL/min, and quantified per Area of MRM (multiple reaction monitoring).

#### Organoid PD model

Cell lines: Human induced pluripotent stem cells (hiPSCs; iPSC-LRRK2 isogenic control and iPSC-LRRK2-G2019S) were cultured in Essential 8^TM^ Flex Medium-Kit (Thermo Fisher) on vitronectin (VTN-N; Thermo Fisher) coated cell culture dishes. The cells were passaged every three days as clumps with 0.5 M EDTA (0.5 M EDTA, 5 M NaCl, PBS). The hiPSCs contained a NURR1 GFP reporter cassette and a single point mutation (G2019S) was incorporated into the LRRK2 gene, resulting in the generation of the PD iPSC-LRRK2-G2019S cell line^47^ (cell lines were kindly provided by the Tchieu Lab, Cincinnati). Cells were regularly tested for pluripotency levels using the markers Nanog and Oct-4 and for mycoplasma contamination. Generation of human midbrain-like organoids: Human midbrain-like organoids (hMLOs) were formed according to the protocol of Jo et.al. ^59^. In brief, hiPSCs were dissociated via accutase for 1 h at 37 °C into single cells after reaching 70% confluency. Subsequently, 10.000 cells were seeded in each well of a 96-well v-shaped ultra-low attachment plate (Sbio®) in neuronal induction media (NIC; DMEM/F12 (Thermo Fisher): Neurobasal media (Thermo Fisher) (1:1), 1:100 N2 supplement (Gibco), 1:50 B27 without Vitamin A (Gibco), 1% GlutaMAX (Gibco), 1% minimum essential media-nonessential amino acid (MEM-NEAA) (Gibco), 0.1% ß-mercaptoethanol (Gibco) supplemented with 1 µg/mL heparin (Merck), 10 µM SB431542 (Miltenyi), 200 ng/mL human Noggin (Miltenyi), 0.8 µM CHIR99021 (R&D) and 10 µM Rock inhibitor Y27632 (R&D)). Following a two-day incubation period, the rock inhibitor was removed, and cells were cultured in NIC media until day 3. On day 4, hMLOs were supplemented with 100 ng/mL SHH-C25II (Miltenyi) and 100 ng/mL human FGF8b (Miltenyi). On day 7, the cells were embedded in 30 µL growth factor reduced matrigel (Merck) for 30 min. at 37 °C and cultured for 24 h in tissue growth induction media (Neurobasal media, 1:100 N2 supplement, 1:50 B27 without vitamin A, 1% GlutaMAX, 1% MEM-NEAA, 0.1% ß-mercaptoethanol supplemented with 2.5 µg/mL insulin, 200 ng/mL mouse laminin, 100 ng/mL SHH-C25II and 100 ng/mL human FGF8b. The following day, hMLOs were transferred into ultra-low-attachment 6-well plates (Corning) containing final differentiation media (Neurobasal media, 1:100 N2 supplement, 1:50 B27 without Vitamin A, 1% GlutaMAX, 1% MEM-NEAA, 0.1% ß-mercaptoethanol, 1% Pen/Strep (Gibco), 10 ng/mL BDNF (Miltenyi), 10 ng/mL GDNF (Miltenyi), 100 µM ascorbic acid (Sigma) and 125 µM db-cAMP (Sigma). The hMLOs were cultured on a shaker and medium was changed every three days. hMLOs were treated on day 44 of organoid formation with the respective test compounds for 24 h. At day 45, organoids were harvested for further analysis. RNA isolation, reverse transcription and quantitative real-time PCR (qRT-PCR): Total RNA from hMLOs was isolated using TRIzol (Invitrogen) following the manufacturer´s protocol. RNA extraction was performed using chloroform:isoamyl alcohol (24:1)(Thermo Scientific), precipitated in isopropanol and resuspended in nuclease-free ddH_2_O. After DNAse digestion, 500 µg total RNA was reverse transcribed using the RevertAid First-strand-cDNA-synthesis kit (Thermo Fisher) and random hexamers following the manufacturer´s protocol. qRT-PCR was conducted with specific primers targeting hTH (5′-GCT GGA CAA GTG TCA TCA CCT G-3′ (fw) and 5′-CCT GTA CTG GAA GGC GAT CTC A-3′ (rev)), Nurr1 (5’-GGC TGA AGC CAT GCC TTG T-3’ (fw) and 5’-GTG AGG TCC ATG CTA AAC TTG ACA-3’ (rev)) and RNA polymerase 2 (5’-GCA CCA CGT CCA ATG ACA-3’ (fw) and 5’-GTC GGC TGC TTC CAT AA-3’ (rev)) using the LightCycler ® 480 SYBR Green I (Roche) on a Roche LightCycler 480 II qPCR system. Relative gene expression was determined using the -ΔΔCt method. All genes were normalized to RNA polymerase II values. Immunostaining of hMLOs: hMLOs were washed twice with PBS and fixed in 4% paraformaldehyde (PFA) overnight at 4 °C. On the following day, hMLOs were washed twice with PBS, cryoprotected and stored in 30% sucrose/PBS. Organoids were sectioned at 25 µm on a cryostat (Leica CM 3050 S, Leica Biosystems, Wetzlar, Germany). Immunostaining was performed within 1.5 mL Eppendorf tubes. Sections were permeabilized in 0.3% Triton X-100, blocked in 5% FBS, 0.1% Triton X-100/PBS and incubated as floating section in 1:200 dilution of primary antibody (rabbit anti-Tyrosine-Hydroxylase, Merck (AB152)) in blocking solution overnight. The next day, sections were washed twice with 0.1% Triton X-100, incubated in 1:500 dilution of secondary antibody (AlexaFluor^TM^ 594 goat anti-rabbit IgG (H + L), Invitrogen) in blocking solution for 1.5 h and Hoechst 33342 (ChemCruz) was added in 1:10 000 dilution for 30 min for nuclear staining. Sections were mounted onto glass slides and imaged using a Zeiss LSM 980 microscope (Carl Zeiss AG, Oberkochen, Germany). The staining protocol was adapted from^60,61^. Images were manually quantified by selecting a fixed area and counting TH+ neurons. The images used for quantification were acquired from four different sections of two different organoids for each condition. Statistical analysis: All experiments were performed at least in triplicate, if not otherwise indicated. Results are expressed as mean ± SD. Statistical significance was analyzed using two-tailed Student’s t-test (*p < 0.05, **p < 0.01 and ***p < 0.001).

### Reporting summary

Further information on research design is available in the Nature Portfolio Reporting Summary linked to this article.

### Supplementary information

Supplementary Information

Description of Additional Supplementary Files

Supplementary Data 1

Supplementary Data 2

Reporting Summary

## Data Availability

All data supporting the results of this study are available from the corresponding author. Source data for Figs. 1, 4, and 5 are provided in Supplementary Data 2.
