# Supplementary material for: Development of Nurr1 agonists from amodiaquine by scaffold hopping and fragment growing
Source: Commun Chem. 2024 Jun 29;7:149. doi: 10.1038/s42004-024-01224-0 (PMC11217349; doi:10.1038/s42004-024-01224-0)
Supplement: Supplementary file 1 — Supplementary Information [file 42004_2024_1224_MOESM1_ESM.pdf]

- Supplementary Information -

Development of Nurr1 Agonists from Amodiaquine By Scaffold Hopping and Fragment Growing

Minh Sai[1], Emily C. Hank[1], Hin-Man Tai[2], Till Kasch[1], Max Lewandowski[1], Michelle Vincendeau[2,3], Julian A. Marschner[1], Daniel Merk[1]*

[1] Ludwig-Maximilians-Universität München, Department of Pharmacy, 81377 Munich, Germany
[2] Helmholtz Munich, Institute of Virology, 85764 Munich, Germany
[3] Technical University of Munich, Institute of Virology, School of Medicine, 81675 Munich, Germany
* daniel.merk@cup.lmu.de

Table of Contents

Supplementary Figures 2
Supplementary Methods 5
Supplementary References 18

Supplementary Figures

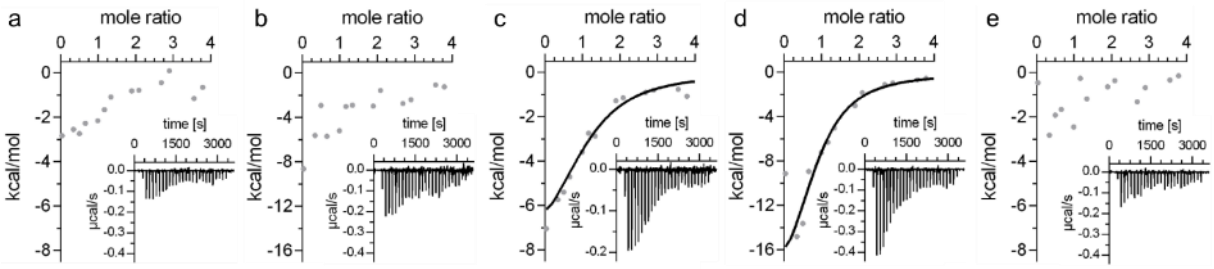

Supplementary Figure 1. Isothermal titration calorimetry (ITC) to determine binding of **9** (a), **10** (b), **11** (c), **12** (d) and **13** (e) to the Nurr1 LBD using 100 µM ligand and 15 µM protein. The fittings of the heat of binding are shown and the isotherms at 25°C are shown as insets.

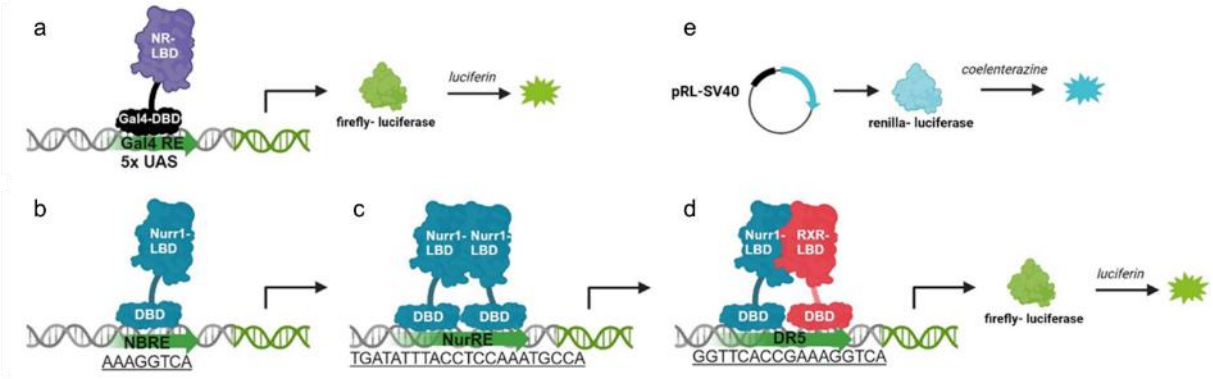

Supplementary Figure 2. Schematic illustration of reporter gene assays used in this study. All reporter gene assays were performed in transiently transfected HEK293T cells and firefly/renilla luminescence was measured using the Dual Glo kit from Promega. (a) Gal4-hybrid reporter gene assays used to characterize Nurr1 agonism during structural optimization and for selectivity profiling are based on chimeric receptors composed of the Gal4-DBD and the hinge region and LBD ("NR LBD") of the respective human nuclear receptor. A firefly luciferase construct with five repeats of the tandem Gal4 binding site UAS was used as reporter gene. (b-d) Selected compounds were studied in reporter gene assays testing the activity of the human full-length nuclear receptor Nurr1 which can act as monomer on the response element NBRE (b), as homodimer on NurRE (c), and as heterodimer with RXR on DR5 (d). For the reporter gene assays, Nurr1 and RXR (only for DR5) were overexpressed and firefly luciferase constructs each comprising a single repeat of either NBRE, NurRE or DR5 (sequences in figure) to control reporter gene expression were used as reporters. (e) A renilla luciferase construct with constitutively active SV40 promoter was co-transfected in all reporter gene assay settings as internal control for normalization of transfection efficiency and to monitor potential test compound toxicity. Created with BioRender.com.

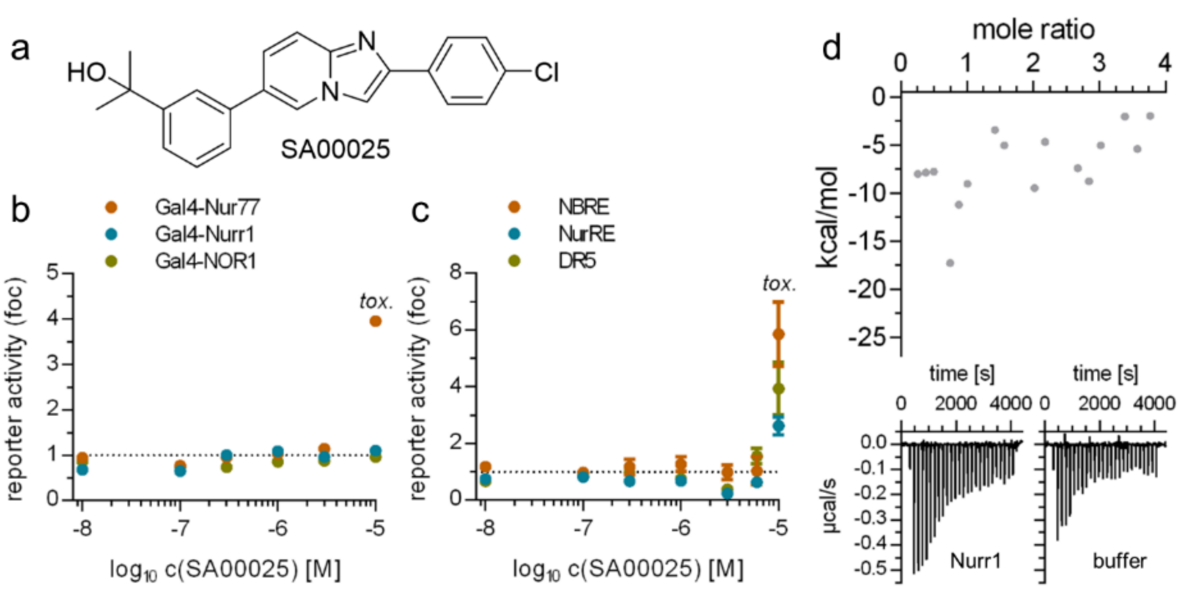

Supplementary Figure 3. In vitro characterization of the imidazo[1,2-*a*]pyridin-based Nurr1 modulator SA00025 (CAS# 1015231-98-7)[1]. (a) Chemical structure of SA00025. (b) SA00025 caused no activation of NR4A receptors in Gal4 hybrid reporter gene assays in the concentration range from 10 nM to 3 µM. Higher concentrations were cytotoxic (tox.) leading to a drop in the control gene (renilla luciferase) luminescence and consequently increased firefly/renilla luminescence ratio that should not be misinterpreted as nuclear receptor activation. Data are the mean±S.E.M., n≥3. (c) SA00025 caused no activation of full-length Nurr1 on the human Nurr1 response elements NBRE, NurRE and DR5 in the concentration range from 10 nM to 3 µM. Higher concentrations were cytotoxic (tox.) leading to a drop in the control gene (renilla luciferase) luminescence and consequently increased firefly/renilla luminescence ratio that should not be misinterpreted as nuclear receptor activation. Data are the mean±S.E.M., n≥3. (d) ITC (50 µM SA00025, 10 µM Nurr1 LBD) indicated potential weak binding of SA00025 to the recombinant Nurr1 LBD but affinity was too low to determine a K_d value.

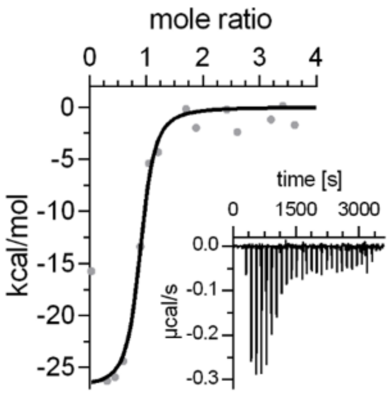

Supplementary Figure 4. The fused compound **37** bound to the recombinant Nurr1 LBD with high affinity (K_d 0.08 µM). The fitting of the heat of binding is shown and the isotherm at 25°C is shown as inset. 30 µM **37** and 5 µM Nurr1 LBD were used.

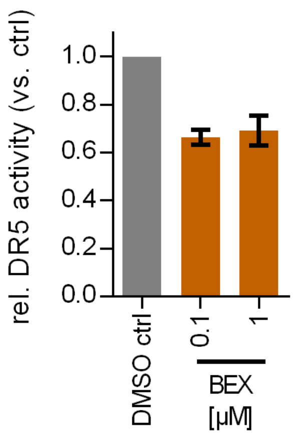

Supplementary Figure 5. Effect of bexarotene (BEX) on DR5 activity. Data are the mean±S.E.M.; n=3.

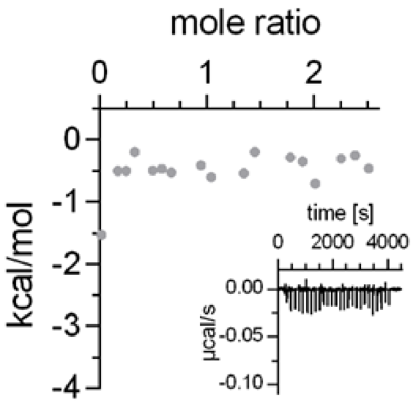

Supplementary Figure 6. **29** showed no binding to the Nurr1 LBD in ITC using 100 µM ligand and 30 µM protein. The fitting of the heat of binding is shown and the isotherm at 25°C is shown as inset.

Supplementary Methods

Synthetic Procedures and Analytical Data

General. All chemicals were of reagent grade, purchased from commercial sources (e.g., Sigma-Aldrich, TCI, BLDpharm) and used without further purification unless otherwise specified. All reactions were conducted under nitrogen or argon atmosphere and in absolute solvents purchased from Sigma-Aldrich. Other solvents, especially for work-up procedures, were of reagent grade or purified by distillation (*iso*-hexane, cyclohexane, ethyl acetate, EtOH). Reactions were monitored by thin layer chromatography (TLC) on TLC Silica gel 60 F254 coated aluminum sheets by Merck and visualized under ultraviolet light (254 nm) or by using ninhydrin or Ehrlichs reagent stains. Purification by column chromatography was performed on a puriFlash® XS520Plus system (Advion, Ithaca, NY, USA) using high performance spherical silica columns (SIHP, 50 µm) by Interchim and a gradient of *iso*-hexane or cyclohexane to ethyl acetate, Reversed-phase CC was performed on a puriFlash® 5.250 system (Advion) using C18HP columns (SIHP, 15 µm) by Interchim and a gradient of H_2O with 10% MeCN to 100% MeCN (HPLC gradient grade). Mass spectra were obtained on a puriFlash®-CMS system (Advion) using atmospheric pressure chemical ionization (APCI). HRMS were obtained with a Thermo Finnigan LTQ FT instrument for electron impact ionization (EI) or electrospray ionization (ESI). NMR spectra were recorded on Bruker Avance III HD 400 MHz or 500 MHz spectrometers equipped with a CryoProbeTM Prodigy broadband probe (Bruker). Chemical shifts are reported in δ values (ppm) relative to residual protium signals in the NMR solvent (^{1}H-NMR: acetone-d_6: δ = 2.04 ppm; DMSO-d_6: δ = 2.50 ppm; MeOD-d_4: δ = 3.31 ppm, ^{13}C-NMR: acetone-d_6: δ = 206.26, 29.84 ppm; DMSO-d_6: δ = 39.52 ppm; MeOD-d_4:δ = 49.0 ppm), coupling constants (*J*) in hertz (Hz). The purity of the compounds was determined by ^{1}H NMR (qHNMR) according to the method described by Pauli et al.[2] with internal calibration. To ensure accurate determination of peak area ratio, the qHNMR measurements were conducted under conditions allowing for complete relaxation. Ethyl 4-(diemethylamino)benzoate (LOT#BCCC6657, purity 99.63%), dimethyl terephthalate (LOT#BCBT9974, purity 99.95%) and maleic acid (LOT#BCBM8127V, purity 99.94%) were used as internal standards in MeOD-d_4, DMSO-d_6, or acetone-d_6. All compounds for biological testing had a purity >95% according to quantitative NMR.

General procedure A for Groebke-Blackburn-Bienaymé reaction and hydrolysis.
2-Amino-3-chloropyridine (**43**, 1.0 eq.) or 2-amino-4-chloropyridine (**44**, 1.0 eq.), the respective aldehyde (**41**-**53**, 1.0-1.1 eq.) and glacial acetic acid (1.5 eq.) were dissolved in dry methanol (0.7 M) under nitrogen. The mixture was stirred for 30–45 min at room temperature (rt) for imine formation. 1,1,3,3-Tetramethylbutylisocyanid (**63**, 1.5 eq.) was subsequently added, and the mixture was stirred at rt for 17-48 h. When TLC monitoring indicated completion, the isocyanide was quenched by addition of 2 N aqueous HCl (2 mL) and further stirring for 30 min. 2 N aqueous NaOH solution and ethyl acetate (10 mL) were then added and the phases were separated. The aqueous layer was extracted with ethyl acetate (3x). The combined organic layers were dried over $MgSO_4$ and the solvent was evaporated under reduced pressure. The crude product was dissolved in a mixture of methylene chloride and trifluoroacetic acid (10 mL, 1:1) or 4 N HCl in dioxane (10 mL) and the mixture was stirred for 30-60 min at rt. When TLC monitoring indicated completion, 2 N aqueous NaOH solution was

added, phases were separated, and the aqueous layer was extracted with ethyl acetate (3x). The combined organic layers were dried over $MgSO_4$ and the solvents were evaporated under reduced pressure. The crude product was purified by flash column chromatography using a gradient of *iso*-hexane or cyclohexane / ethyl acetate as mobile phase, and potentially by reverse phase chromatography using a gradient of H_2O with 10% MeCN to 100% MeCN (HPLC grade).

General procedure B for amide coupling with HATU.

The respective carboxylic acid (**72-80**, 1.2 eq.) and 1-[bis(dimethylamino)methylene]-1*H*-1,2,3-triazolo[4,5-b]pyridinium 3-oxide hexafluorophosphate (HATU, 1.2 eq.) were dissolved in DMF (0.13 M). *N*-Ethyldiisopropylamine (DIPEA, 0.13 M 1.2 eq) was added and the mixture was stirred at rt for 40 min. 3-Aminoimidazo[1,2-*a*]pyridine (**8**, 1.0 eq.) was dissolved in DMF (0.11 M) and added to the activated carboxylic acid. The mixture was stirred at rt overnight. When TLC monitoring indicated completion, the solvent was removed under reduced pressure, the residue was dissolved in ethyl acetate and treated with 5% HCl (0.13M). Phases were separated and the aqueous layer was extracted with ethyl acetate (3x). The combined organic layers were washed with 1 N aqueous NaOH solution and dried over $MgSO_4$. The solvent was evaporated under reduced pressure and the crude product was purified by flash column chromatography using a gradient of *iso*-hexane or cyclohexane / ethyl acetate as mobile phase, and potentially by reverse phase chromatography using a gradient of H_2O with 10% MeCN to 100% MeCN (HPLC gradient grade).

Synthesis and analytical characterization of **8-9, 12, 14-42** and precursors

8-Chloro-2-methylimidazo[1,2-*a*]pyridine-3-amine (8). Preparation according to general procedure A using 2-amino-3-chlorpyridine (**43**, 821 mg, 6.39 mmol, 1.00 eq) and acetaldehyde (**45**, 39.0 µL, 7.03 mmol, 1.10 eq) yielded compound **8** as a colorless solid (344 mg, 30%). ^{1}H-NMR (400 MHz, acetone-*d₆*): δ = 8.05 (dd, *J* = 6.8, 1.1 Hz, 1H), 7.11 (dd, *J* = 7.3, 1.0 Hz, 1H), 6.76 (t, *J* = 7.0 Hz, 1H), 4.17 (s, 2H), 2.33 (s, 3H).^{13}C-NMR (101 MHz, acetone-d_6): δ = 136.1, 130.1, 126.3, 121.5, 121.0, 119.8, 110.0, 11.9. qHNMR (400 MHz, acetone-d_6, ethyl-4(dimethylamino)benzoate as reference): purity = 97.5%. MS (APCI+): *m/z* 181.9 ($[M+H]^+$). HRMS (EI+): *m/z* calculated 181.0407 for $C_8H_8ClN_3$, found 181.0400 ($[M]^{\bullet+}$).

7-Chloro-2-methylimidazo[1,2-*a*]pyridine-3-amine (9). Preparation according to general procedure A using 2-amino-4-chlorpyridine (**44**, 87.0 mg, 0.68 mmol, 1.00 eq) and acetaldehyde (**46**, 0.04 µL, 0.74 mmol, 1.10 eq) yielded compound **9** as a colorless solid (50 mg, 41%). ^{1}H-NMR (400 MHz, acetone-d_6): δ = 8.05 (dd, *J* = 7.2, 0.8 Hz, 1H), 7.32 (dd, *J* = 2.1, 0.8 Hz, 1H), 6.78 (dd, *J* = 7.3, 2.1 Hz, 1H), 4.08 (s, 2H), 2.29 (s, 3H). ^{13}C-NMR (101 MHz, acetone-d_6): δ = 138.9, 130.7, 126.4, 125.0, 122.7, 114.8, 111.4, 11.9. qHNMR (400 MHz, MeOH-d_6, ethyl-4-(dimethylamino)benzoate as reference): purity = 97.8%. MS (APCI+): *m/z* 181.9 ($[M+H]^+$). HRMS (EI+): *m/z* calculated 181.0407 for $C_8H_8ClN_3$, found 181.0400 ($[M]^{\bullet+}$).

8-Chloroimidazo[1,2-*a*]pyridine-3-amine (12). 8-Chloro-3-nitroimidazo[1,2-*a*]pyridine (**65**, 156 mg, 0.08 mmol, 1.00 eq) was dissolved in H_2O/MeOH (10 mL, 1:9). Iron powder (441 mg, 7.90 mmol, 10.0 eq) and ammonium chloride (296 mg, 5.53 mmol, 7.00 eq) were added to the solution and the resulting mixture was refluxed overnight. The mixture was filtered through Celite, diluted with ethyl acetate and neutralized with saturated $NaHCO_3$ solution. Phases were separated, the aqueous layer was extracted with ethyl acetate (10 mL), the combined organic layers were dried over $MgSO_4$, and the solvents were removed under reduced pressure. The crude was purified by flash column chromatography and reverse phase chromatography to yield compound **12** as a yellow solid (75 mg, 57%). ^{1}H-NMR (400 MHz, acetone-d_6): δ = 8.08 (dd, *J* = 6.9, 1.0 Hz, 1H), 7.15 (dd, *J* = 7.2, 1.0 Hz, 1H), 7.03 (s, 1H), 6.80 (t, *J* = 7.0 Hz, 1H), 4.57 (s, 2H). ^{13}C-NMR (101 MHz, MeOD-d_4): δ = 138.8, 133.0, 123.2, 122.8, 122.4, 119.1, 112.43. qHNMR (400 MHz, MeOH-d_4, ethyl-4-(dimethylamino)benzoate as reference): purity = 97.9%. MS (APCI+): *m/z* 168.0 ($[M+H]^+$). HRMS (EI+): *m/z* calculated 167.0250 for $C_7H_6ClN_3$, found 167.0244 ($[M]^{\bullet+}$).

8-Chloro-2-isopropylimidazo[1,2-*a*]pyridine-3-amine (14). Preparation according to general procedure A using 2-amino-4-chlorpyridine (**43**, 395 mg, 3.07 mmol, 1.00 eq) and 2-methylpropanal (**46**, 336 µL, 3.68 mmol, 1.20 eq) yielded compound **14** as a colorless solid (131 mg, 22%). ^{1}H-NMR (400 MHz, acetone-d_6): δ = 8.07 (dd, *J* = 6.8, 1.1 Hz, 1H), 7.12 (dd, *J* = 7.2, 1.1 Hz, 1H), 6.76 (t, *J* = 7.0 Hz, 1H), 4.15 (s, 2H), 3.30–3.21 (m, 1H), 1.30 (d, *J* = 6.9 Hz, 6H). ^{13}C-NMR (101 MHz, MeOD-d_4): δ = 140.1, 138.1, 126.2, 122.7, 122.5, 122.3, 112.0, 27.4, 22.4. qHNMR (400 MHz, acetone-d_6, ethyl-4-(dimethylamino)benzoate as reference): purity = 95.0%. MS (APCI+): *m/z* 209.9 ($[M+H]^+$). HRMS (EI+): *m/z* calculated 209.0720 for $C_{10}H_{12}ClN_3$, found 209.0713 ($[M]^{\bullet+}$).

8-Chloro-2-phenylimidazo[1,2-*a*]pyridine-3-amine (15). Preparation according to general procedure A using 2-amino-4-chlorpyridine (**43**, 390 mg, 3.03 mmol, 1.0 eq) and benzaldehyde (**47**, 322 mg, 3.03 mmol, 1.00 eq) yielded compound **15** as a brown solid (270 mg, 35%). ^{1}H-NMR (400 MHz, acetone-d_6): δ = 8.26–8.19 (m, 1H), 8.19–8.12 (m, 2H), 7.48–7.38 (m, 2H), 7.31–7.18 (m, 2H), 6.84 (t, *J* = 7.1 Hz, 1H), 4.68 (s, 2H). ^{13}C-NMR (101 MHz, acetone-d_6): δ = 136.7, 135.1, 130.8, 128.3, 126.8, 126.2, 126.4, 122.2, 121.4, 121.2, 110.6. qHNMR (400 MHz, acetone-d_6, ethyl-4-(dimethylamino)benzoate as reference) purity = 97.1%. MS (APCI+): *m/z* 243.8 ([M+H]$^+$). HRMS (EI+): *m/z* calculated 243.0563 for $C_{13}H_{10}ClN_3$, found 243.0557 ([M]$\bullet^+$).

8-Chloro-2-(furan-2-yl)imidazo[1,2-*a*]pyridin-3-amine (16). Preparation according to general procedure A using 2-amino-4-chlorpyridine (**43**, 300 mg, 2.33 mmol, 1.00 eq) and furan-3-carbaldehyde (**48**, 246 mg, 2.56 mmol, 1.10 eq) yielded compound **16** as a colorless solid (270 mg, 48%). ^{1}H-NMR (400 MHz, acetone-d_6): δ = 8.18 (dd, *J* = 6.9, 1.0 Hz, 1H), 8.15–8.13 (m, 1H), 7.63 (t, *J* = 1.7 Hz, 1H), 7.19 (dd, *J* = 7.2, 1.0 Hz, 1H), 7.13–7.06 (m, 1H), 6.82 (t, *J* = 7.0 Hz, 1H), 4.48 (s, 2H). ^{13}C-NMR (101 MHz, acetone-d_6): δ = 143.1, 139.5, 136.9, 126.1, 125.8, 121.9, 121.3, 120.9, 120.7, 110.6, 109.3. qHNMR (400 MHz, acetone-d_6, ethyl-4-(dimethylamino)benzoate as reference): purity = 98.8%. MS (APCI+): *m/z* 233.6 ([M+H]$^+$). HRMS (EI+): *m/z* calculated 233.0356 for $C_{11}H_8ClN_3O$, found 233.0350 ([M]$\bullet^+$).

8-Chloro-2-(4-chlorophenyl)imidazo[1,2-*a*]pyridine-3-amine (17). Preparation according to general procedure A using 2-amino-4-chlorpyridine (**43**, 123 mg, 960 µmol, 1.00 eq) and 4-chlorobenzaldehyde (**49**, 135 mg, 960 µmol, 1.00 eq) yielded compound **17** as a yellow solid (14 mg, 5%). ^{1}H-NMR (400 MHz, MeOH-d_4): δ = 8.16 (dd, *J* = 6.9, 1.0 Hz, 1H), 7.96–7.91 (m, 2H), 7.48–7.41 (m, 2H), 7.28 (dd, *J* = 7.3, 1.0 Hz, 1H), 6.86 (t, *J* = 7.0 Hz, 1H). ^{13}C-NMR (101 MHz, MeOH-d_4): δ = 137.1, 132.6, 132.4, 128.9, 128.5, 128.2, 127.5, 122.2, 121.6, 121.2, 111.2. qHNMR (400 MHz, acetone-d_6, ethyl-4-(dimethylamino)benzoate as reference): purity = 95.7%. MS (APCI+): *m/z* 277.9 ([M+H]$^+$). HRMS (EI+): *m/z* calculated 277.0174 for $C_{13}H_9Cl_2N_3$, found 277.0167 ([M]$\bullet^+$).

8-Chloro-2-(3-chlorophenyl)imidazo[1,2-*a*]pyridine-3-amine (18). Preparation according to general procedure A using 2-amino-4-chlorpyridine (**43,** 302 mg, 2.35 mmol, 1.00 eq) and 3-chlorobenzaldehyde (**50**, 328mg, 2.33 mmol, 1.00 eq) yielded compound **18** as a yellow solid (25 mg, 4%). ^{1}H-NMR (400 MHz, acetone-d_6): δ = 8.29–8.20 (m, 2H), 8.17–8.08 (m, 1H), 7.44 (t, *J* = 7.9 Hz, 1H), 7.32–7.21 (m, 2H), 6.86 (t, *J* = 7.0 Hz, 1H), 4.79 (s, 2H). ^{13}C-NMR (101 MHz, MeOD-d_4): δ = 138.6, 137.4, 135.5, 131.0, 129.8, 129.2, 128.2, 127.9, 126.5, 123.8, 123.1, 122.7, 112.6. qHNMR (400 MHz, acetone-d_6, ethyl-4-(dimethylamino)benzoate as reference) purity = 97.2%. MS (APCI+): *m/z* 278.0 ([M+H]$^+$). HRMS (EI+): *m/z* calculated 277.0174 for $C_{13}H_9Cl_2N_3$, found 277.0168 ([M]$\bullet^+$).

8-Chloro-2-(2-chlorophenyl)imidazo[1,2-*a*]pyridine-3-amine (19). Preparation according to general procedure A using 2-amino-4-chlorpyridine (**43**, 300 mg, 2.33 mmol, 1.00 eq) and 2-chlorobenzaldehyde (**51**, 328 mg, 2.33 mmol, 1.00 eq) yielded compound **19** as a white solid (209 mg, 23%). ^{1}H-NMR (400 MHz, acetone-d_6): δ = 8.19 (dd, *J* = 6.9, 1.1 Hz, 1H), 7.70 –7.63 (m, 1H), 7.55–7.48 (m, 1H), 7.47–7.36 (m, 2H), 7.24 (dd, *J* = 7.3, 1.0 Hz, 1H), 6.87 (t, *J* = 7.1 Hz, 1H), 4.53 (s, 2H). ^{13}C-NMR (101 MHz, MeOD-d_4): δ = 142.0, 136.9, 133.6, 132.6, 129.4, 128.4, 128.3, 127.8, 126.6, 122.1, 121.7, 121.3, 111.2. qHNMR (400 MHz, acetone-d_6, ethyl-

4-(dimethylamino)benzoate as reference): purity = 95.6%. MS (APCI+): *m/z* 277.8 ($[M+H]^+$). HRMS (EI+): *m/z* calculated 277.0174 for $C_{13}H_9Cl_2N_3$, found 277.0167 ($[M]^{•+}$).

8-Chloro-2-(*p*-tolyl)imidazo[1,2-*a*]pyridine-3-amine (20). Preparation according to general procedure A using 2-amino-4-chlorpyridine (**43**, 309 mg, 2.40 mmol, 1.0 eq) and 4-methylbenzaldehyde (**52**, 289 mg, 2.40 mmol, 1.0 eq) yielded compound **20** as a yellow solid (178 mg, 29%). ^{1}H-NMR (400 MHz, acetone-*d*$_6$): δ = 8.20 (dd, *J* = 6.9, 1.0 Hz, 1H), 8.07–7.99 (m, 2H), 7.28–7.17 (m, 3H), 6.83 (t, *J* = 7.1 Hz, 1H), 4.60 (s, 2H), 2.36 (s, 3H).^{13}C-NMR (101 MHz, acetone-*d*$_6$): δ = 136.6, 136.1, 132.2, 131.2, 128.9, 126.8, 126.3, 122.1, 121.4, 121.0, 110.5, 20.3. qHNMR (400 MHz, acetone-*d*$_6$, ethyl-4-(dimethylamino)benzoate as reference): purity = 96.5%. MS (APCI+): *m/z* 258.0 ($[M+H]^+$). HRMS (EI+): *m/z* calculated 257.0720 for $C_{14}H_{12}ClN_3$, und 257.0714 ($[M]^{•+}$).

8-Chloro-2-(*m*-tolyl)imidazo[1,2-*a*]pyridine-3-amine (21). Preparation according to general procedure A using 2-amino-4-chlorpyridine (**43**, 395 mg, 3.07 mmol, 1.00 eq) and 3-methylbenzaldehyde (**53**, 369 mg, 3.07 mmol, 1.00 eq) yielded compound **21** as a yellow solid (239 mg, 34%). ^{1}H-NMR (400 MHz, acetone-*d*$_6$): δ = 8.24–8.16 (m, 1H), 7.98 (s, 1H), 7.93 (d, *J* = 7.9 Hz, 1H), 7.31 (td, *J* = 7.7, 1.4 Hz, 1H), 7.21 (d, *J* = 7.2 Hz, 1H), 7.13–7.05 (m, 1H), 6.87–6.79 (m, 1H), 4.68 (s, 2H), 2.39 (s, 3H). ^{13}C-NMR (101 MHz, acetone-*d*$_6$): δ = 137.7, 136.6, 135.0, 130.8, 128.2, 127.4, 127.3, 126.8, 123.9, 122.2, 121.4, 121.0, 110.6, 20.7. qHNMR (400 MHz, acetone-*d*$_6$, dimethyl terephthalate as reference): purity = 95.3%. MS (APCI+): *m/z* 257.9 ($[M+H]^+$). HRMS (EI+): *m/z* calculated 257.0720 for $C_{14}H_{12}ClN_3$, found 257.0714 ($[M]^{•+}$).

8-Chloro-2-(4-(trifluoromethyl)yphenyl)imidazo[1,2-*a*]pyridine-3-amine (22). Preparation according to general procedure A using 2-amino-4-chlorpyridine (**43**, 395 mg, 3.07 mmol, 1.00 eq) and 4-(trifluoromethyl)benzaldehyde (**54**, 535 mg, 3.07 mmol, 1.00 eq) yielded compound **22** as a yellow solid (33 mg, 3%). ^{1}H-NMR (400 MHz, acetone-*d*$_6$): δ = 8.42–8.35 (m, 2H), 8.29–8.23 (m, 1H), 7.80–7.72 (m, 2H), 7.27 (dd, *J* = 7.3, 1.0 Hz, 1H), 6.88 (t, *J* = 7.1 Hz, 1H), 4.90 (s, 2H).^{13}C-NMR (101 MHz, MeOH-*d*$_4$): δ = 137.9, 137.3, 128.5, 128.1 (q, *J* = 32.4 Hz) , 127.8, 127.1, 124.5 (q, *J* = 270.8 Hz), 125.0 (q, *J* = 3.8 Hz), 122.5, 121.8, 121.3, 111.3. qHNMR (400 MHz, acetone-*d*$_6$, dimethyl terephthalate as reference): purity = 98.2%. MS (APCI+): *m/z* 311.5 ($[M+H]^+$). HRMS (EI+): *m/z* calculated 311.0437 for $C_{14}H_9ClF_3N_3$, found 311.0431 ($[M]^{•+}$).

8-Chloro-2-(3-(trifluoromethyl)yphenyl)imidazo[1,2-*a*]pyridine-3-amine (23). Preparation according to general procedure A using 2-amino-4-chlorpyridine (**43**, 405 mg, 3.15 mmol, 1.00 eq) and 3-(trifluoromethyl)benzaldehyde (**55**, 548 mg, 3.15 mmol, 1.00 eq) yielded compound **23** as a yellow solid (27 mg, 3%). ^{1}H-NMR (400 MHz, acetone-*d*$_6$): δ = 8.60–8.52 (m, 1H), 8.48 (d, *J* = 8.2 Hz, 1H), 8.32–8.23 (m, 1H), 7.70–7.62 (m, 1H), 7.62–7.56 (m, 1H), 7.31–7.23 (m, 1H), 6.93–6.83 (m, 1H), 4.82 (s, 2H).^{13}C-NMR (126 MHz, MeOD-*d*$_4$): δ = 137.4, 135.0, 130.5 (q, *J* = 31.78 Hz), 130.2, 128.9, 128.7, 127.9, 124.5 (q, *J* = 270.93) 123.6(q, *J* = 4.0 Hz), 123.0 (q, *J* = 3.9 Hz), 122.6, 121.8, 121.4, 111.3. qHNMR (400 MHz, acetone-*d*$_6$, dimethyl terephthalate as reference): purity = 95.3%. MS (APCI+): *m/z* 311.5 ($[M+H]^+$). HRMS (EI+): *m/z* calculated 311.0437 for $C_{14}H_9ClF_3N_3$, found 311.0429 ($[M]^{•+}$).

8-Chloro-2-(3,4-dichlorophenyl)imidazo[1,2-*a*]pyridine-3-amine (24). Preparation according to general procedure A using 2-amino-4-chlorpyridine (**43**, 1.30 g, 10.0 mmol, 1.00 eq) and 3,4-dichlorobenzaldehyde (**56**, 1.93 g, 11.0 mmol, 1.10 eq) yielded compound **24** as a yellow solid (197 mg, 6%). ^{1}H-NMR (400 MHz, acetone-*d₆*): δ = 8.41–8.35 (m, 1H), 8.30–8.23 (m, 1H), 8.20–8.12 (m, 1H), 7.63–7.56 (m, 1H), 7.27 (dd, *J* = 7.3, 1.1 Hz, 1H), 6.88 (t, *J* = 7.1 Hz, 1H), 4.83 (s, 2H).^{13}C-NMR (101 MHz, MeOD-d_4): δ = 137.2, 134.4, 132.1, 130.1, 130.0, 128.6, 128.0, 127.5, 126.3, 122.6, 121.7, 121.3, 111.3. qHNMR (400 MHz, acetone-d_6, ethyl-4-(dimethylamino)benzoate as reference): purity = 95.7%. MS (APCI+): *m/z* 311.9 ($[M+H]^+$). HRMS (EI+): *m/z* calculated 310.9784 for $C_{13}H_8Cl_3N_3$, found 310.9778 ($[M]^{\bullet +}$).

8-Chloro-2-(3,4-dimethylyphenyl)imidazo[1,2-*a*]pyridine-3-amine (25). Preparation according to general procedure A using 2-amino-4-chlorpyridine (**43**, 395 mg, 3.07 mmol, 1.00 eq) and 3,4-dimethylbenzaldehyde (**57**, 412 mg, 3.07 mmol, 1.00 eq) yielded compound **25** as a yellow solid (307 mg, 40%). ^{1}H-NMR (400 MHz, DMSO-d_6): δ = 8.27–8.20 (m, 1H), 7.81 (s, 1H), 7.79–7.72 (m, 1H), 7.20 (t, *J* = 7.7 Hz, 2H), 6.82 (t, *J* = 7.1 Hz, 1H), 5.30 (s, 2H), 2.30 (s, 3H), 2.25 (s, 3H). ^{13}C-NMR (101 MHz, DMSO-d_6): δ = 136.5, 135.7, 134.7, 132.6, 130.0, 128.3, 128.2, 127.8, 124.3, 122.1, 121.4, 121.1, 111.0, 20.0, 19.6. qHNMR (400 MHz, acetone-d_6, dimethyl terephthalate as reference): purity = 99.8%. MS (APCI+): *m/z* 271.9 ($[M+H]^+$). HRMS (EI+)**:** *m/z* calculated 271.0876 for $C_{15}H_{14}ClN_3$, found 271.0870 ($[M]^{\bullet +}$).

***N*-(8-Chloro-2-methylimidazo[1,2-*a*]pyridin-3-yl)-5-(4-chlorophenyl)furan-2-carboxamide (26).** Preparation according to general procedure B using 8-chloro-2-methylimidazo[1,2-*a*]pyridine-3-amine (**8**, 50.0 mg, 275 µmol, 1.00 eq) and 5-(4-chlorophenyl)furan-2-carboxylic acid (**74**, 74.0 mg, 330 µmol, 1.20 eq) yielded compound **26** as a colorless solid (46 mg, 43%). ^{1}H-NMR (400 MHz, acetone-d_6): δ = 9.87 (s, 1H), 8.14 (d, *J* = 6.7 Hz, 1H), 7.98 (d, *J* = 8.3 Hz, 2H), 7.52 (d, *J* = 8.7 Hz, 2H), 7.40–7.34 (m, 2H), 7.18 (d, *J* = 3.7 Hz, 1H), 6.88 (t, *J* = 7.1 Hz, 1H), 2.36 (s, 3H). ^{13}C-NMR (101 MHz, acetone-d_6): δ = 157.0, 154.9, 146.8, 139.2, 138.8, 134.1, 129.1, 128.5, 126.2, 122.9, 122.6, 121.9, 117.7, 116.7, 111.1, 108.3, 12.4. qHNMR (400 MHz, acetone-d_6, ethyl-4-(dimethylamino)benzoate as reference) purity = 95.2%. MS (APCI+): *m/z* 385.8 ($[M+H]^+$). HRMS (EI+): *m/z* calculated 385.0385 for $C_{19}H_{13}Cl_2N_3O_2$, found 385.0380 ($[M]^{\bullet +}$).

***N*-(8-Chloro-2-methylimidazo[1,2-*a*]pyridin-3-yl)-5-phenylfuran-2-carboxamide (27).** Preparation according to general procedure B using 8-chloro-2-methylimidazo[1,2-*a*]pyridine-3-amine (**8**, 50.0 mg, 275 µmol, 1.00 eq) and 5-phenylfuran-2-carboxylic acid (**79**, 62.0 mg, 330 µmol, 1.20 eq) yielded compound **27** as a colorless solid (66 mg, 68%). ^{1}H-NMR (400 MHz, acetone-d_6): δ = 9.83 (s, 1H), 8.14 (d, *J* = 6.8 Hz, 1H), 7.96 (d, *J* = 7.1 Hz, 2H), 7.49 (t, *J* =7.39 Hz, 2H), 7.45–7.37 (m, 2H), 7.37–7.33 (m, 1H), 7.13 (d, *J* = 3.6 Hz, 1H), 6.88 (t, *J* = 7.1 Hz, 1H), 2.36 (s, 3H). ^{13}C-NMR (101 MHz, acetone-d_6): δ = 157.1, 156.2, 146.6, 139.1, 138.8, 129.7, 128.9, 128.9, 124.6, 122.9, 122.6, 121.9, 117.6, 116.8, 111.1, 107.6, 12.4. qHNMR (400 MHz, acetone-d_6, ethyl-4-(dimethylamino)benzoate as reference): purity = 96.1%. MS (APCI+): *m/z* 351.9 ($[M+H]^+$). HRMS (EI+): *m/z* calculated 351.0775 for $C_{19}H_{14}ClN_3O_2$, found 351.0767 ($[M]^{\bullet +}$).

***N*-(8-Chloro-2-methylimidazo[1,2-*a*]pyridin-3-yl)-5-phenylthiophene-2-carboxamide (28).** Preparation according to general procedure B using 8-chloro-2-methylimidazo[1,2-*a*]pyridine-

3-amine (**8**, 50.0 mg, 275 µmol, 1.00 eq) and commercially available 5-phenylthiophene-2-carboxylic acid (**81**, 67.0 mg, 330 µmol, 1.20 eq) yielded compound **28** as a colorless solid (21 mg, 21%). ^{1}H-NMR (400 MHz, acetone-*d₆*): δ = 9.73 (s, 1H), 8.14 (d, *J* = 1.0 Hz, 1H), 8.08–8.02 (m, 1H), 7.79 (d, *J* = 7.6 Hz, 2H), 7.63–7.57 (m, 1H), 7.55–7.36 (m, 4H), 6.91 (t, *J* = 7.1 Hz, 1H), 2.38 (s, 3H). ^{13}C-NMR (101 MHz, acetone-d_6): δ = 160.8, 150.0, 139.1, 137.1, 133.4, 130.7, 129.3, 128.8, 126.0, 124.2, 123.0, 122.6, 121.9, 118.2, 117.2, 111.1, 12.3. qHNMR (400 MHz, acetone-d_6, ethyl-4-(dimethylamino)benzoate as reference): purity= 96.9%. MS (APCI+): *m/z* 367.3 ([M]$^+$). HRMS (EI+): *m/z* calculated 367.0546 for $C_{19}H_{14}ClN_4OS$, found 367.0539 ([M]•$^+$).

***N*-(8-Chloro-2-methylimidazo[1,2-*a*]pyridin-3-yl)-5-phenyl-1*H*-pyrrole-2-carboxamide (29).** 5-Phenyl-1*H*-pyrrole-2-carboxylic acid (**80**, 74.0 mg, 396 µmol, 1.20 eq) was refluxed in thionyl chloride (4 mL) for 3 h. After cooling to rt, remaining thionyl chloride was removed under reduced pressure. 8-Chloro-2-methylimidazo[1,2-*a*]pyridine-3-amine (**8**, 60.0 mg, 330 µmol, 1.00 eq) dissolved in chloroform (5 mL) was added to the crude acyl chloride at 0°C and the mixture was stirred at rt overnight. The solvent was removed under reduced pressure, the residue was dissolved in ethyl acetate and treated with 5% HCl (5 mL). Phases were separated, the aqueous layer was extracted with ethyl acetate (3x), the combined organic layers were washed with 1 N aqueous NaOH solution, and dried over $MgSO_4$. The solvent was removed under reduced pressure and the crude product was purified by flash column chromatography and reverse column chromatography giving compound **29** as a colorless solid (13 mg, 11%). ^{1}H-NMR (400 MHz, acetone-d_6): δ = 11.85 (s, 1H), 10.51 (s, 1H), 8.31 (d, *J* = 6.7 Hz, 1H), 7.98 (d, *J* = 7.5 Hz, 2H), 7.63 (d, *J* = 7.4 Hz, 1H), 7.41 (t, *J* = 7.7 Hz, 2H), 7.34–7.23 (m, 1H), 7.21–7.06 (m, 2H), 6.75–6.63 (m, 1H), 2.44 (s, 3H). ^{13}C-NMR (101 MHz, acetone-d_6): δ = 159.6, 154.3, 154.1, 137.4, 136.7, 131.9, 128.7, 127.2, 126.2, 126.0, 125.0, 123.7, 118.9, 114.9, 113.1, 107.3, 11.2. qHNMR (400 MHz, acetone-d_6, dimethyl terephthalate as reference): purity = 97.1%. MS (APCI+): *m/z* 350.3 ([M]$^+$). HRMS (ESI+): *m/z* calculated 351.0007 for $C_{19}H_{16}ClN_4O^+$, found 351.10048 ([M+H]$^+$).

***N*-(8-Chloro-2-methylimidazo[1,2-*a*]pyridin-3-yl)-[1,1'-biphenyl]-4-carboxamide (30).** Preparation according to general procedure B using 8-chloro-2-methylimidazo[1,2-*a*]pyridine-3-amine (**8**, 60.0 mg, 330 µmol, 1.00 eq) and [1,1'-biphenyl]-4-carboxylic acid (79.0 mg, 396 µmol, 1.20 eq) yielded compound **30** as a colorless solid (36 mg, 30%). ^{1}H-NMR (400 MHz, acetone-d_6): δ = 9.76 (s, 1H), 8.27–8.20 (m, 2H), 8.16–8.08 (m, 1H), 7.91–7.71 (m, 4H), 7.57–7.48 (m, 2H), 7.48–7.40 (m, 1H), 7.36 (dd, *J* = 7.3, 1.0 Hz, 1H), 6.88 (t, *J* = 7.1 Hz, 1H), 2.37 (s, 3H). ^{13}C-NMR (101 MHz, acetone-d_6): δ = 165.9, 144.7, 139.8, 139.1, 138.4, 132.2, 129.0, 129.0, 128.6, 128.2, 127.1, 127.0 122.8, 122.7, 121.9, 111.0, 12.3. qHNMR (400 MHz, acetone-d_6, ethyl-4-(dimethylamino)benzoate as reference): purity = 95.5%. MS (APCI+): *m/z* 361.3 ([M]$^+$). HRMS (ESI+): *m/z* calculated 362.1055 for $C_{21}H_{17}ClN_3O^+$, found 362.1054 ([M+H]$^+$).

***N*-(8-Chloro-2-methylimidazo[1,2-*a*]pyridin-3-yl)-5-(4-(trifluoromethyl)phenyl)furan-2-carboxamide (31).** Preparation according to general procedure B using 8-chloro-2-methylimidazo[1,2-*a*]pyridine-3-amine (**8**, 50.0 mg, 275 µmol, 1.00 eq) and 5-(4-(trifluoromethyl)phenyl)furan-2-carboxylic acid (**75**, 85.0 mg, 330 µmol, 1.20 eq) yielded compound **31** as a colorless solid (70 mg, 61%). ^{1}H-NMR (400 MHz, acetone-d_6): δ = 9.94 (s,

1H), 8.27–8.08 (m, 3H), 7.83 (d, *J* = 8.3 Hz, 2H), 7.47–7.28 (m, 3H), 6.88 (t, *J* = 7.1 Hz, 1H), 2.36 (s, 3H). ^{13}C-NMR (126 MHz, MeOD-*d$_4$*): δ = 158.5, 155.3, 146.5, 139.5, 138.2, 132.9, 130.1 (q, *J* = 32.5 Hz), 125.6 (q, *J* = 3.8 Hz), 124.9, 124.2, 124.1 (q, *J* = 271.43), 122.2, 121.4, 118.2, 116.5, 112.0, 109.3, 11.2. qHNMR (400 MHz, acetone-*d$_6$*, ethyl-4-(dimethylamino)benzoate as reference): purity= 98.9%. MS (APCI+): *m/z* 419.0 ([M]$^+$). HRMS (EI+): *m/z* calculated 419.0648 for $C_{20}H_{13}ClF_3N_3O_2$, found 419.0646 ([M]•$^+$).

***N*-(8-Chloro-2-methylimidazo[1,2-*a*]pyridin-3-yl)-5-(*p*-tolyl)furan-2-carboxamide (32).** Preparation according to general procedure B using 8-chloro-2-methylimidazo[1,2-*a*]pyridine-3-amine (**8**, 50.0 mg, 275 µmol, 1.00 eq) and 5-(*p*-tolyl)furan-2-carboxylic acid (**76**, 67.0 mg, 330 µmol, 1.20 eq) yielded compound **32** as a colorless solid (69 mg, 69%). ^{1}H-NMR (400 MHz, acetone-*d$_6$*): δ = 9.78 (s, 1H), 8.13 (d, *J* = 6.7 Hz, 1H), 7.85 (d, *J* = 7.9 Hz, 2H), 7.40–7.33 (m, 2H), 7.31 (d, *J* = 8.0 Hz, 2H), 7.06 (d, *J* = 3.6 Hz, 1H), 6.88 (t, *J* = 7.1 Hz, 1H), 2.43–2.31 (m, 6H). ^{13}C-NMR (101 MHz, acetone-*d$_6$*): δ = 157.1, 156.5, 146.2, 139.1, 138.9, 138.8, 129.5, 127.0, 124.6, 122.9, 122.6, 121.9, 117.7, 116.8, 111.0, 106.9, 20.4, 12.4. qHNMR (400 MHz, acetone-*d$_6$*, ethyl-4-(dimethylamino)benzoate as reference) purity = 97.8%. MS (APCI+): *m/z* 365.8 ([M+H]$^+$). HRMS (EI+): *m/z* calculated 365.0931 for $C_{20}H_{16}ClN_3O_2$, found 365.0923 ([M]•$^+$).

***N*-(8-Chloro-2-methylimidazo[1,2-*a*]pyridin-3-yl)-5-(4-(trifluoromethoxy)phenyl)furan-2-carboxamide (33).** Preparation according to general procedure B using 8-chloro-2-methylimidazo[1,2-*a*]pyridine-3-amine (**8**, 50.0 mg, 275 µmol, 1.00 eq) and 5-(4-(trifluoromethoxy)phenyl)furan-2-carboxylic acid (**77**, 90.0 mg, 330 µmol, 1.20 eq) yielded compound **33** as a colorless solid (85 mg, 71%). ^{1}H-NMR (400 MHz, acetone-*d$_6$*): δ = 9.86 (s, 1H), 8.19–8.09 (m, 3H), 7.48 (d, *J* = 8.4 Hz, 2H), 7.44–7.36 (m, 2H), 7.23 (d, *J* = 3.7 Hz, 1H), 6.90 (t, *J* = 7.1 Hz, 1H), 2.38 (s, 3H). ^{13}C-NMR (126 MHz, acetone-*d$_6$*): δ = 157.0, 154.7, 149.1–148.9 (m), 147.0, 139.2, 138.8, 128.9, 126.4, 123.0, 122.6, 121.9, 121.6, 120.5 (q, *J* = 255.5 Hz) 117.6, 116.7, 111.1, 108.5, 12.3. qHNMR (400 MHz, DMSO-*d$_6$*, ethyl-4-(dimethylamino)benzoate as reference): purity = 95.6%. MS (APCI+): *m/z* 435.9 ([M+H]$^+$). HRMS (ESI+): *m/z* calculated 436.0670 for $C_{20}H_{14}ClF_3N_3O_3^+$, found 436.0663 ([M+H]$^+$).

***N*-(8-Chloro-2-methylimidazo[1,2-*a*]pyridin-3-yl)-5-(4-methoxyphenyl)furan-2-carboxamide (34).** Preparation according to general procedure B using 8-chloro-2-methylimidazo[1,2-*a*]pyridine-3-amine (**8**, 50.0 mg, 275 µmol, 1.00 eq) and 5-(4-methoxyphenyl)furan-2-carboxylic acid (**79**, 72.0 mg, 330 µmol, 1.20 eq) yielded compound **34** as a colorless solid (58 mg, 55%). ^{1}H-NMR (400 MHz, acetone-*d$_6$*): δ = 9.76 (s, 1H), 8.12 (d, *J* = 6.9 Hz, 1H), 7.89 (d, *J* = 8.5 Hz, 2H), 7.41–7.30 (m, 2H), 7.04 (d, *J* = 8.8 Hz, 2H), 7.00–6.95 (m, 1H), 6.88 (t, *J* = 7.1 Hz, 1H), 3.86 (s, 3H), 2.36 (s, 3H). ^{13}C-NMR (101 MHz, acetone-*d$_6$*): δ = 160.5, 157.1, 156.5, 145.9, 139.1, 138.7, 126.2, 122.9, 122.6, 122.5, 121.9, 117.8, 116.9, 114.4, 111.0, 106.0, 54.9, 12.4. qHNMR (400 MHz, acetone-*d$_6$*, ethyl-4-(dimethylamino)benzoate as reference): purity = 95.9%. MS (APCI+): *m/z* 381.6 ([M+H]$^+$). HRMS (EI+): *m/z* calculated 381.0880 for $C_{20}H_{16}ClN_3O_3$, found 381.0875 ([M]•$^+$).

***N*-(8-Chloro-2-methylimidazo[1,2-*a*]pyridin-3-yl)-5-(4-(methylamino)phenyl)furan-2-carboxamide (35).** Preparation according to general procedure B using 8-chloro-2-methylimidazo[1,2-*a*]pyridine-3-amine (**8**, 50.0 mg, 275 µmol, 1.00 eq) and 5-(4-

(methylamino)phenyl)furan-2-carboxylic acid (**72**, 72.0 mg, 330 µmol, 1.20 eq) yielded compound **35** as a yellow solid (35 mg, 33%). ^{1}H-NMR (400 MHz, acetone-*d*$_6$): δ = 9.65 (s, 1H), 8.11 (d, *J* = 1.1 Hz, 1H), 7.71 (d, *J* = 8.3 Hz, 2H), 7.36 (dd, *J* = 7.3, 1.0 Hz, 1H), 7.33–7.28 (m, 1H), 6.87 (t, *J* = 7.0 Hz, 1H), 6.79 (d, *J* = 3.6 Hz, 1H), 6.68 (d, *J* = 8.5 Hz, 2H), 5.41 (s, 1H), 2.85 (s, 3H), 2.35 (s, 3H). ^{13}C-NMR (400 MHz, DMSO-*d*$_6$): δ = 157.9, 157.7, 151.1, 144.5, 138.8, 138.3, 126.5, 123.7, 123.4, 121.3, 118.8, 117.6, 117.1, 111.9, 111.8, 104.5, 29.9, 13.4. qHNMR (400 MHz, acetone-*d*$_6$, maleic acid as reference): purity = 96.2%. MS (APCI+): *m/z* 380.7 ($[M+H]^+$). HRMS (ESI+): *m/z* calculated 381.1113 for $C_{20}H_{18}ClN_4O_2^+$, found 381.1110 ($[M+H]^+$).

***N*-(8-Chloro-2-methylimidazo[1,2-*a*]pyridin-3-yl)-5-(4-(dimethylamino)phenyl)furan-2-carboxamide (36).** Preparation according to general procedure B using 8-chloro-2-methylimidazo[1,2-*a*]pyridine-3-amine (**8**, 50.0 mg, 275 µmol, 1.00 eq) and 5-(4-(dimethylamino)phenyl)furan-2-carboxylic acid (**73**, 76.0 mg, 330 µmol, 1.20 eq) yielded compound **36** as a yellow solid (69 mg, 64%). ^{1}H-NMR (400 MHz, acetone-*d*$_6$) δ = 9.68 (s, 1H), 8.11 (d, *J* = 1.0 Hz, 1H), 7.77 (d, *J* = 8.5 Hz, 2H), 7.40–7.27 (m, 2H), 6.94–6.73 (m, 4H), 3.00 (s, 6H), 2.36 (s, 3H). ^{13}C-NMR (101 MHz, acetone-*d*$_6$) δ = 157.7, 157.2, 151.0, 145.1, 139.1, 138.7, 125.9, 122.8, 122.6, 121.8, 118.0, 117.6, 117.0, 112.0, 111.0, 104.3, 39.4, 12.4. qHNMR (400 MHz, acetone-*d*$_6$, maleic acid as reference) purity = 95.1%. MS (APCI+): *m/z* 394.7 ($[M+H]^+$). HRMS (EI+): *m/z* calculated 394.1197 for $C_{21}H_{19}ClN_4O_2$, found 394.1194 ($[M]^{\bullet +}$).

***N*-(8-Chloro-2-(3,4-dichlorophenyl)imidazo[1,2-*a*]pyridin-3-yl)-5-(4-(dimethylamino) phenyl)furan-2-carboxamidearboxamide (37).** *N,N*-Dimethyl-4-(4,4,5,5-tetramethyl-1,3,2-dioxaborolan-2-yl)aniline (**69,** 40.8 mg, 0.165 mmol, 1.00 eq), 5-bromo-*N*-(8-chloro-2-(3,4-dichlorophenyl)imidazo[1,2-*a*]pyridin-3-yl)furan-2-carboxamide (**84**, 80.0 mg, 0.165 mmol, 1.00 eq) and sodium carbonate (52.5 mg, 3.00 mol, 3.00 eq) were dissolved in dioxane/H_2O (10 mL, 9:1). The solution was degassed by freeze-pump-thaw cycles (3x). $Pd(PPh_3)_4$ (9.53 mg, 8.25 µmol, 0.05 eq) was added and the mixture was refluxed for 3 h under argon atmosphere. The resulting suspension was filtered, and the precipitate was washed with 2 N aqueous NaOH solution, EtOH, methylene chloride and brine giving **37** as a colorless solid (59 mg, 68%). ^{1}H-NMR (400 MHz, DMSO-*d*$_6$): δ = 10.86 (s, 1H), 8.27–8.17 (m, 2H), 8.02–7.94 (m, 1H), 7.85–7.72 (m, 3H), 7.60 (d, *J* = 7.3 Hz, 1H), 7.57–7.50 (m, 1H), 7.02–6.92 (m, 2H), 6.80 (d, *J* = 8.8 Hz, 2H), 2.98 (s, 6H). ^{13}C-NMR (126 MHz, DMSO-*d*$_6$): δ = 158.0, 151.1, 144.5, 139.7, 136.5, 136.0, 134.1, 132.0, 131.6, 128.6, 127.1, 126.4, 125.6, 124.1, 122.0, 119.4, 117.3, 115.3, 113.0, 112.4, 110.4, 105.2, 31.2. qHNMR (400 MHz, DMSO-*d*$_6$, maleic acid as reference): purity = 96.1%. MS (APCI+): *m/z* 524.2 ($[M+H]^{\bullet +}$). HRMS (ESI+): *m/z* calculated 525.0646 for $C_{26}H_{20}Cl_3N_4O_2^+$, found 525.0641 ($[M+H]^+$).

5-(4-Aminophenyl)-*N*-(8-chloro-2-methylimidazo[1,2-*a*]pyridin-3-yl)furan-2-carboxamide (38). 4-(4,4,5,5-Tetramethyl-1,3,2-dioxaborolan-2-yl)aniline (**85,** 37 mg, 0.169 mmol, 1.00 eq), 5-bromo-*N*-(8-chloro-2-methylimidazo[1,2-*a*]pyridin-3-yl)furan-2-carboxamide (**84**, 60.0 mg, 0.169 mmol, 1.00 eq) and sodium carbonate (53.7 mg, 3.00 mol, 3.00 eq) were dissolved in dioxane/H_2O (10 mL, 9:1). The solution was degassed by freeze-pump-thaw cycles (3x). $Pd(PPh_3)_4$ (9.76 mg, 8.45 µmol, 0.05 eq) was added and the mixture was refluxed for 3 h under argon atmosphere. The resulting suspension was filtered through Celite and the solvents were

removed under reduced pressure. The residue was dissolved in 2 N aqueous NaOH solution and extracted with ethyl acetate (3x). The combined organic layers were dried over $MgSO_4$ and the solvent was removed under reduced pressure. The crude product was purified by flash column chromatography, reverse column chromatography and preparative HPLC giving **38** as a yellow solid (25 mg, 40%). ^{1}H-NMR (400 MHz, DMSO-*d*$_6$) δ = 10.30 (s, 1H), 8.07 (d, *J* = 6.7 Hz, 1H), 7.65 (d, *J* = 8.2 Hz, 2H), 7.44 (d, *J* = 7.3 Hz, 1H), 7.37 (d, *J* = 3.6 Hz, 1H), 6.88 (t, *J* = 7.1 Hz, 1H), 6.82 (d, *J* = 3.6 Hz, 1H), 6.63 (d, *J* = 8.3 Hz, 2H), 5.54 (s, 2H), 2.31 (s, 3H). ^{13}C-NMR (101 MHz, DMSO-*d*$_6$) δ = 158.0, 157.7, 150.3, 144.4, 138.8, 138.3, 126.5, 123.7, 123.4, 121.3, 118.8, 117.6, 117.3, 114.1, 111.9, 104.4, 13.4. qHNMR (400 MHz, DMSO-*d*$_6$, maleic acid as reference) purity = 99.1%. MS (APCI+): *m/z* 366.6 ($[M+H]^{\bullet +}$). HRMS (ESI+): *m/z* calculated 367.0956 for $C_{19}H_{16}ClN_4O_2^+$, found 367.0956 ($[M+H]^+$).

***N*-(8-Chloro-2-methylimidazo[1,2-*a*]pyridin-3-yl)-5-(4-morpholinophenyl)furan-2-carboxamide (39).** 4-(4-(4,4,5,5-Tetramethyl-1,3,2-dioxaborolan-2-yl)phenyl)morpholine (**86,** 48.9 mg, 0.169 mmol, 1.00 eq), 5-bromo-*N*-(8-chloro-2-methylimidazo[1,2-*a*]pyridin-3-yl)furan-2-carboxamide (**84**, 60.0 mg, 0.169 mmol, 1.00 eq) and sodium carbonate (53.7 mg, 3.00 mol, 3.00 eq) were dissolved in dioxane/H_2O (10 mL, 9:1). The solution was degassed by freeze-pump-thaw cycles (3x). $Pd(PPh_3)_4$ (9.76 mg, 8.45 µmol, 0.05 eq) was added and the mixture was refluxed for 3 h under argon atmosphere. The resulting suspension was filtered through Celite and the solvents were removed under reduced pressure. The residue was dissolved in 2 N aqueous NaOH solution and extracted with ethyl acetate (3x). The combined organic layers were dried over $MgSO_4$ and the solvent was removed under reduced pressure. The crude product was purified by flash column chromatography and reverse column chromatography giving **39** as a yellow solid (33 mg, 45%). ^{1}H-NMR (400 MHz, acetone-*d*$_6$): δ = 9.72 (s, 1H), 8.12 (d, *J* = 1.1 Hz, 1H), 7.82 (d, *J* = 8.5 Hz, 2H), 7.40–7.31 (m, 2H), 7.05 (d, *J* = 8.8 Hz, 2H), 6.95–6.84 (m, 2H), 3.81–3.76 (m, 4H), 3.27–3.21 (m, 4H), 2.36 (s, 3H). ^{13}C-NMR (101 MHz, acetone-*d*$_6$): δ = 157.9, 152.8. 152.5, 146.4, 140.0, 139.6, 126.7, 123.7, 123.5, 122.7, 121.4, 118.8, 117.8, 115.8, 111.9, 106.2, 67.2, 49.1, 13.3. qHNMR (400 MHz, acetone-*d*$_6$, ethyl-4(dimethylamino)benzoate as reference): purity = 95.2%. MS (APCI+): *m/z* 436.2 ($[M]^{\bullet +}$). HRMS (ESI+): *m/z* calculated $C_{23}H_{22}ClN_4O_3^+$ for 437.1375, found 437.1369 ($[M+H]^+$).

***N*-(8-Chloro-2-methylimidazo[1,2-*a*]pyridin-3-yl)-5-(4-(4-methylpiperazin-1-yl)phenyl)furan-2-carboxamide (40).** 1-Methyl-4-(4-(4,4,5,5-tetramethyl-1,3,2-dioxaborolan-2-yl)phenyl)piperazine (**87,** 51.1 mg, 0.169 mmol, 1.00 eq), 5-bromo-*N*-(8-chloro-2-methylimidazo[1,2-*a*]pyridin-3-yl)furan-2-carboxamide (**84**, 60.0 mg, 0.169 mmol, 1.00 eq) and sodium carbonate (53.7 mg, 3.00 mol, 3.00 eq) were dissolved in dioxane/H_2O (10 mL, 9:1). The solution was degassed by freeze-pump-thaw cycles (3x). $Pd(PPh_3)_4$ (9.76 mg, 8.45 µmol, 0.05 eq) was added and the mixture was refluxed for 3 h under argon atmosphere. The resulting suspension was filtered through Celite and the solvents were removed under reduced pressure. The residue was dissolved in 2 N aqueous NaOH solution and extracted with ethyl acetate (3x). The combined organic layers were dried over $MgSO_4$ and the solvent was removed under reduced pressure. The crude product was purified by flash column chromatography and reverse column chromatography giving **40** as a yellow solid (30 mg, 40%). ^{1}H-NMR (400 MHz, acetone-*d*$_6$): δ = 9.71 (s, 1H), 8.12 (d, *J* = 6.6 Hz, 1H), 7.80 (d, *J* = 8.4 Hz, 2H), 7.39–7.30 (m, 2H), 7.03 (d, *J* = 8.7 Hz, 2H), 6.92–6.83 (m, 2H), 3.31–3.24 (m, 4H), 2.53–2.46 (m, 4H), 2.35 (s, 3H), 2.26 (s, 3H). ^{13}C-NMR (101 MHz, acetone-*d*$_6$): δ = 157.1,

151.8, 145.5, 139.1, 138.7, 125.8, 122.8, 122.6, 121.8, 120.0, 117.9, 117.0, 115.0, 111.0, 105.1, 54.8, 47.8, 45.5, 12.4. qHNMR (400 MHz, acetone-*d*$_6$, ethyl-4(dimethylamino)benzoate as reference): purity = 96.5%. MS (APCI+): *m/z* 449.5 ($[M+H]^+$). HRMS (ESI+): *m/z* calculated $C_{24}H_{25}ClN_5O_2^+$ for 450.1691, found 450.1686 ($[M+H]^+$).

***N*-(8-Chloro-2-methylimidazo[1,2-*a*]pyridin-3-yl)-5-(4-isopropoxyphenyl)furan-2-carboxamide (41).** 4-Isopropyloxyphenylboronic acid (**88**, 30.4 mg, 169 mmol, 1.00 eq), 5-bromo-*N*-(8-chloro-2-methylimidazo[1,2-*a*]pyridin-3-yl)furan-2-carboxamide (**84**, 60.0 mg, 0.169 mmol, 1.00 eq) and sodium carbonate (53.7 mg, 3.00 mol, 3.00 eq) were dissolved in dioxane/H_2O (10 mL, 9:1). The solution was degassed by freeze-pump-thaw cycles (3x). $Pd(PPh_3)_4$ (9.76 mg, 8.45 µmol, 0.05 eq) was added and the mixture was refluxed for 3 h under argon atmosphere. The resulting suspension was filtered through Celite and the solvents were removed under reduced pressure. The residue was dissolved in 2 N aqueous NaOH solution and extracted with ethyl acetate (3x). The combined organic layers were dried over $MgSO_4$ and the solvent was removed under reduced pressure. The crude product was purified by flash column chromatography and reverse column chromatography giving **41** as a colorless solid (47 mg, 68%). ^{1}H-NMR (500 MHz, acetone-*d*$_6$): δ = 9.76 (s, 1H), 8.13 (d, *J* = 6.8 Hz, 1H), 7.87 (d, *J* = 8.3 Hz, 2H), 7.39–7.32 (m, 2H), 7.02 (d, *J* = 8.4 Hz, 2H), 6.98–6.94 (m, 1H), 6.88 (t, *J* = 7.0 Hz, 1H), 4.77–4.66 (m, 1H), 2.36 (s, 3H), 1.35–1.30 (m, 6H). ^{13}C-NMR (126 MHz, acetone-*d*$_6$): δ = 158.8, 157.1, 156.6, 145.8, 139.1, 138.7, 126.3, 122.8, 122.6, 122.2, 121.9, 117.8, 116.9, 116.0, 111.0, 105.9, 69.6, 21.3, 12.4. qHNMR (400 MHz, acetone-*d*$_6$, ethyl-4(dimethylamino)benzoate as reference): purity = 99.9%. MS (APCI+): *m/z* 409.7 ($[M+H]^+$). HRMS (ESI+): *m/z* calculated $C_{22}H_{21}ClN_3O_3^+$ for 410.1266, found 410.1260 ($[M+H]^+$).

***N*-(8-Chloro-2-methylimidazo[1,2-*a*]pyridin-3-yl)-5-(4-cyclopropoxyphenyl)furan-2-carboxamide (42).** 4-Cyclopropyloxyphenylboronic acid (**89**, 30.1 mg, 169 mmol, 1.00 eq), 5-bromo-*N*-(8-chloro-2-methylimidazo[1,2-*a*]pyridin-3-yl)furan-2-carboxamide (**84**, 60.0 mg, 0.169 mmol, 1.00 eq) and sodium carbonate (53.7 mg, 3.00 mol, 3.00 eq) were dissolved in dioxane/H_2O (10 mL, 9:1). The solution was degassed by freeze-pump-thaw cycles (3x). $Pd(PPh_3)_4$ (9.76 mg, 8.45 µmol, 0.05 eq) was added and the mixture was refluxed for 3 h under argon atmosphere. The resulting suspension was filtered through Celite and the solvent was removed under reduced pressure. The residue was dissolved in 2 N aqueous NaOH and extracted with ethyl acetate (3x). The combined organic layers were dried over $MgSO_4$ and the solvent was removed under reduced pressure. The crude product was purified by flash column chromatography and reverse column chromatography giving **42** as a colorless solid (45 mg, 65%). ^{1}H-NMR (400 MHz, acetone-*d*$_6$): δ = 9.76 (s, 1H), 8.13 (d, *J* = 1.0 Hz, 1H), 7.89 (d, *J* = 8.5 Hz, 2H), 7.41–7.29 (m, 2H), 7.22–7.09 (m, 2H), 6.98 (d, *J* = 3.6 Hz, 1H), 6.88 (t, *J* = 6.8 Hz, 1H), 3.93–3.85 (m, 1H), 2.36 (s, 3H), 0.89–0.67 (m, 4H). ^{13}C-NMR (126 MHz, acetone-*d*$_6$): δ = 159.8, 157.1, 156.5, 145.9, 139.1, 138.7, 126.1, 122.9, 122.8, 122.6, 121.9, 117.8, 116.9, 115.4, 111.0, 106.0, 50.8, 12.4, 5.7. qHNMR (400 MHz, acetone-*d*$_6$, ethyl-4(dimethylamino)benzoate as reference): purity = 97.5%. MS (APCI+): *m/z* 407.7 ($[M+H]^+$). HRMS (ESI+): *m/z* calculated 408.1109 for $C_{22}H_{19}ClN_3O_3^+$, found 408.1103 ($[M+H]^+$).

8-chloro-3-nitroimidazo[1,2-*a*]pyridine (61). 8-Chloroimidazo[1,2-*a*]pyridine (**60**, 300 mg, 1.97 mmol, 1.00 eq) was dissolved in conc. sulfuric acid (10 mL) and cooled to 0°C. Nitric acid (65%, 358 µL, 5.60 mmol, 2.80 eq.) was added and the mixture was stirred for 1 h at 0°C. The

solution was then slowly poured into a H_2O-ice mixture leading to precipitation of the product. The precipitate was washed with cold H_2O to yield **61** as a yellow solid (363 mg, 93%). ^{1}H-NMR (400 MHz, acetone-d_6): δ = 9.39 (dd, *J* = 7.0, 1.0 Hz, 1H), 8.68 (s, 1H), 7.93 (dt, *J* = 7.7, 0.8 Hz, 1H), 7.48 (t, *J* = 7.3 Hz, 1H). ^{13}C-NMR (101 MHz, acetone-d_6): 145.0, 138.5, 130.3, 127.5, 124.7, 120.1, 117.6. MS (APCI+): *m/z* 197.7 ($[M+H]^+$).

Methyl 5-(4-(methylamino)phenyl)furan-2-carboxylate (66). *N*-Methyl-4-(4,4,5,5-tetramethyl-1,3,2-dioxaborolan-2-yl)aniline (**64,** 250 mg, 1.10 mmol, 1.10 eq) and tripotassium phosphate (637 mg, 3.00 mol, 3.00 eq) were dissolved in dioxane/H_2O (10 mL, 9:1). The solution was degassed by freeze-pump-thaw cycles (3x). Methyl 5-bromofuran-2-carboxylate (**63**, 205 mg, 1.00 mmol, 1.00 eq) and XPhos Pd G2 (79.0 mg, 0.10 mmol, 0.10 eq) were added and the mixture was refluxed for 3 h under nitrogen atmosphere. The resulting suspension was filtered through Celite and the solvents were removed under reduced pressure. The residue was dissolved in 2 N aqueous HCl and extracted with ethyl acetate (3x). The combined organic layers were dried over $MgSO_4$ and the solvent was removed under reduced pressure. The crude product was purified by flash column chromatography using a gradient of cyclohexane/ethyl acetate as mobile phase giving **66** as a brown solid (200 mg, 87%). ^{1}H-NMR (400 MHz, acetone-d_6): δ = 7.63–7.57 (m, 2H), 7.24 (d, *J* = 0.6 Hz, 1H), 6.72–6.65 (m, 3H), 5.42 (s, 1H), 3.84 (s, 3H), 2.83 (s, 3H). ^{13}C-NMR (101 MHz, acetone-d_6): δ = 159.0, 158.6, 151.0, 142.1, 126.0, 120.3, 117.6, 111.8, 103.7, 50.8, 24.4. MS (APCI+): *m/z* 231.8 ($[M+H]^+$).

Methyl 5-(4-(dimethylamino)phenyl)furan-2-carboxylate (67). 4-(Dimethylamino)phenylboronic acid (**65,** 198 mg, 1.20 mmol, 1.20 eq) and tripotassium phosphate (637 mg, 3.00 mmol, 3.00 eq) were dissolved in dioxane/H_2O (10 mL, 9:1). The solution was degassed by freeze-pump-thaw cycles (3x). Methyl 5-bromofuran-2-carboxylate (**63**, 205 mg, 1.00 mmol, 1.00 eq) and XPhos Pd G2 (79.0 mg, 0.10 mmol, 0.10 eq) were added and the mixture was refluxed for 3 h. The resulting suspension was filtered through Celite and the solvents were removed under reduced pressure. The residue was dissolved in 2 N aqueous HCl and extracted with ethyl acetate (3x). The combined organic layers were dried over $MgSO_4$ and the solvent was removed under reduced pressure. The crude product was purified by flash column chromatography using a gradient of cyclohexane/ethyl acetate as mobile phase giving **67** as a brown solid (237 mg, 97%). ^{1}H-NMR (400 MHz, acetone-d_6): δ = 7.70–7.61 (m, 2H), 7.26 (d, *J* = 3.6 Hz, 1H), 6.85–6.78 (m, 2H), 6.74 (d, *J* = 3.7 Hz, 1H), 3.84 (s, 3H), 3.01 (s, 6H). ^{13}C-NMR (101 MHz, acetone-d_6): δ = 159.7, 159.5, 152.0, 143.1, 126.7, 121.2, 118.3, 112.9, 104.9, 51.8, 40.2. MS (APCI+): *m/z* 245.8 ($[M+H]^+$).

5-(4-(Methylamino)phenyl)furan-2-carboxylic (68). Methyl 5-(4-(methylamino)phenyl)furan-2-carboxylate (**66**, 180 mg, 778 µmol, 1.00 eq) and LiOH (93.0 mg, 3.89 mmol, 5.00 eq) were dissolved in H_2O/THF (10 mL, 1:1). The mixture was stirred at rt overnight. The solvents were removed under reduced pressure and the crude product was purified by reverse column chromatography using a gradient of H_2O/MeCN as mobile phase giving **68** as a colorless solid (113 mg, 67%). ^{1}H-NMR (400 MHz, DMSO-d_6): δ = 7.45 (d, *J* = 8.7 Hz, 2H), 6.65–6.62 (m, 1H), 6.60–6.52 (m, 2H), 6.48 (d, *J* = 3.3 Hz, 1H), 5.93–5.85 (m, 1H), 2.69 (d, *J* = 5.0 Hz, 3H). ^{13}C-NMR (101 MHz, DMSO-d_6): δ = 171.9, 162.6, 153.8, 149.9, 125.3, 119.2, 113.6, 112.1, 103.3, 30.1. MS (APCI+): *m/ z* 217.9 ($[M+H]^+$).

5-(4-(Dimethylamino)phenyl)furan-2-carboxylic acid (69). Methyl 5-(4-(dimethylamino)phenyl)furan-2-carboxylate (**67**, 189 mg, 771 µmol, 1.00 eq) and LiOH (92.0 mg, 3.89 mmol, 5.00 eq) were dissolved in H_2O/THF (10 mL, 1:1). The solution was stirred at rt overnight. The solvents were removed under reduced pressure and the crude product was purified by reverse column chromatography using a gradient of H_2O/MeCN as mobile phase giving **69** as a colorless solid (130 mg, 73%). ^{1}H-NMR (400 MHz, DMSO-*d*$_6$) δ = 7.57–7.48 (m, 2H), 6.79–6.71 (m, 2H), 6.63 (d, *J* = 3.2 Hz, 1H), 6.54 (d, *J* = 3.2 Hz, 1H), 2.93 (s, 6H). ^{13}C-NMR (101 MHz, MeOH-*d*$_6$) δ = 166.0, 156.0, 150.5, 148.6, 125.2, 119.3, 115.6, 112.2, 103.0, 39.3. MS (APCI+): *m/z* 232.1 ([M+H]$^+$).

5-Bromo-*N*-(8-chloro-2-(3,4-dichlorophenyl)imidazo[1,2-*a*]pyridin-3-yl)furan-2-carboxamide (83). 5-Bromofuran-2-carboxylic acid (**82**, 345 mg, 1.81 mmol, 3.14 eq) was dissolved in methylene chloride (2 mL) under Ar atmosphere. Oxalyl chloride (310 µL, 3.62 mmol, 6.3 eq) was added dropwise to the solution at 0 °C. After 3 h the solvent was removed under reduced pressure, 8-chloro-2-(3,4-dichlorophenyl)imidazo[1,2-*a*]pyridine-3-amine (**24**, 180 mg, 576 mmol, 1.00 eq) dissolved in a mixture of pyridine (1 mL) and toluene (4 mL) was added, and the mixture was stirred at rt overnight. 2 N aqueous NaOH solution (10 mL) was added, phases were separated, and the aqueous layer was extracted with ethyl acetate (3x). The combined organic layers were dried over $MgSO_4$ and the solvent was removed under reduced pressure. The crude product was purified by flash column chromatography using a gradient of cyclohexane/ ethyl acetate as mobile phase giving **83** as a brown solid (180 mg, 64%). ^{1}H-NMR (400 MHz, DMSO-*d*$_6$): δ = 10.87 (s, 1H), 8.29–8.24 (m, 1H), 8.15 (d, *J* = 2.0 Hz, 1H), 7.92 (dd, *J* = 8.4, 2.1 Hz, 1H), 7.76 (d, *J* = 8.4 Hz, 1H), 7.62–7.57 (m, 1H), 7.50 (d, *J* = 3.6 Hz, 1H), 7.02–6.92 (m, 2H). ^{13}C-NMR (101 MHz, DMSO-*d*$_6$): δ = 157.0, 148.8, 139.8, 136.5, 133.9, 132.0, 131.7, 131.1, 128.6, 127.1, 127.1, 125.7, 124.1, 122.0, 119.2, 117.2, 115.2, 113.1. MS (APCI+): *m/z* 483.3 ([M+H]$^+$)

5-Bromo-*N*-(8-chloro-2-methylimidazo[1,2-*a*]pyridin-3-yl)furan-2-carboxamide (84). Preparation according to general procedure B using 8-chloro-2-methylimidazol[1,2-*a*]pyridine-3-amine (**8**, 340 mg, 1.87 mmol, 1.00 eq) and 5-bromofuran-2-carboxylic acid (**82**, 429 mg, 2.24 mmol, 1.20 eq) yielded compound **84** as a colorless solid (536 mg, 81%). ^{1}H-NMR (400 MHz, MeOD-*d*$_4$): δ = 8.00 (d, *J* = 1.0 Hz, 1H), 7.43 (d, *J* = 1.0 Hz, 1H), 7.33 (d, *J* = 3.6 Hz, 1H), 6.91 (t, *J* = 7.1 Hz, 1H), 6.73 (d, *J* = 3.7 Hz, 1H), 2.39 (s, 3H). ^{13}C-NMR (101 MHz, MeOD-*d*$_4$): δ = 157.5, 148.5, 139.5, 138.1, 126.6, 124.2, 122.2, 121.4, 118.5, 116.3, 114.4, 111.9, 11.2. MS (APCI+): *m/z* 355.4 ([M+H]$^+$).

Supplementary References

1. Almario Garcia, A., Lardenois, P. & Olivier, A. Derivatives of 2-aryl-6-phenyl-imid azo [1, 2-α]pyridines, their preparation and their therapeutic use. WO 2008/034974A1. *Sanofi-Aventis* (2008).
2. Pauli, G. F. *et al.* Importance of purity evaluation and the potential of quantitative 1H NMR as a purity assay. *J. Med. Chem.* **57**, 9220–9231 (2014).
