# Supplementary material for: Development of Nurr1 agonists from amodiaquine by scaffold hopping and fragment growing
Source: Commun Chem. 2024 Jun 29;7:149. doi: 10.1038/s42004-024-01224-0 (PMC11217349; doi:10.1038/s42004-024-01224-0)
Supplement: Supplementary file 2 — Description of Additional Supplementary Files [file 42004_2024_1224_MOESM2_ESM.pdf]

Description of Additional Supplementary Files

File name: Supplementary Data 1

Description: NMR spectra (1H, 13C and qH) and HRMS of compounds 8-1 9, 12, 14-42.

File name: Supplementary Data 2

Description: Source data for Figures 2, 4 and 5.
