# Supplementary material for: Development of Nurr1 agonists from amodiaquine by scaffold hopping and fragment growing
Source: Commun Chem. 2024 Jun 29;7:149. doi: 10.1038/s42004-024-01224-0 (PMC11217349; doi:10.1038/s42004-024-01224-0)

- Supplementary Data 1-

Development of Nurr1 Agonists from Amodiaquine By Scaffold Hopping and Fragment Growing

Minh Sai[1], Emily C. Hank[1], Hin-Man Tai[2], Till Kasch[1], Max Lewandowski[1], Michelle Vincendeau[2,3], Julian A. Marschner[1], Daniel Merk[1]*

[1] Ludwig-Maximilians-Universität München, Department of Pharmacy, 81377 Munich, Germany
[2] Helmholtz Munich, Institute of Virology, 85764 Munich, Germany
[3] Technical University of Munich, Institute of Virology, School of Medicine, 81675 Munich, Germany
* daniel.merk@cup.lmu.de

Table of Contents

NMR spectra (^{1}H, ^{13}C and qH) of **8-9, 12, 14-42** 2
HRMS spectra of **8-9, 12, 14-42** 98

NMR spectra (^{1}H, ^{13}C and gH) of **8-9, 12** and **14-42**

^{1}H spectrum of compound **8:**

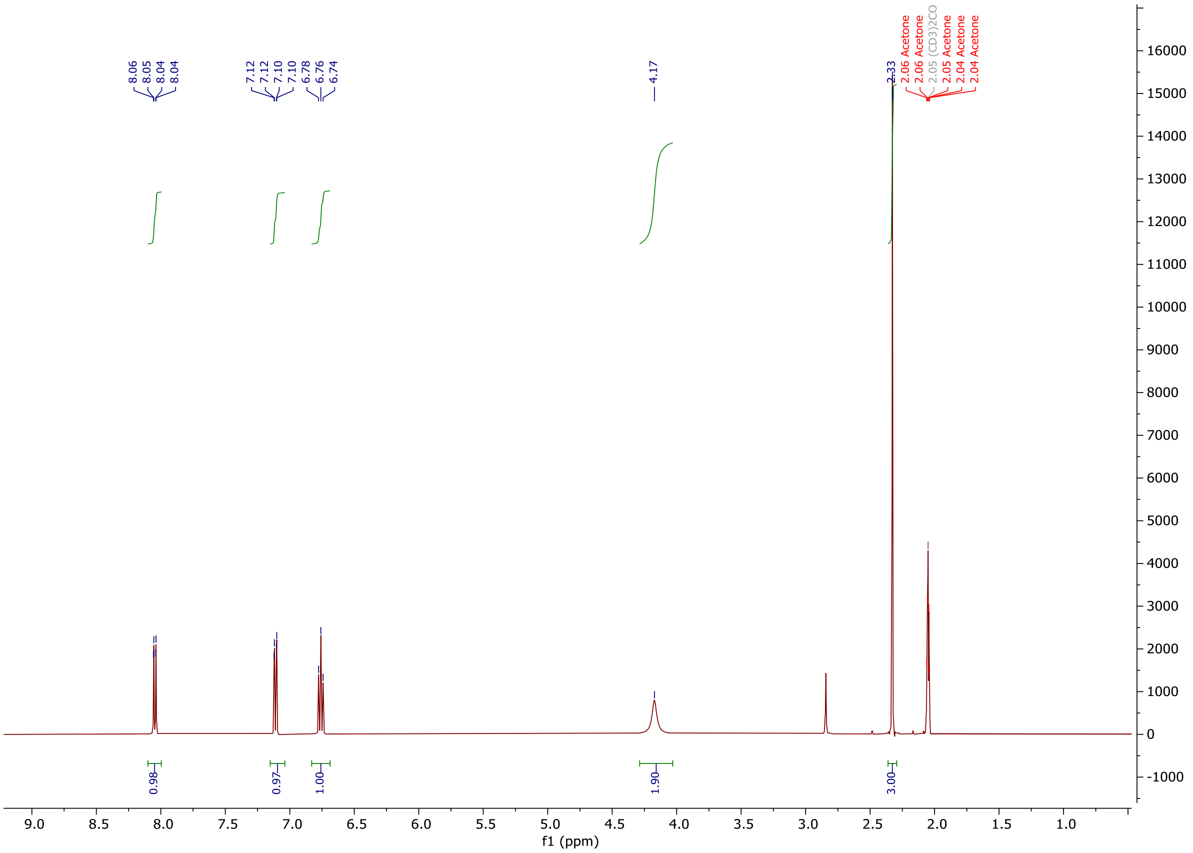

^{13}C spectrum of compound **8:**

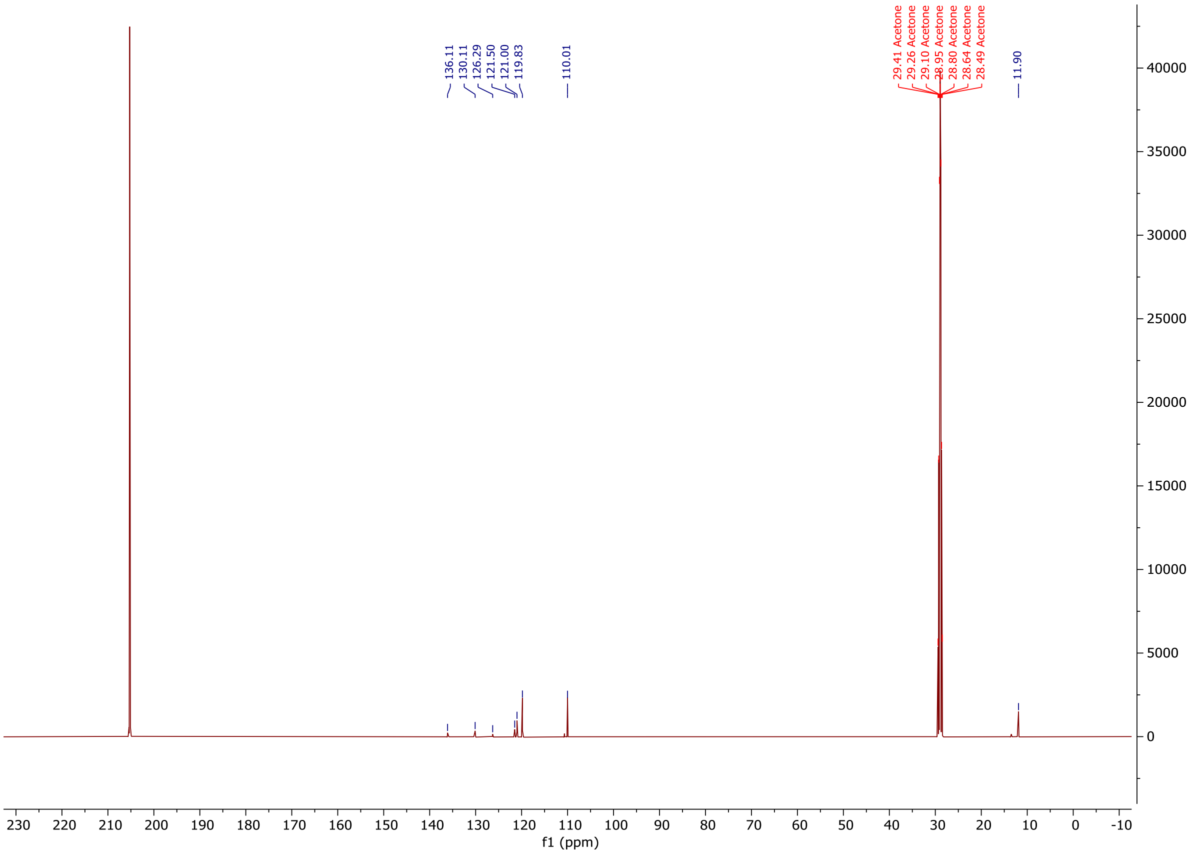

qHNMR spectrum of compound **8:**

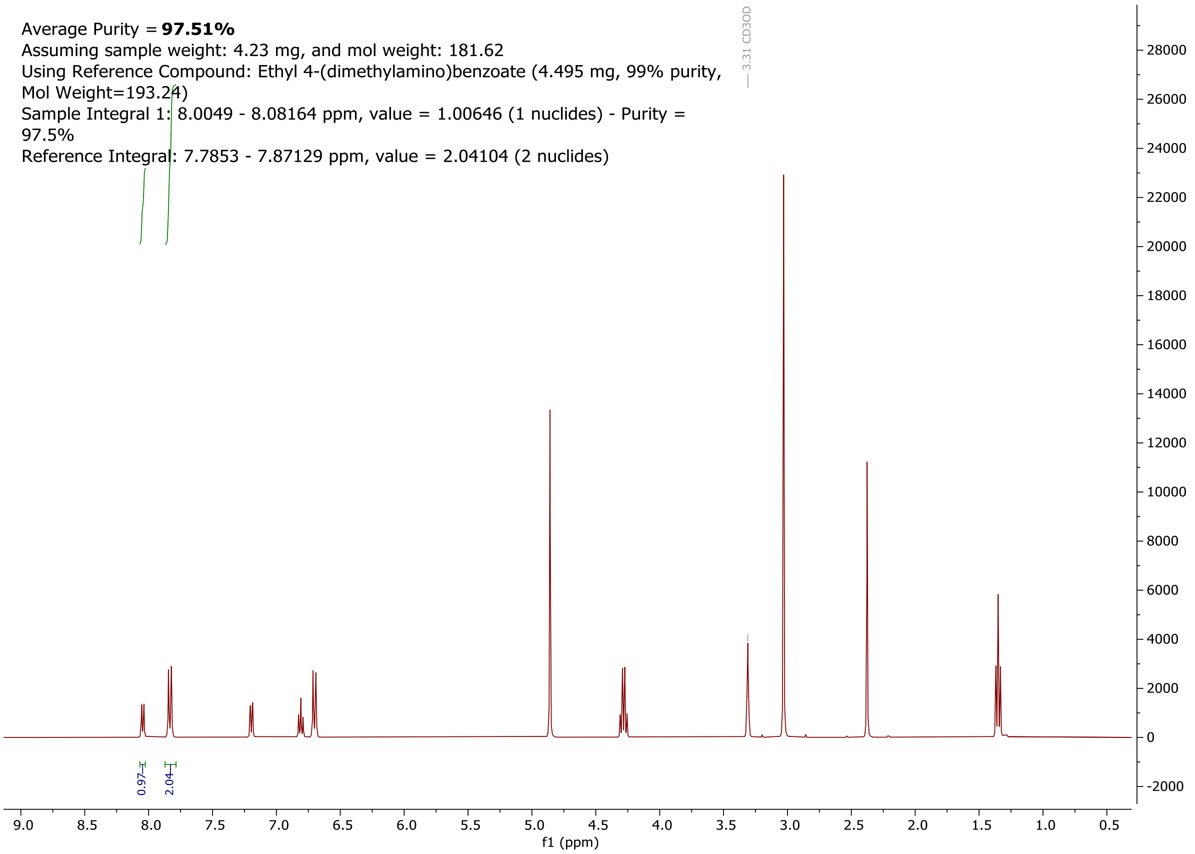

^{1}H spectrum of compound **9:**

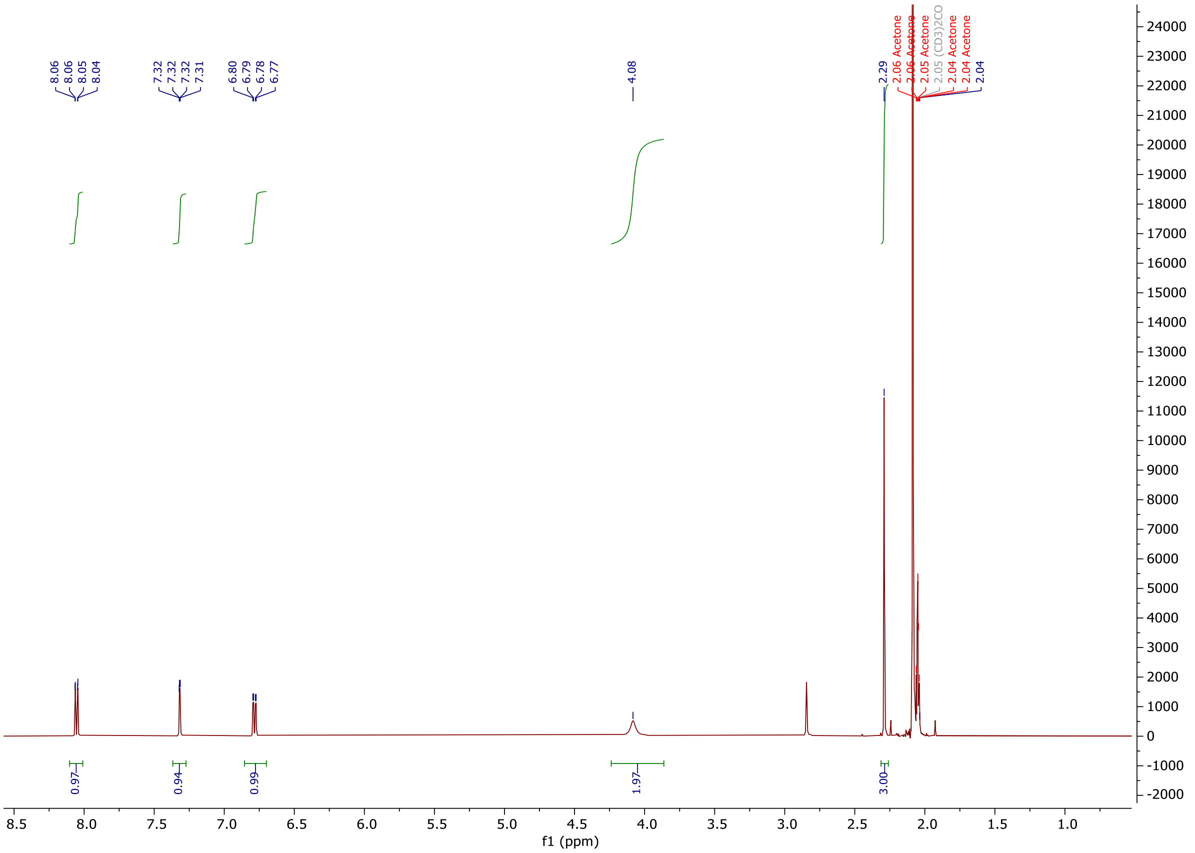

^{13}C spectrum of compound **9:**

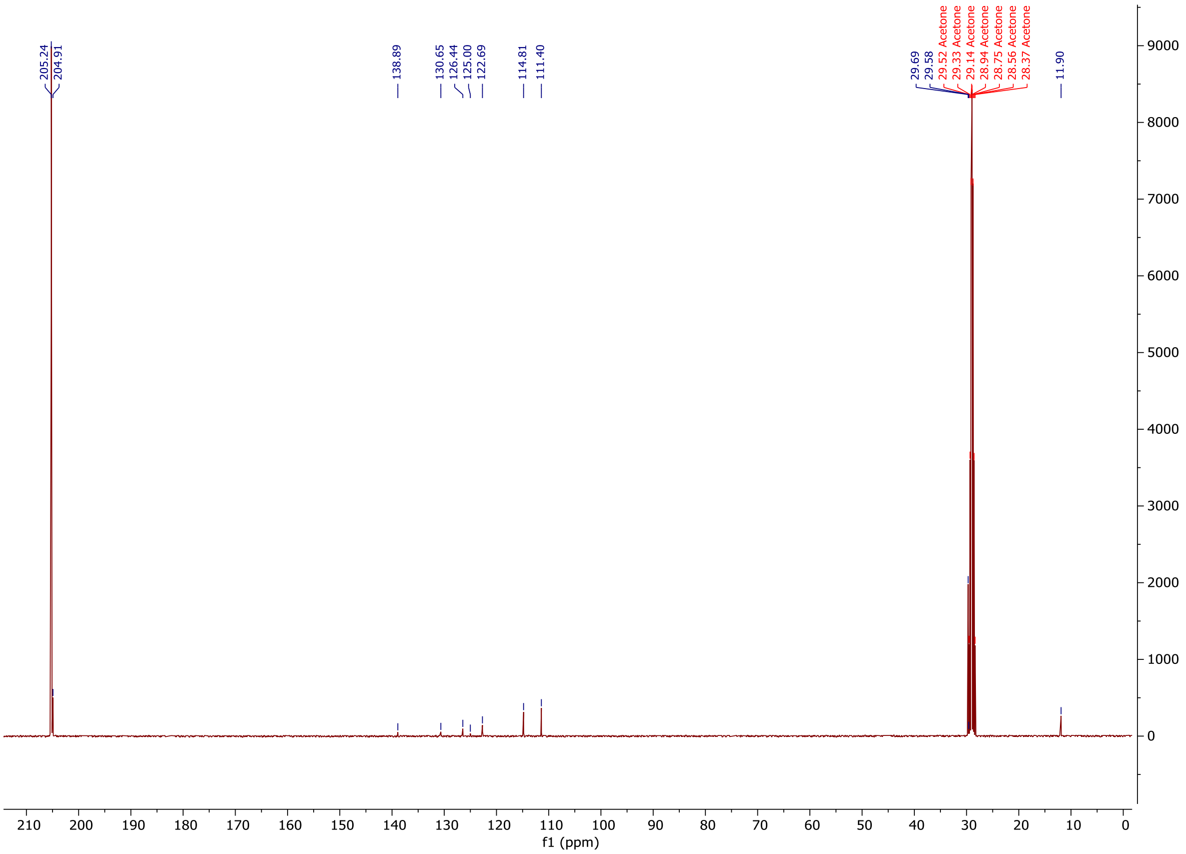

qHNMR spectrum of compound **9:**

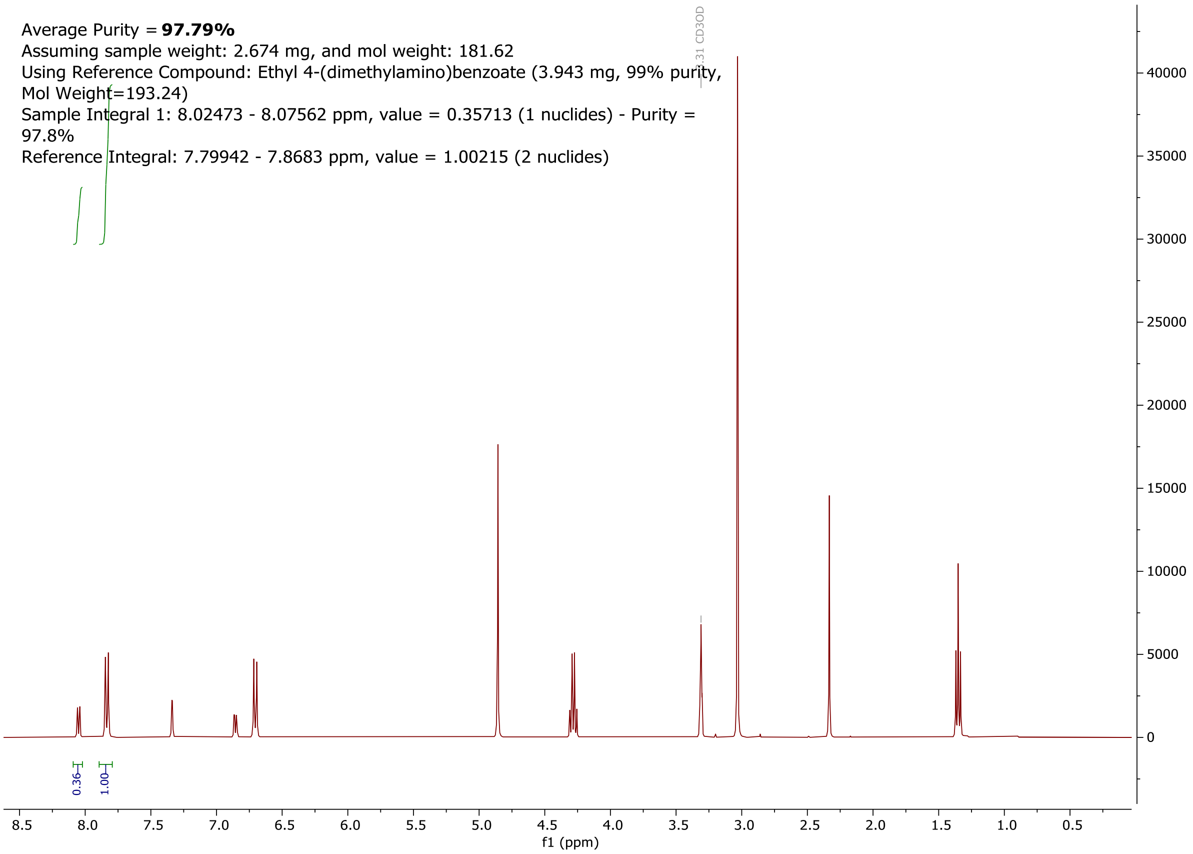

^{1}H spectrum of compound **12:**

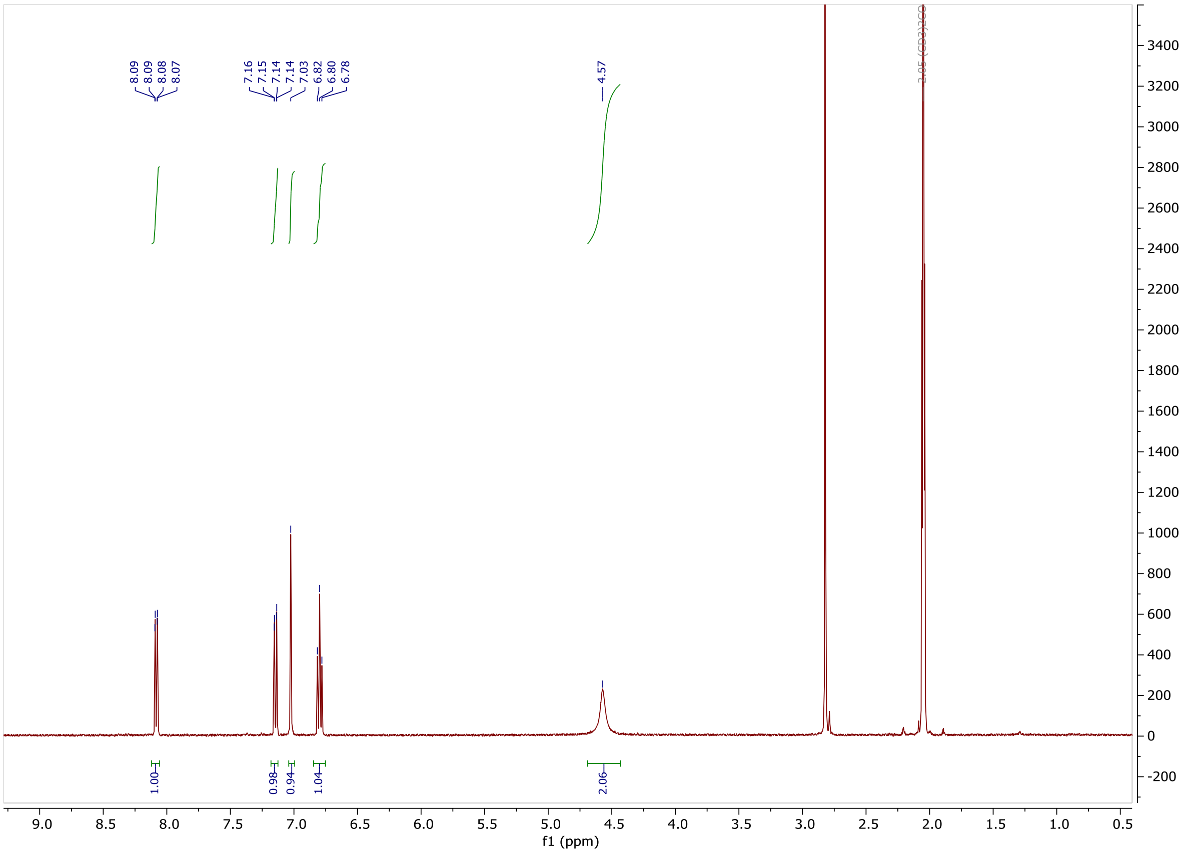

^{13}C spectrum of compound **12:**

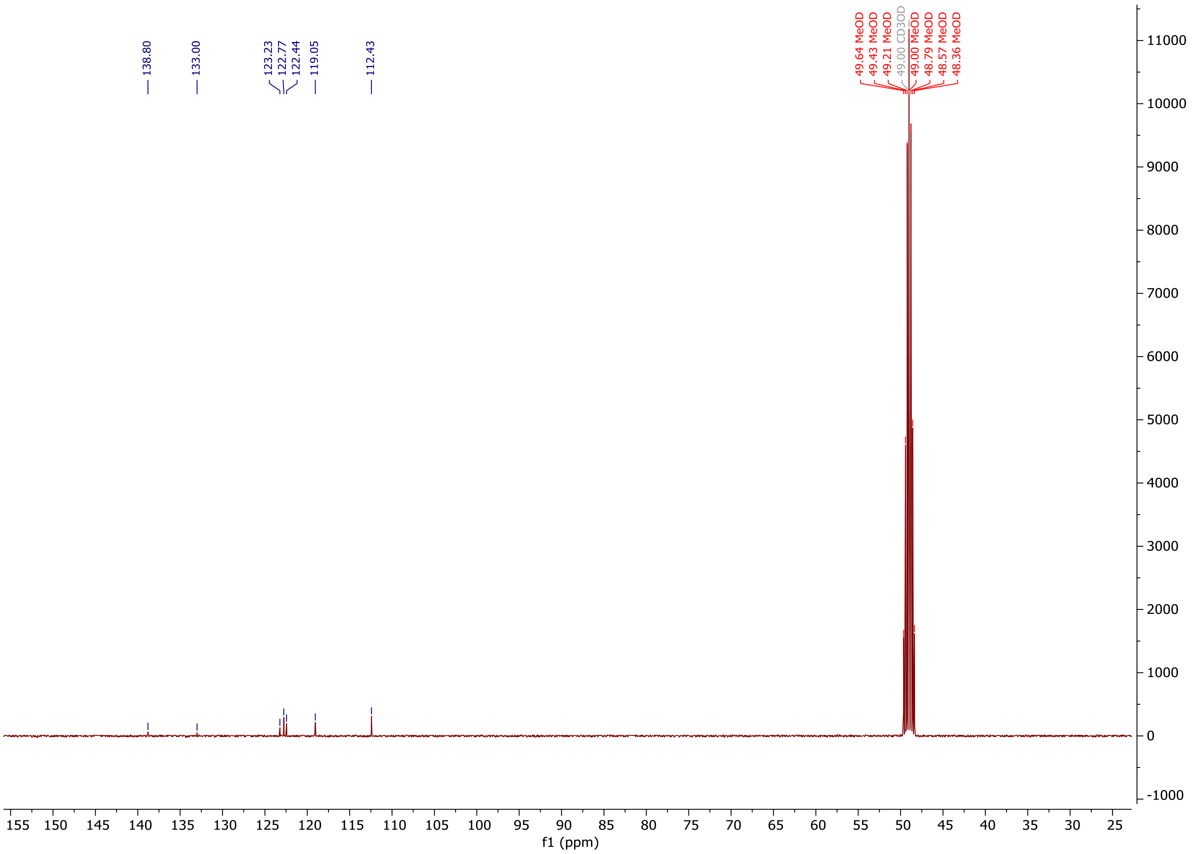

qHNMR spectrum of compound **12:**

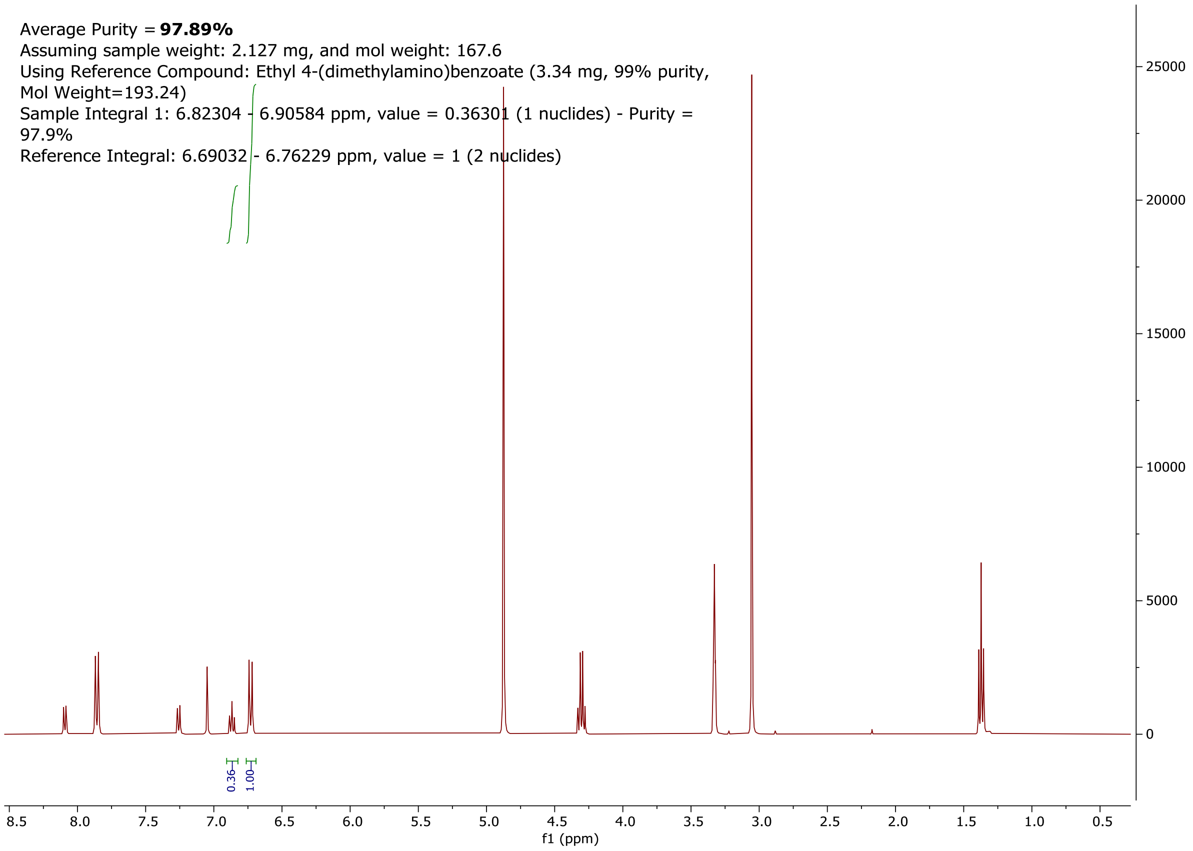

^{1}H spectrum of compound **14:**

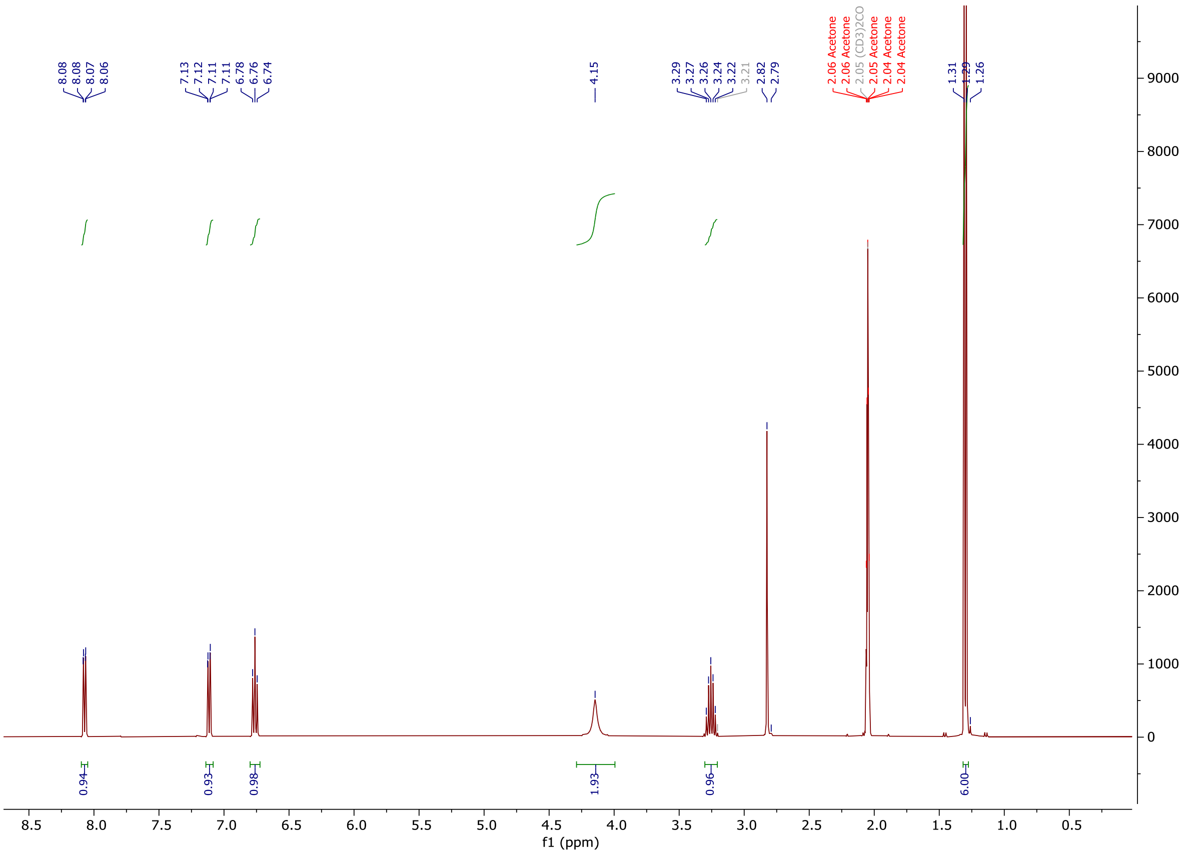

^{13}C spectrum of compound **14:**

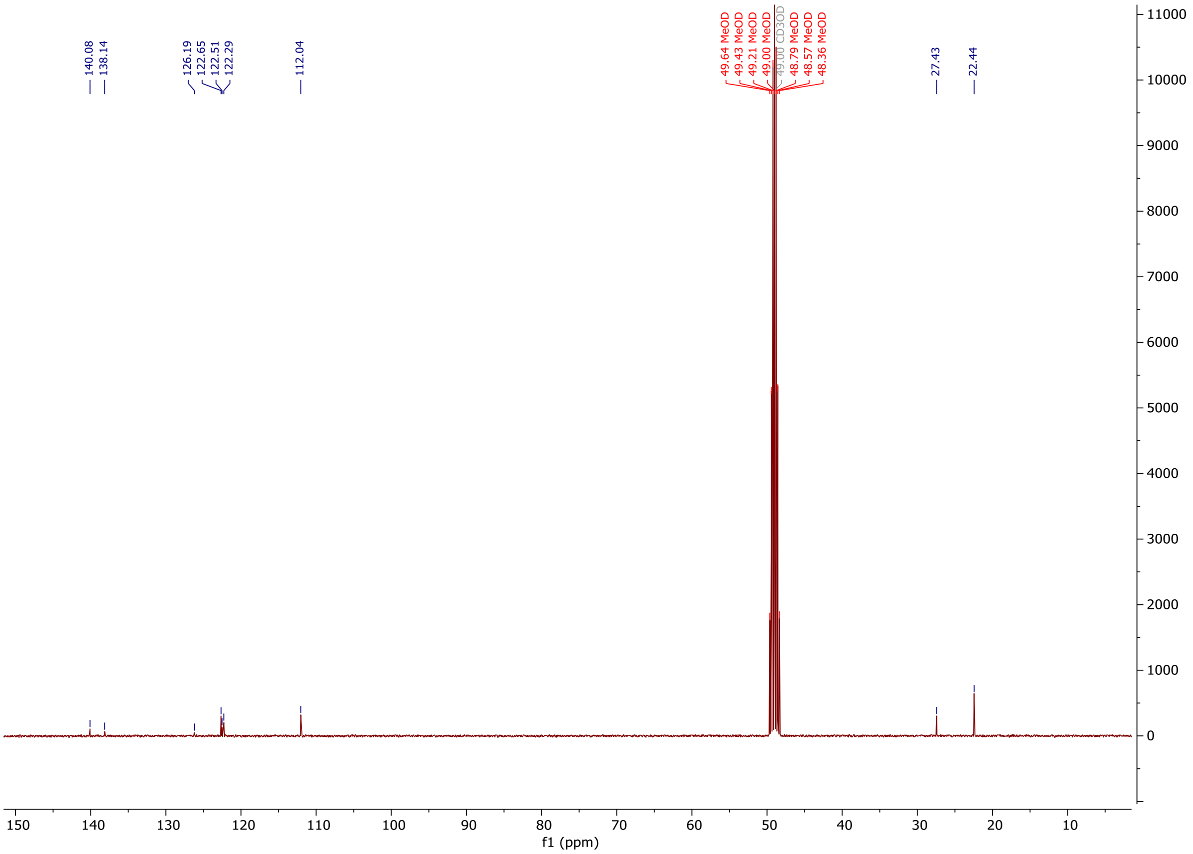

qHNMR spectrum of compound **14:**

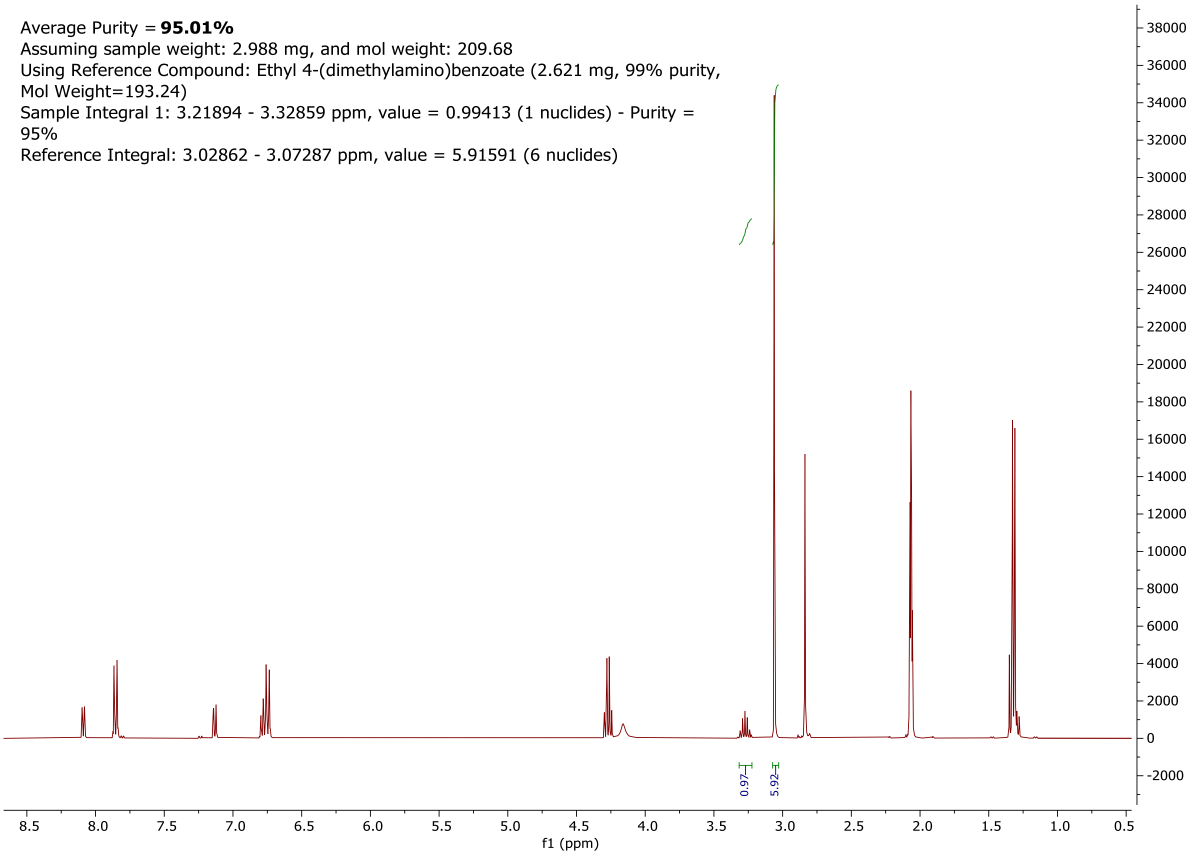

^{1}H spectrum of compound **15:**

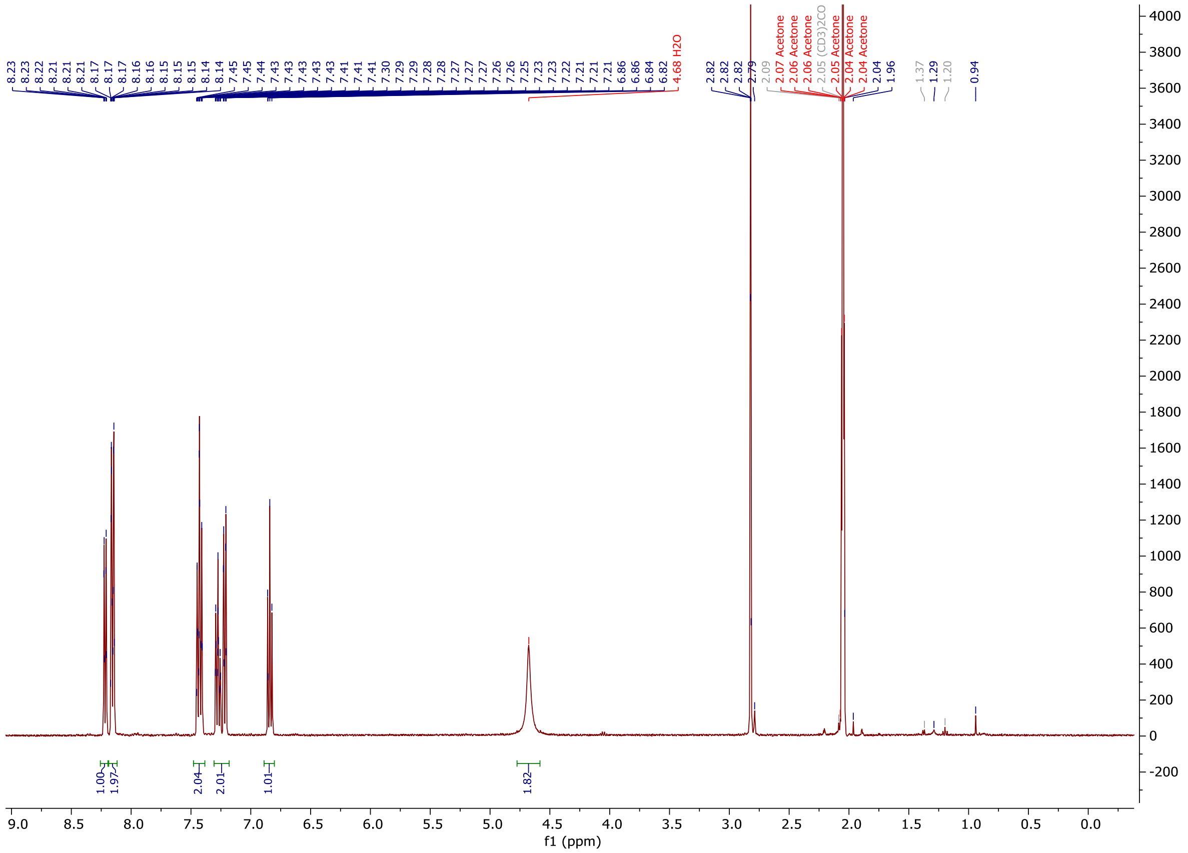

^{13}C spectrum of compound **15:**

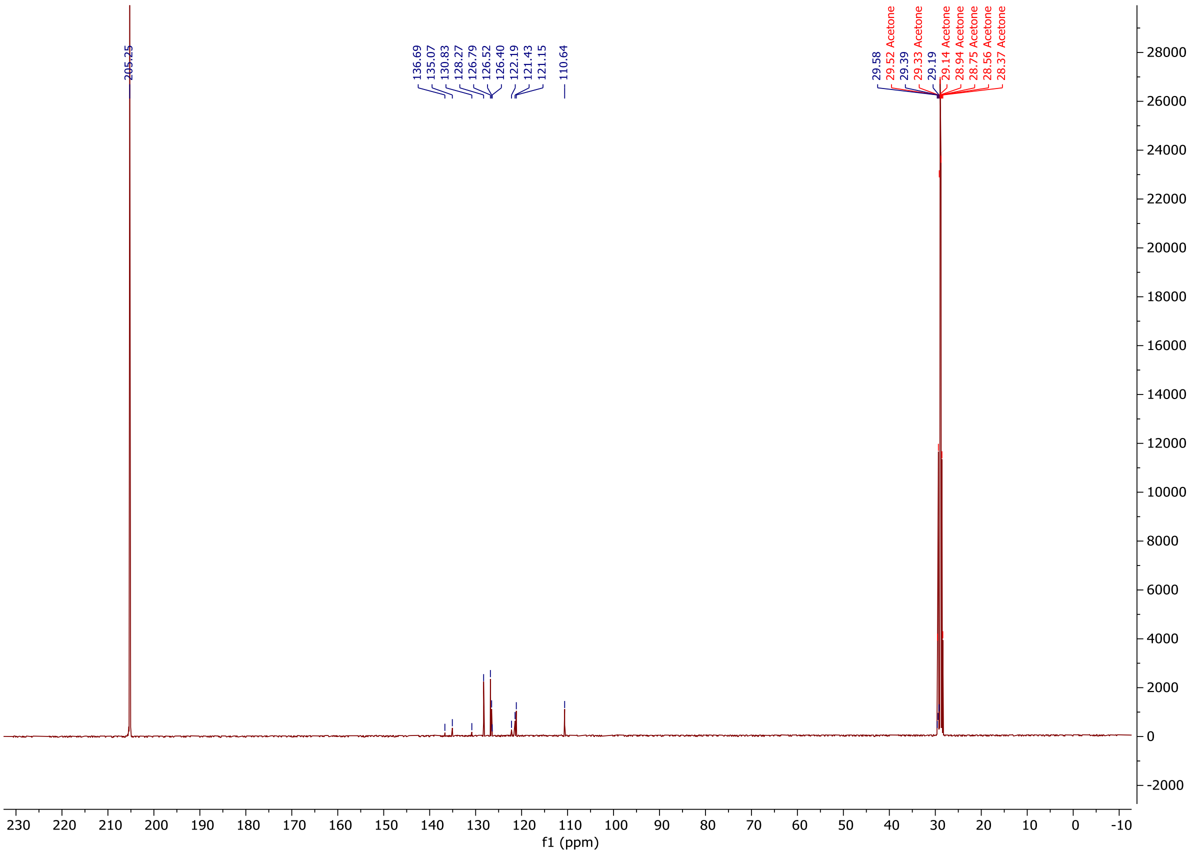

^{13}C spectrum of compound **15:**

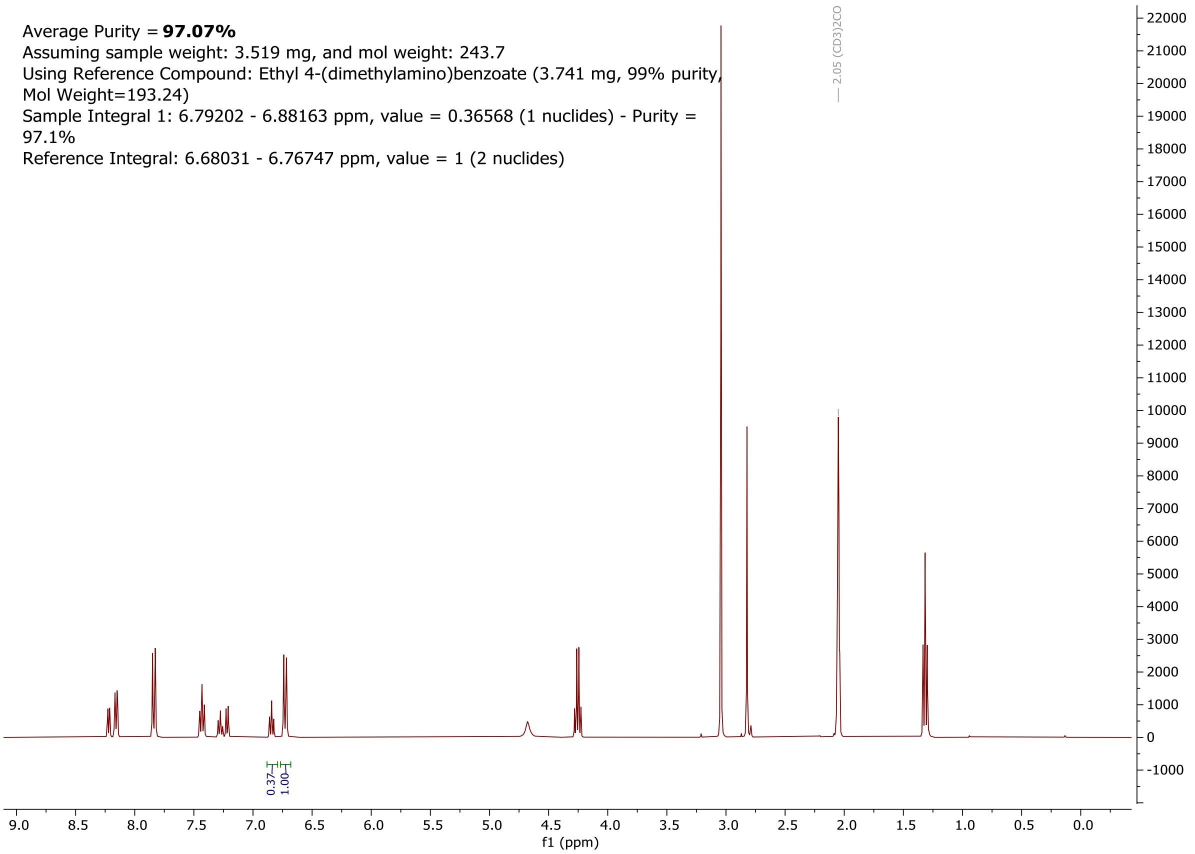

^{1}H spectrum of compound **16:**

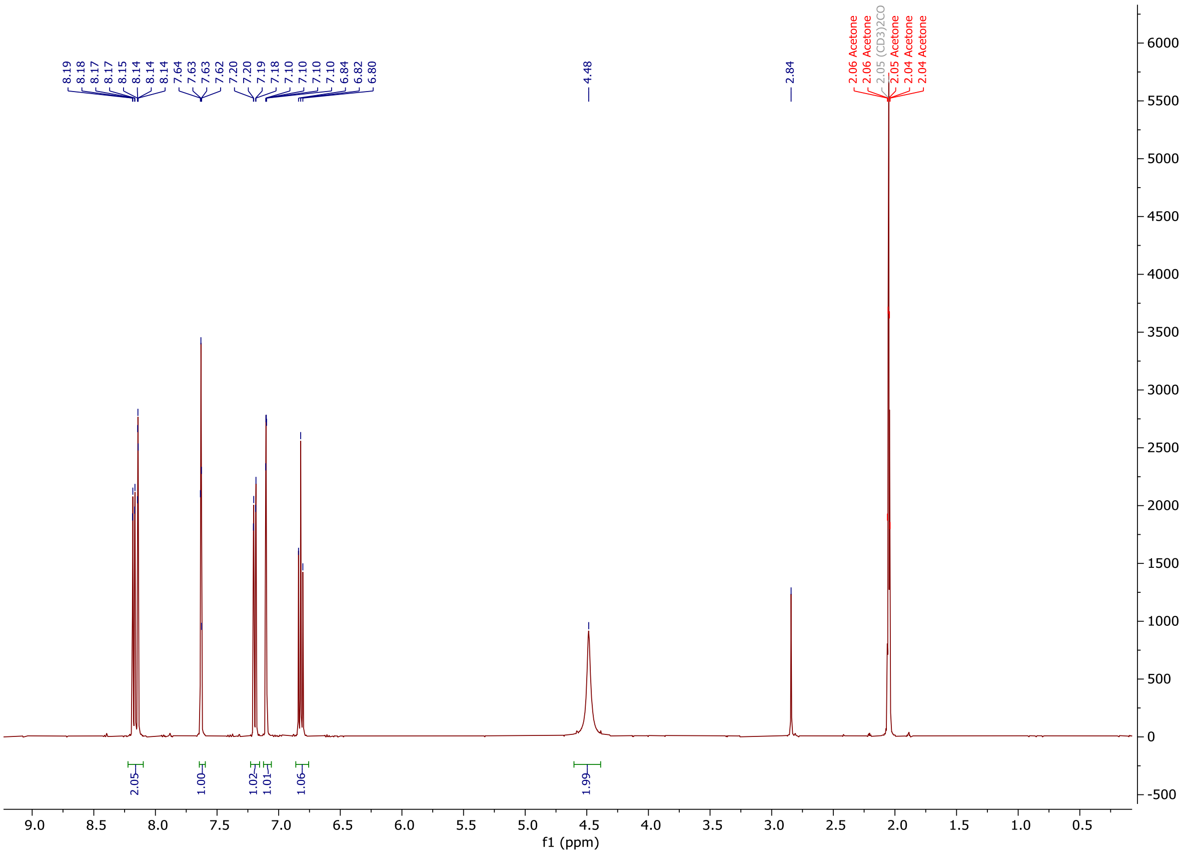

^{13}C spectrum of compound **16:**

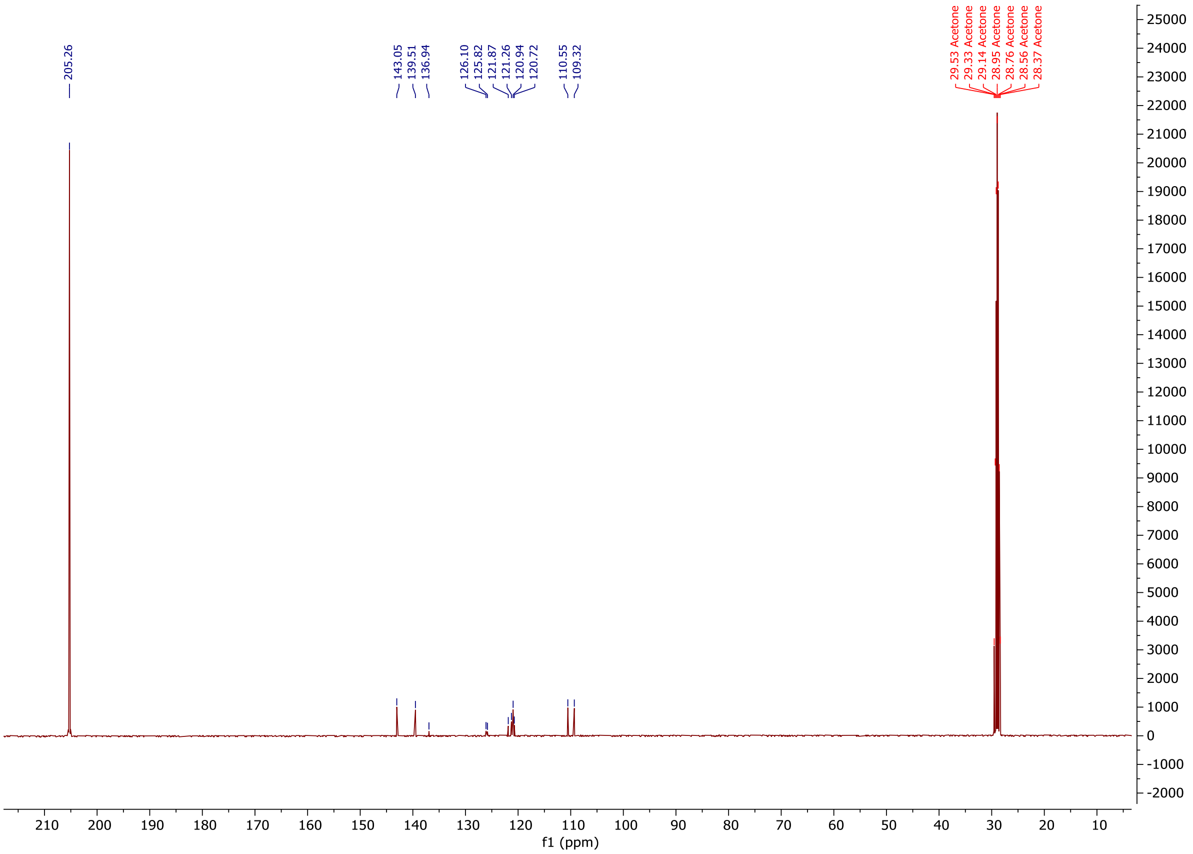

qHNMR spectrum of compound **16:**

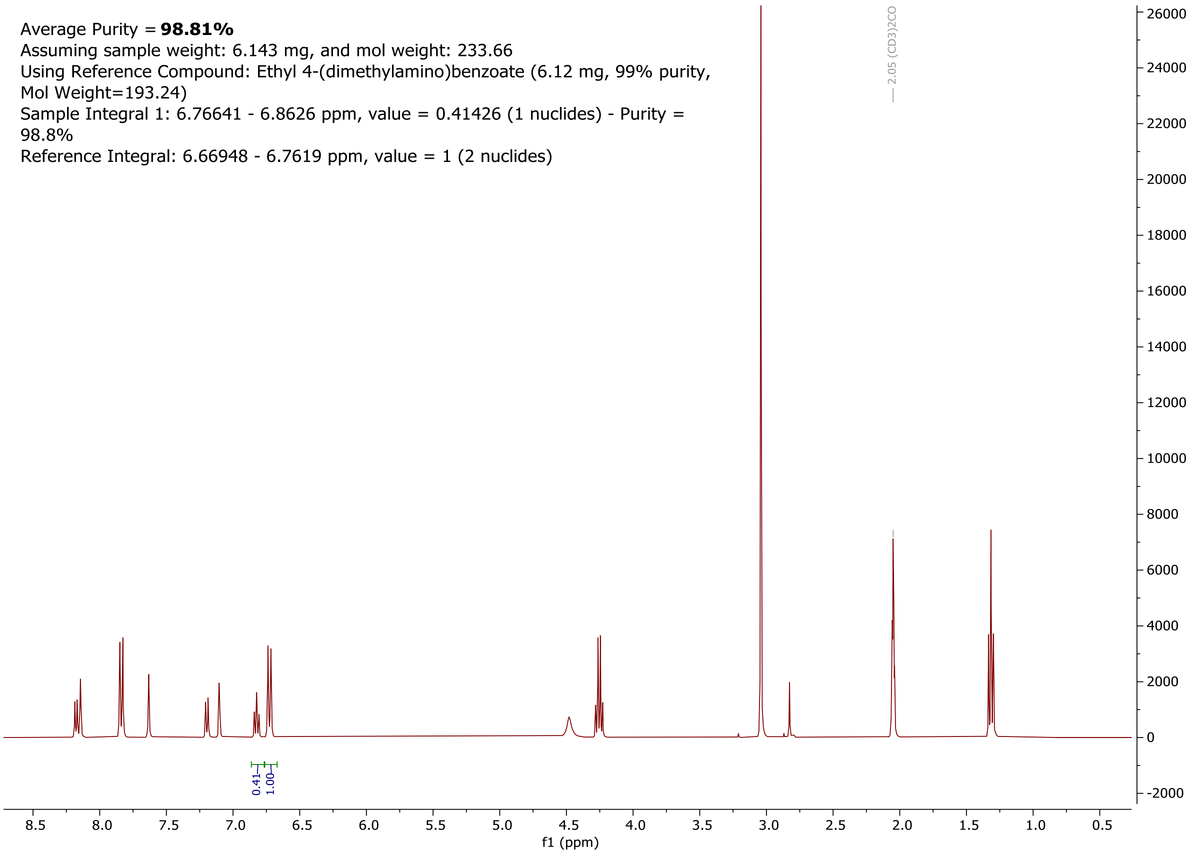

^{1}H spectrum of compound **17:**

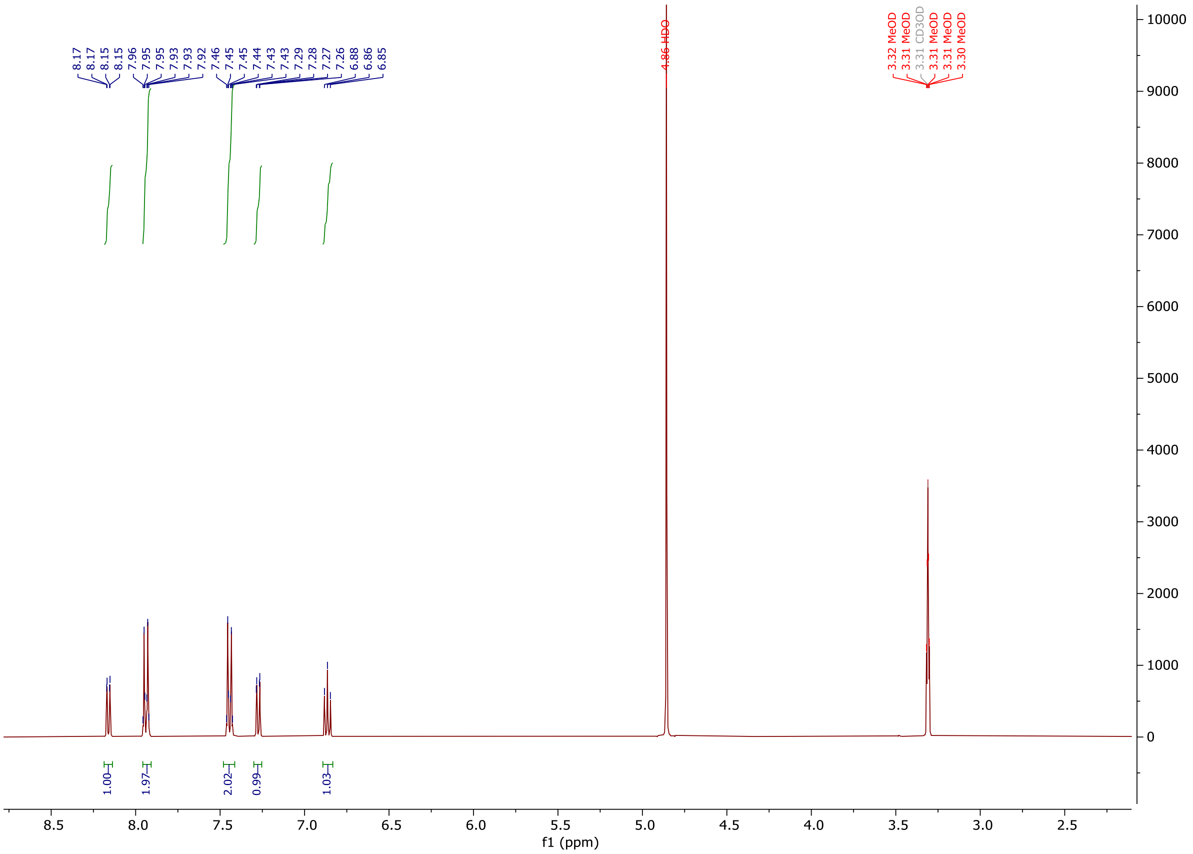

^{13}C spectrum of compound **17:**

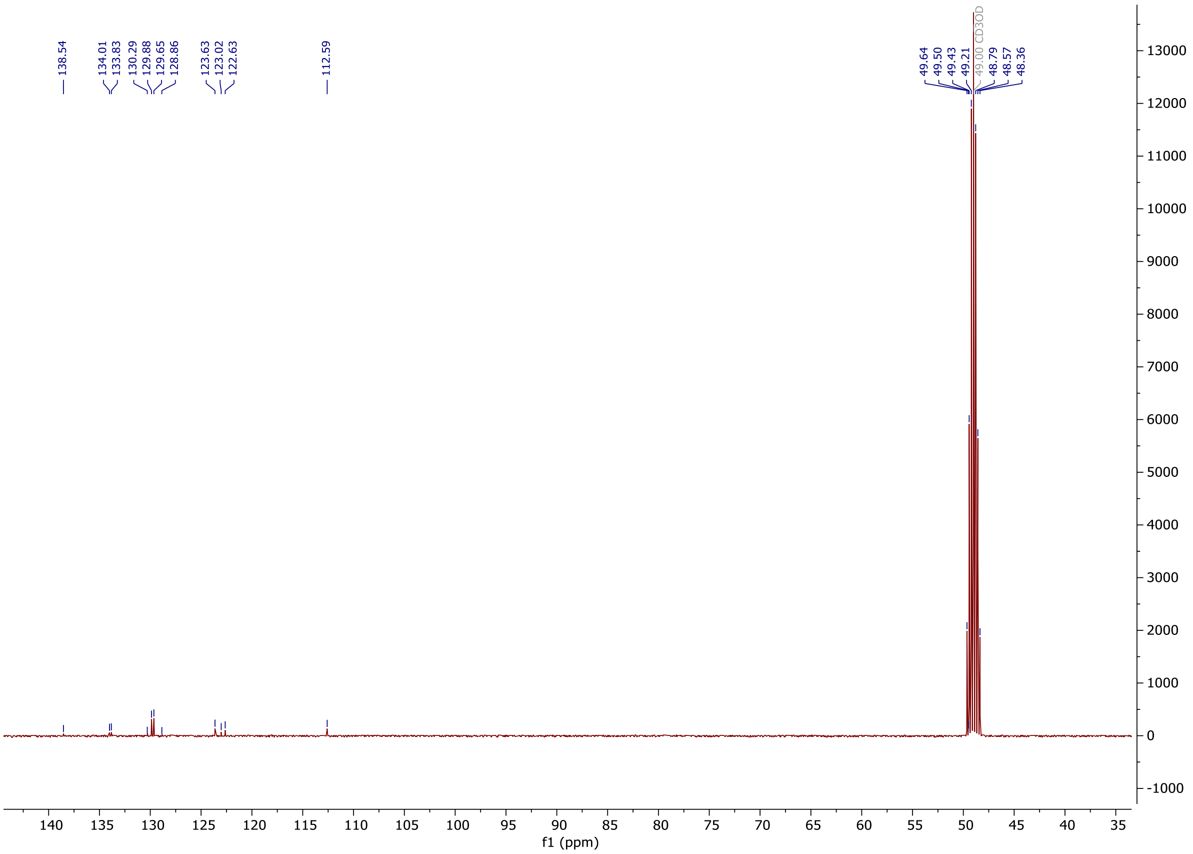

qHNMR spectrum of compound **17:**

Average Purity = **95.66%**
Assuming sample weight: 1.395 mg, and mol weight: 278.14
Using Reference Compound: Ethyl 4-(dimethylamino)benzoate (1.71 mg, 99% purity, Mol Weight=193.24)
Sample Integral 1: 7.36714 - 7.49978 ppm, value = 0.54764 (1 nuclides) - Purity = 95.7%
Reference Integral: 6.63845 - 6.77264 ppm, value = 1 (1 nuclides)

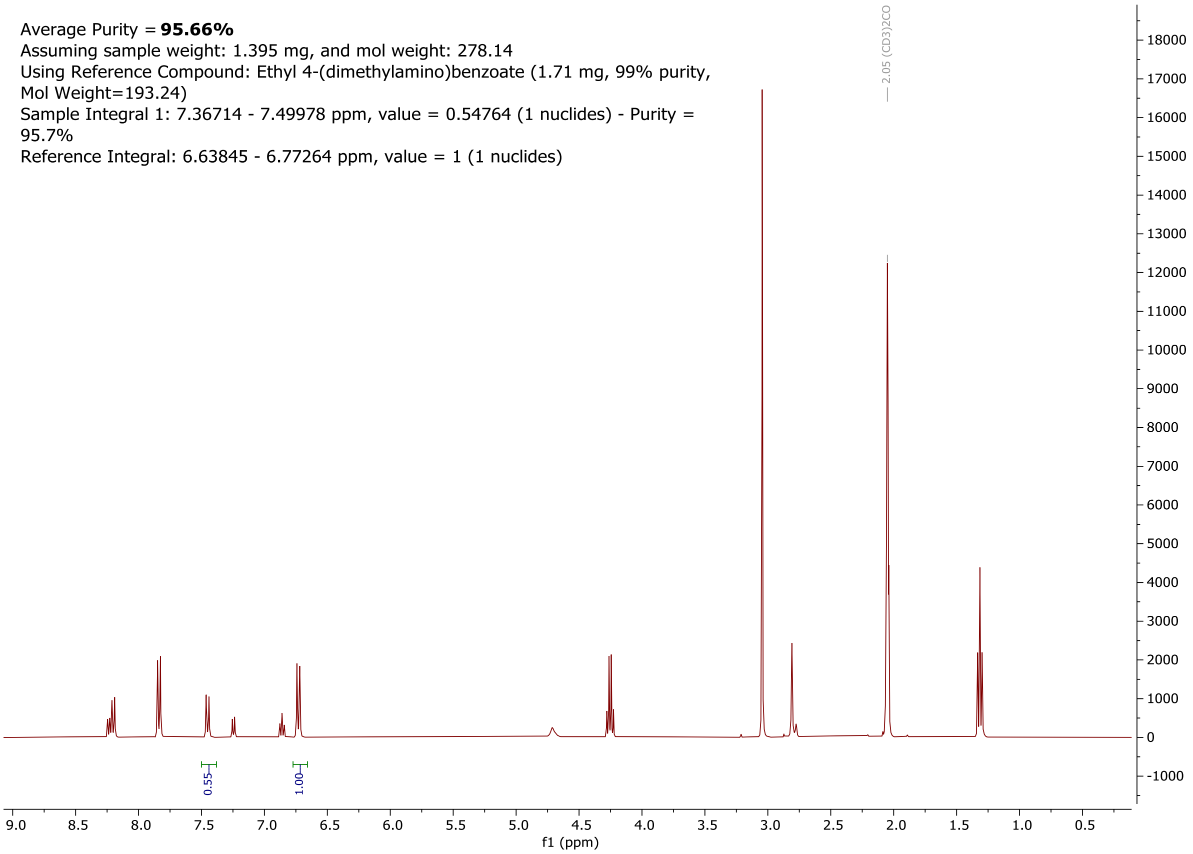

^{1}H spectrum of compound **18:**

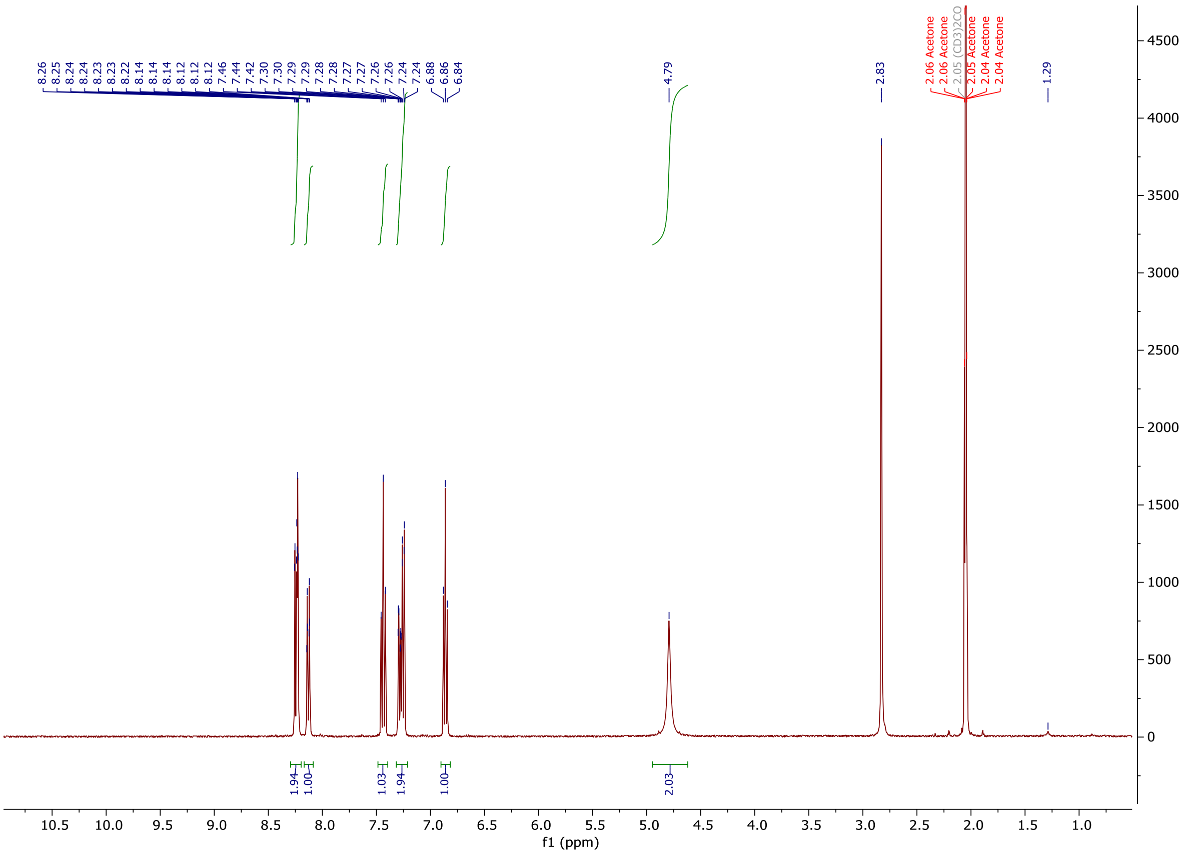

^{13}C spectrum of compound **18:**

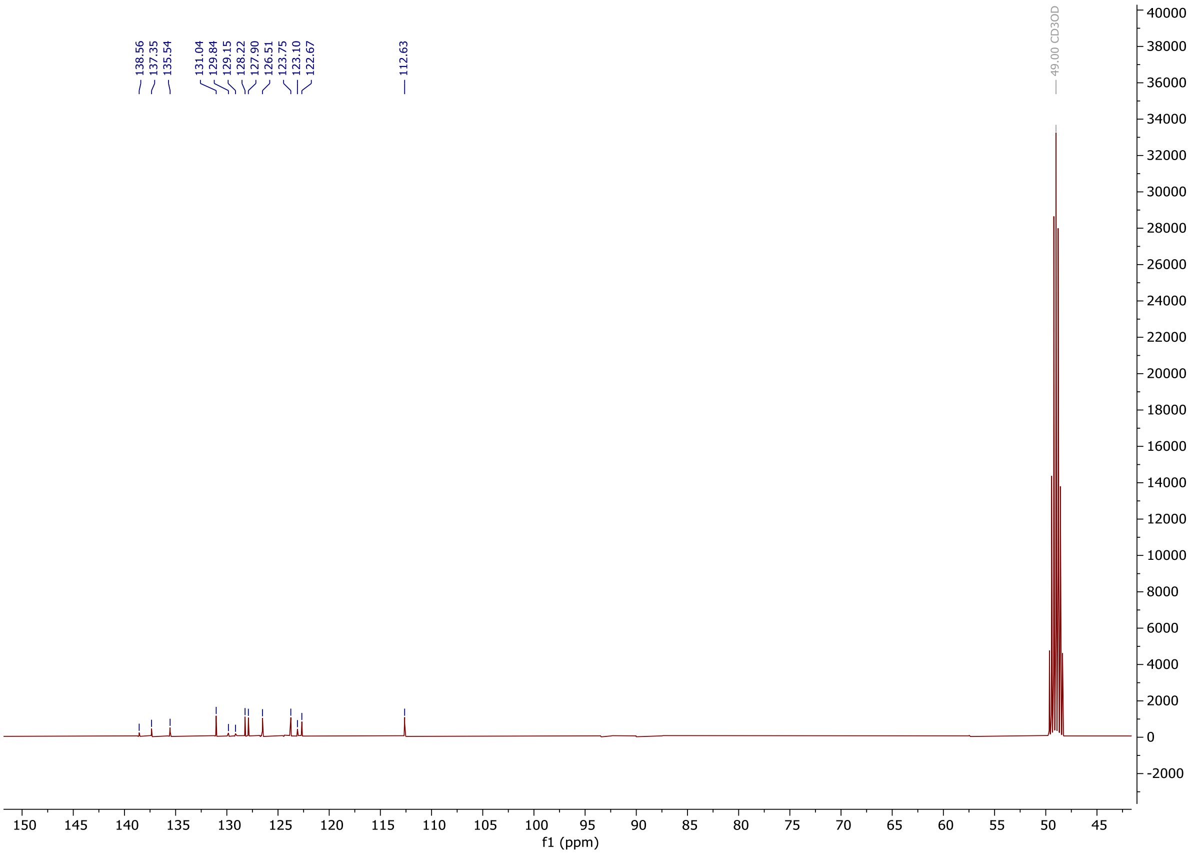

qHNMR spectrum of compound **18:**

Average Purity = **97.16%**
Assuming sample weight: 3.797 mg, and mol weight: 278.14
Using Reference Compound: Ethyl 4-(dimethylamino)benzoate (3.221 mg, 99% purity, Mol Weight=193.24)
Sample Integral 1: 7.40174 - 7.47438 ppm, value = 0.4019 (1 nuclides) - Purity = 97.2%
Reference Integral: 7.79609 - 7.86873 ppm, value = 1 (2 nuclides)

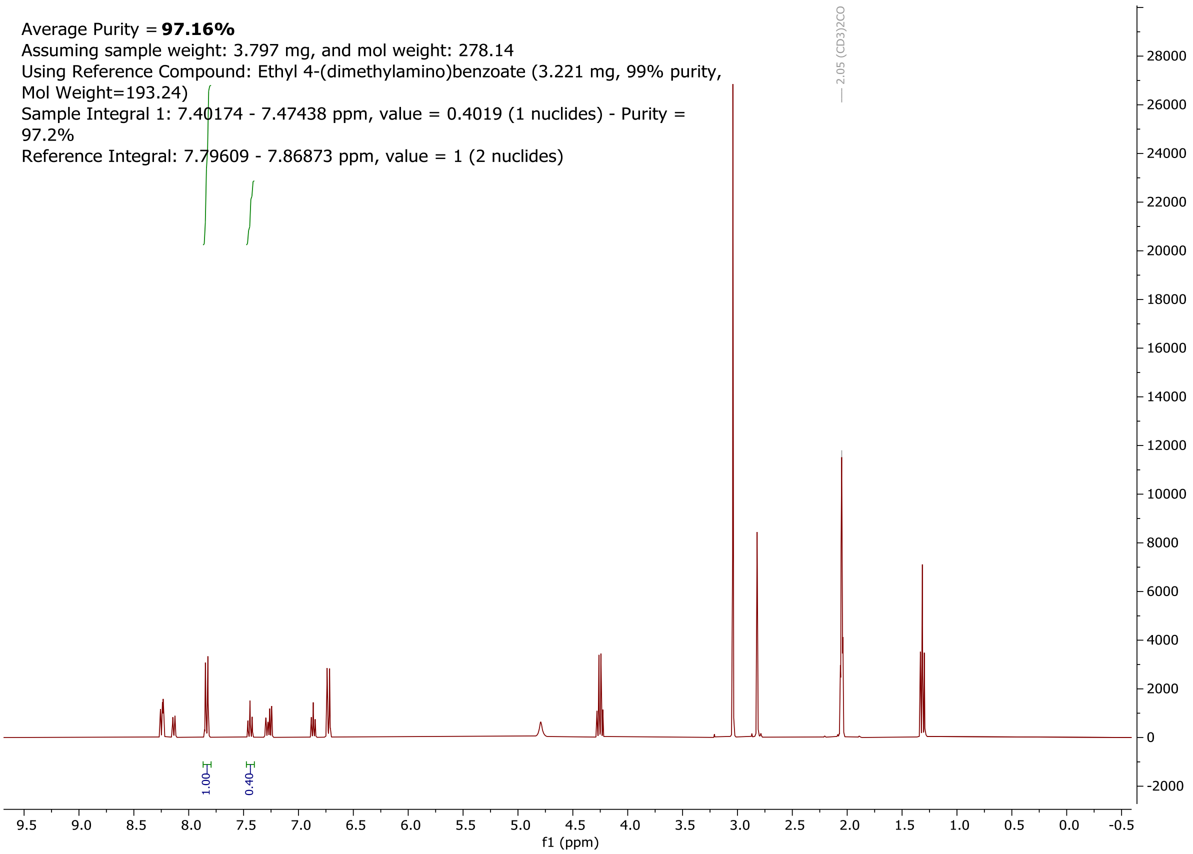

^{1}H spectrum of compound **19:**

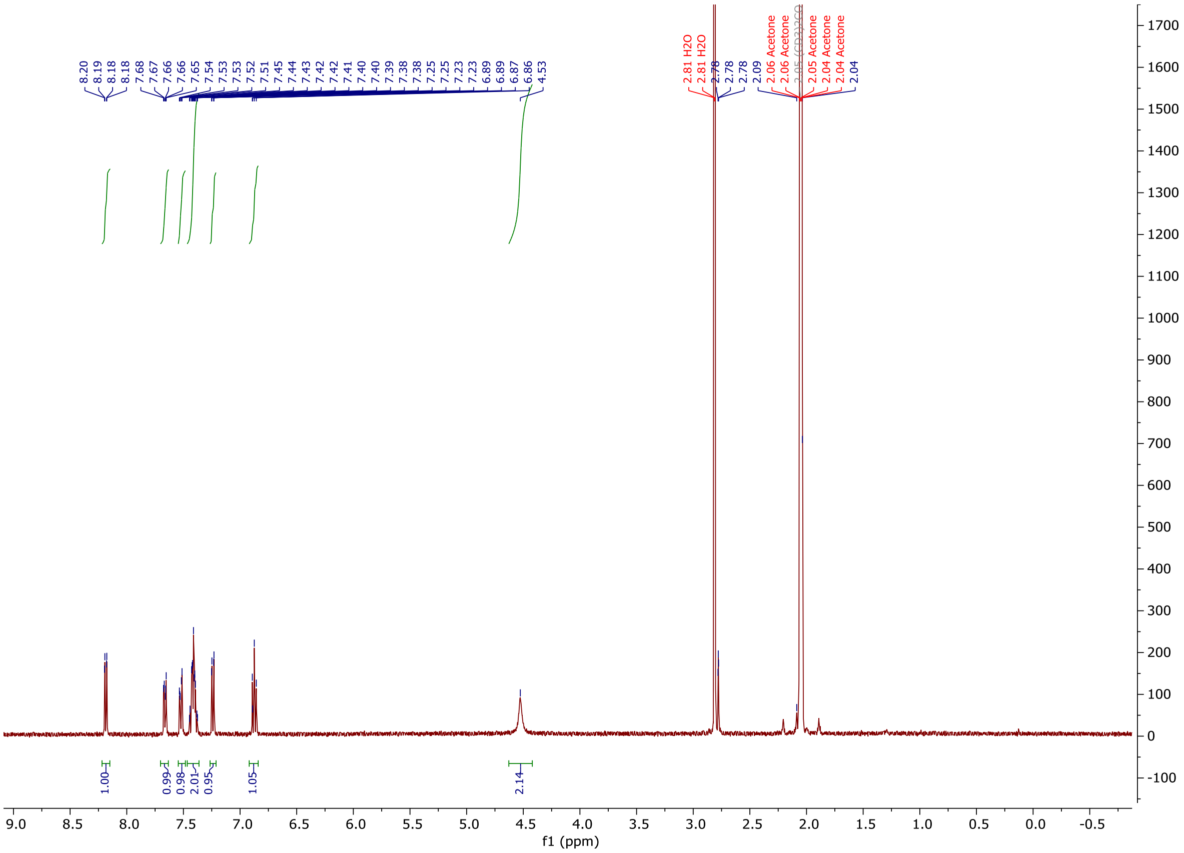

^{13}C spectrum of compound **19:**

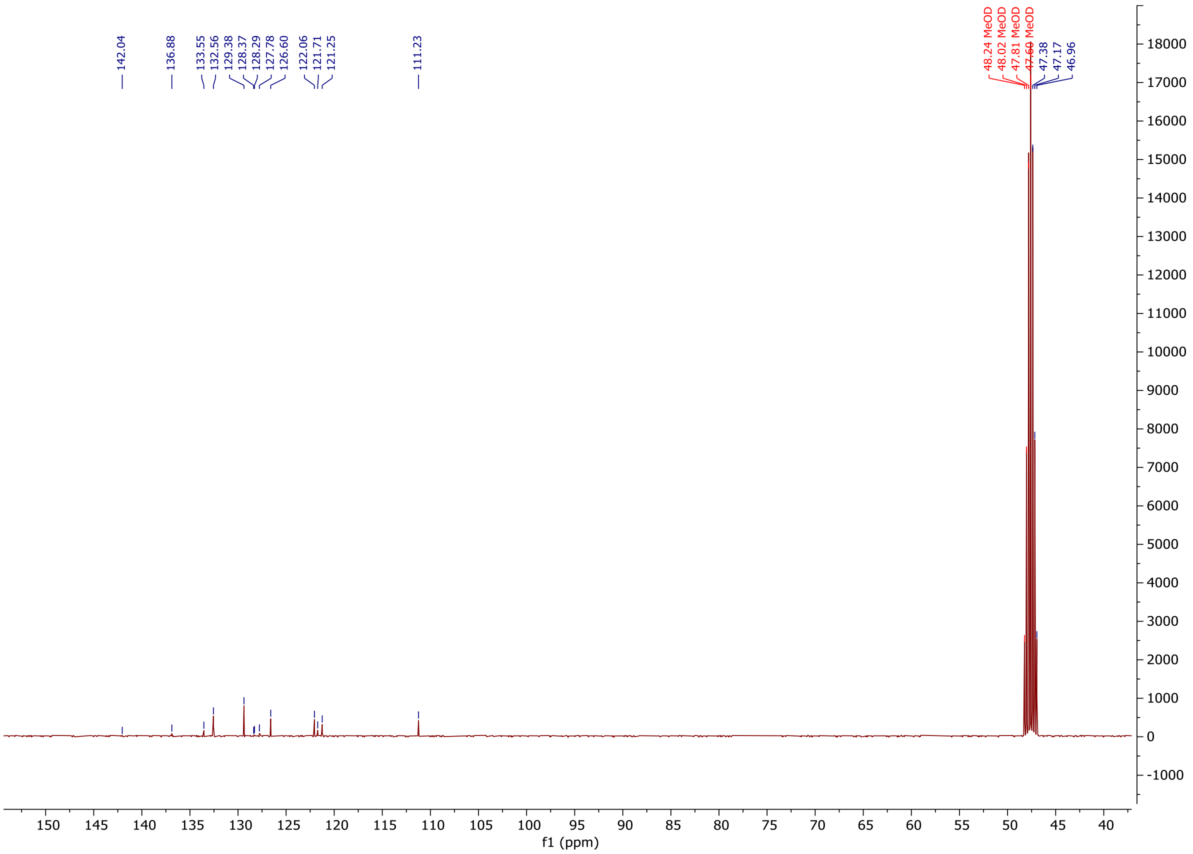

qHNMR spectrum of compound **19:**

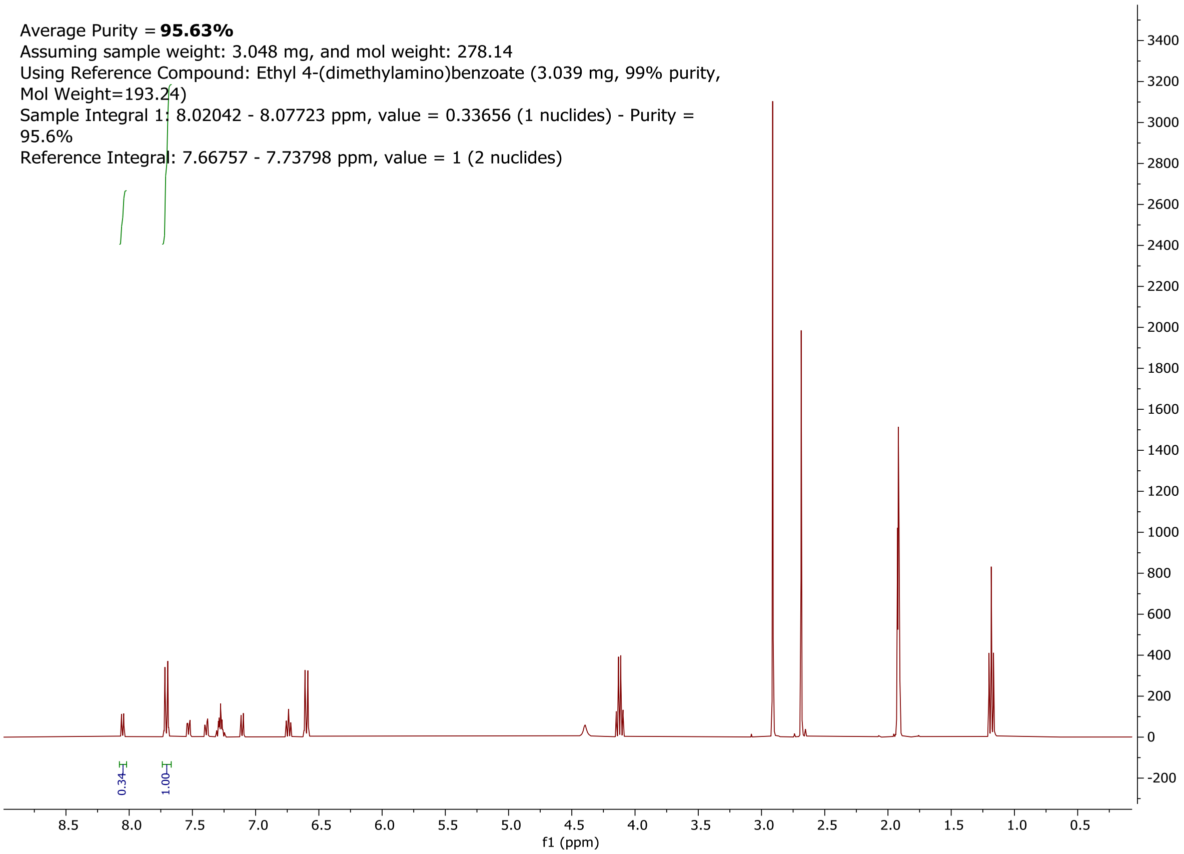

^{1}H spectrum of compound **20:**

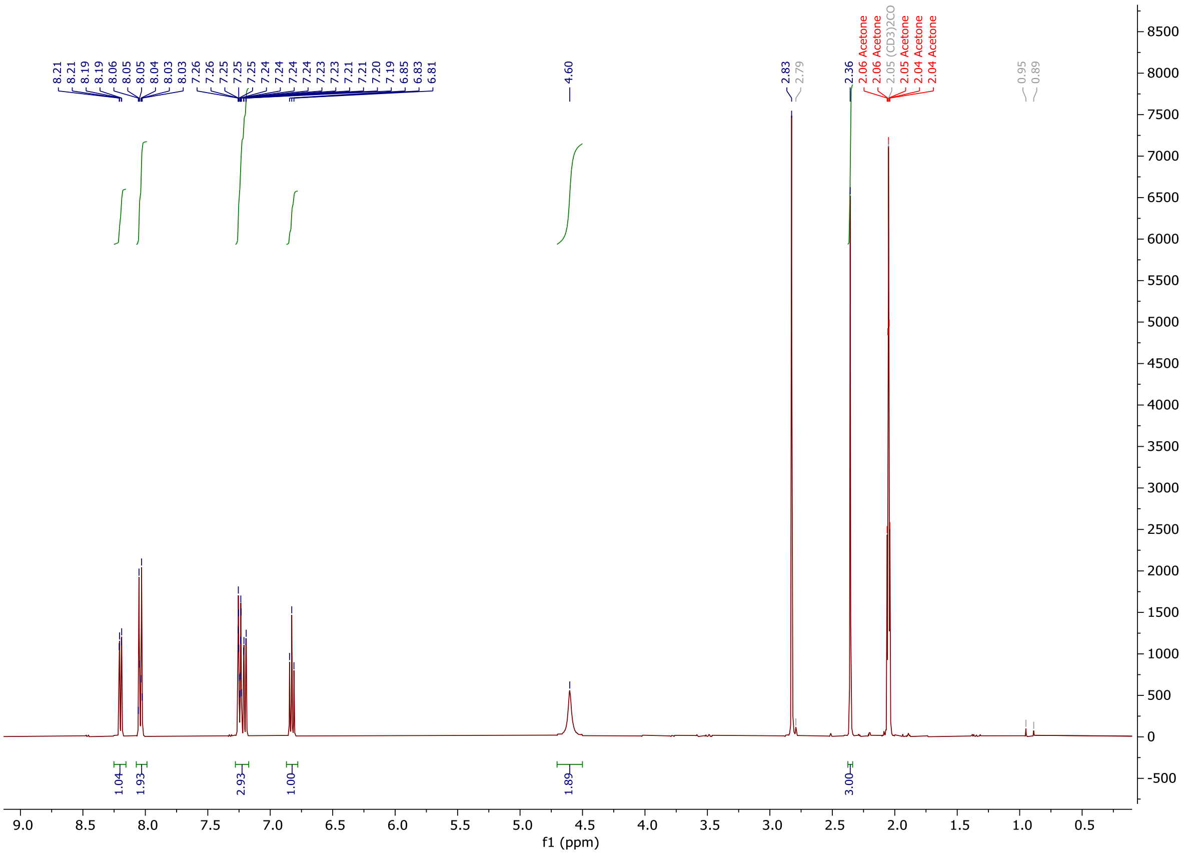

^{13}C spectrum of compound **20:**

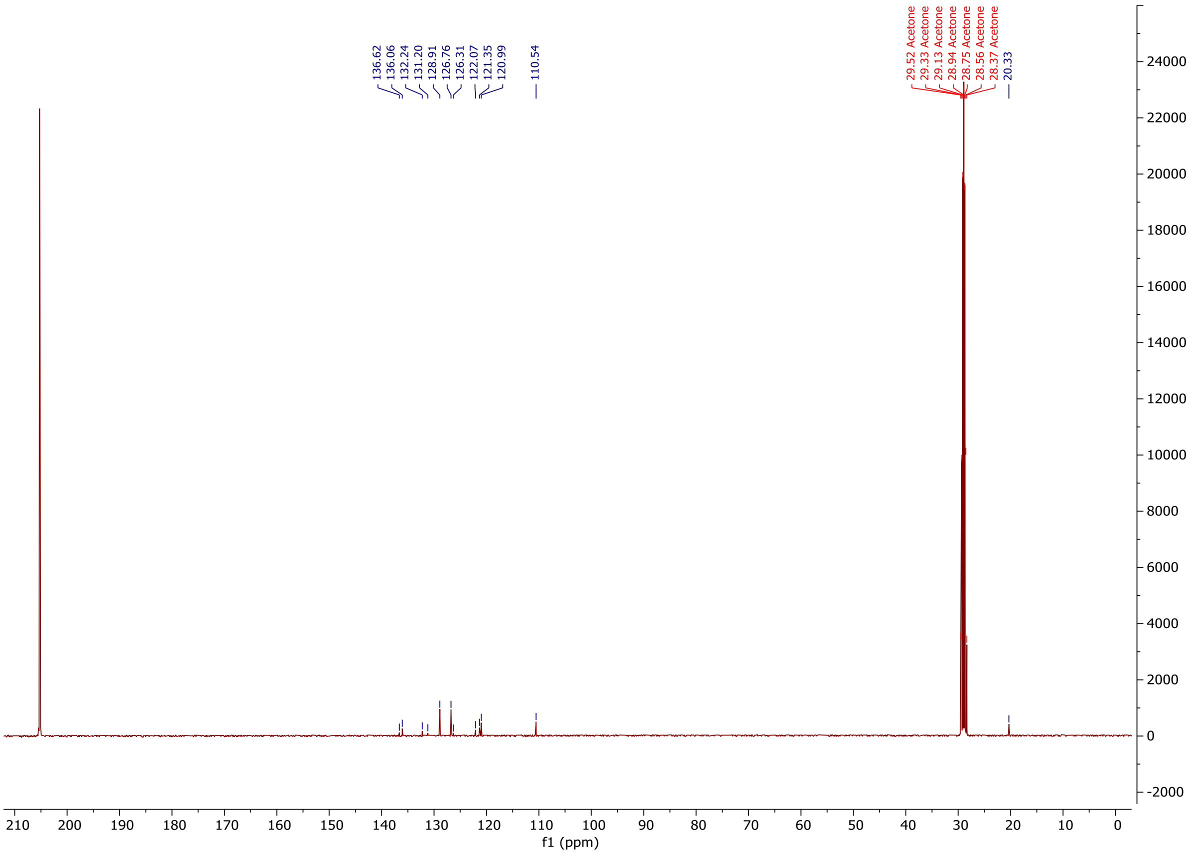

qHNMR spectrum of compound **20:**

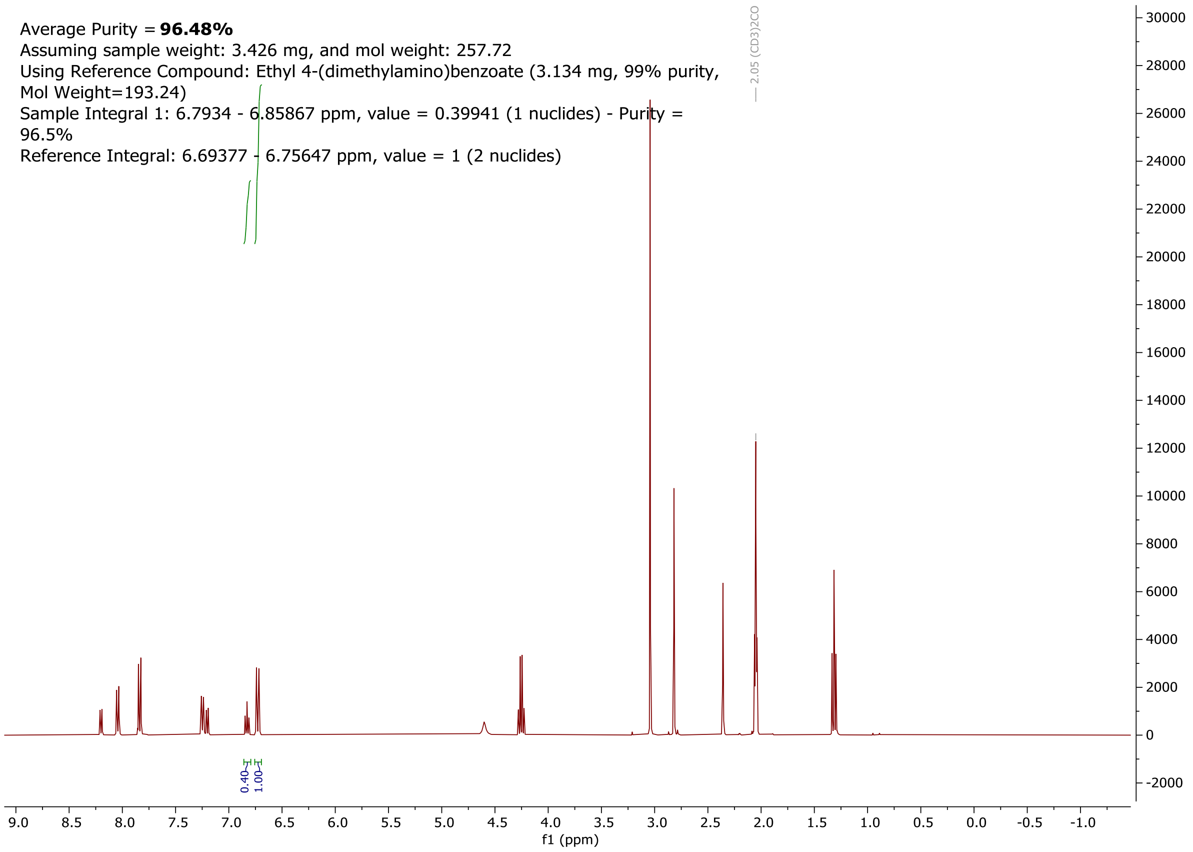

^{1}H spectrum of compound **21:**

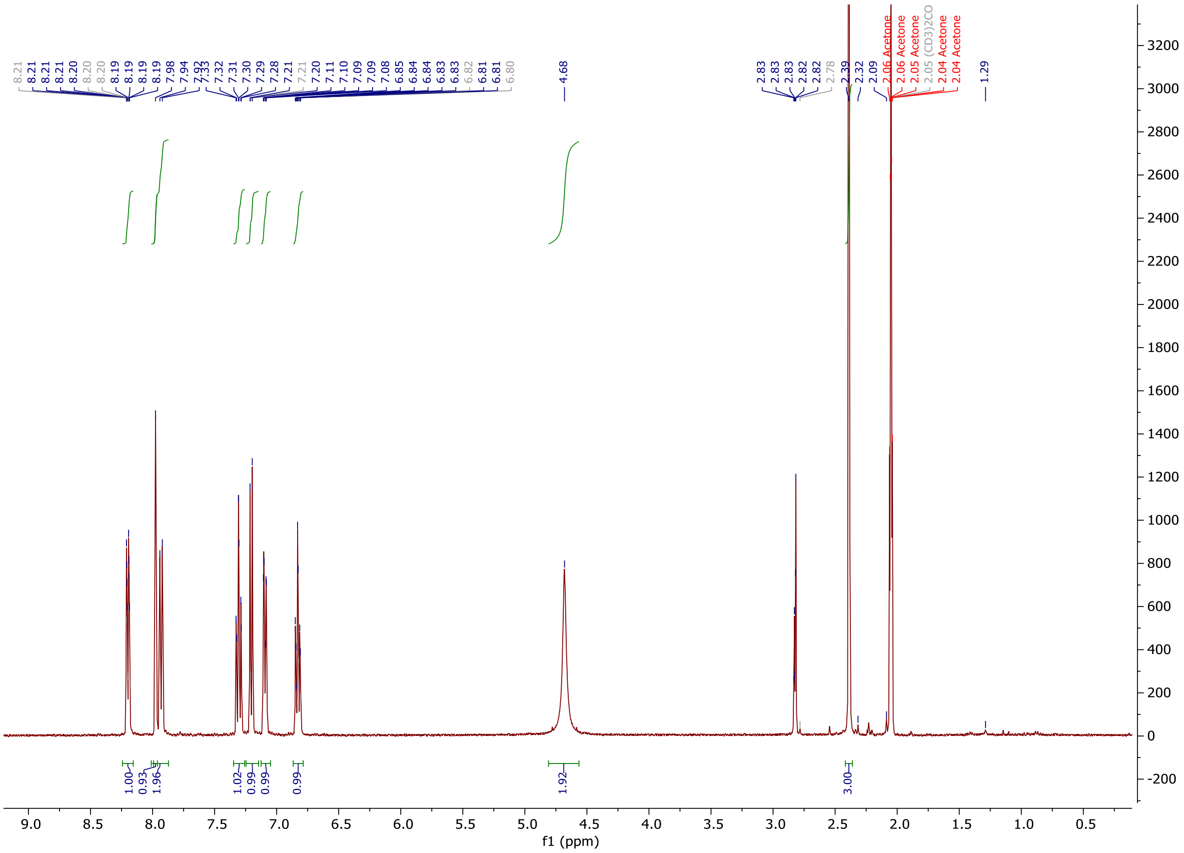

^{13}C spectrum of compound **21:**

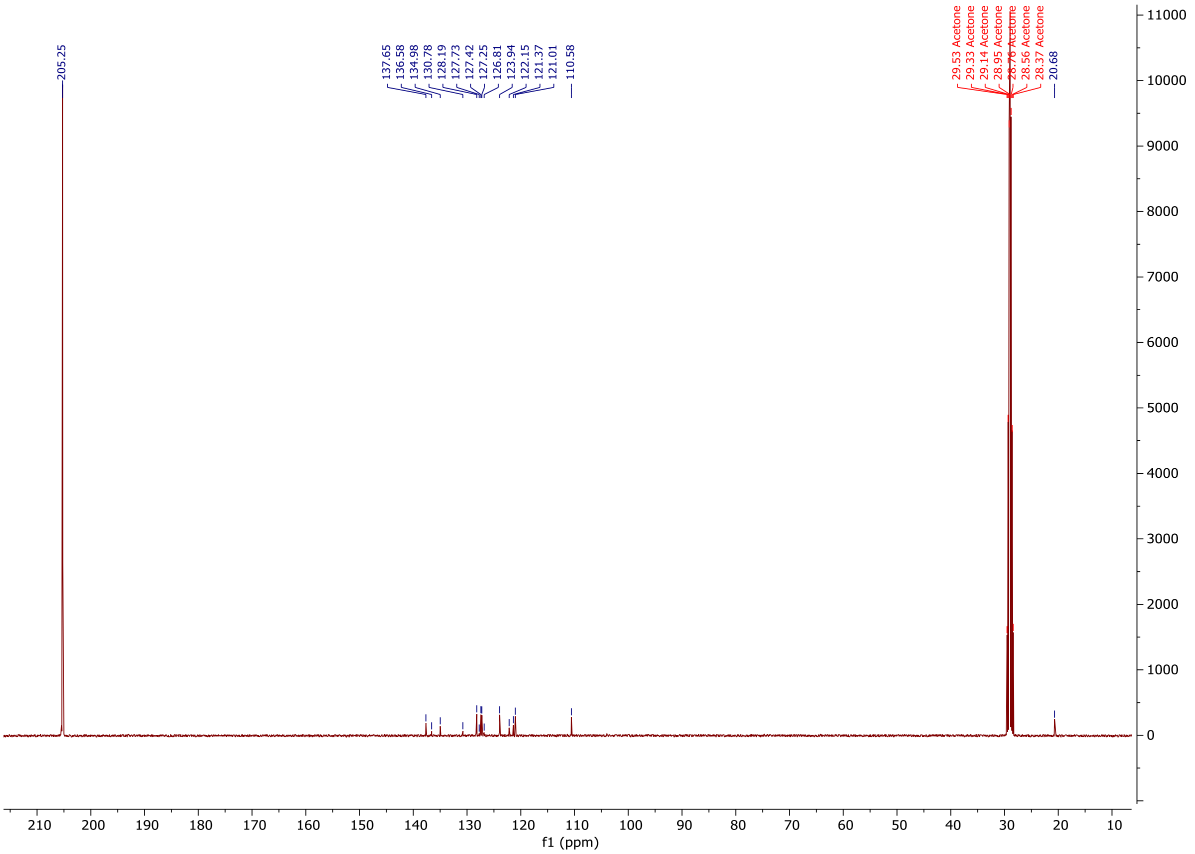

qHNMR spectrum of compound **21:**

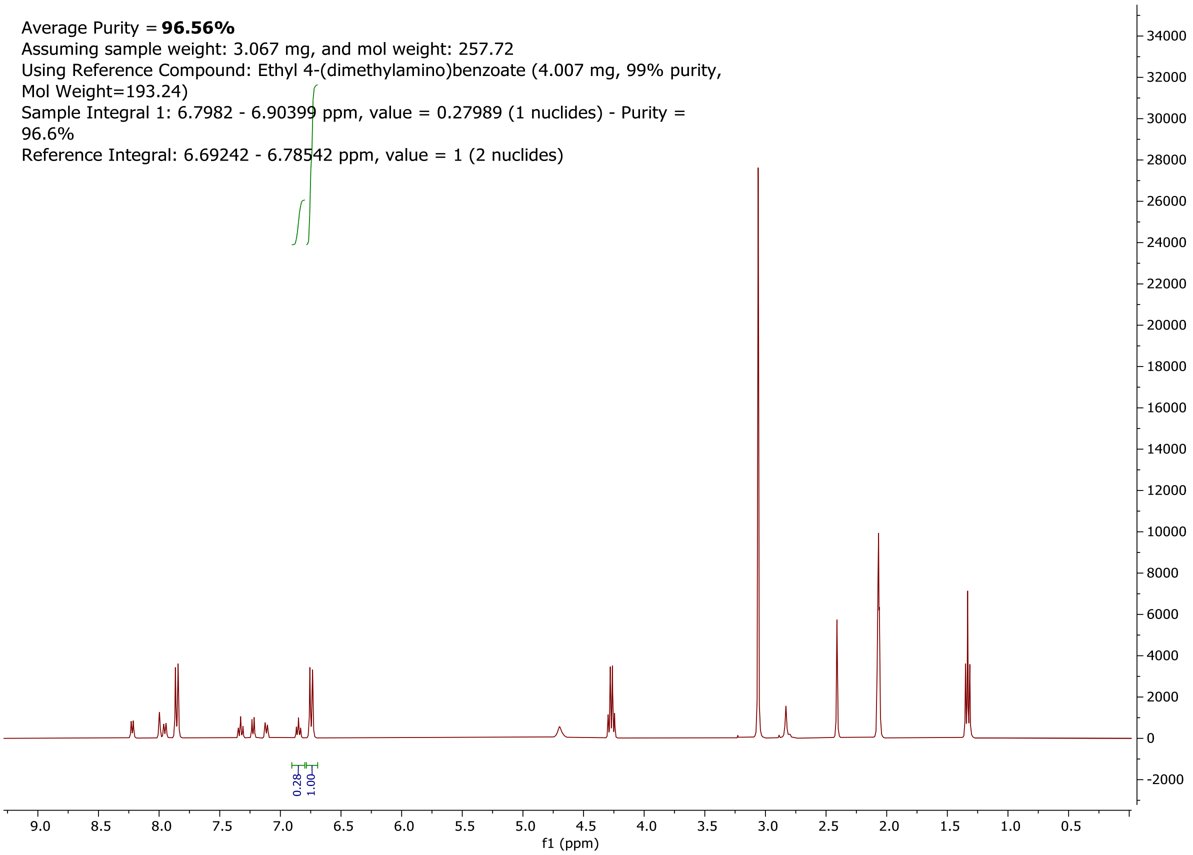

^{1}H spectrum of compound **22:**

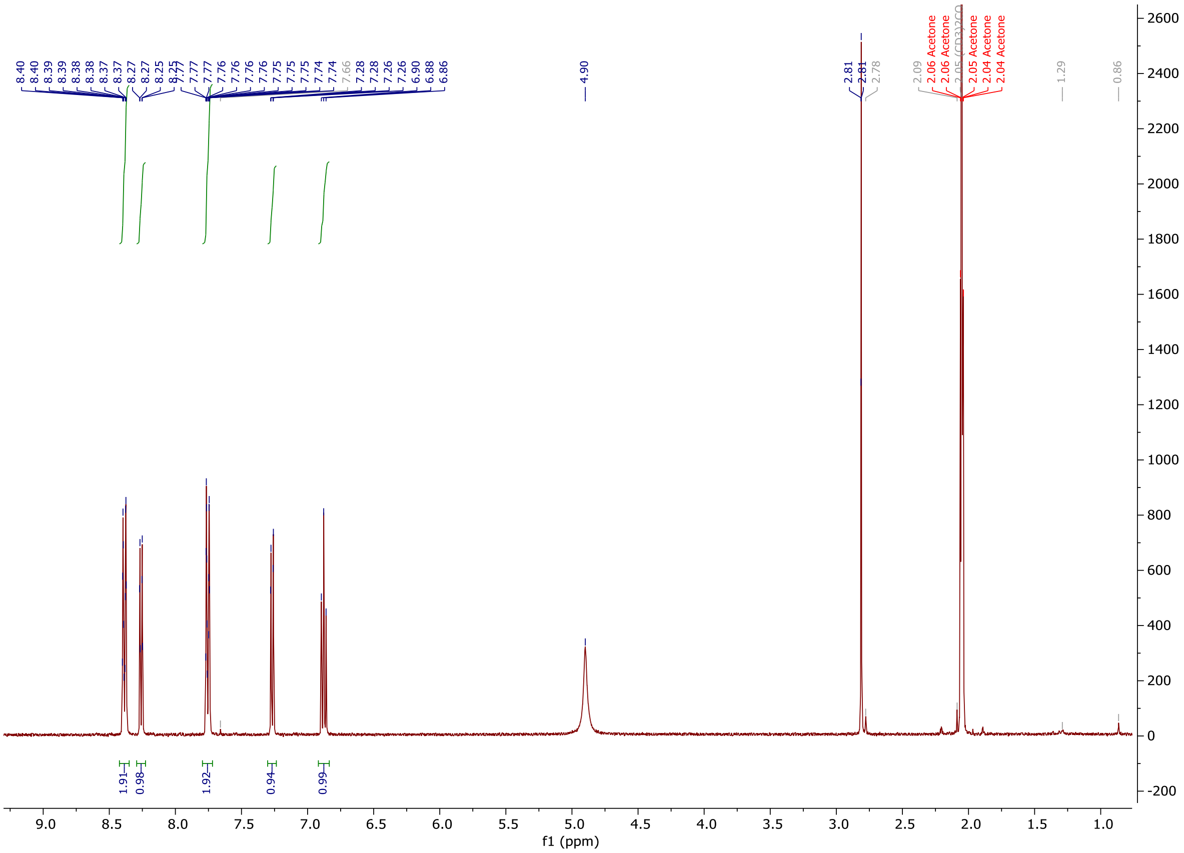

^{13}C spectrum of compound **22:**

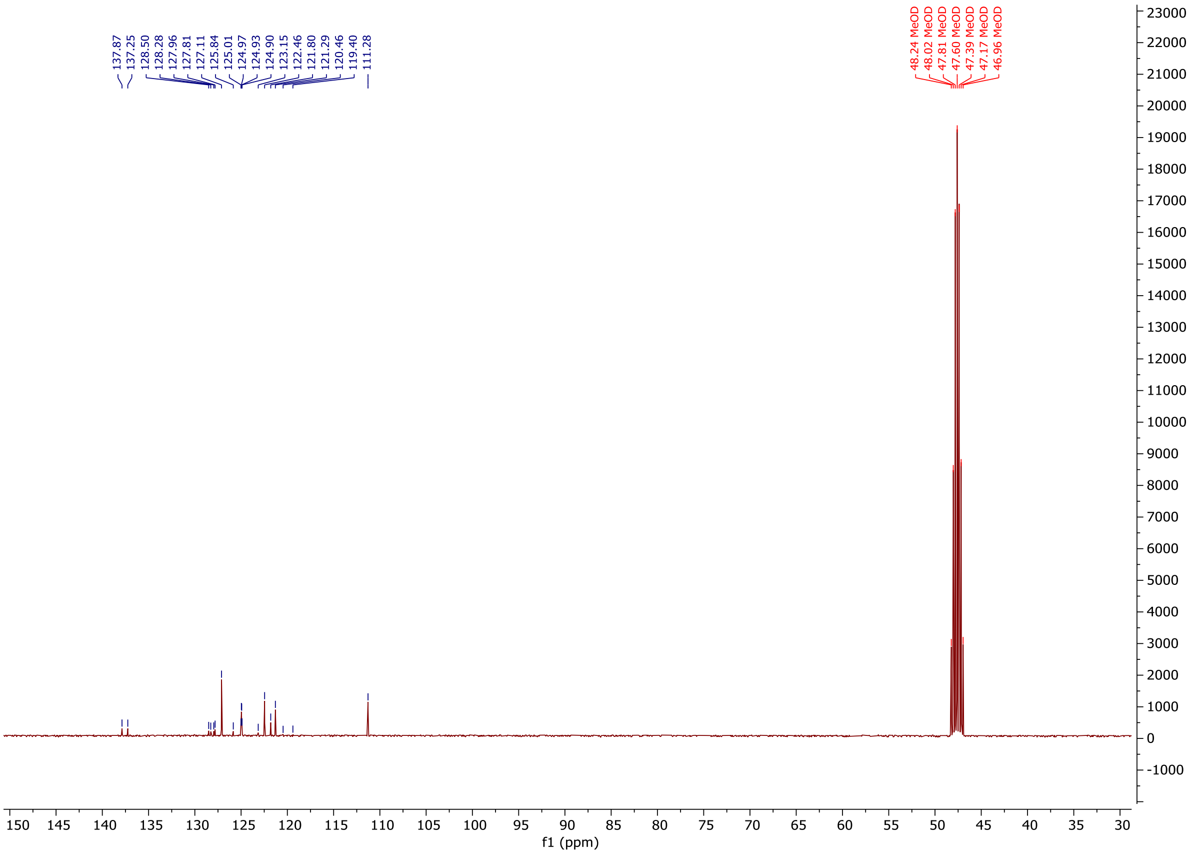

qHNMR spectrum of compound **22:**

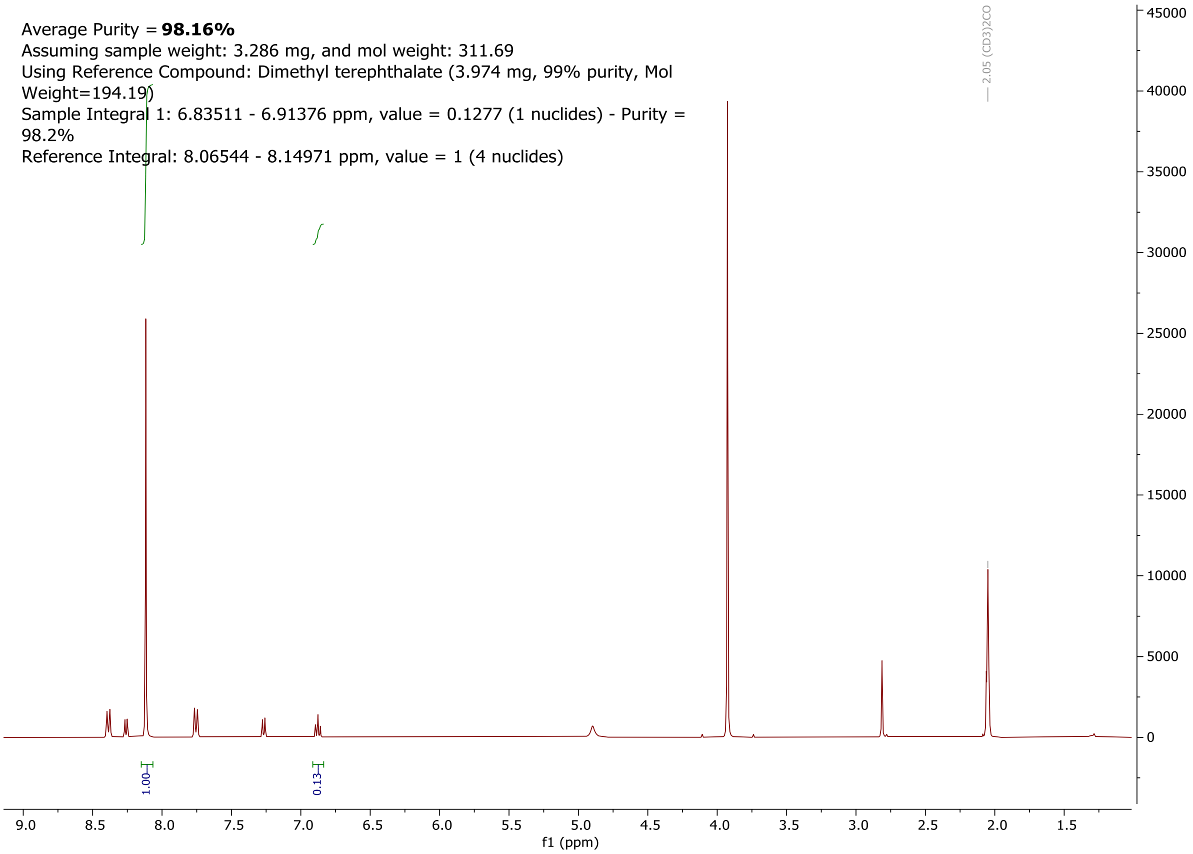

^{1}H spectrum of compound **23:**

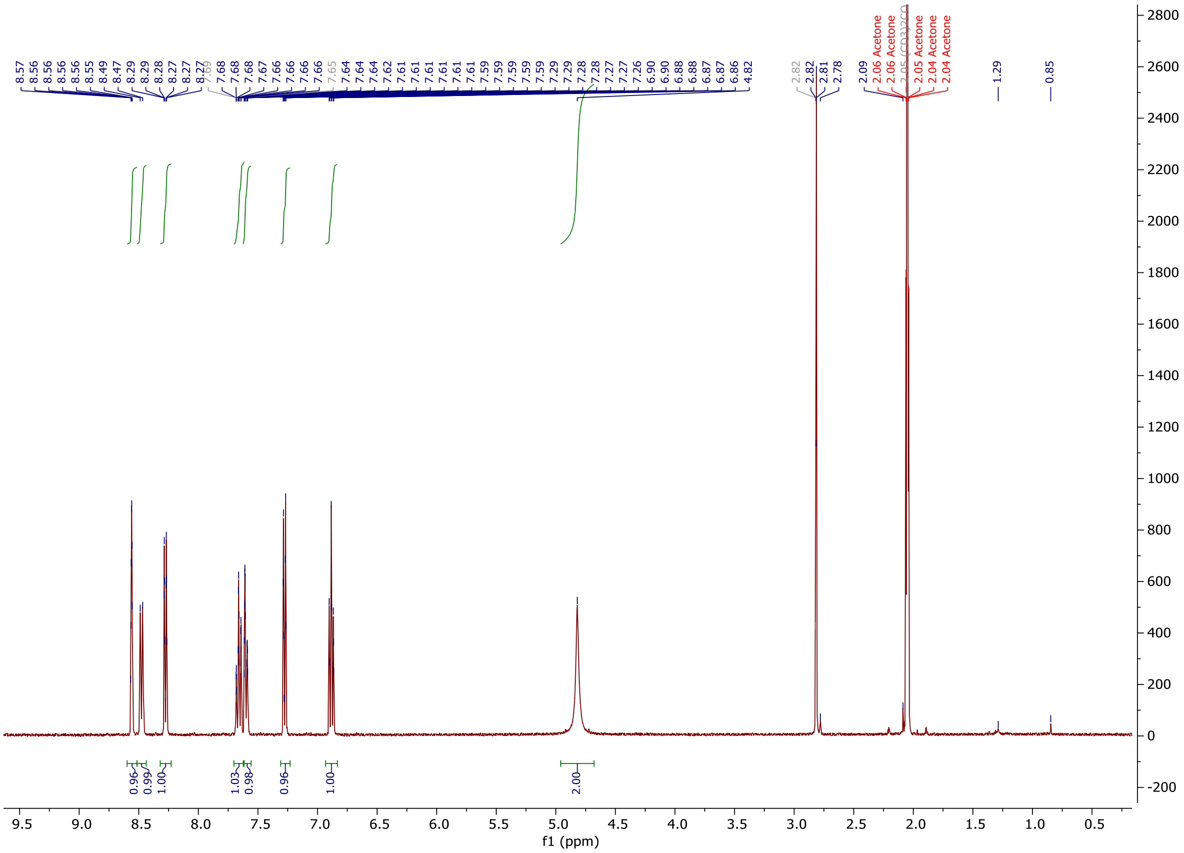

^{13}C spectrum of compound **23:**

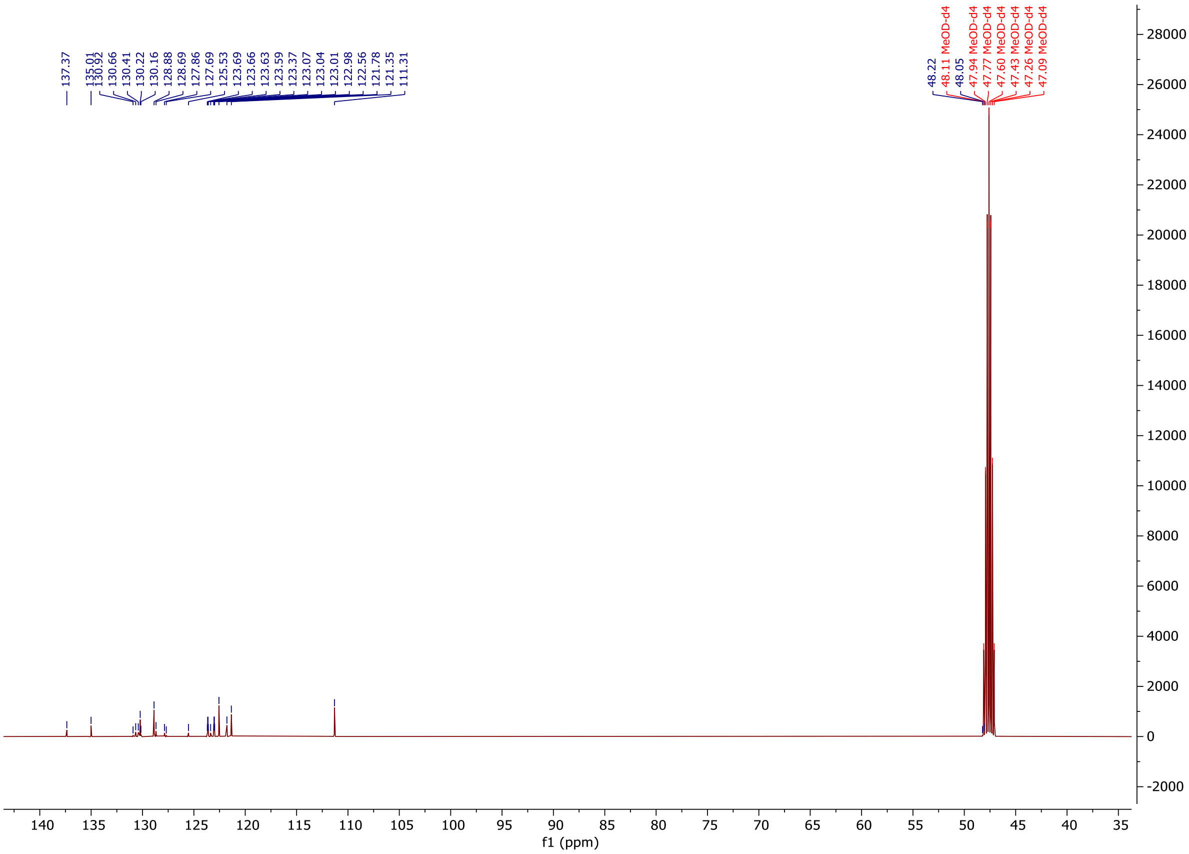

qHNMR spectrum of compound **23:**

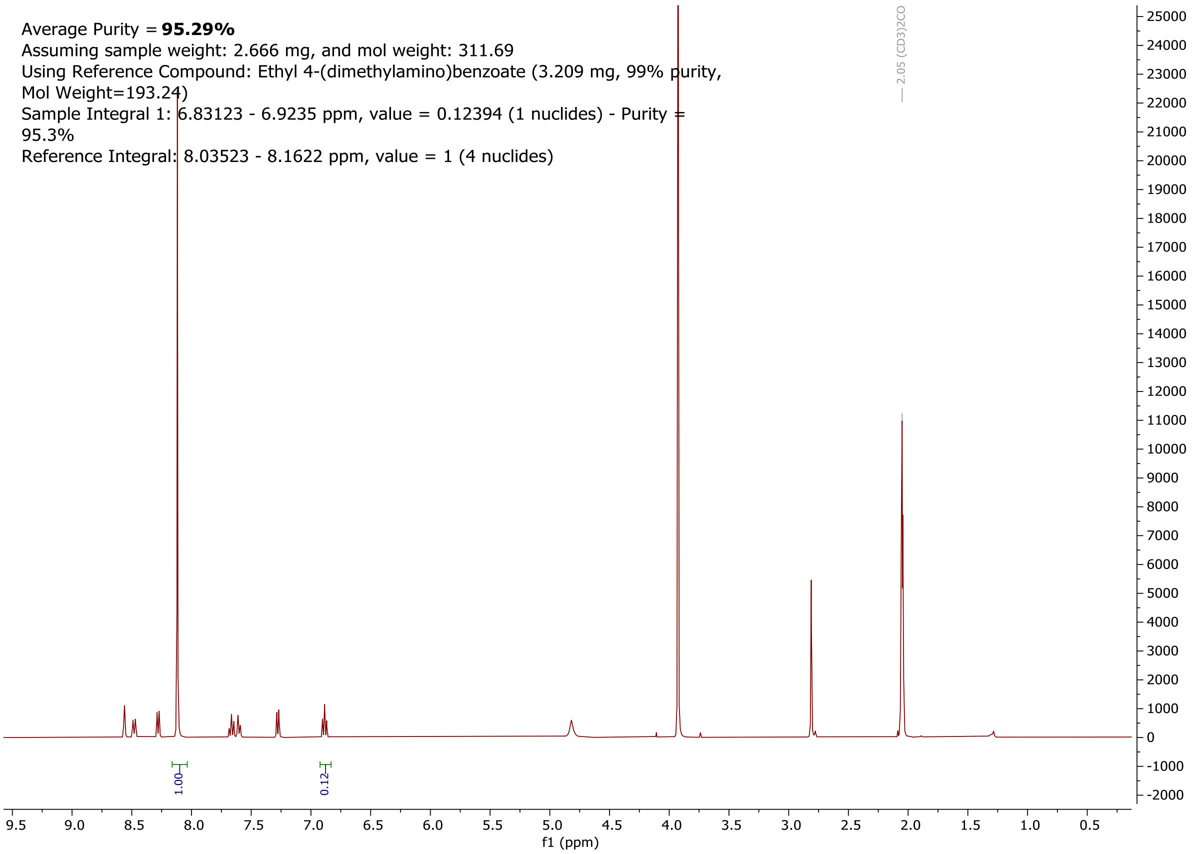

^1H spectrum of compound **24:**

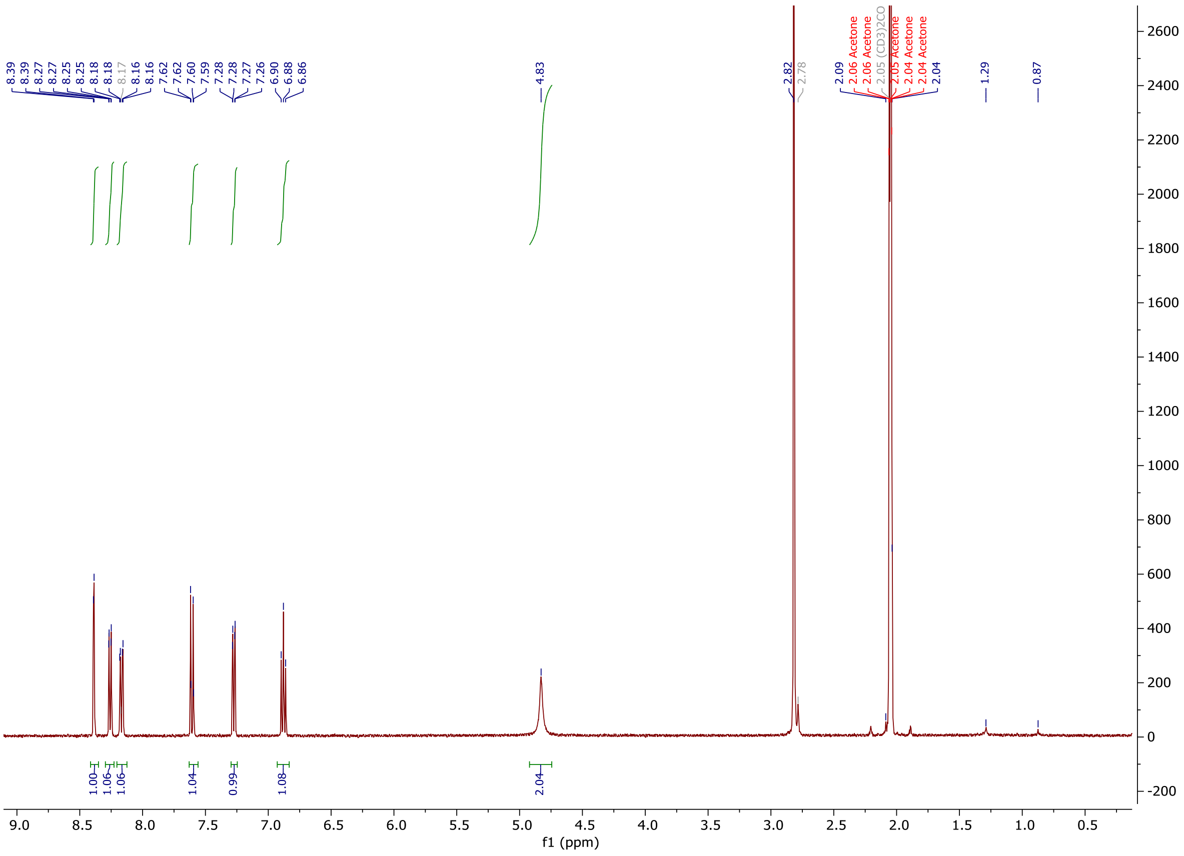

^{13}C spectrum of compound **24:**

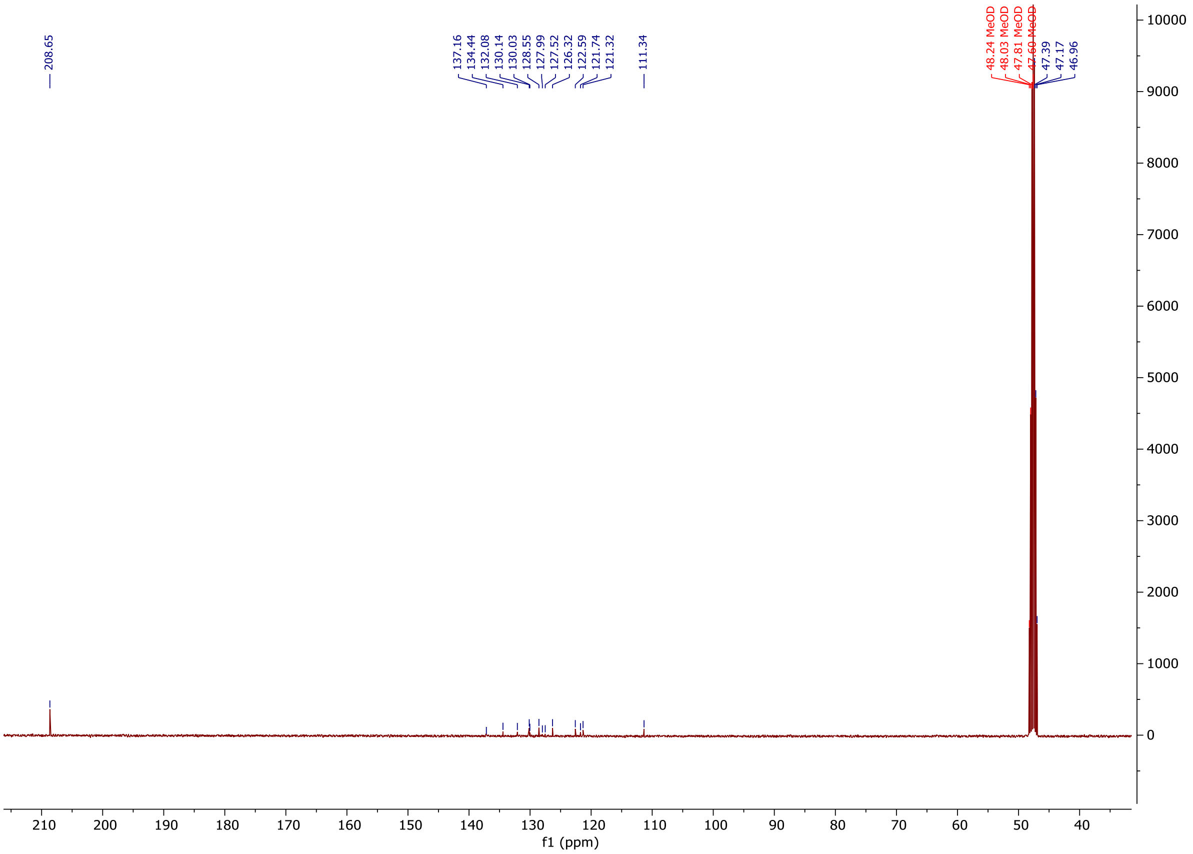

qHNMR spectrum of compound **24:**

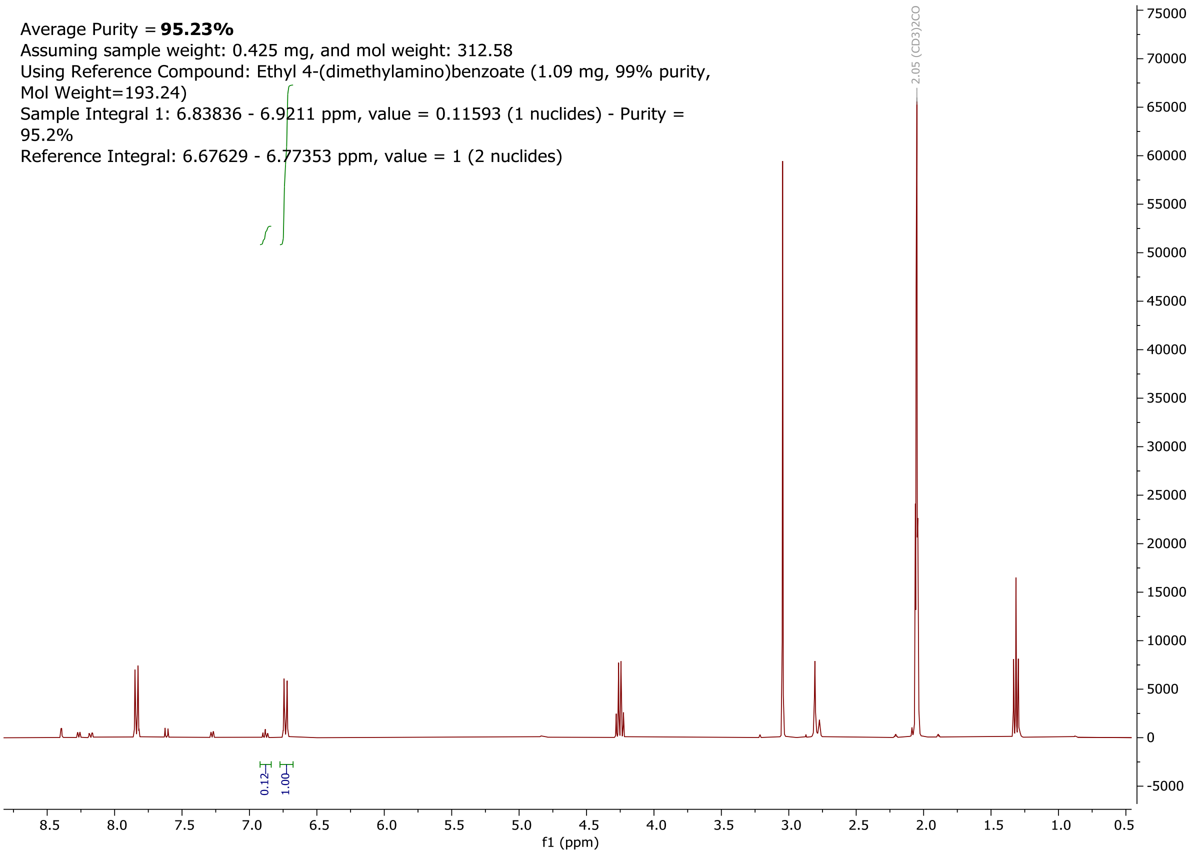

^{1}H spectrum of compound **25:**

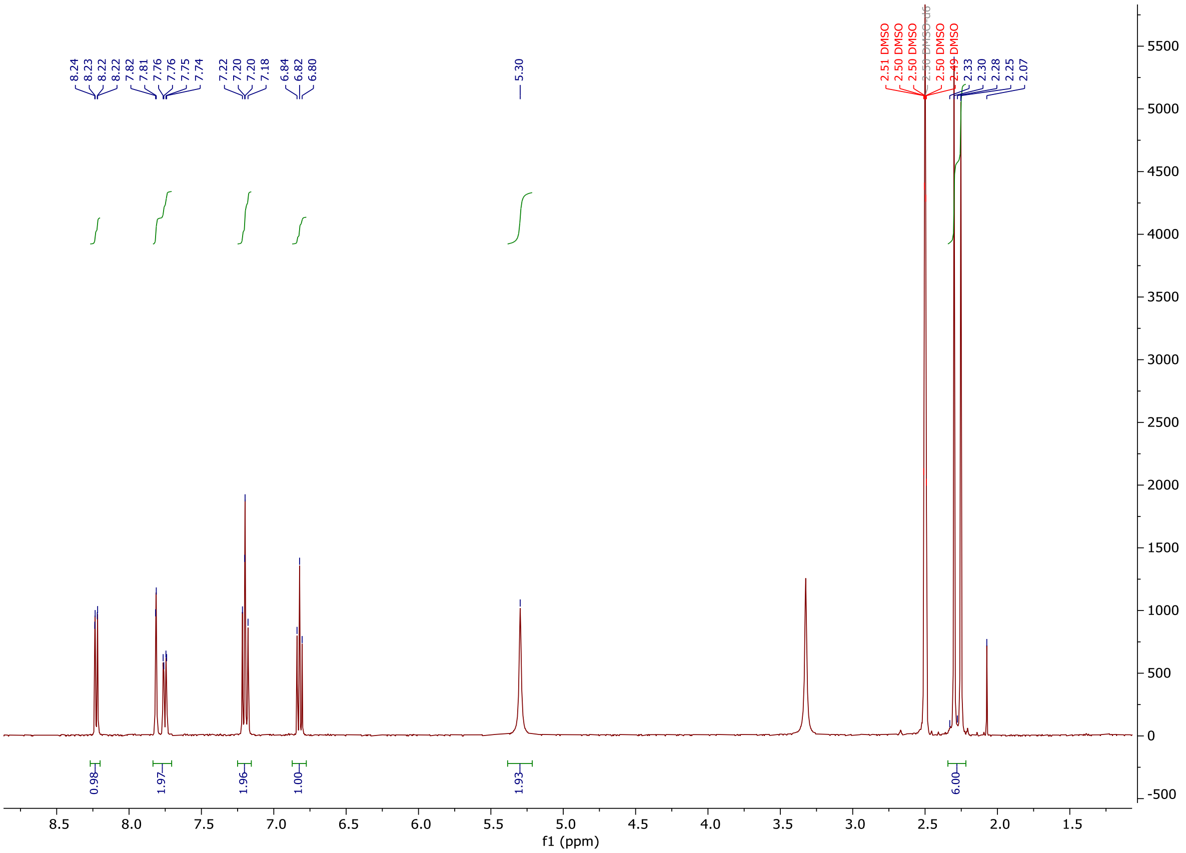

^{13}C spectrum of compound **25:**

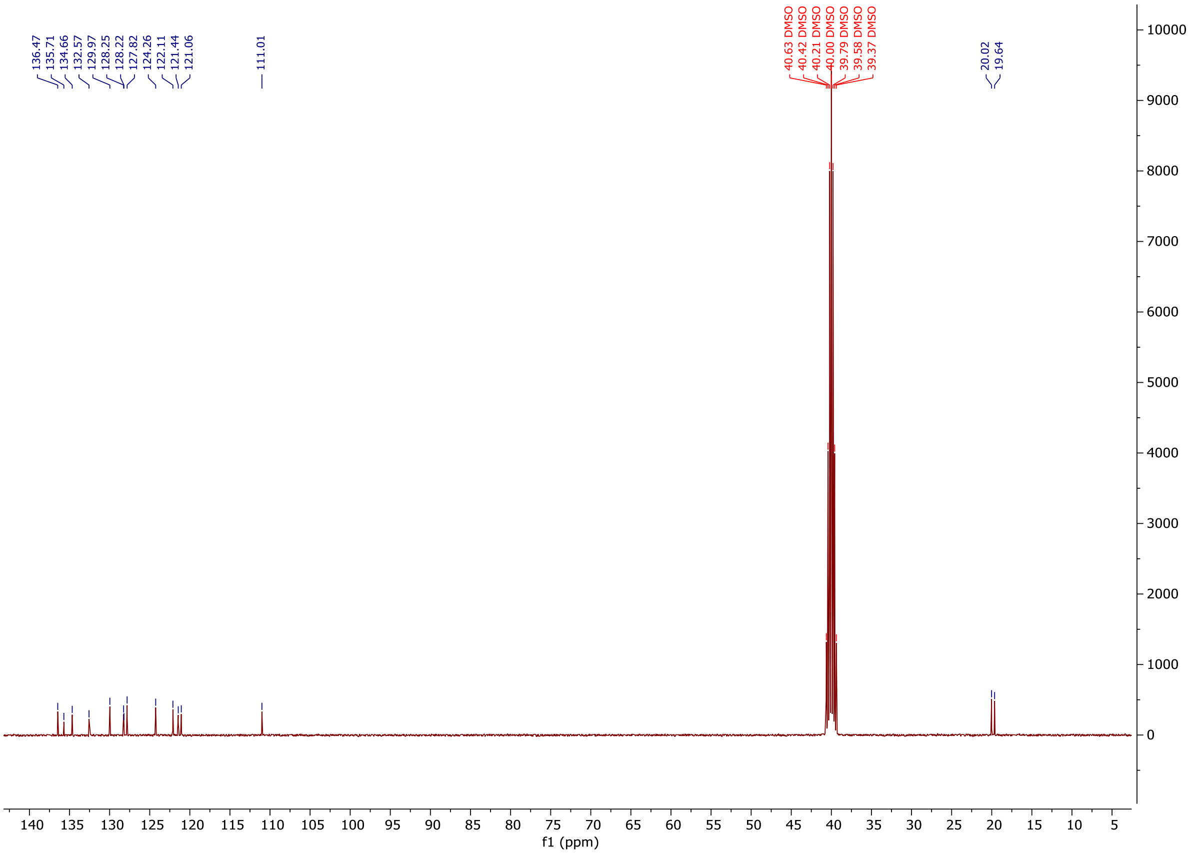

qHNMR spectrum of compound **25:**

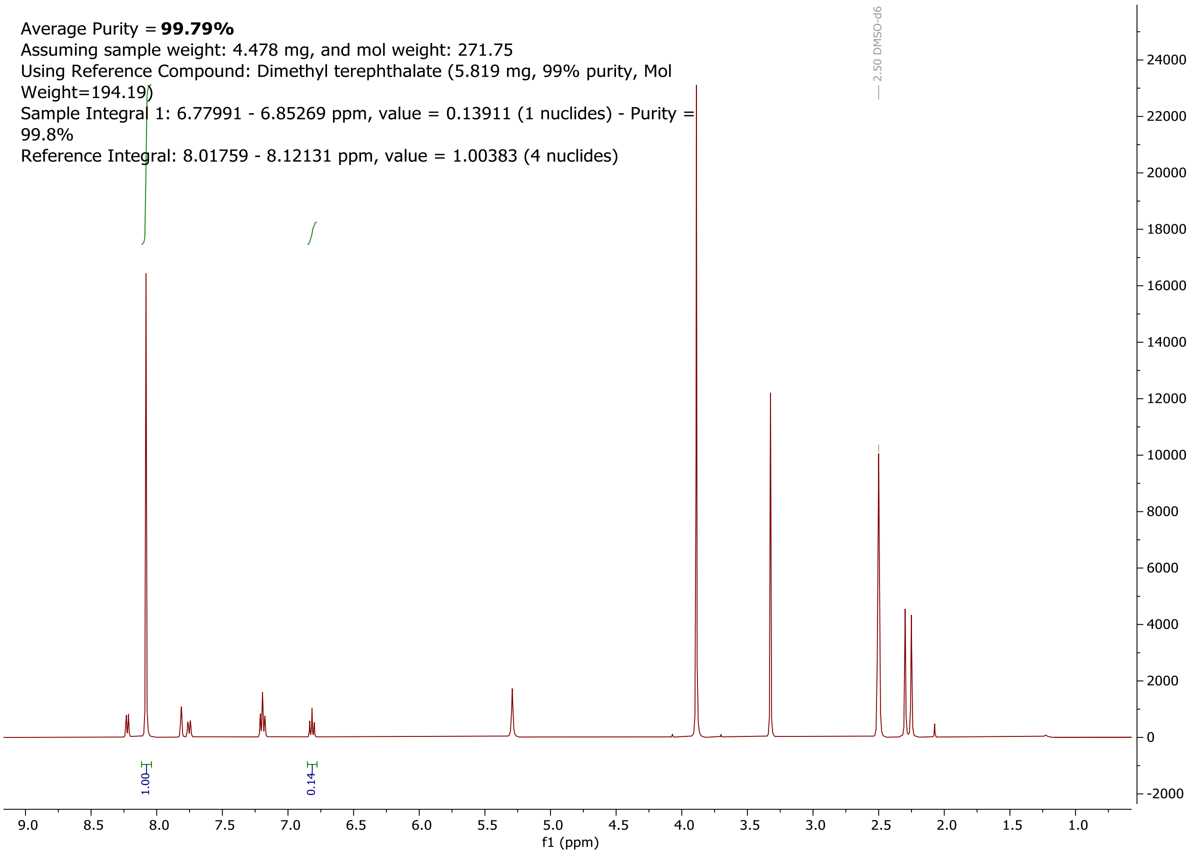

^{1}H spectrum of compound **26:**

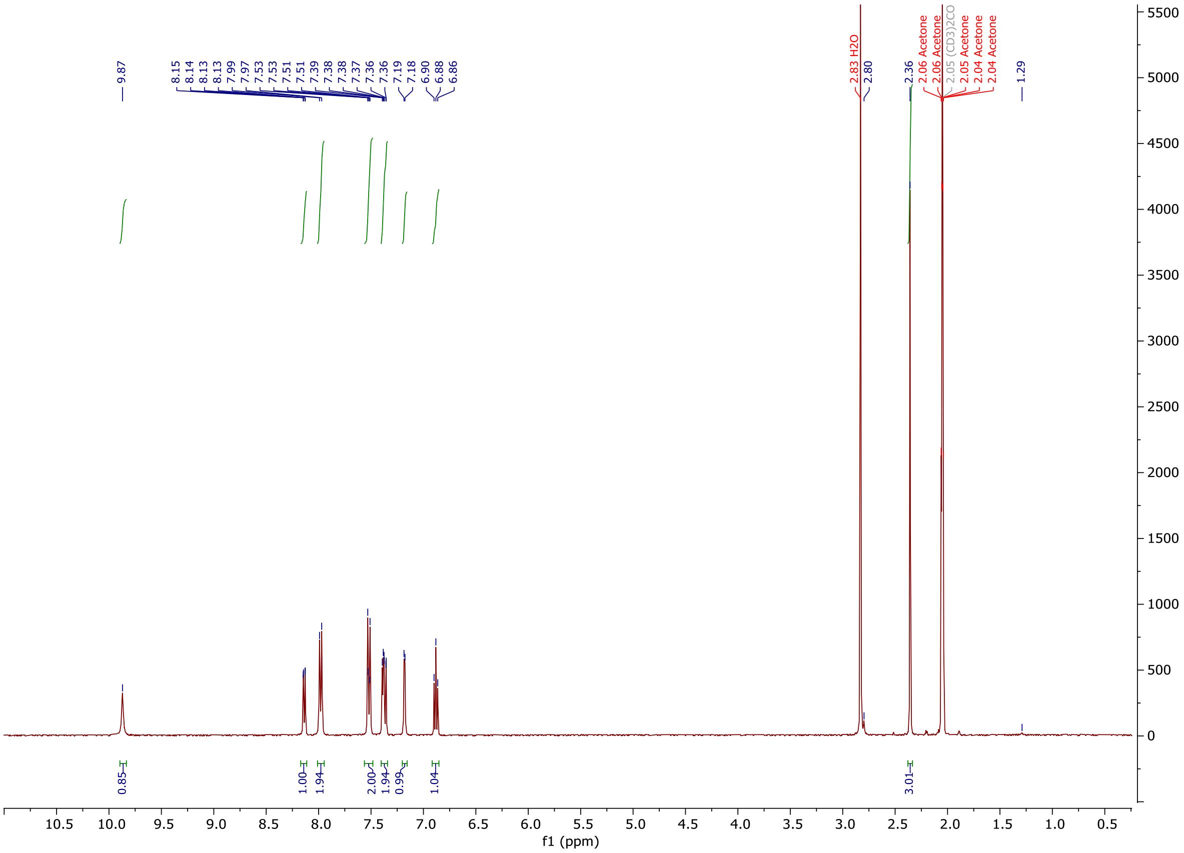

^{13}C spectrum of compound **26:**

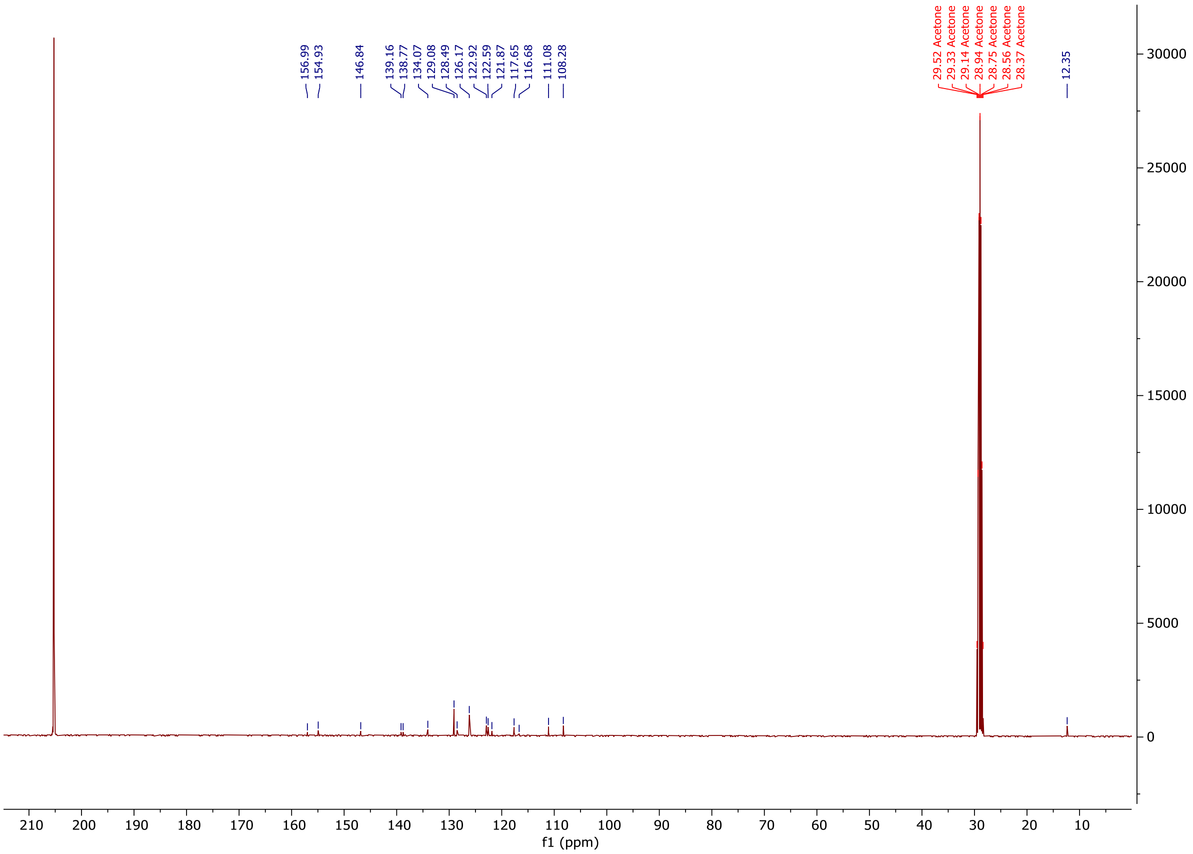

qHNMR spectrum of compound **26:**

Average Purity = **95.17%**
Assuming sample weight: 3.444 mg, and mol weight: 386.24
Using Reference Compound: Ethyl 4-(dimethylamino)benzoate (3.242 mg, 99% purity, Mol Weight=193.24)
Sample Integral 1: 7.05716 - 7.25629 ppm, value = 0.25546 (1 nuclides) - Purity = 95.2%
Reference Integral: 4.14199 - 4.31567 ppm, value = 1 (2 nuclides)

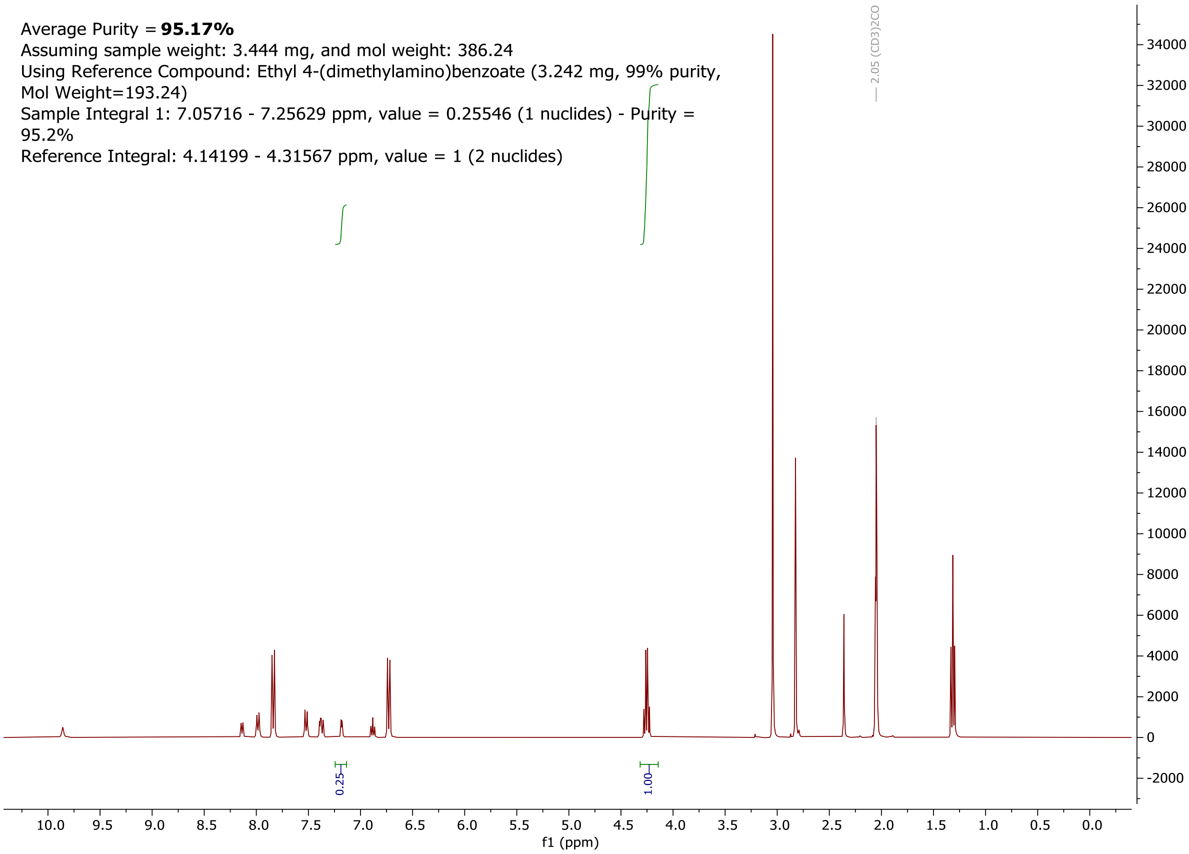

^{1}H spectrum of compound **27:**

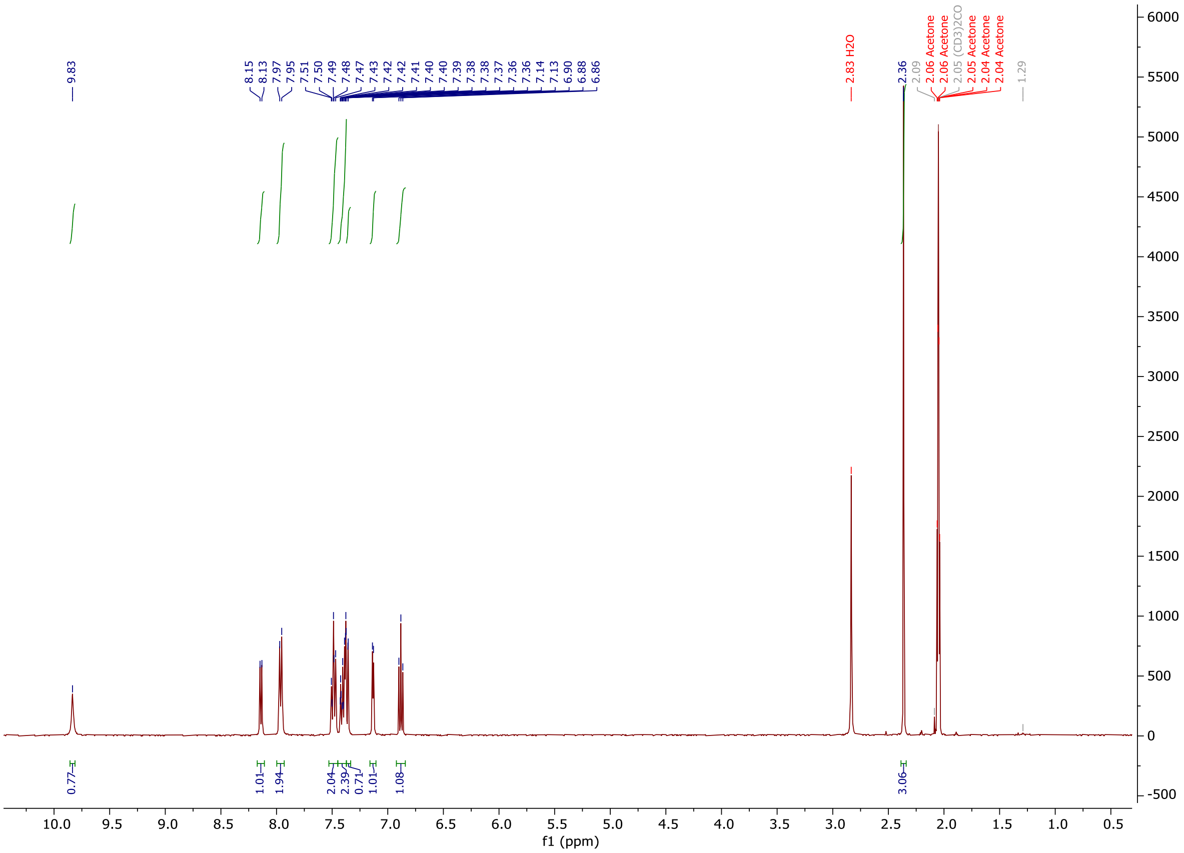

^{13}C spectrum of compound **27:**

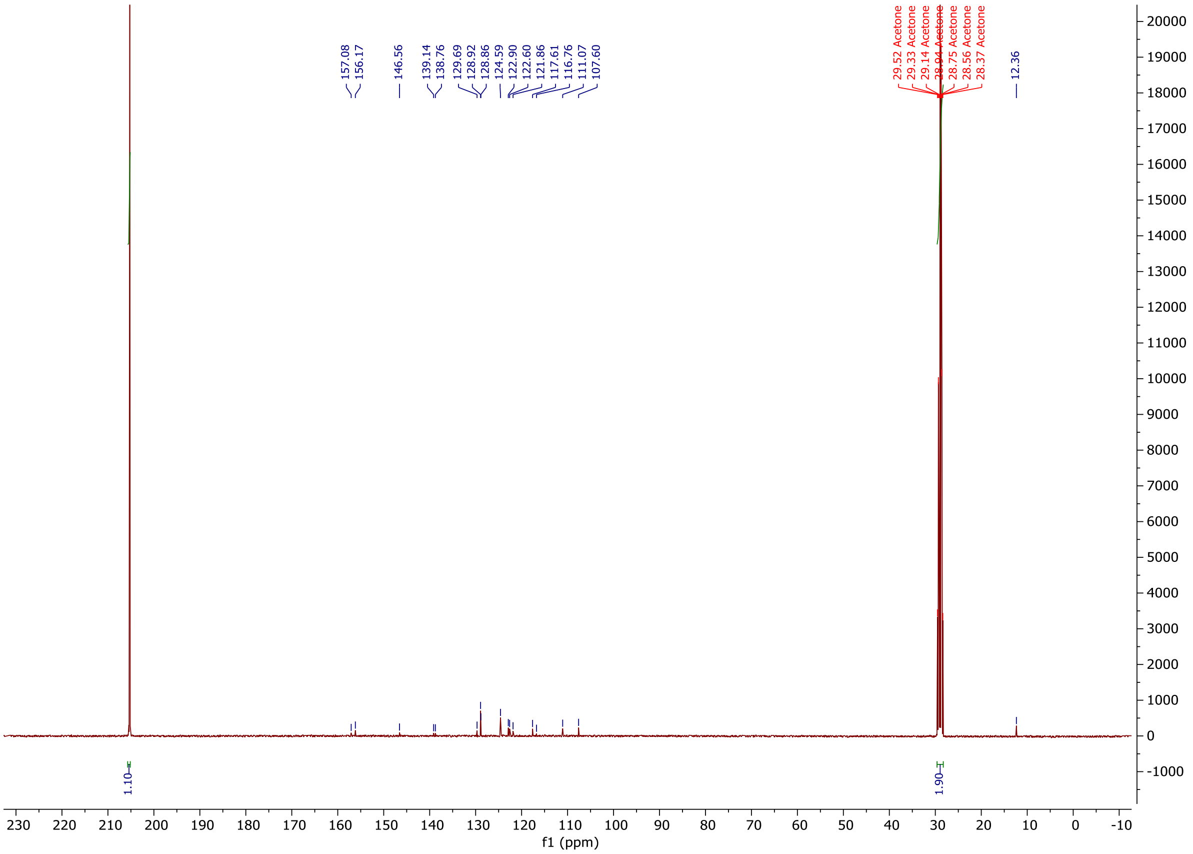

qHNMR spectrum of compound **27:**

Average Purity = **96.06%**
Assuming sample weight: 2.658 mg, and mol weight: 351.79
Using Reference Compound: Ethyl 4-(dimethylamino)benzoate (3.397 mg, 99% purity, Mol Weight=193.24)
Sample Integral 1: 6.85088 - 6.90967 ppm, value = 0.20852 (1 nuclides) - Purity = 96.1%
Reference Integral: 6.68527 - 6.7598 ppm, value = 1 (2 nuclides)

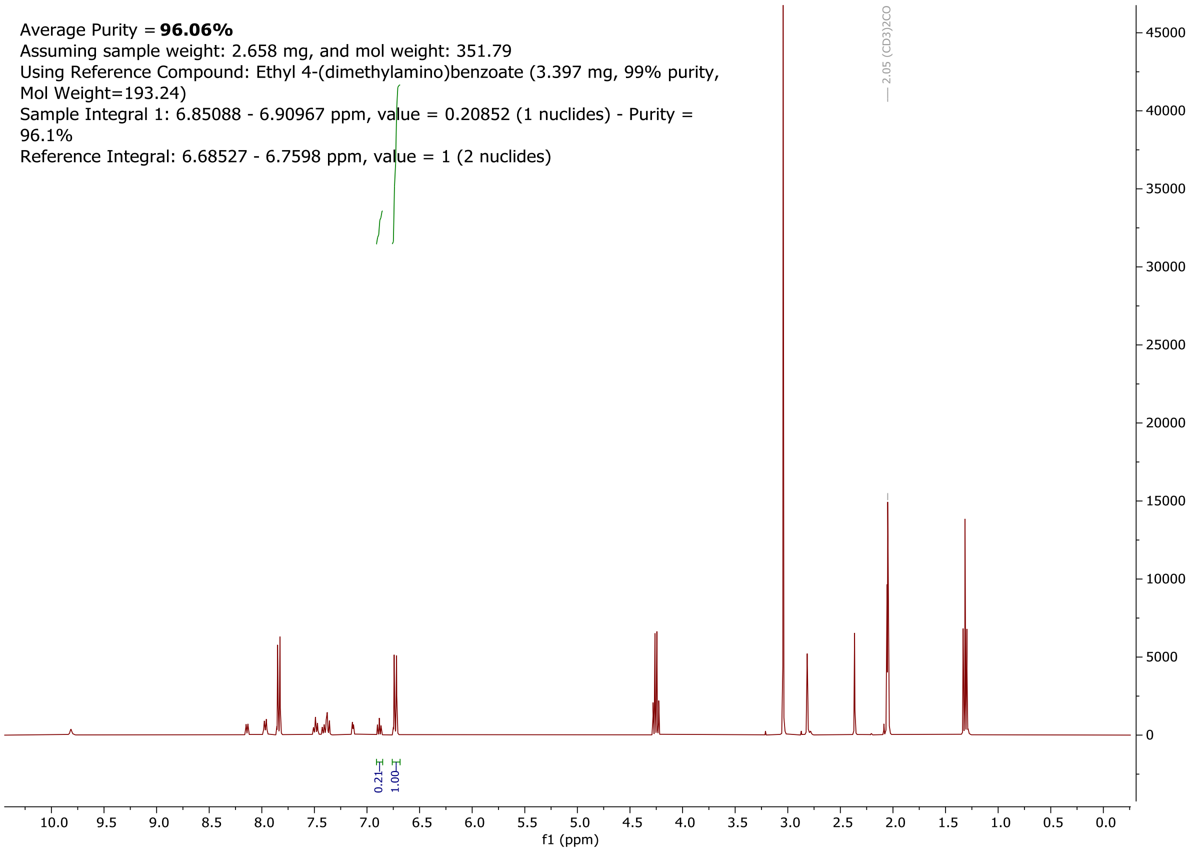

^{1}H spectrum of compound **28:**

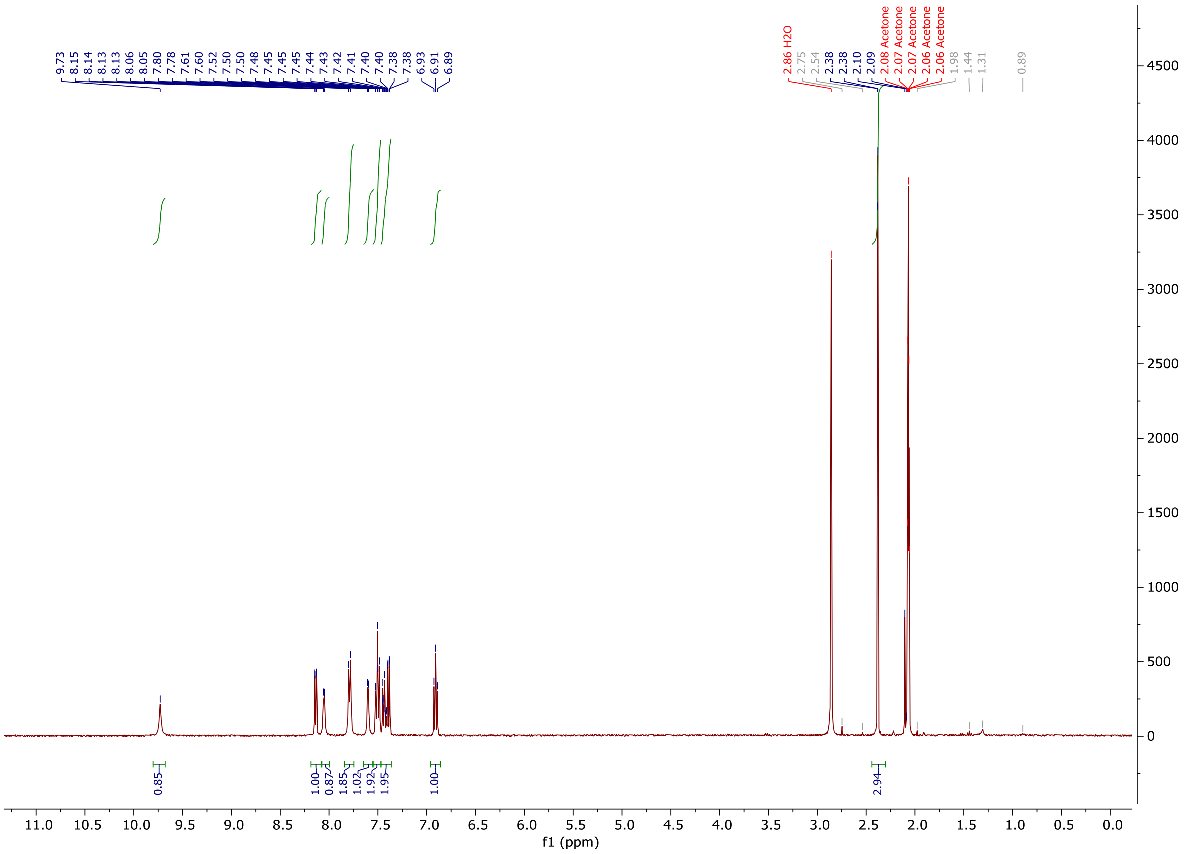

^{13}C spectrum of compound **28:**

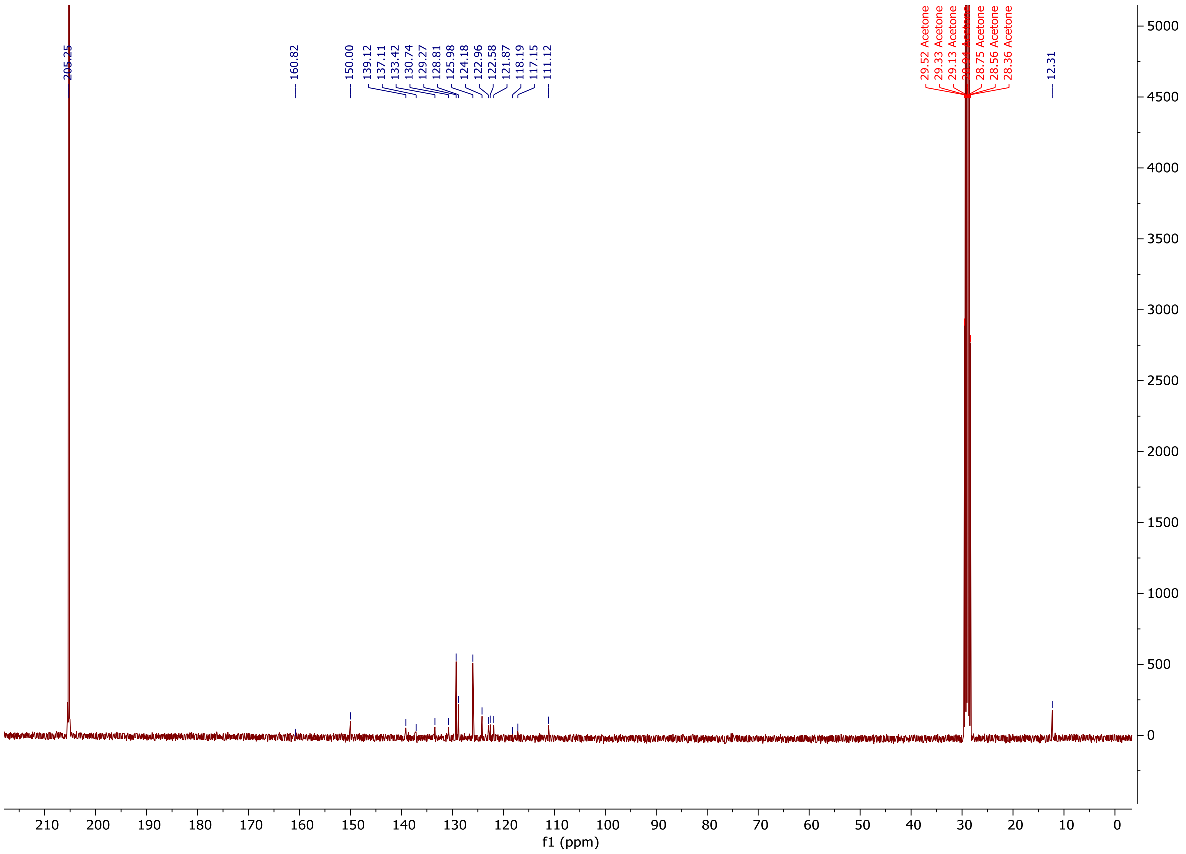

qHNMR spectrum of compound **28:**

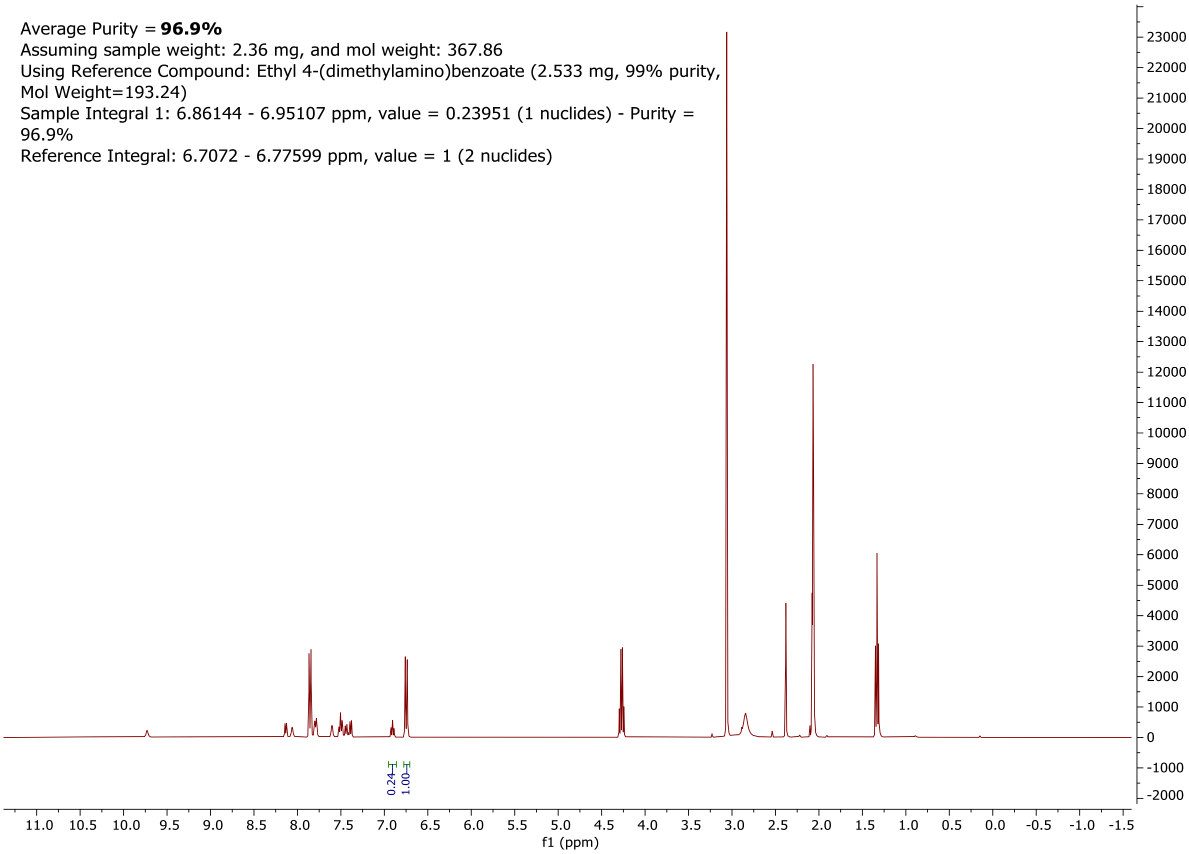

^{1}H spectrum of compound **29:**

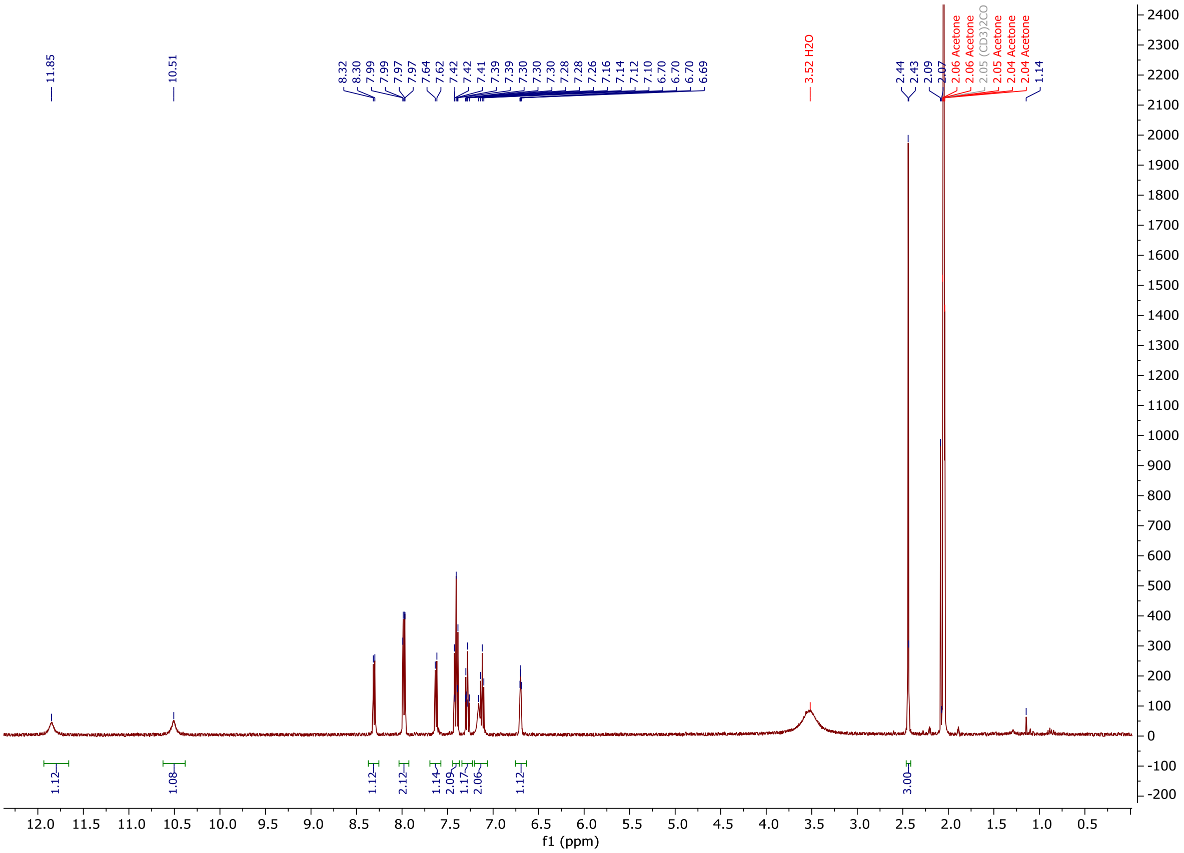

^{13}C spectrum of compound **29:**

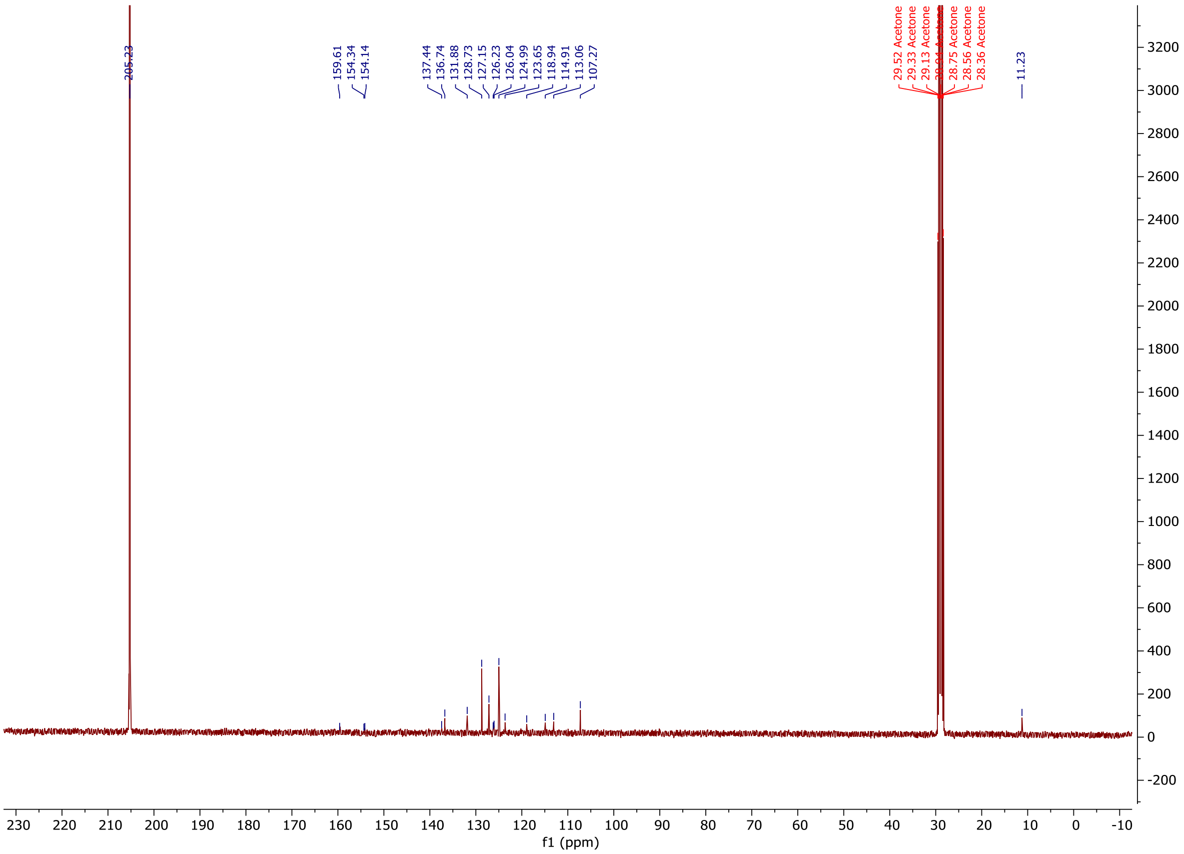

qHNMR spectrum of compound **29:**

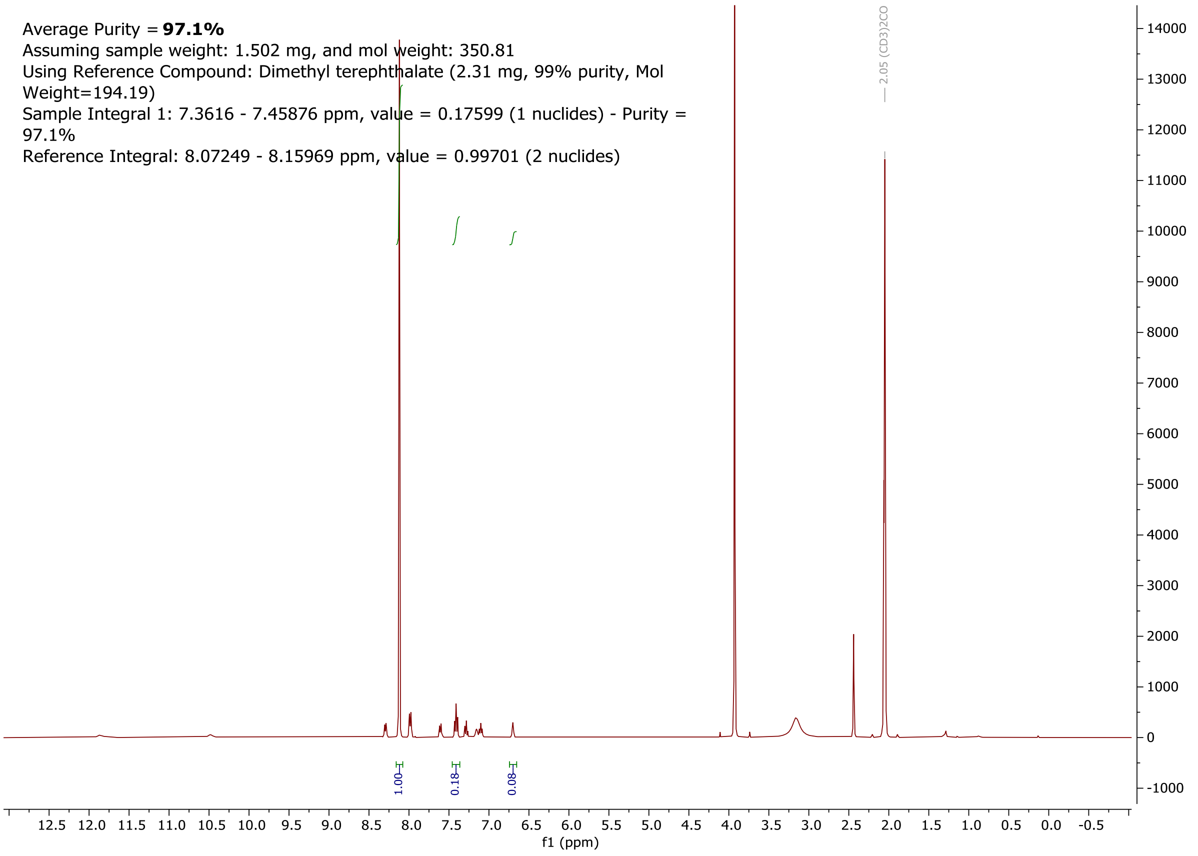

^{1}H spectrum of compound **30:**

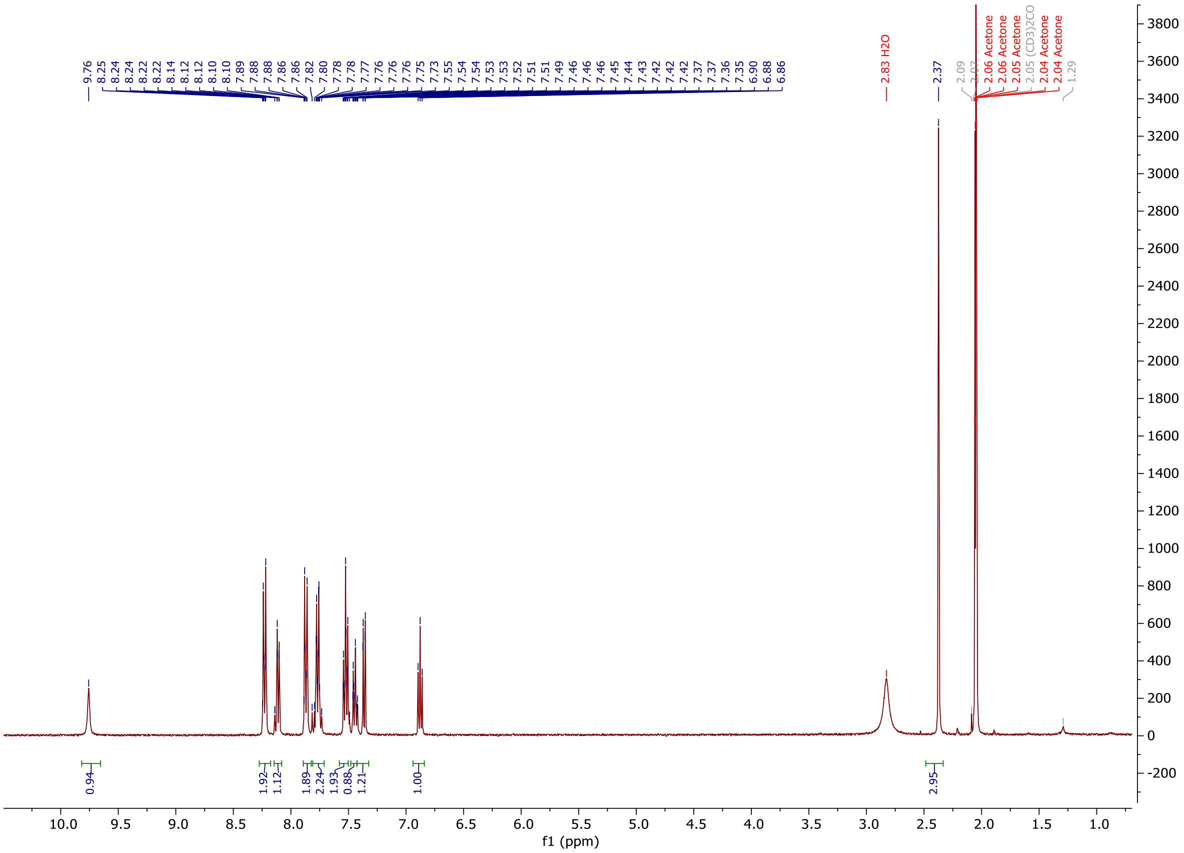

^{13}C spectrum of compound **30:**

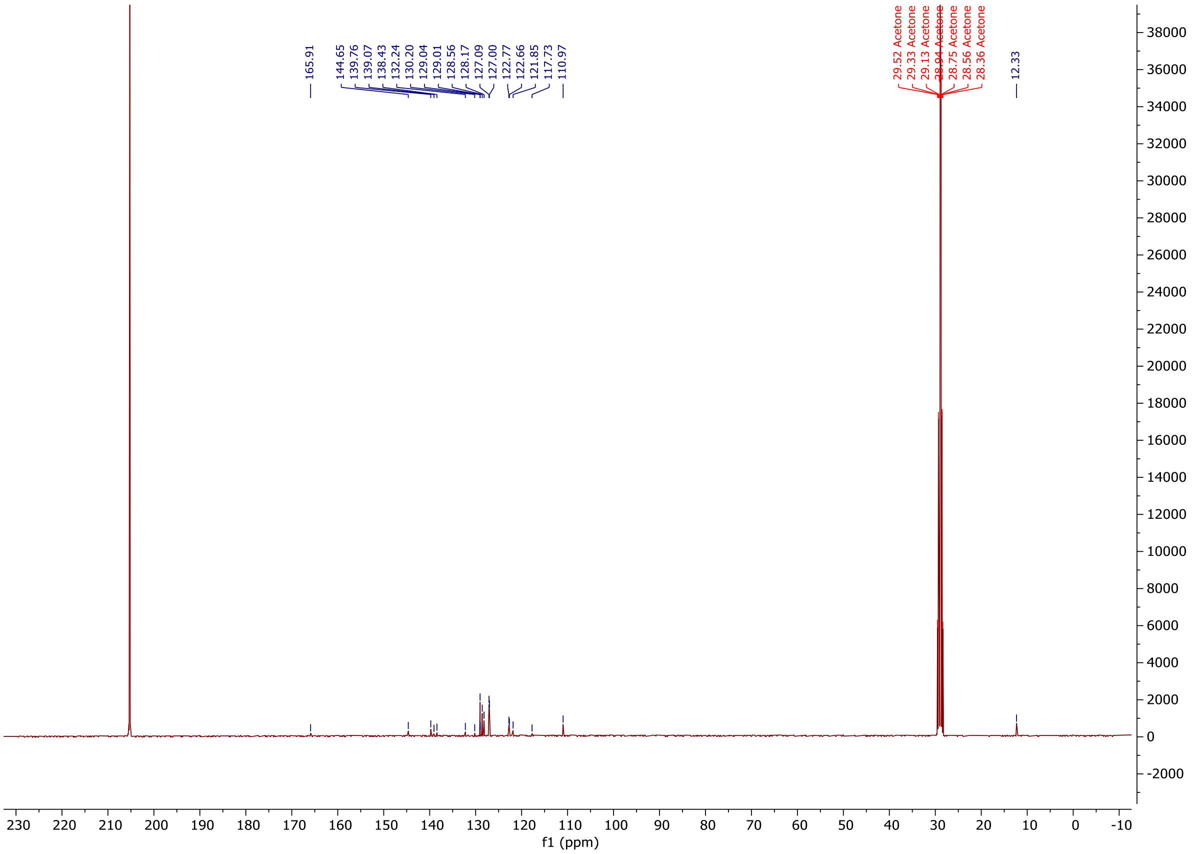

qHNMR spectrum of compound **30:**

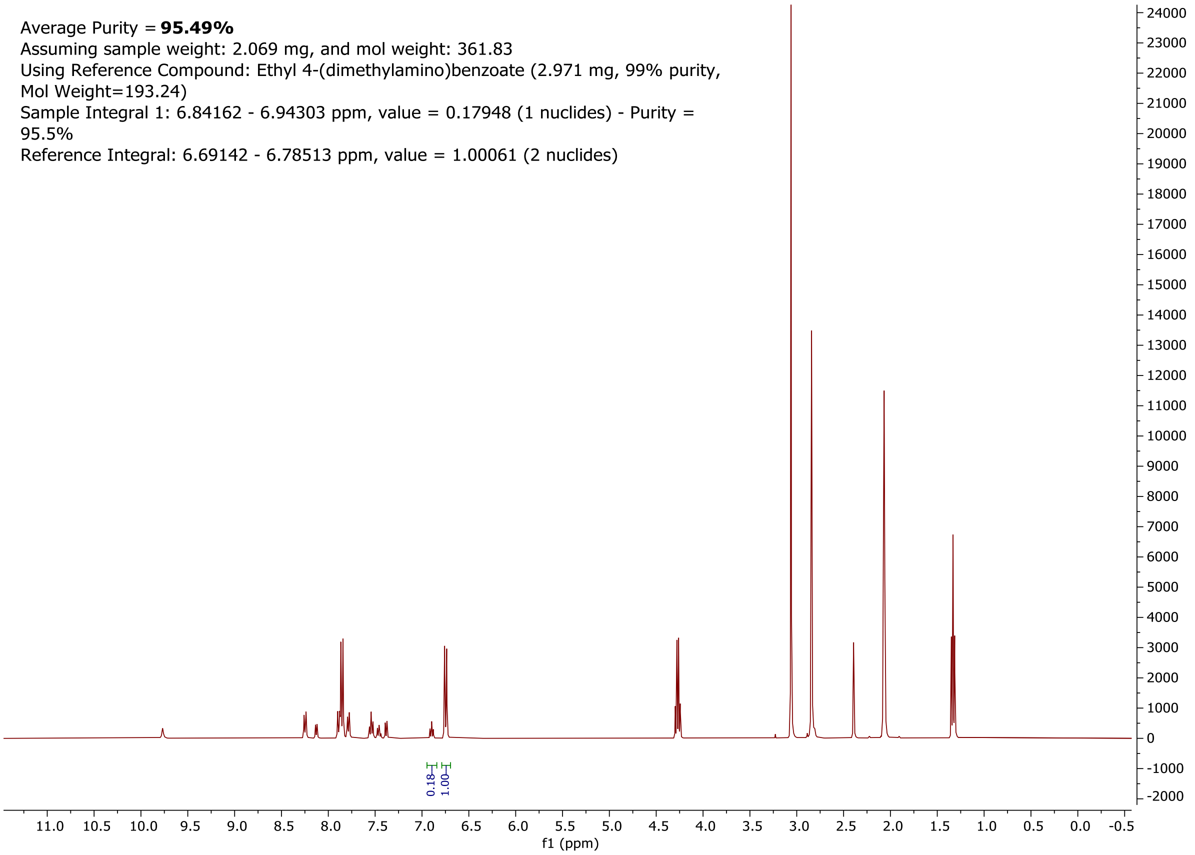

^{1}H spectrum of compound **31:**

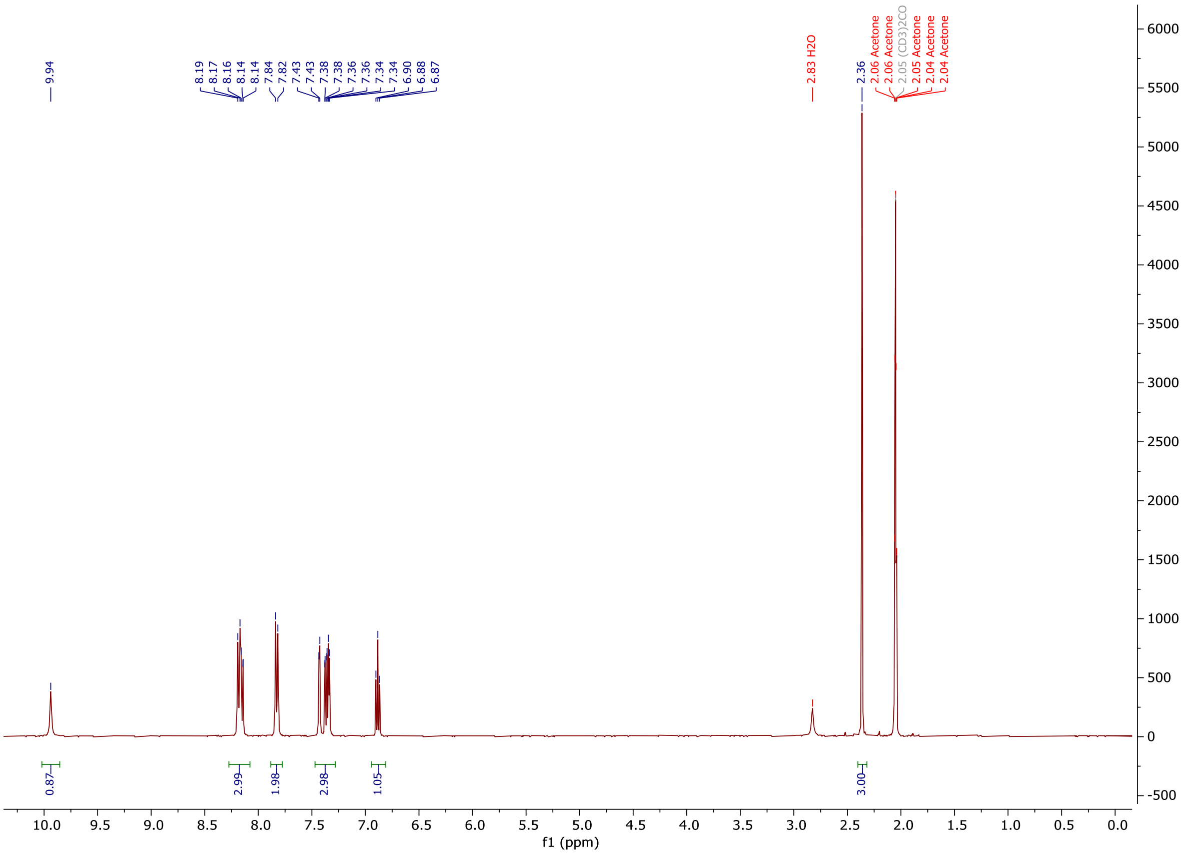

^{13}C spectrum of compound **31:**

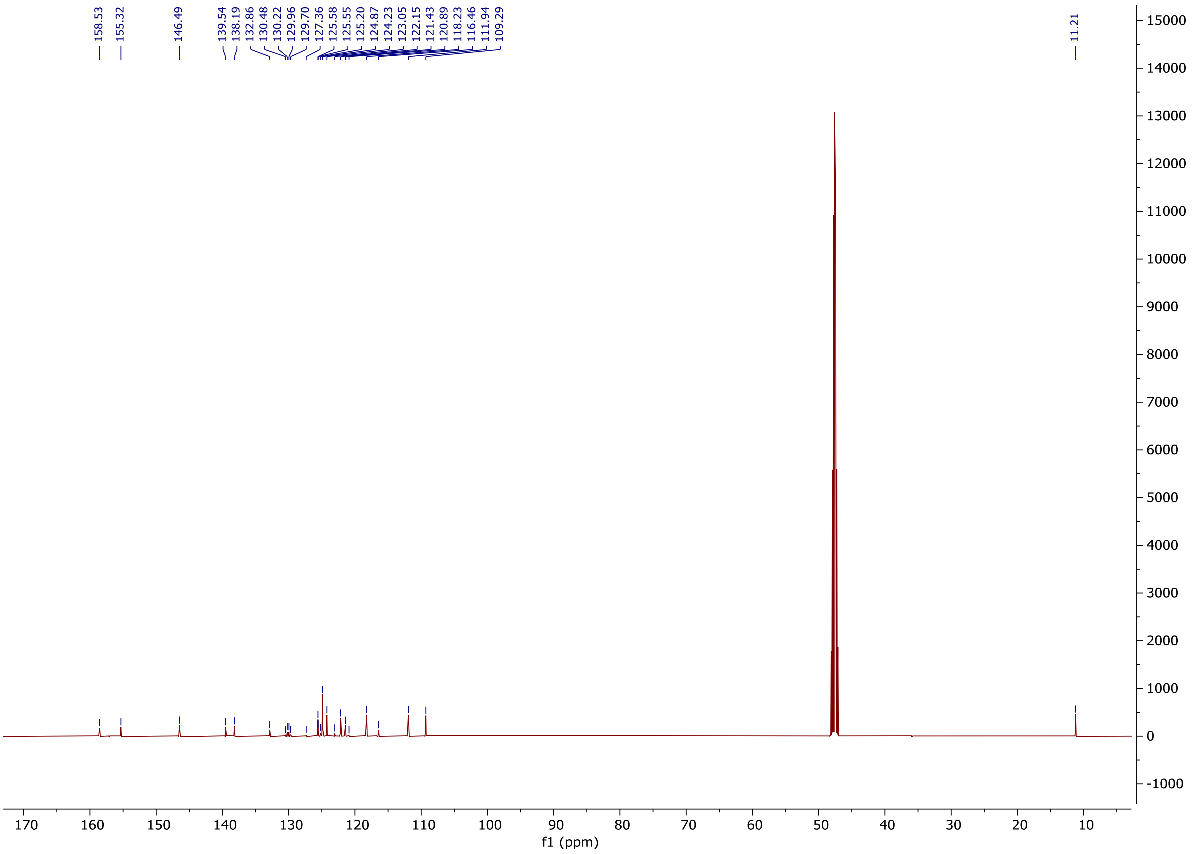

qHNMR spectrum of compound **31:**

Average Purity = **98.9%**
Assuming sample weight: 2.679 mg, and mol weight: 419.79
Using Reference Compound: Ethyl 4-(dimethylamino)benzoate (2.693 mg, 99% purity, Mol Weight=193.24)
Sample Integral 1: 6.83379 - 6.93664 ppm, value = 0.22874 (1 nuclides) - Purity = 98.9%
Reference Integral: 6.66412 - 6.76961 ppm, value = 1 (2 nuclides)

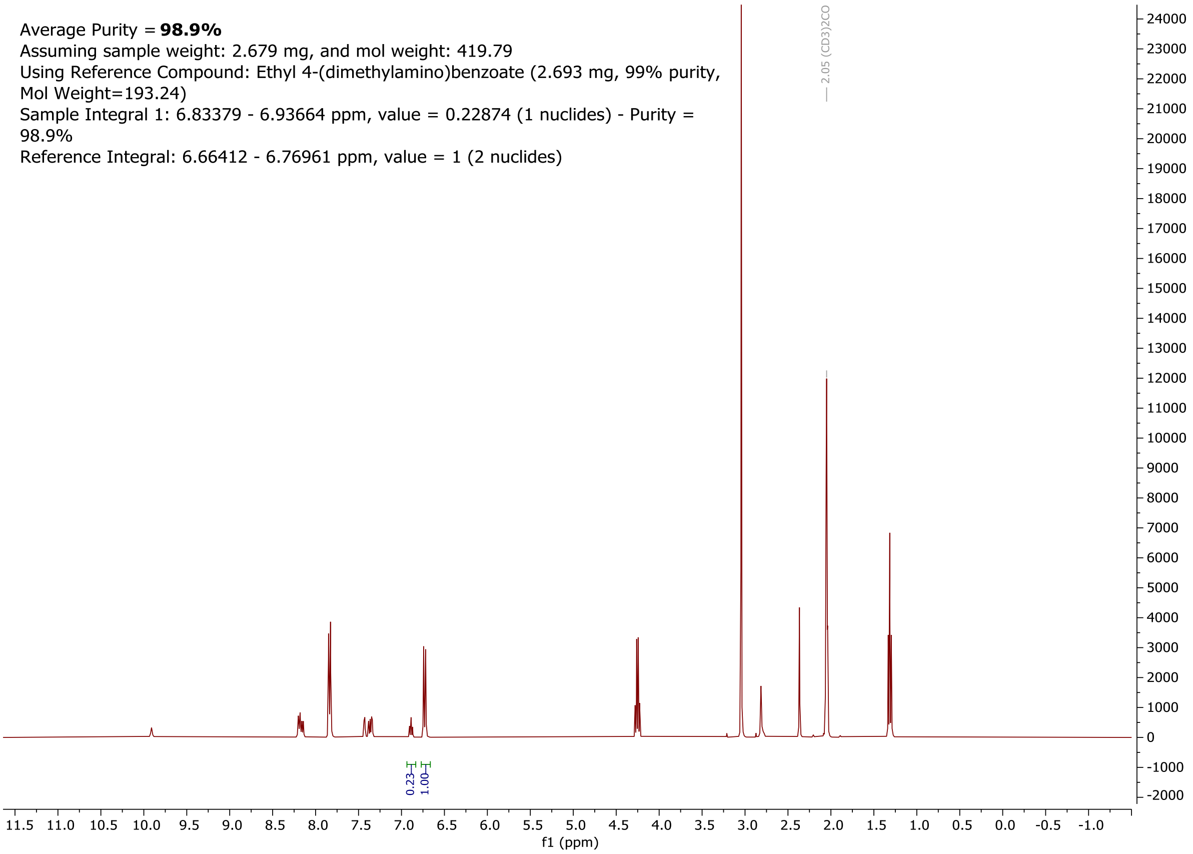

^{1}H spectrum of compound **32:**

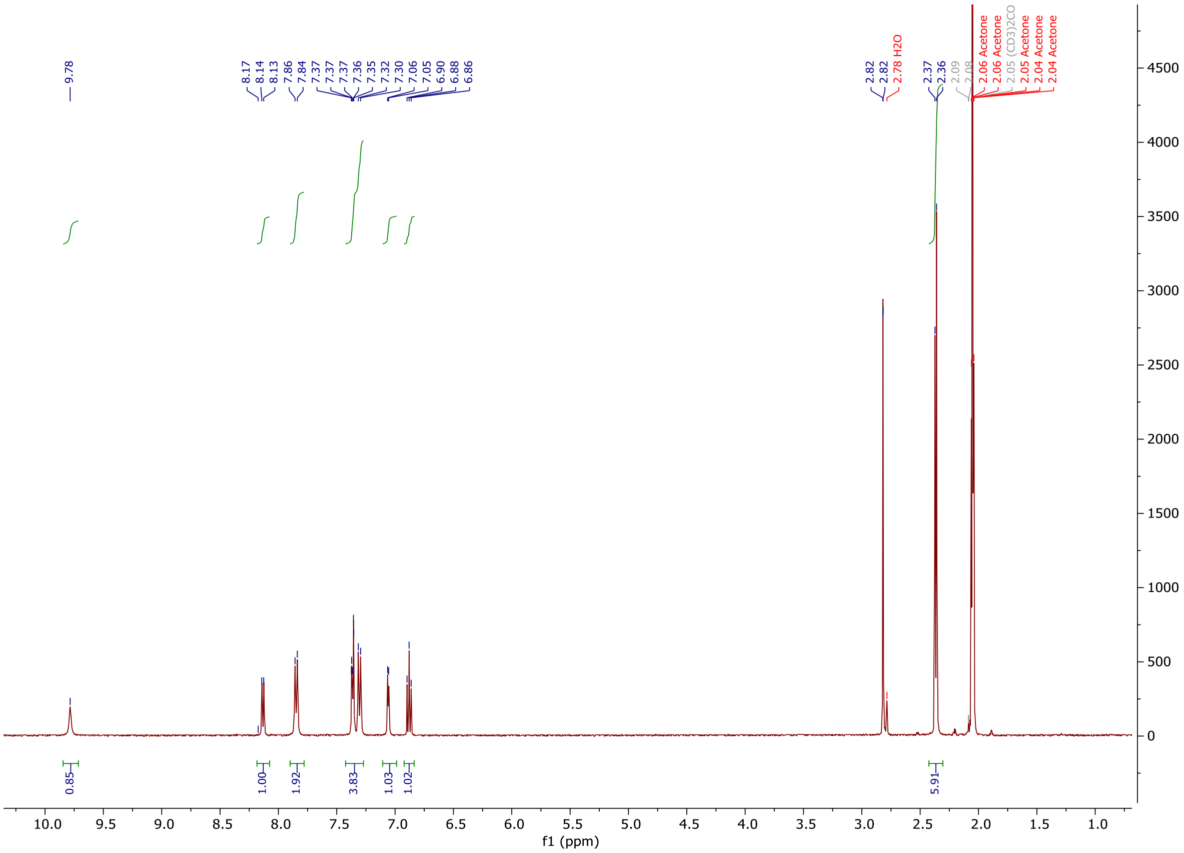

^{13}C spectrum of compound **32:**

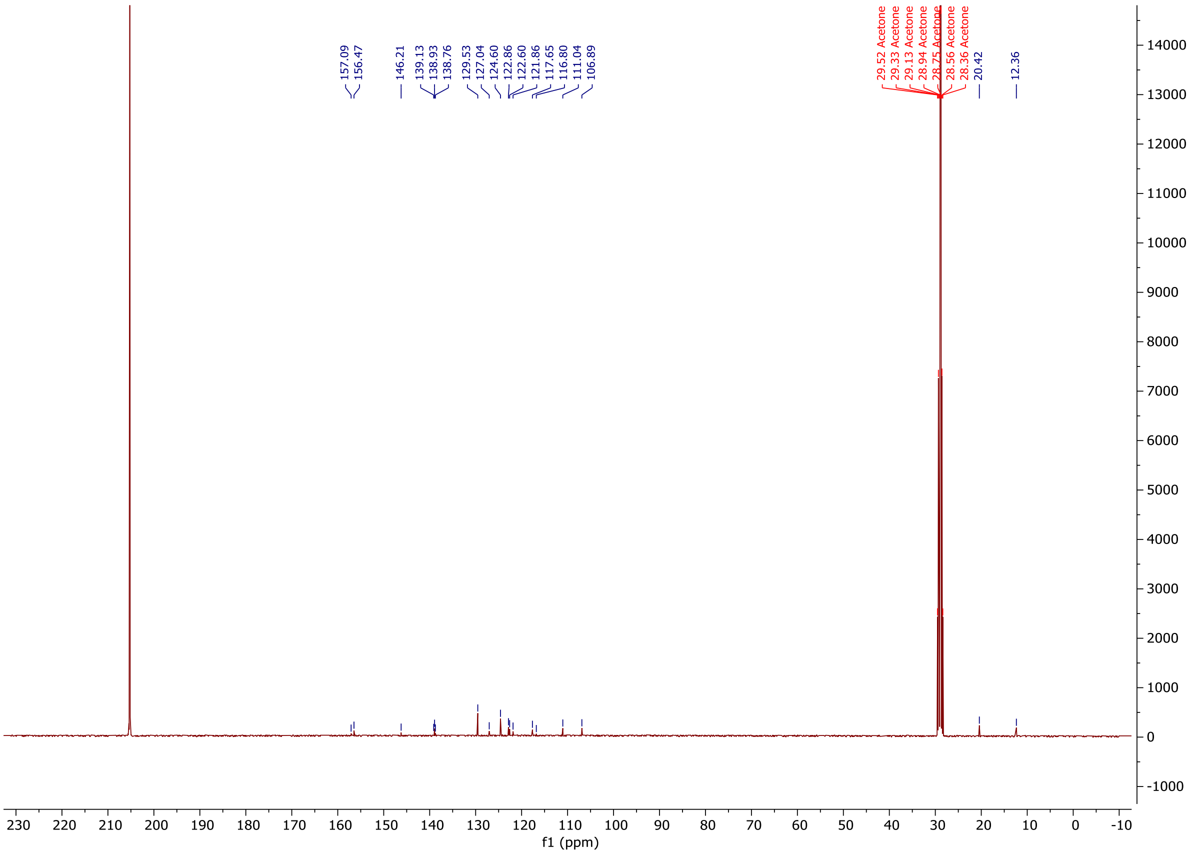

qHNMR spectrum of compound **32:**

Average Purity = **97.82%**
Assuming sample weight: 3.268 mg, and mol weight: 365.82
Using Reference Compound: Ethyl 4-(dimethylamino)benzoate (3.124 mg, 99% purity, Mol Weight=193.24)
Sample Integral 1: 6.85088 - 6.93784 ppm, value = 0.27301 (1 nuclides) - Purity = 97.8%
Reference Integral: 6.68174 - 6.78894 ppm, value = 1 (2 nuclides)

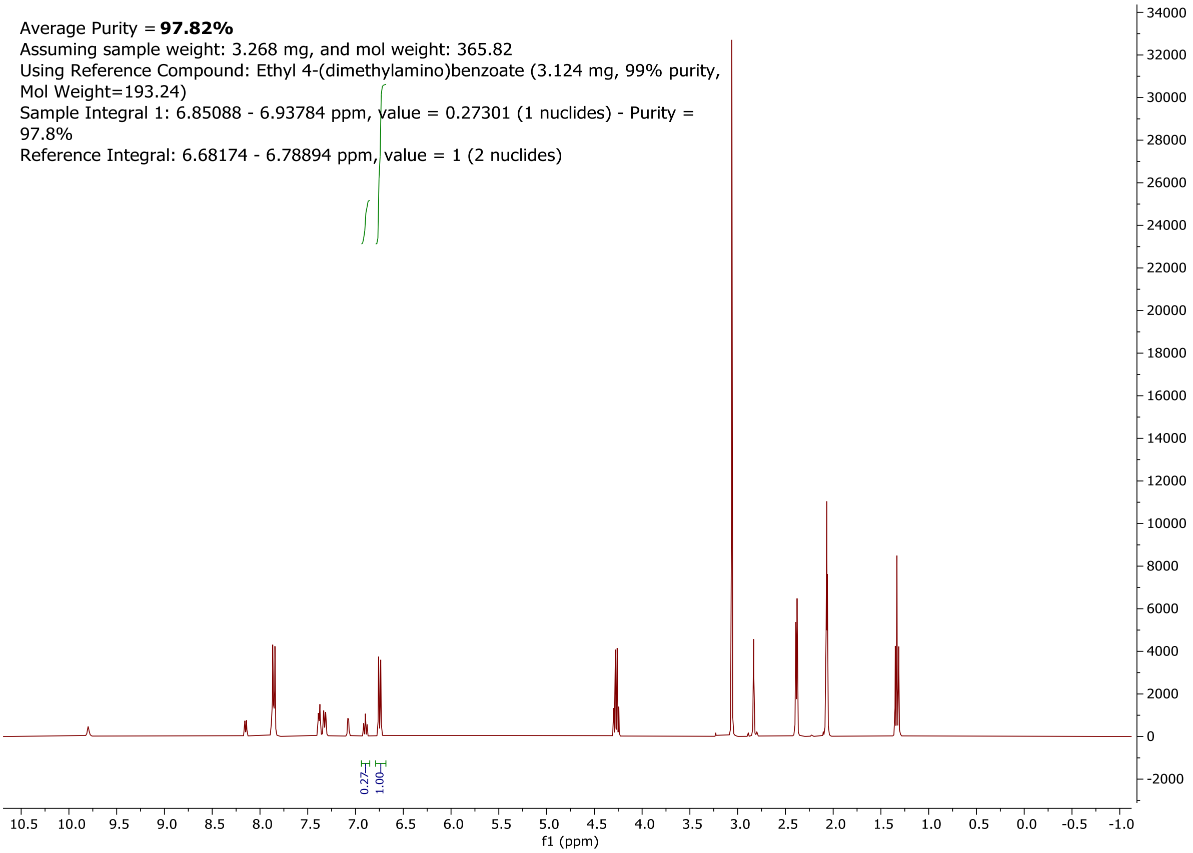

^{1}H spectrum of compound **33:**

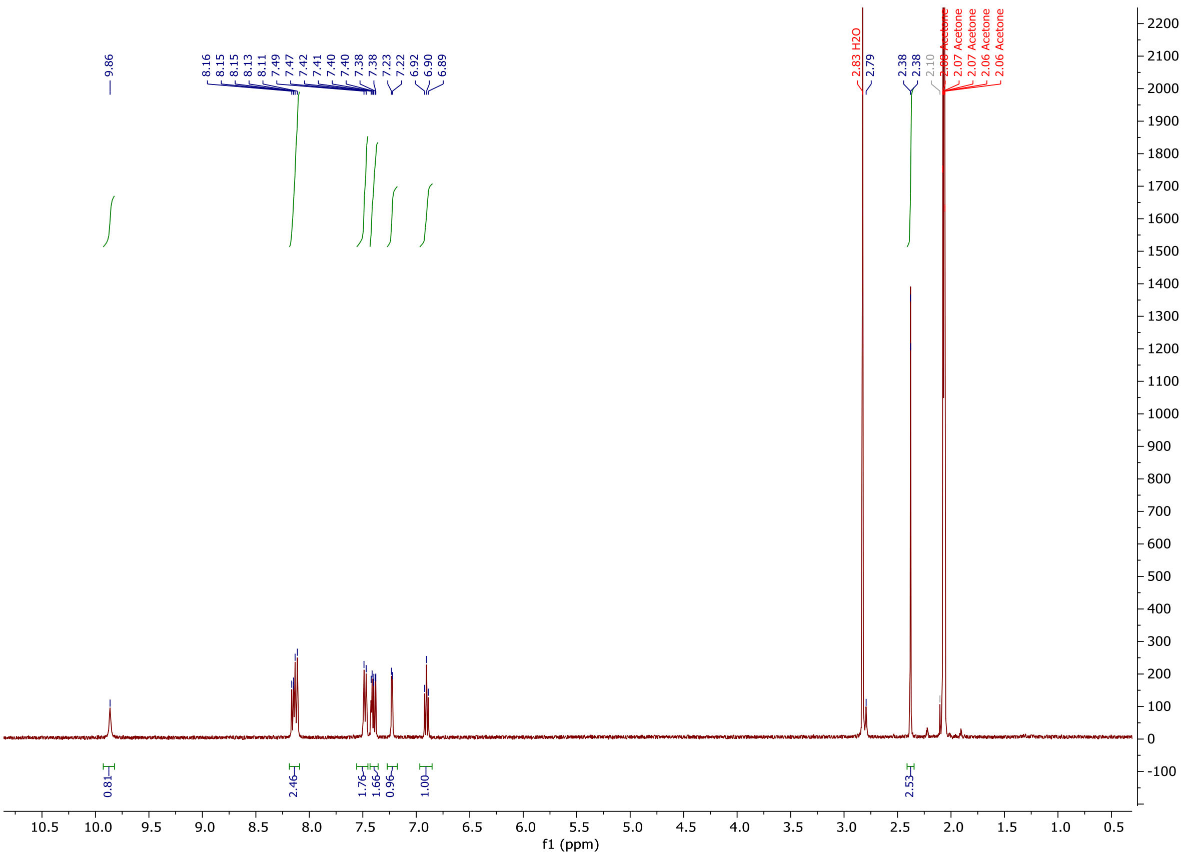

^{13}C spectrum of compound **33:**

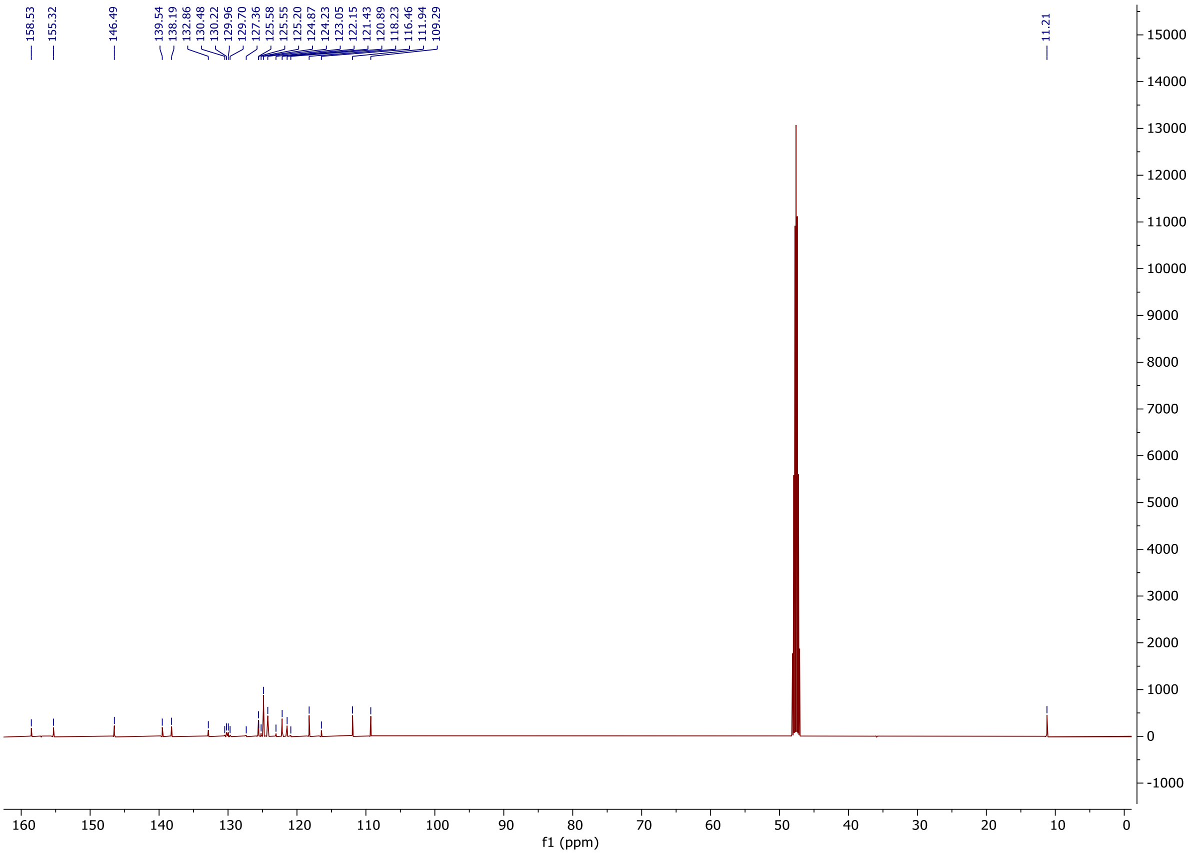

qHNMR spectrum of compound **33:**

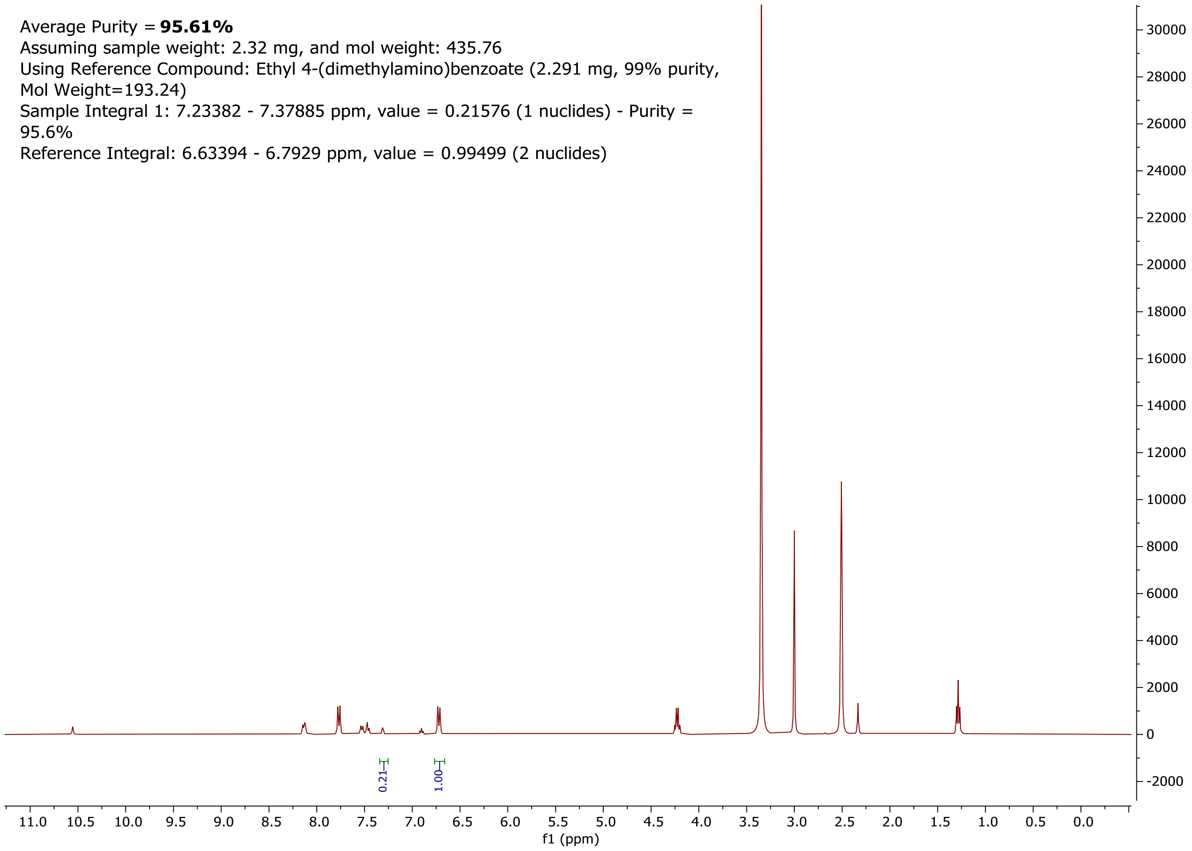

^{1}H spectrum of compound **34:**

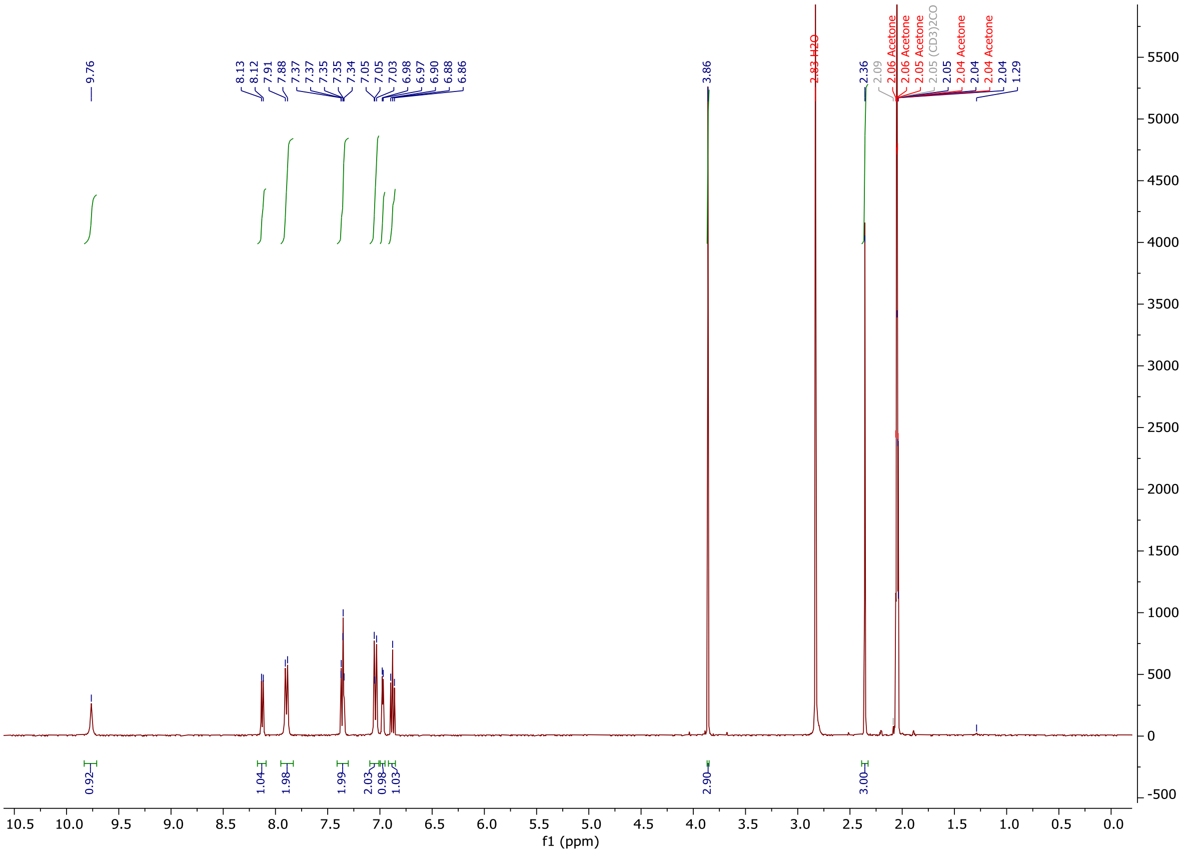

^{13}C spectrum of compound **34:**

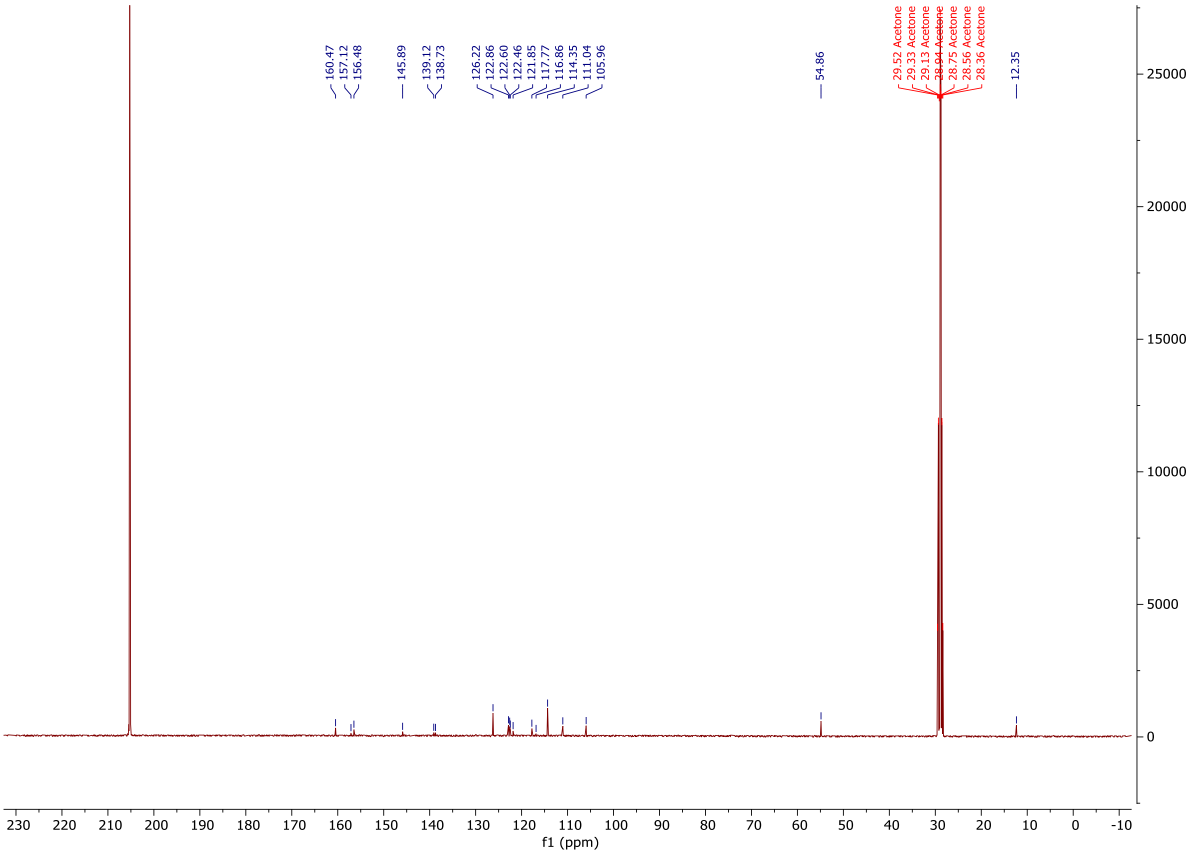

qHNMR spectrum of compound **34:**

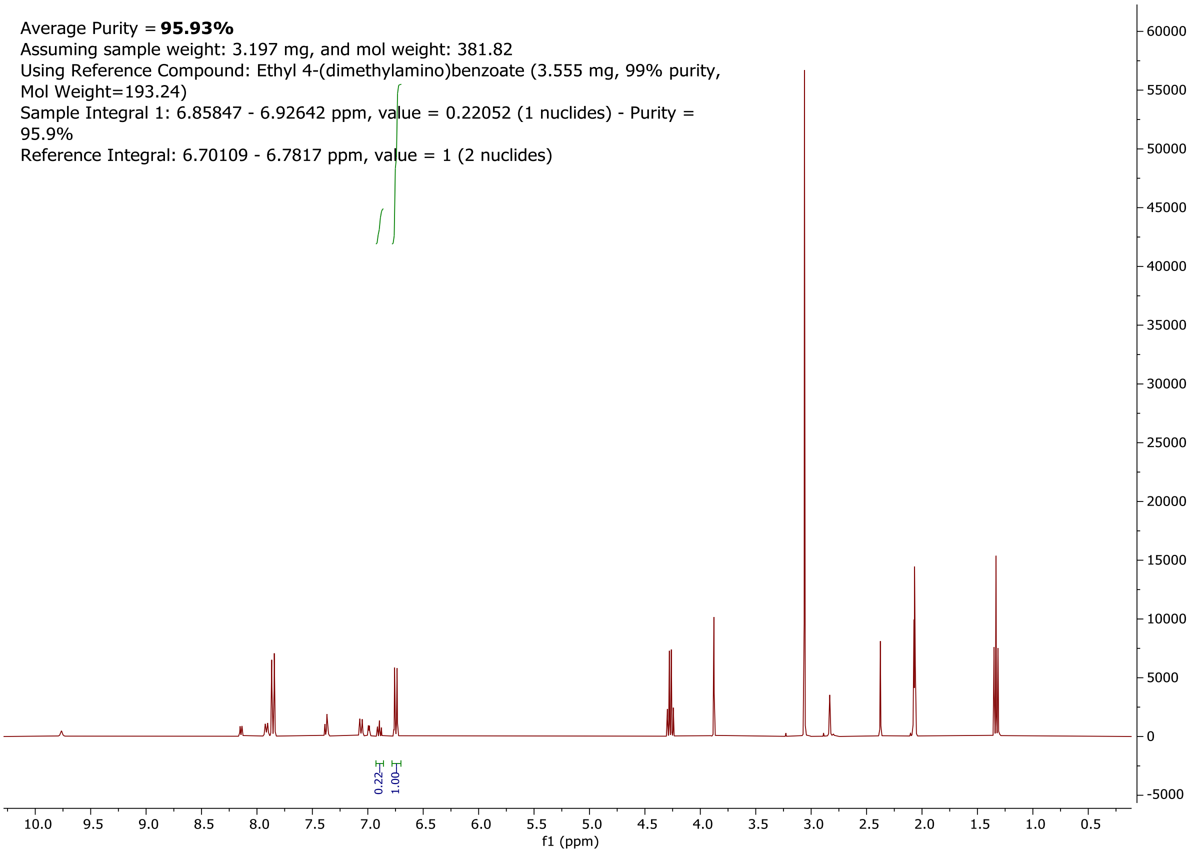

^{1}H spectrum of compound **35:**

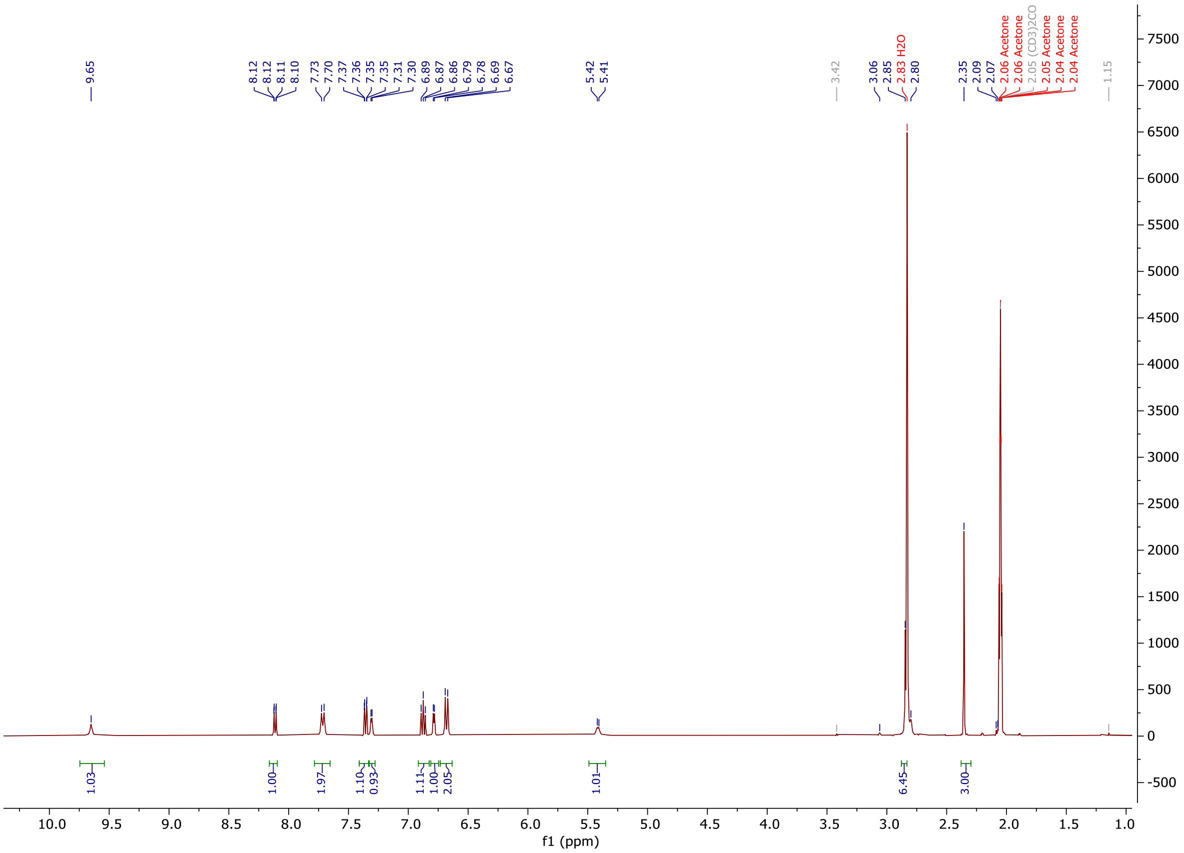

^{13}C spectrum of compound **35:**

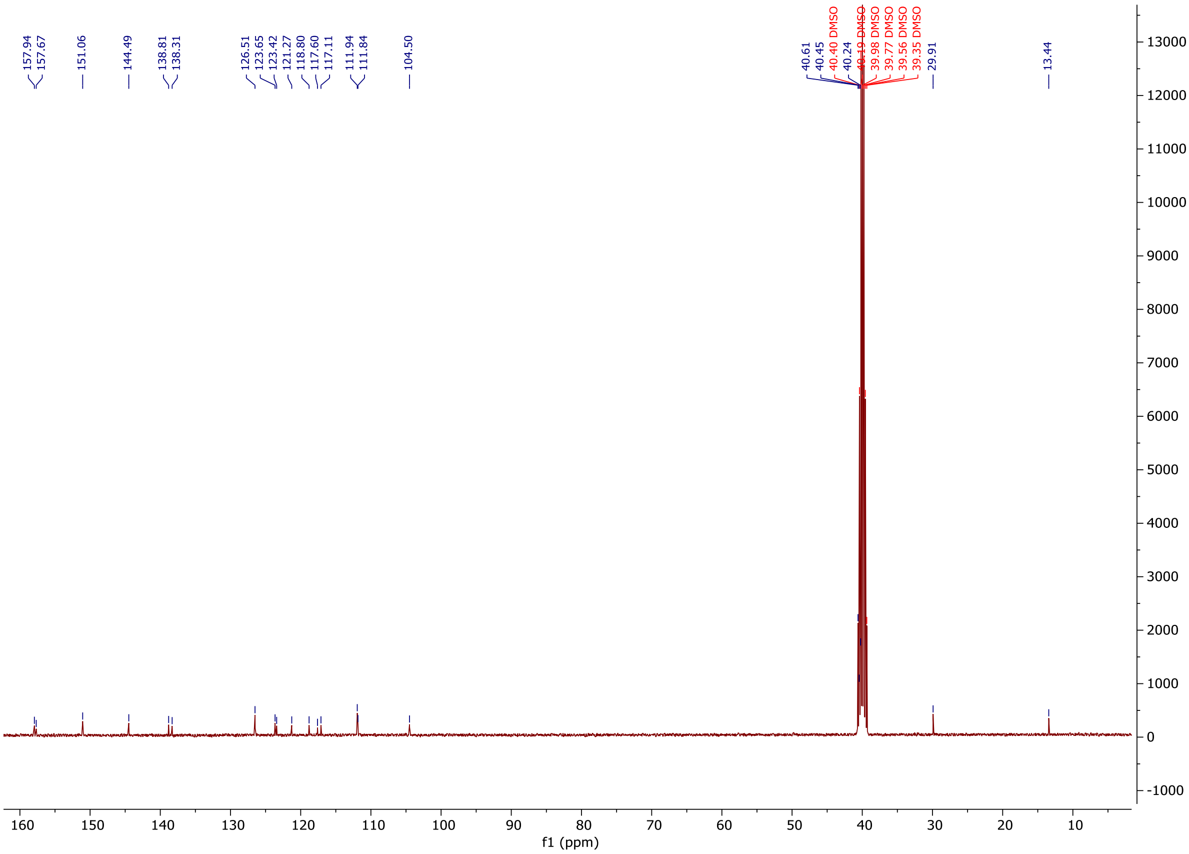

qHNMR spectrum of compound **35:**

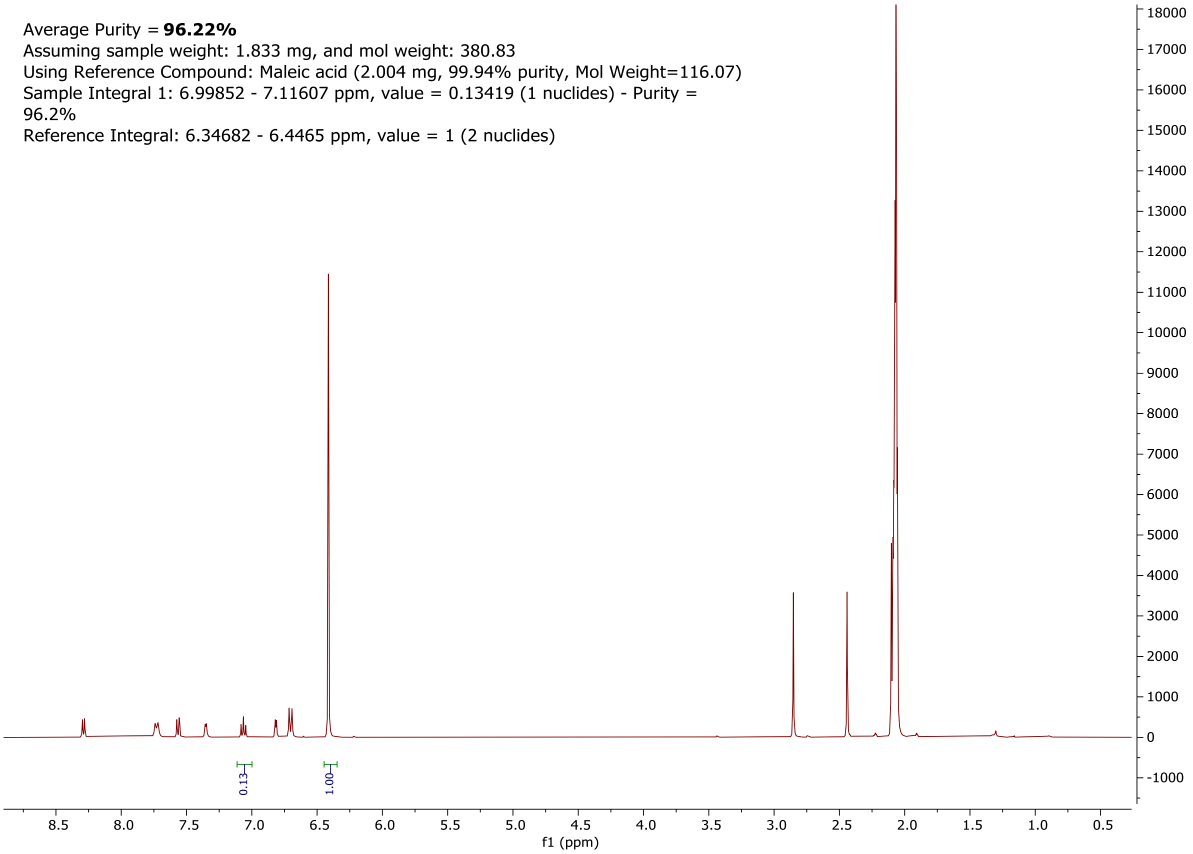

^{1}H spectrum of compound **36:**

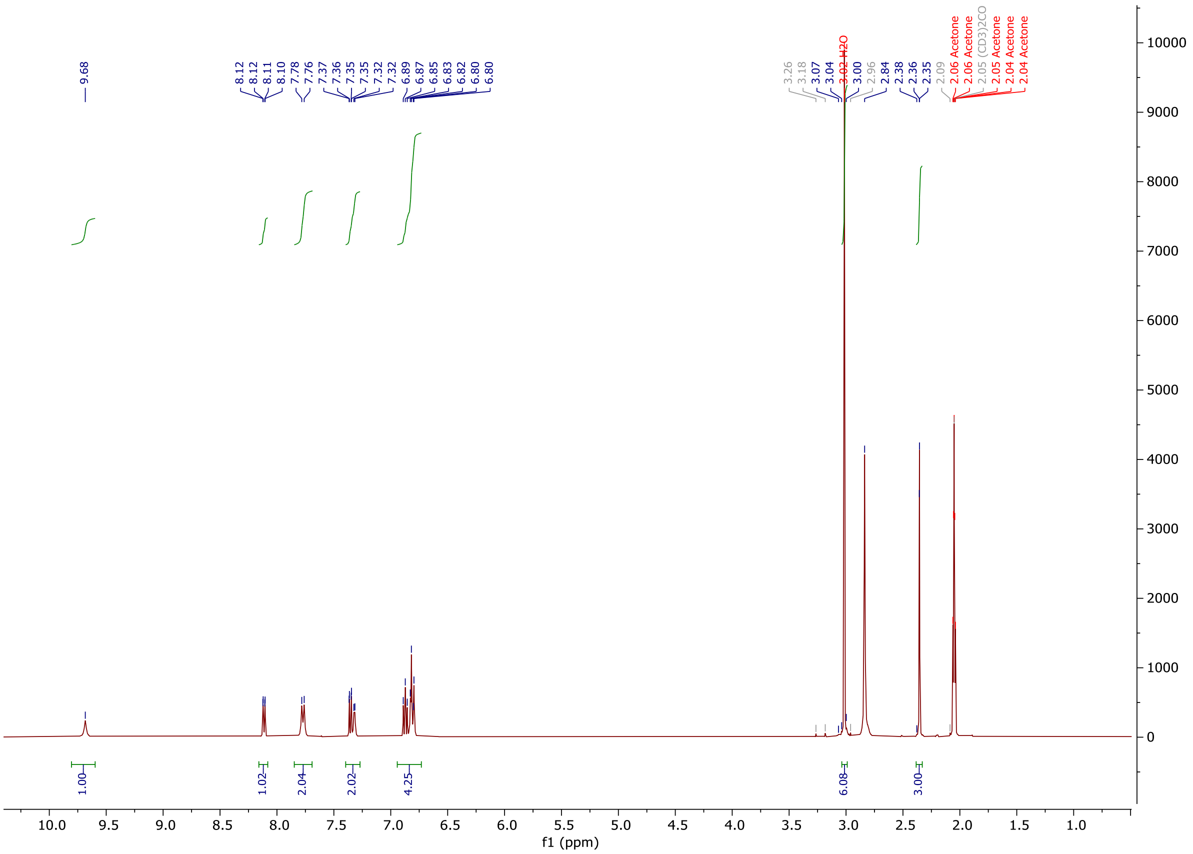

^{13}C spectrum of compound **36:**

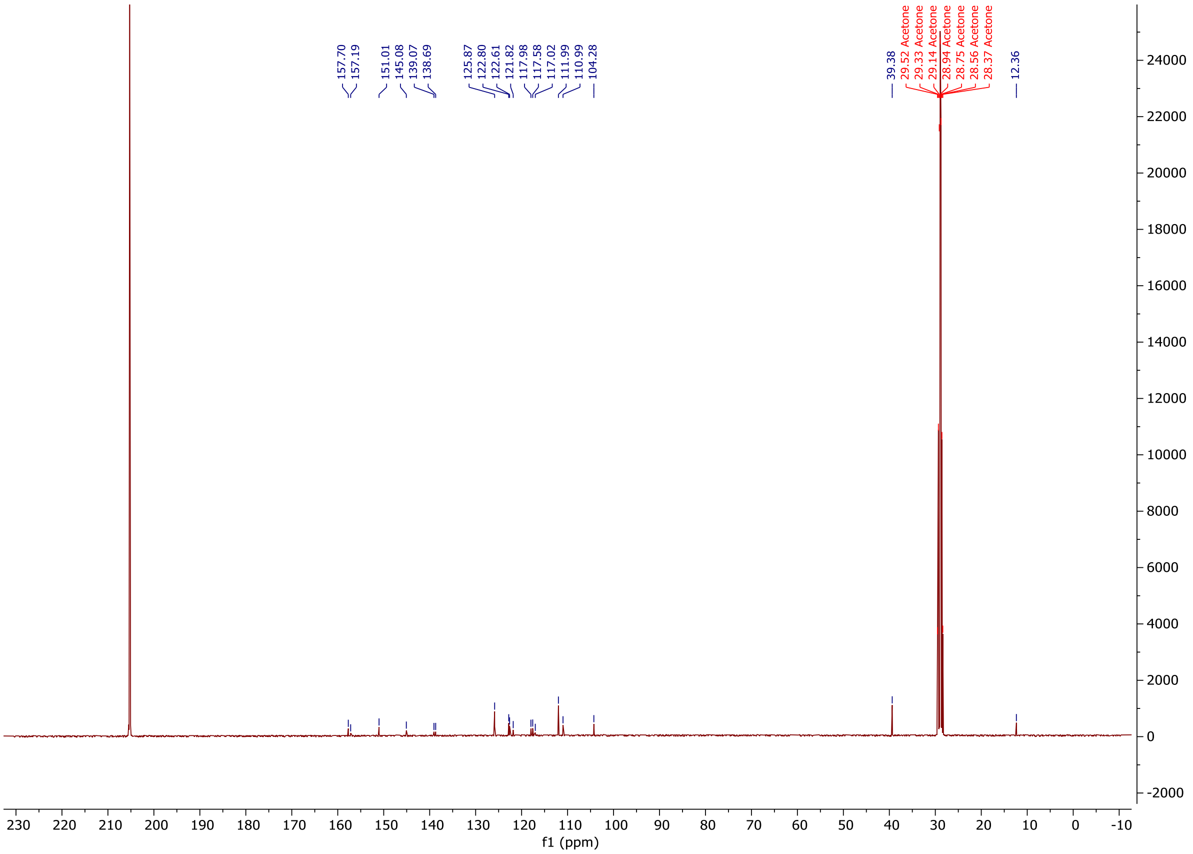

qHNMR spectrum of compound **36:**

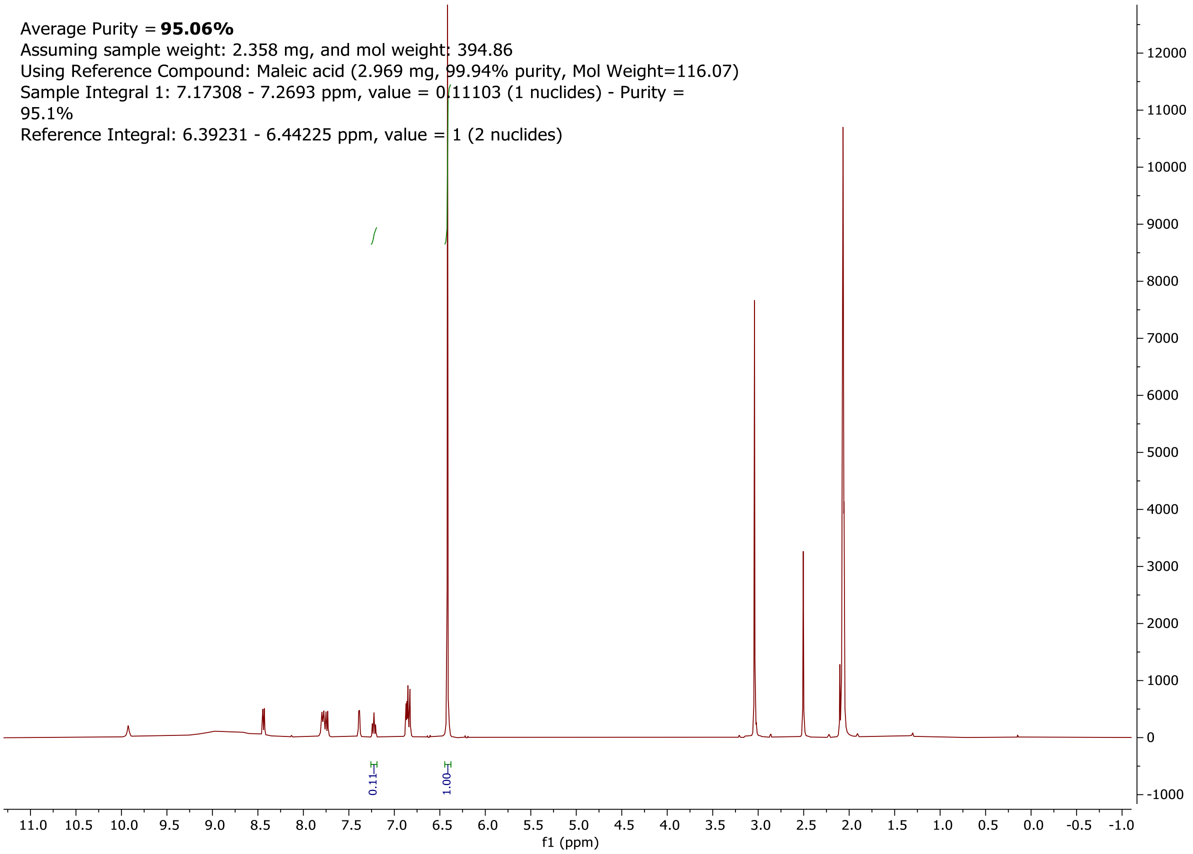

^{1}H spectrum of compound **37:**

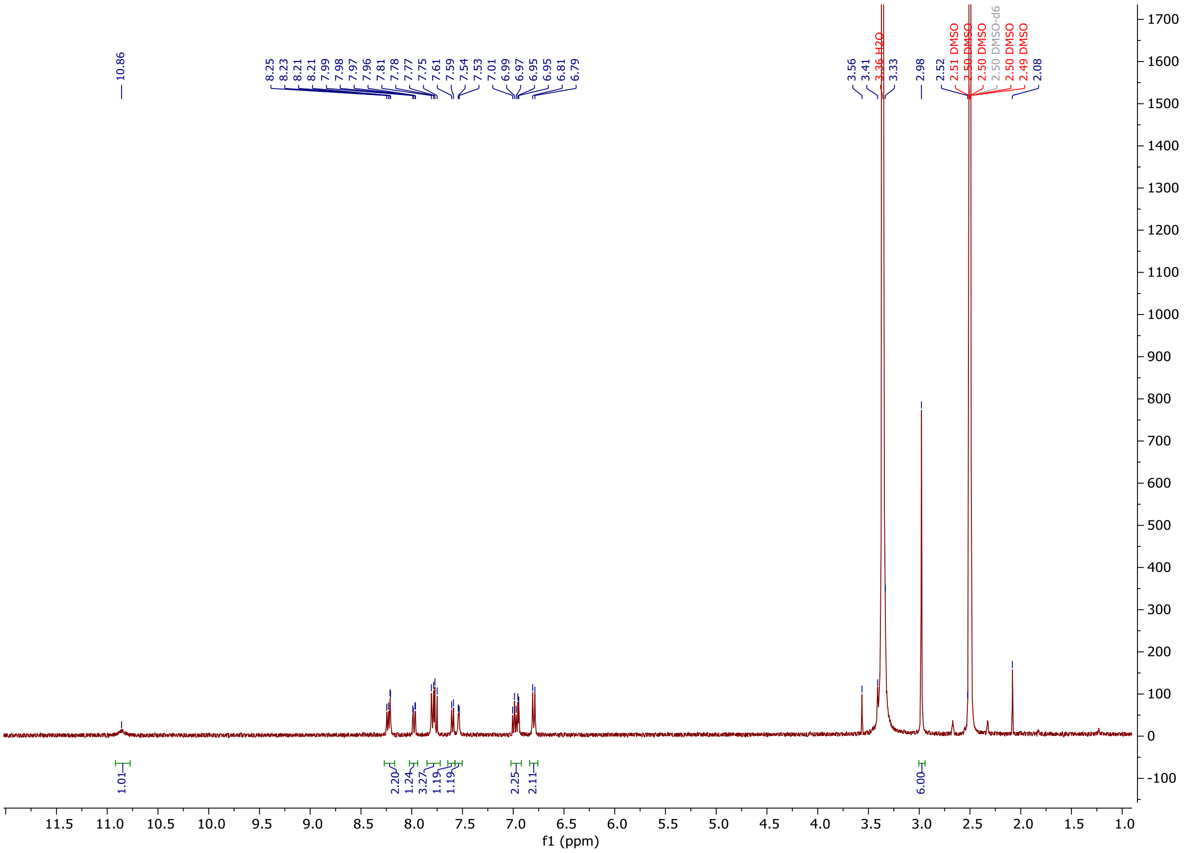

^{13}C spectrum of compound **37:**

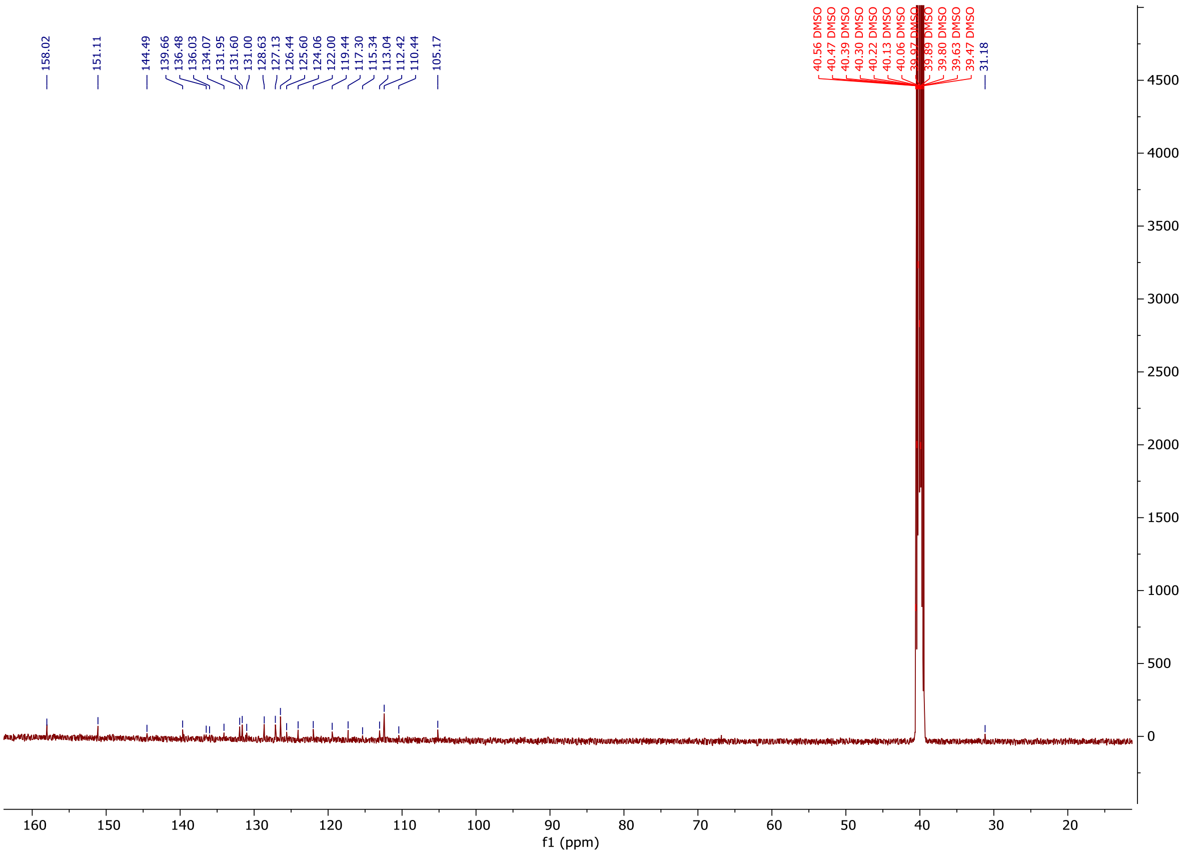

qHNMR spectrum of compound **37:**

Average Purity = **96.16%**
Assuming sample weight: 1.1 mg, and mol weight: 525.82
Using Reference Compound: Maleic acid (7.368 mg, 99.94% purity, Mol Weight=116.07)
Sample Integral 1: 6.89722 - 7.07833 ppm, value = 1.90589 (2 nuclides) - Purity = 96.2%
Reference Integral: 6.18925 - 6.29462 ppm, value = 60.10516 (2 nuclides)

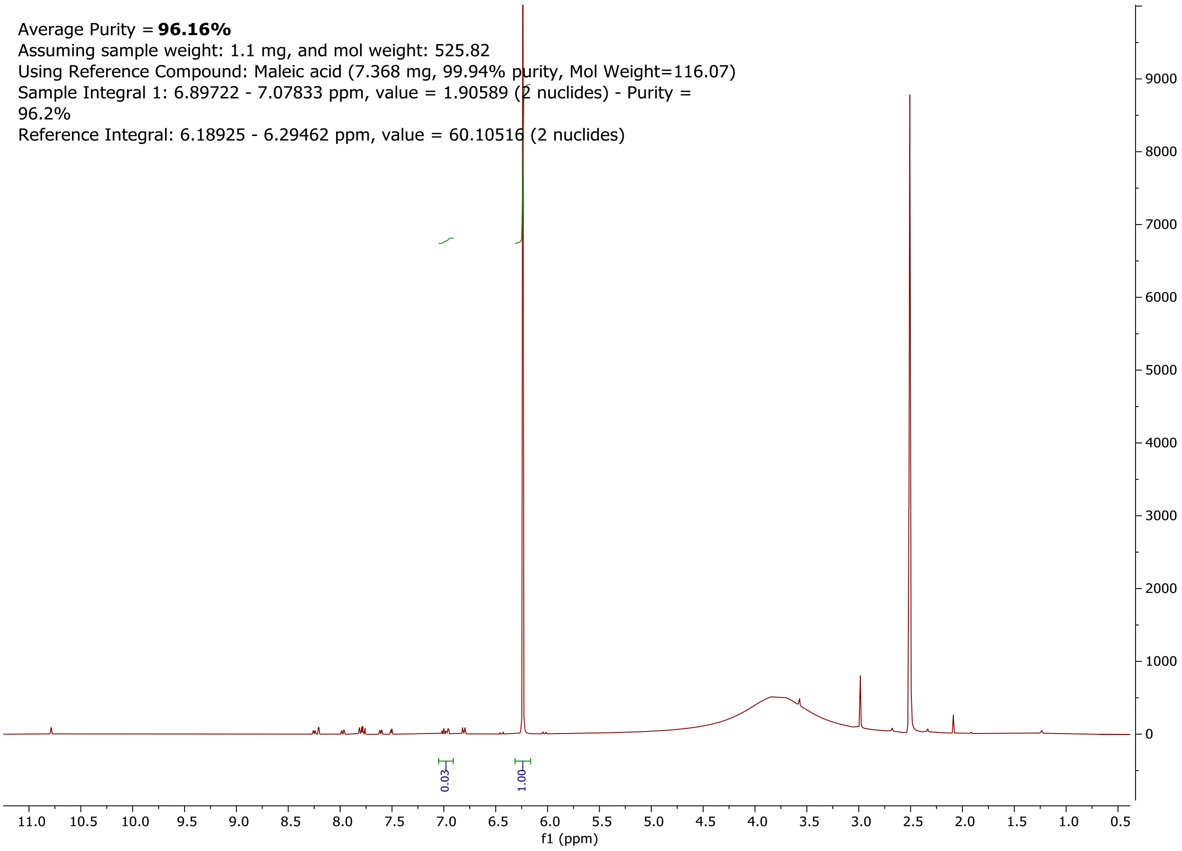

^{1}H spectrum of compound **38:**

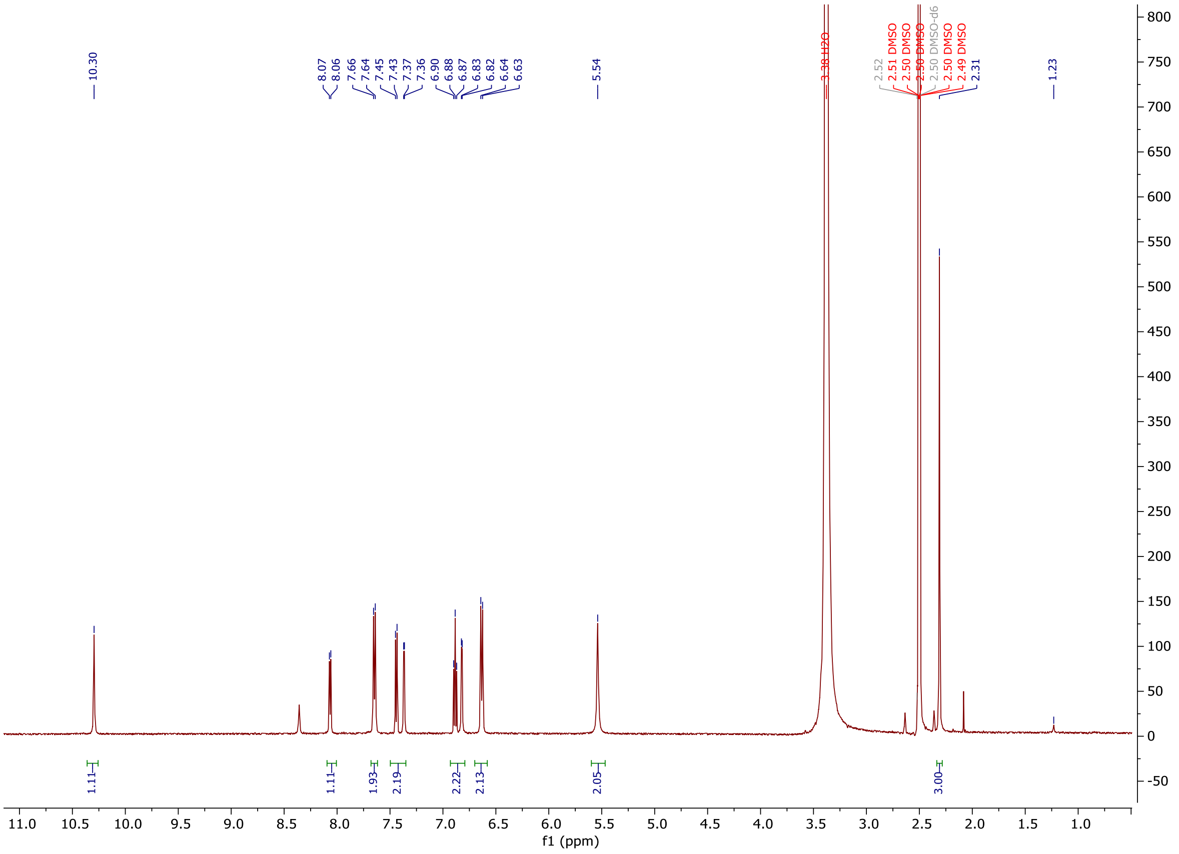

^{13}C spectrum of compound **38:**

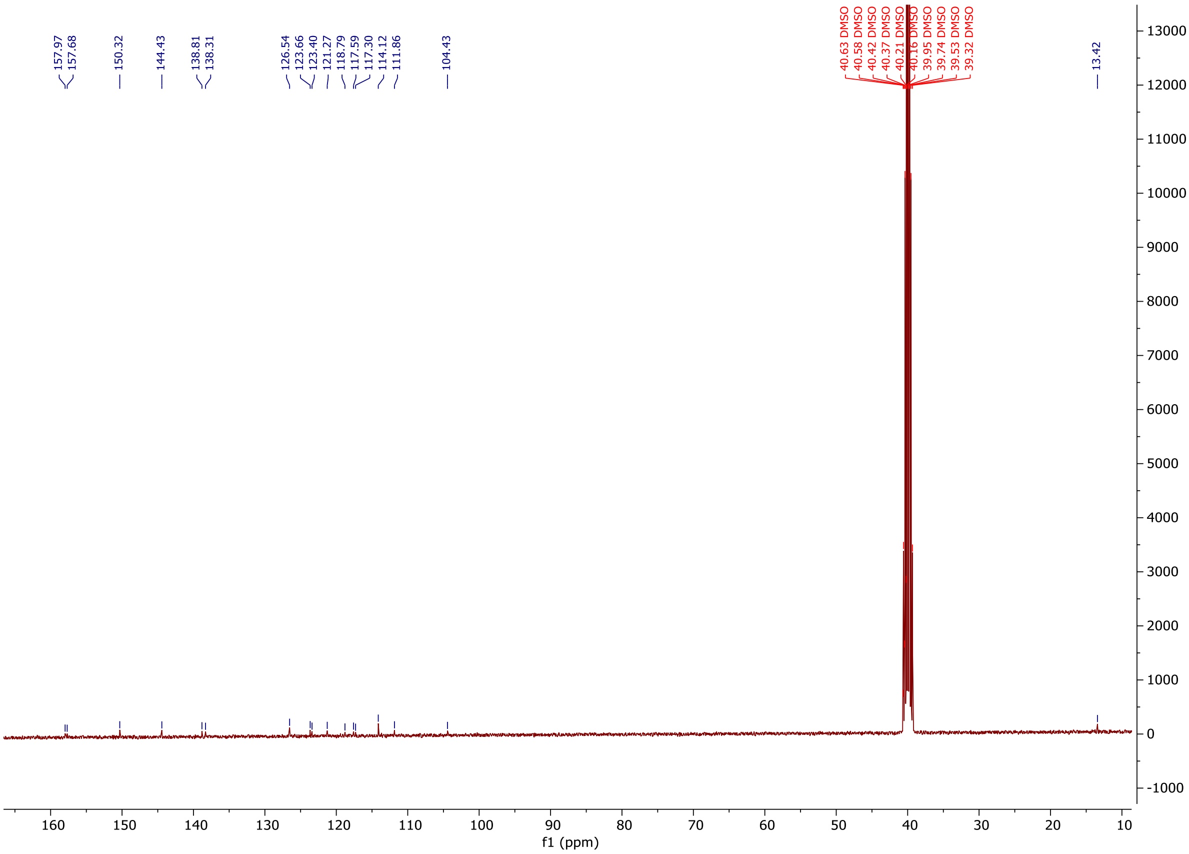

qHNMR spectrum of compound **38:**

Average Purity = **99.1%**
Assuming sample weight: 1.141 mg, and mol weight: 366.81
Using Reference Compound: Maleic acid (2.671 mg, 99.94% purity, Mol Weight=116.07)
Sample Integral 1: 6.5692 - 6.7022 ppm, value = 0.13469 (2 nuclides) - Purity = 99.1%
Reference Integral: 6.11434 - 6.26862 ppm, value = 1.00483 (2 nuclides)

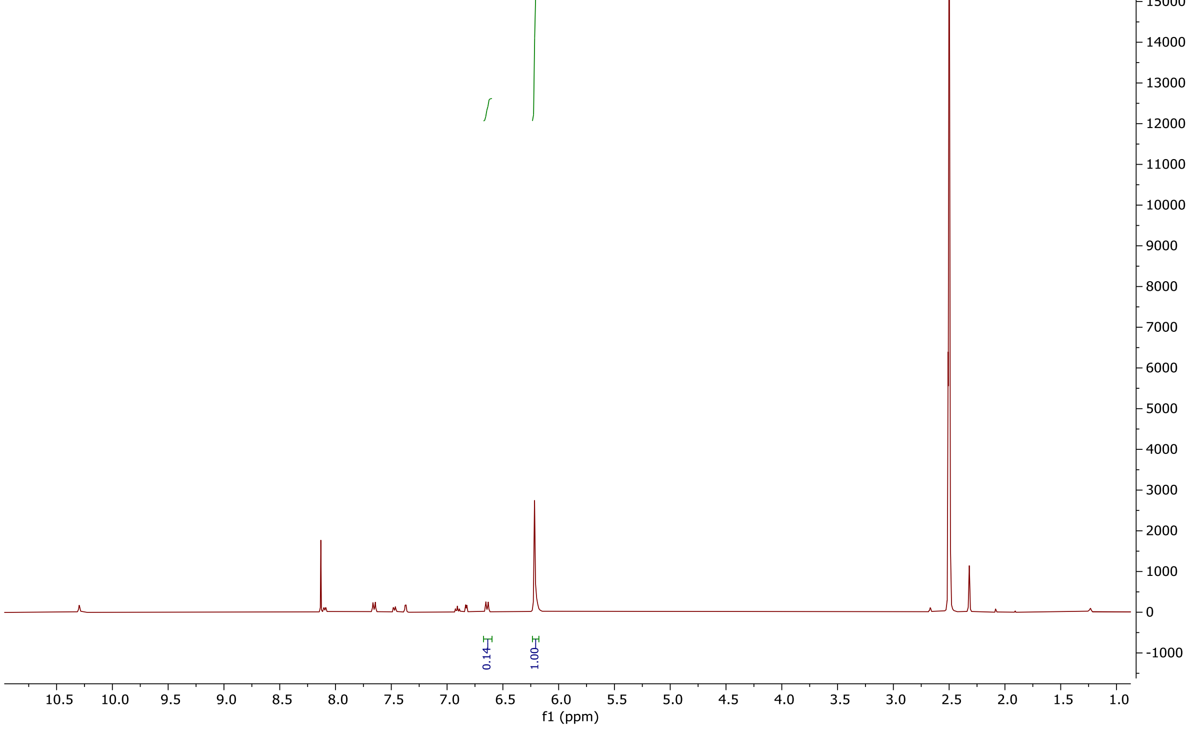

^{1}H spectrum of compound **39:**

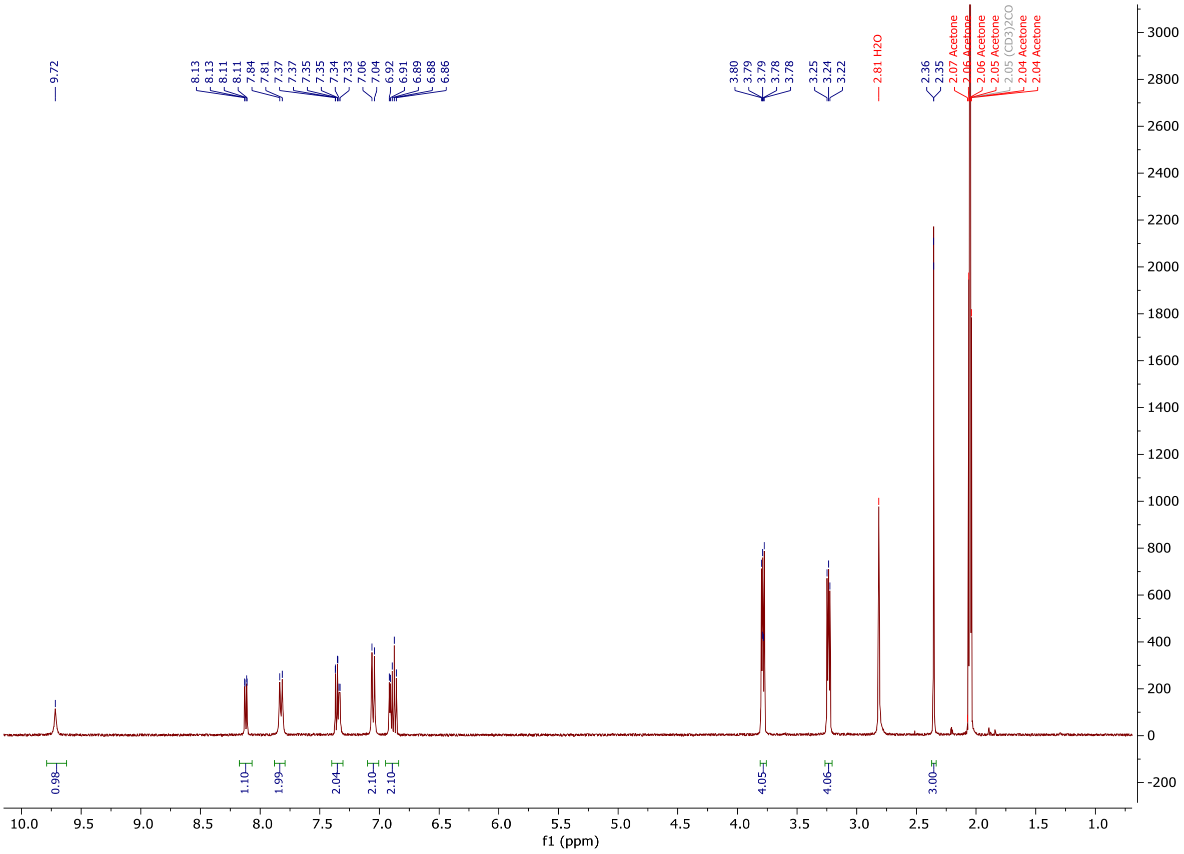

^{13}C spectrum of compound **39:**

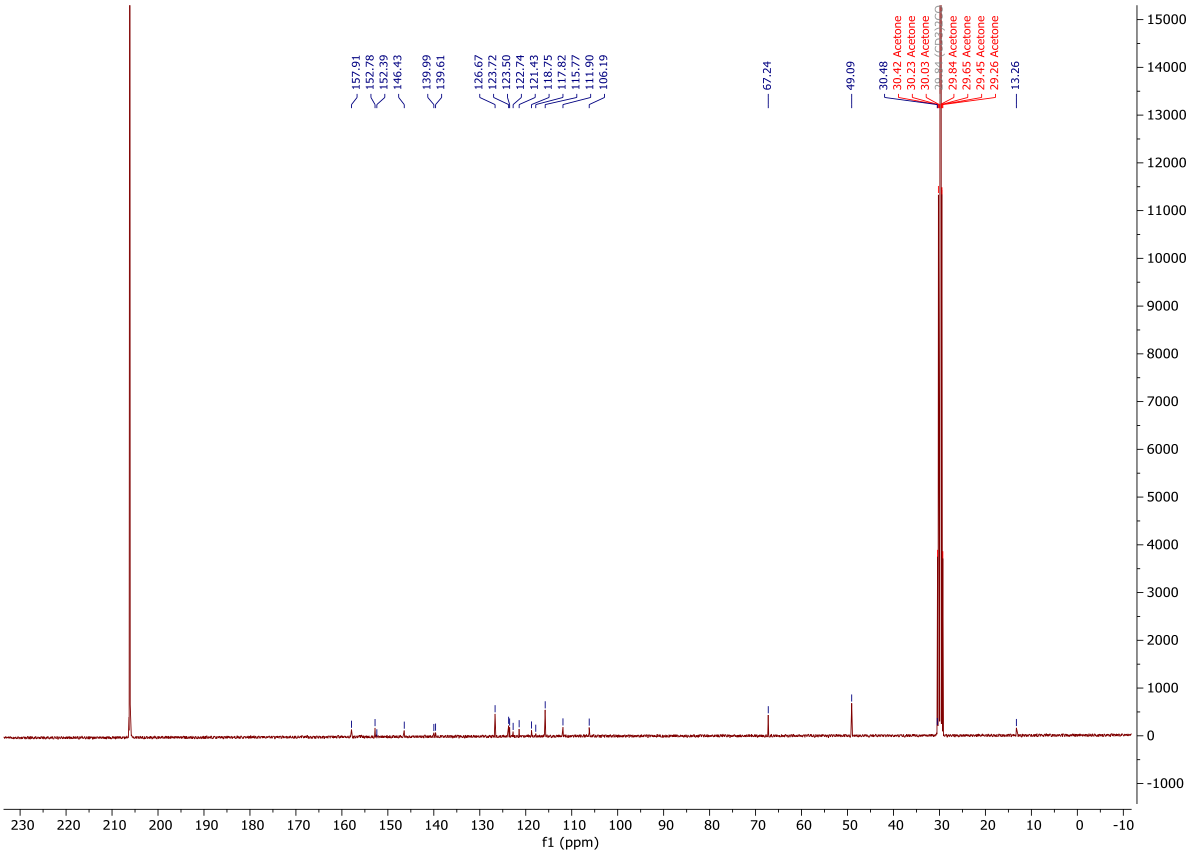

qHNMR spectrum of compound **39:**

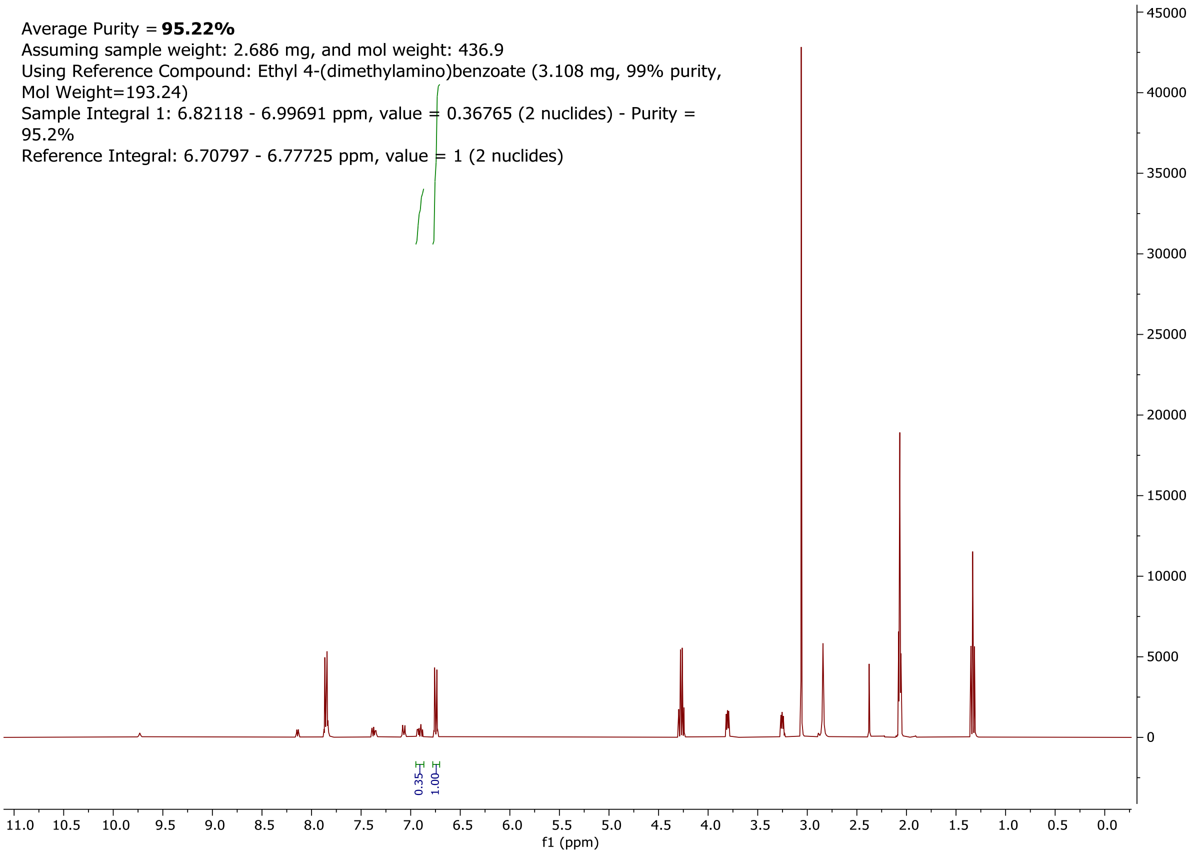

^{1}H spectrum of compound **40:**

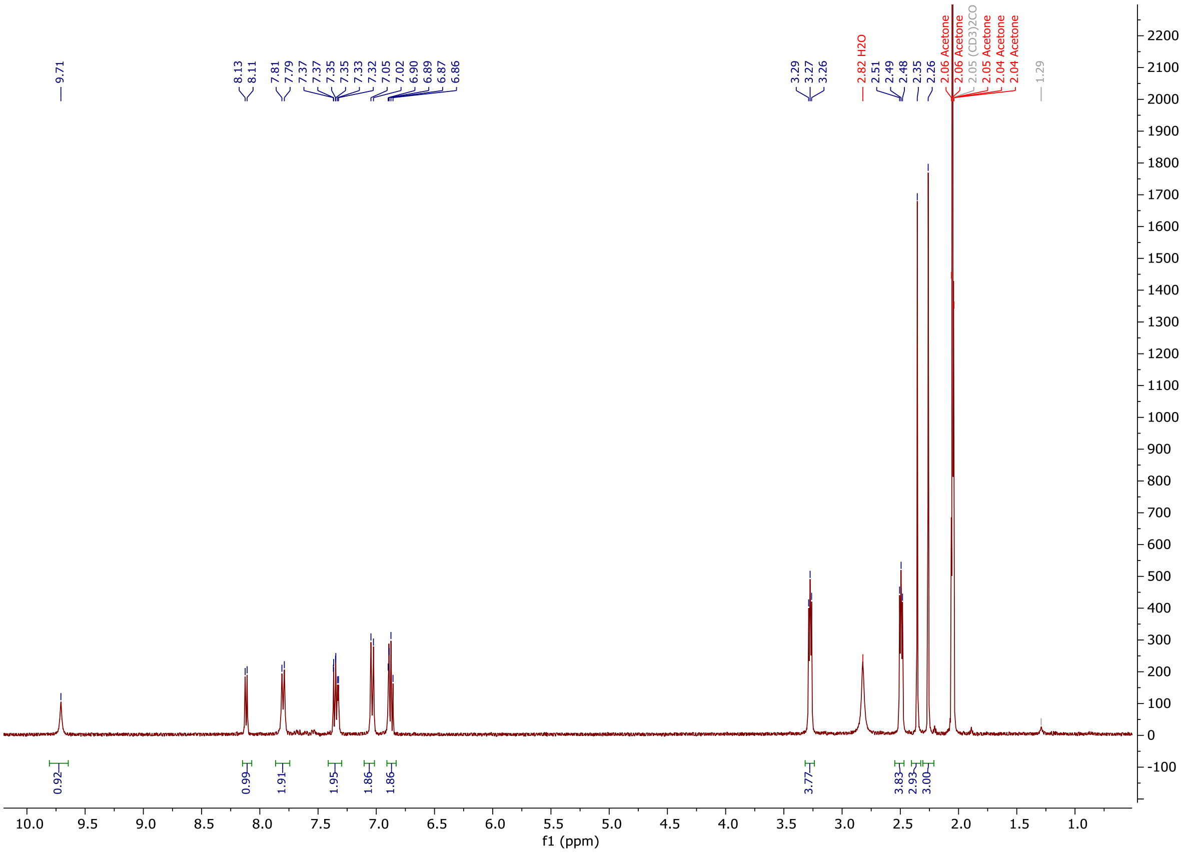

^{13}C spectrum of compound **40:**

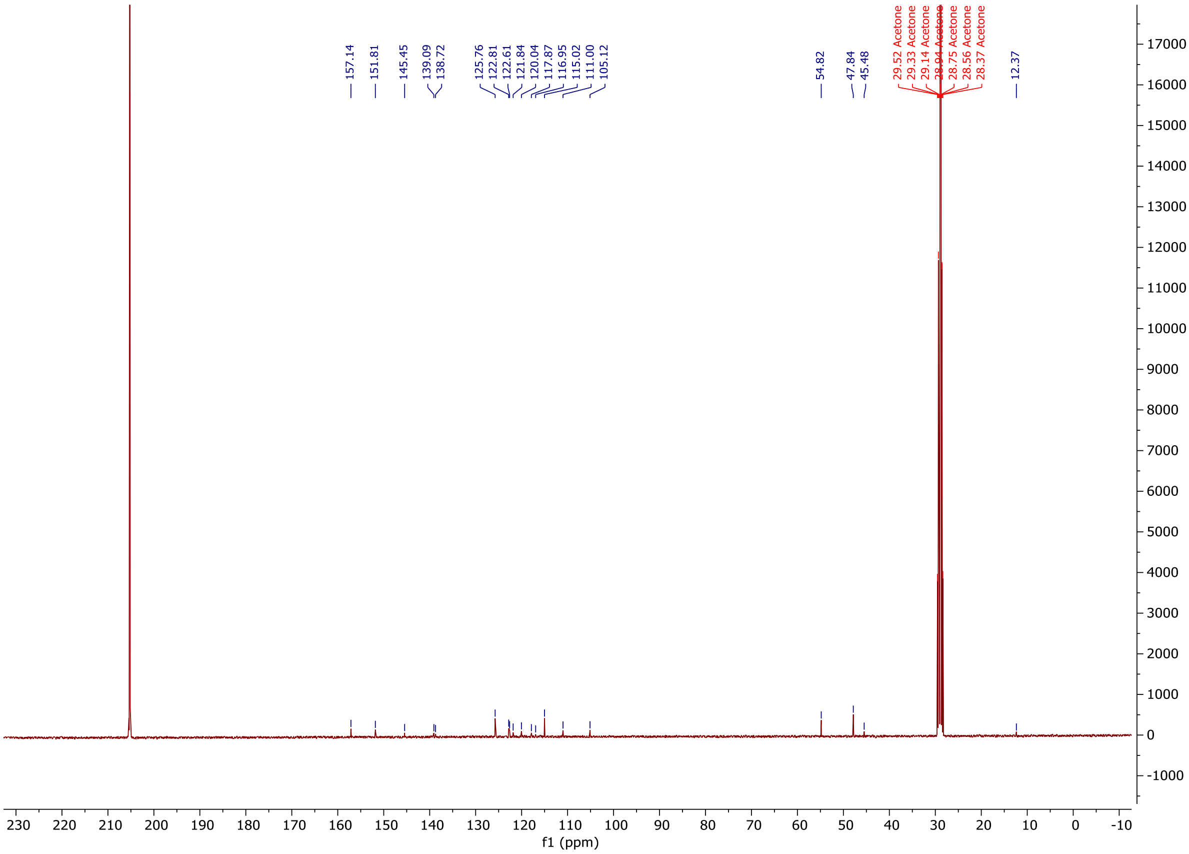

qHNMR spectrum of compound 40**:**

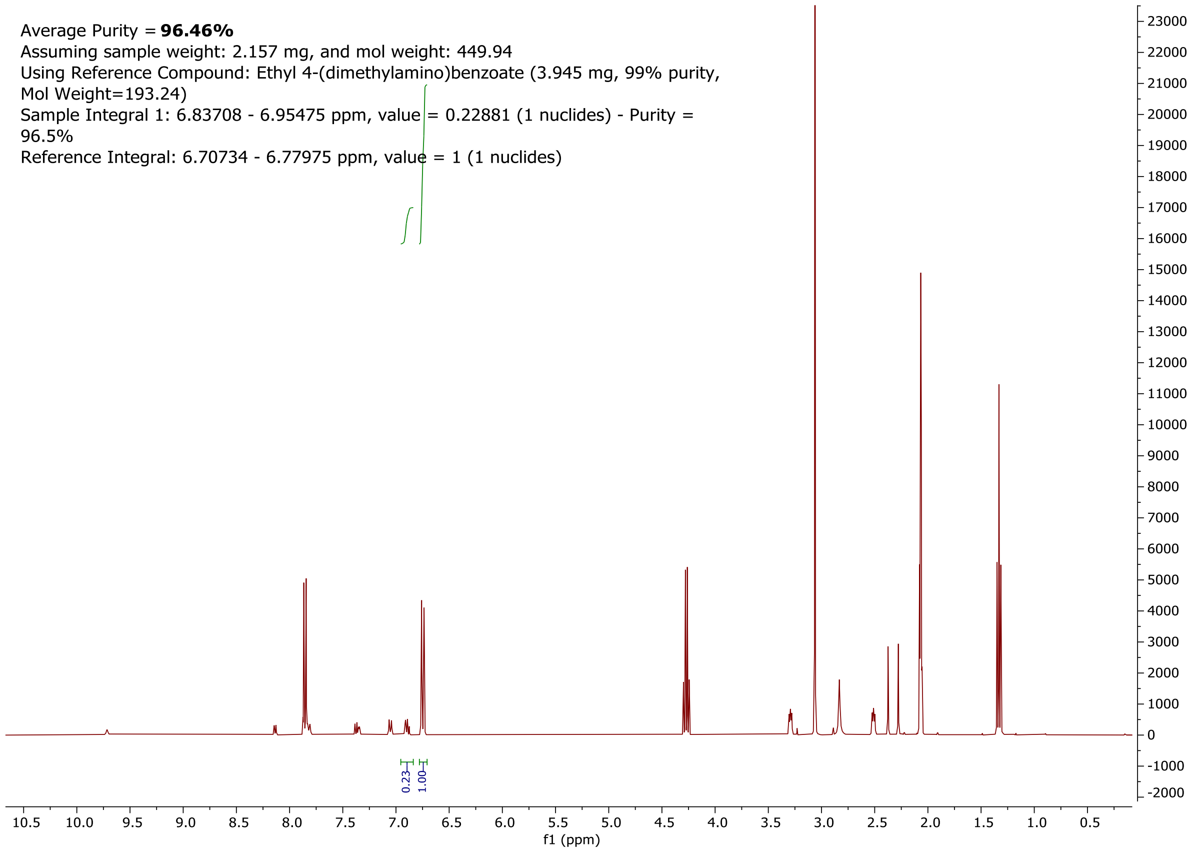

^{1}H spectrum of compound **41:**

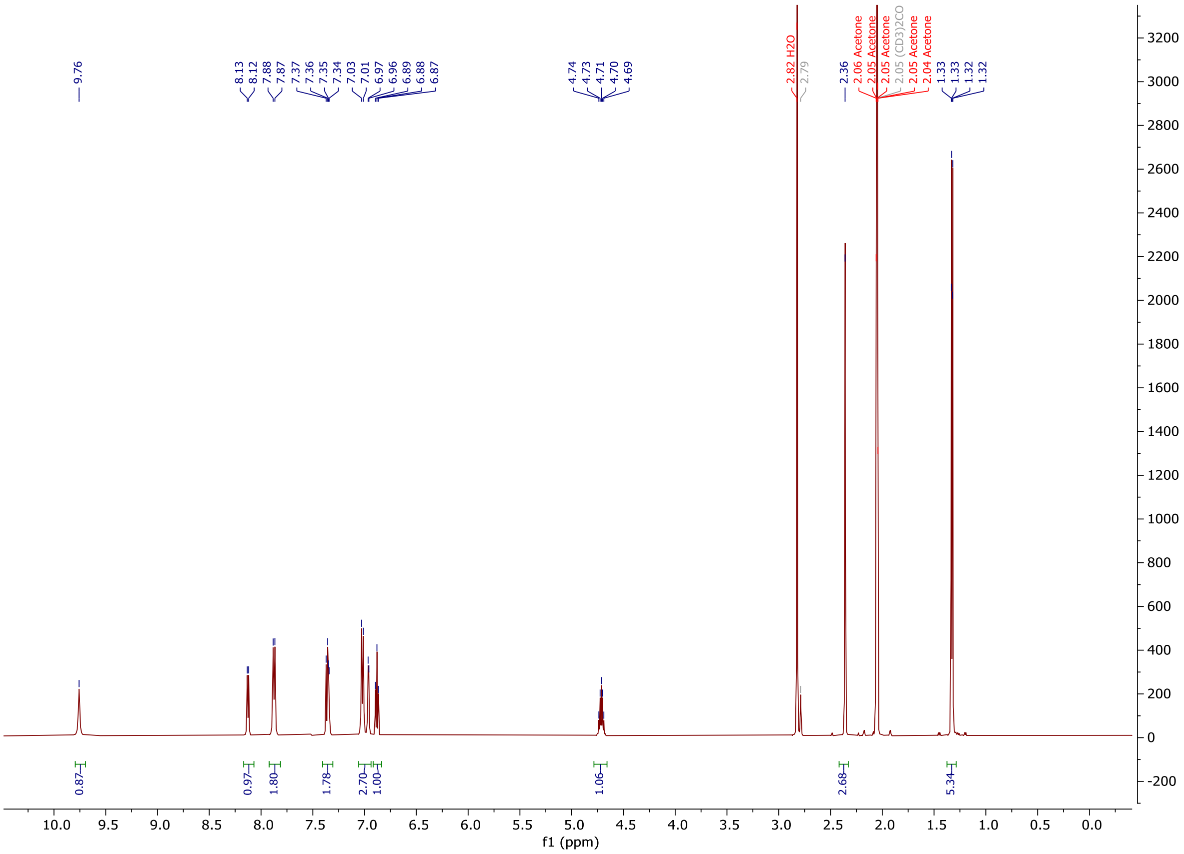

^{13}C spectrum of compound **41:**

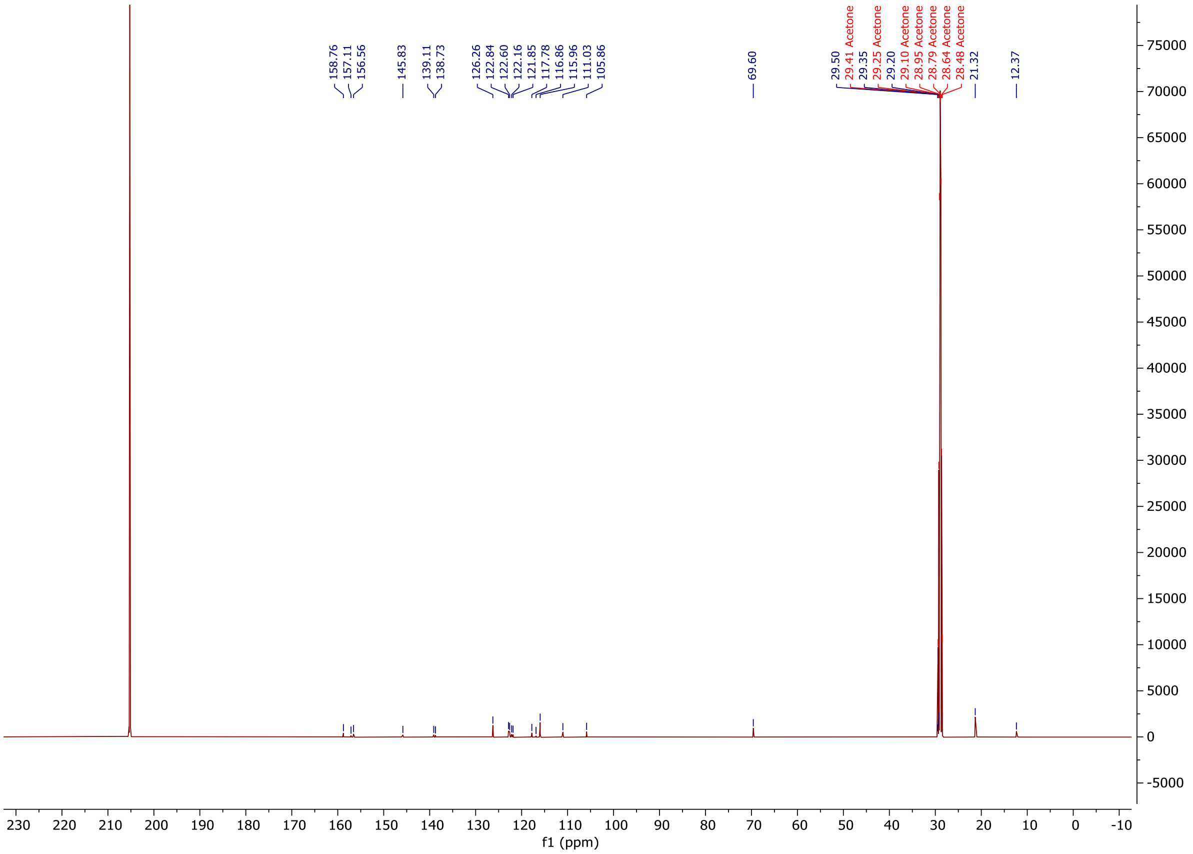

qHNMR spectrum of compound **41:**

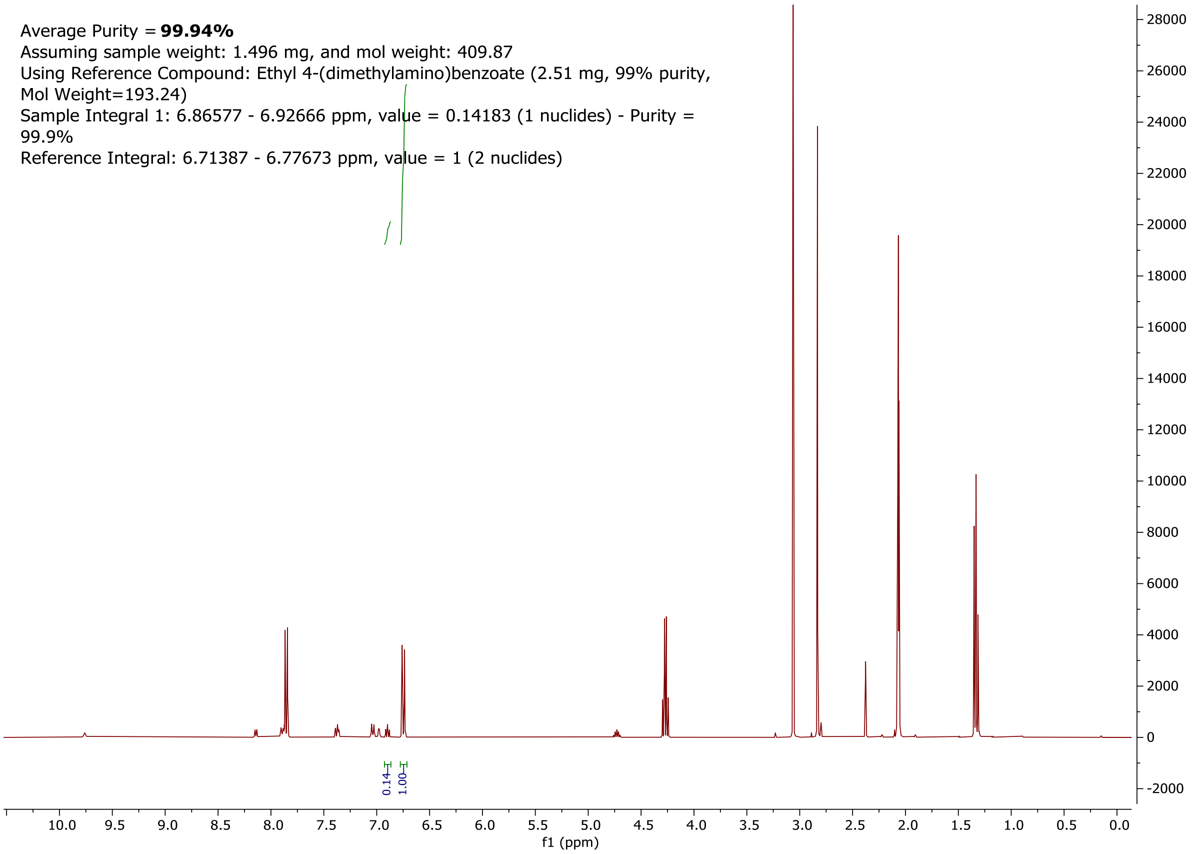

^{1}H spectrum of compound **42:**

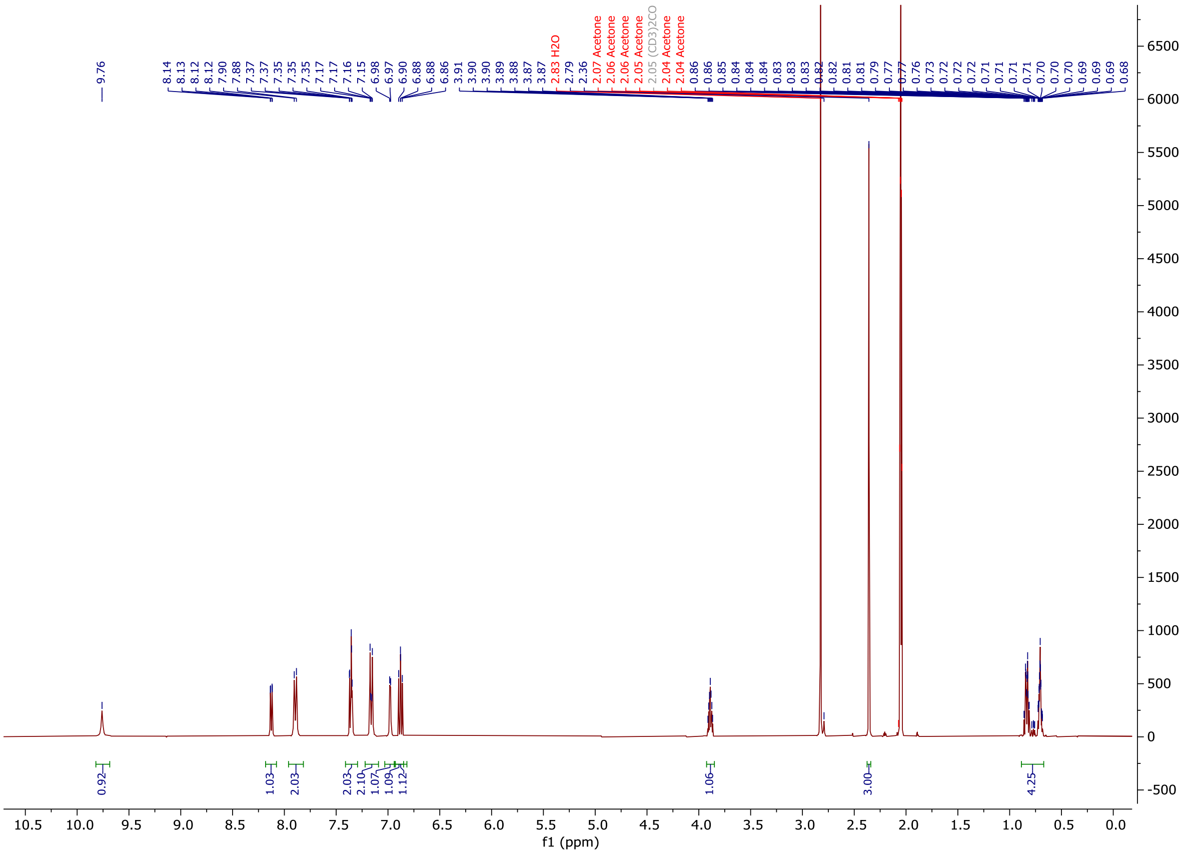

^{13}C spectrum of compound **42:**

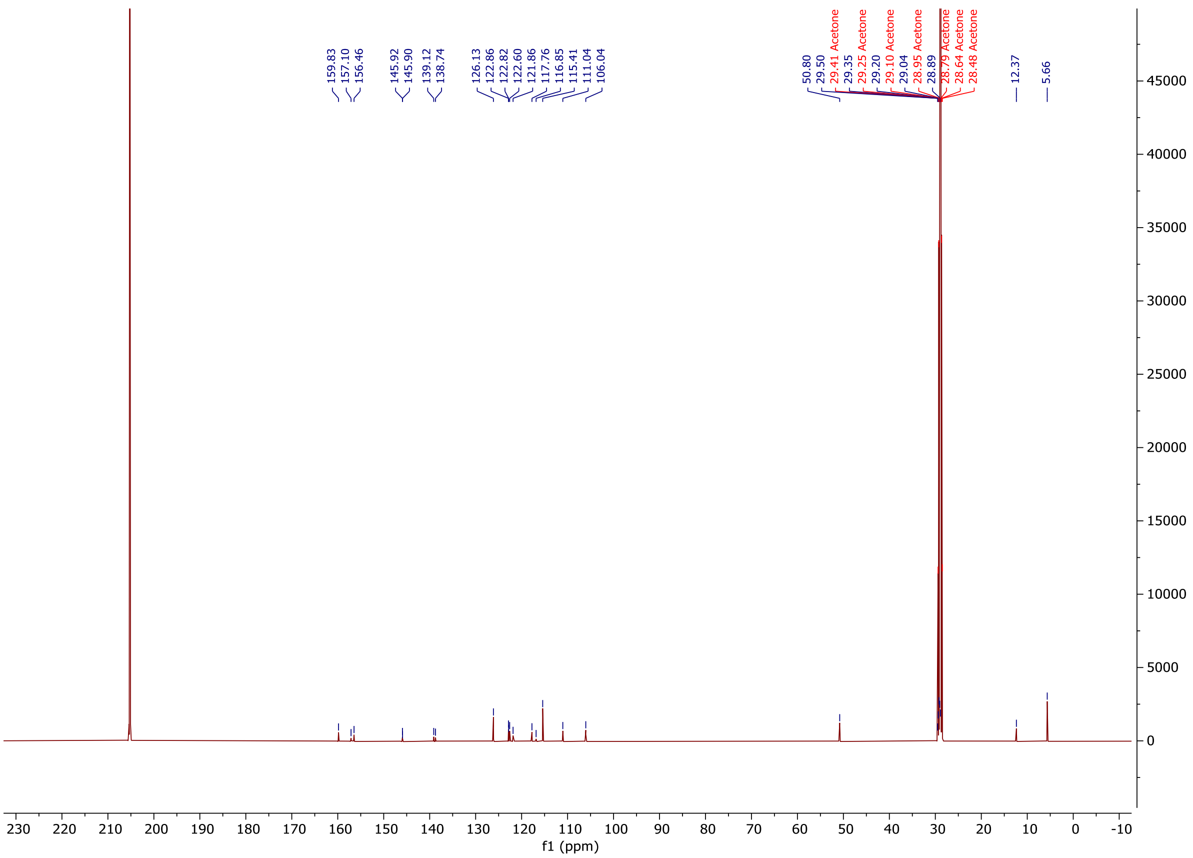

qHNMR spectrum of compound **42:**

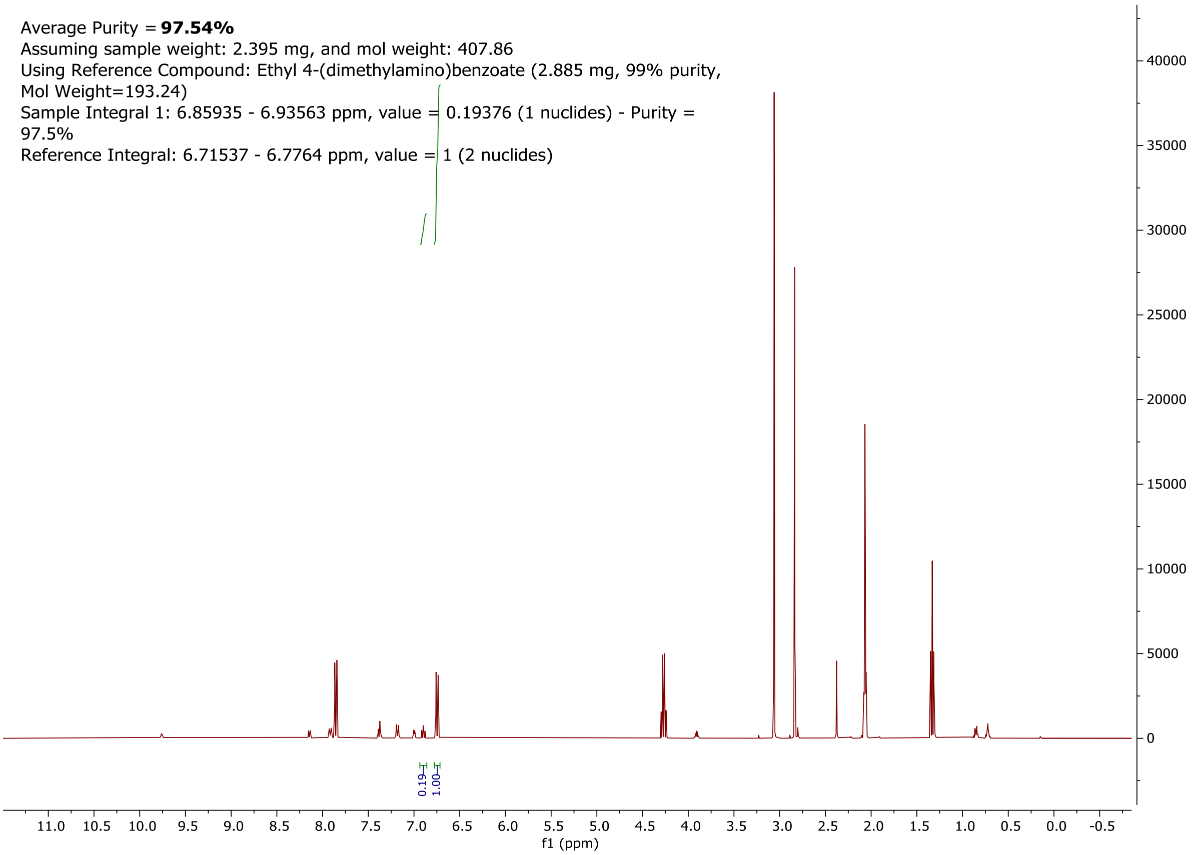

HRMS spectra of **8-9, 12** and **14-42**

HRMS spectrum of compound **8:**

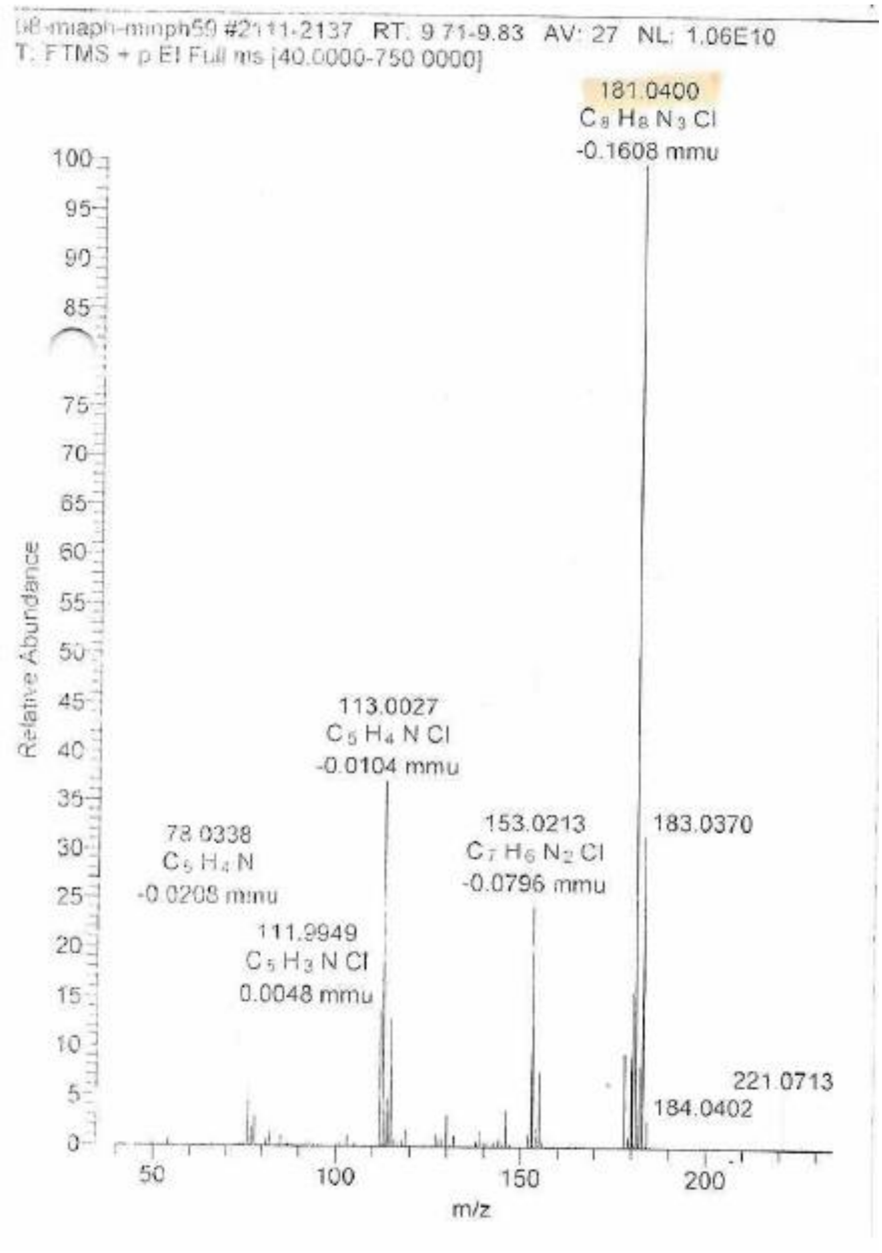

HRMS spectrum of compound **9:**

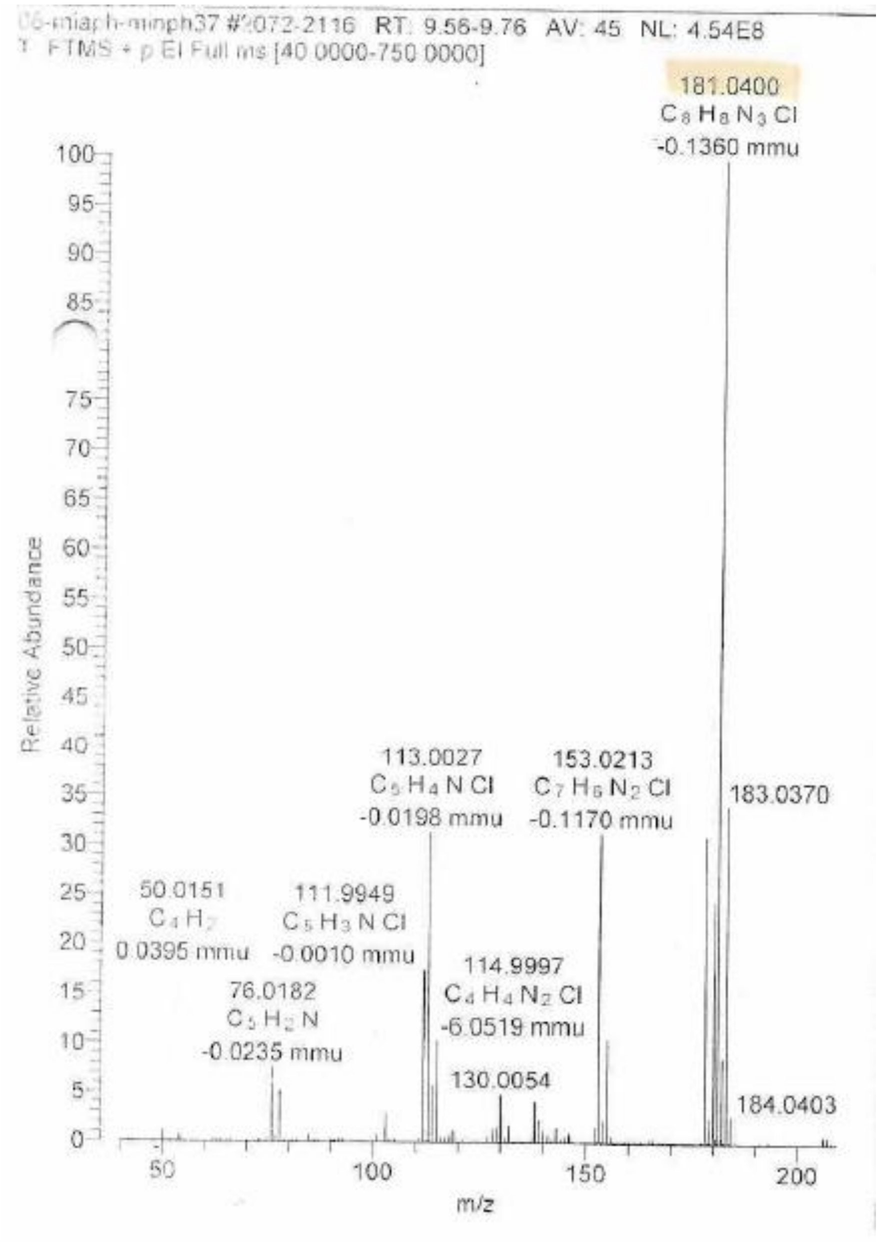

HRMS spectrum of compound **12:**

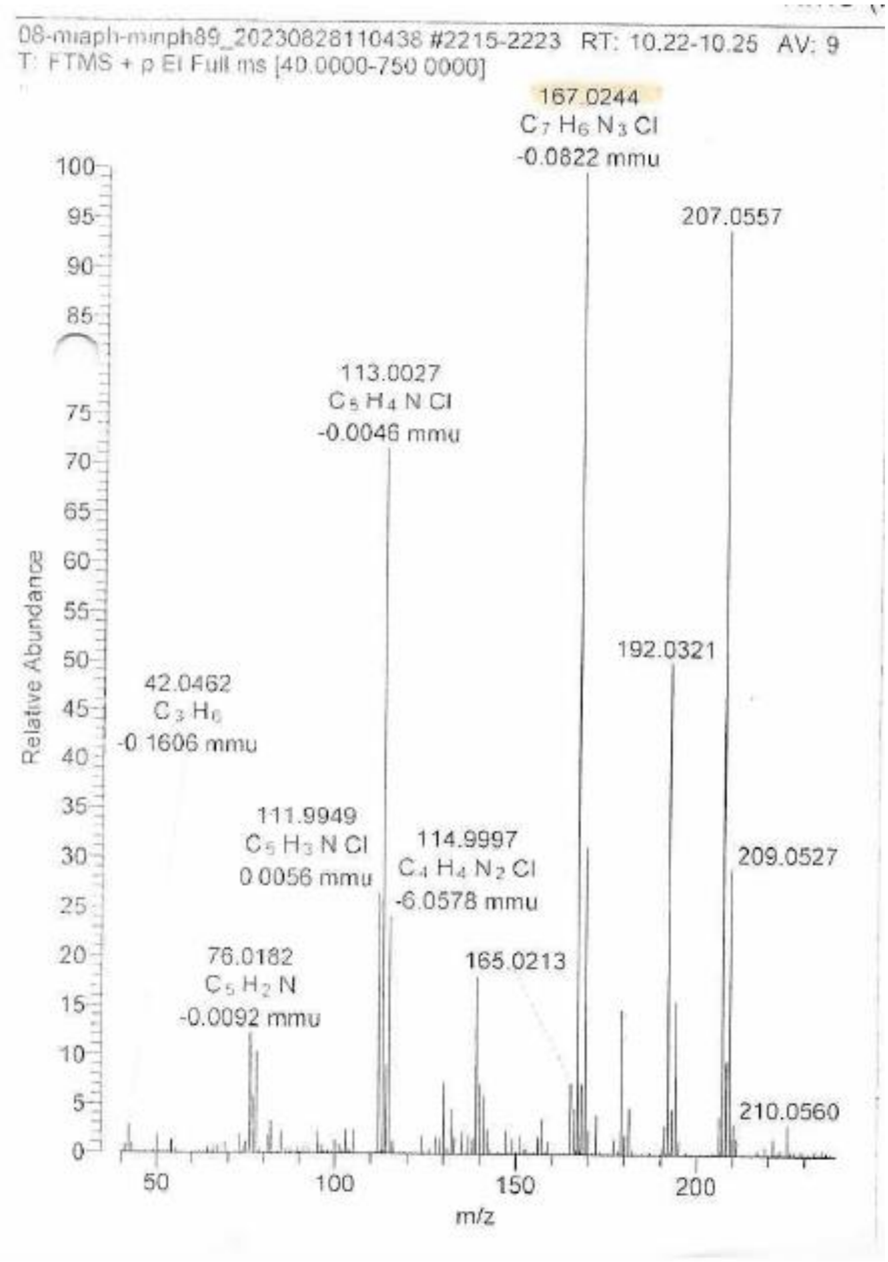

HRMS spectrum of compound **14:**

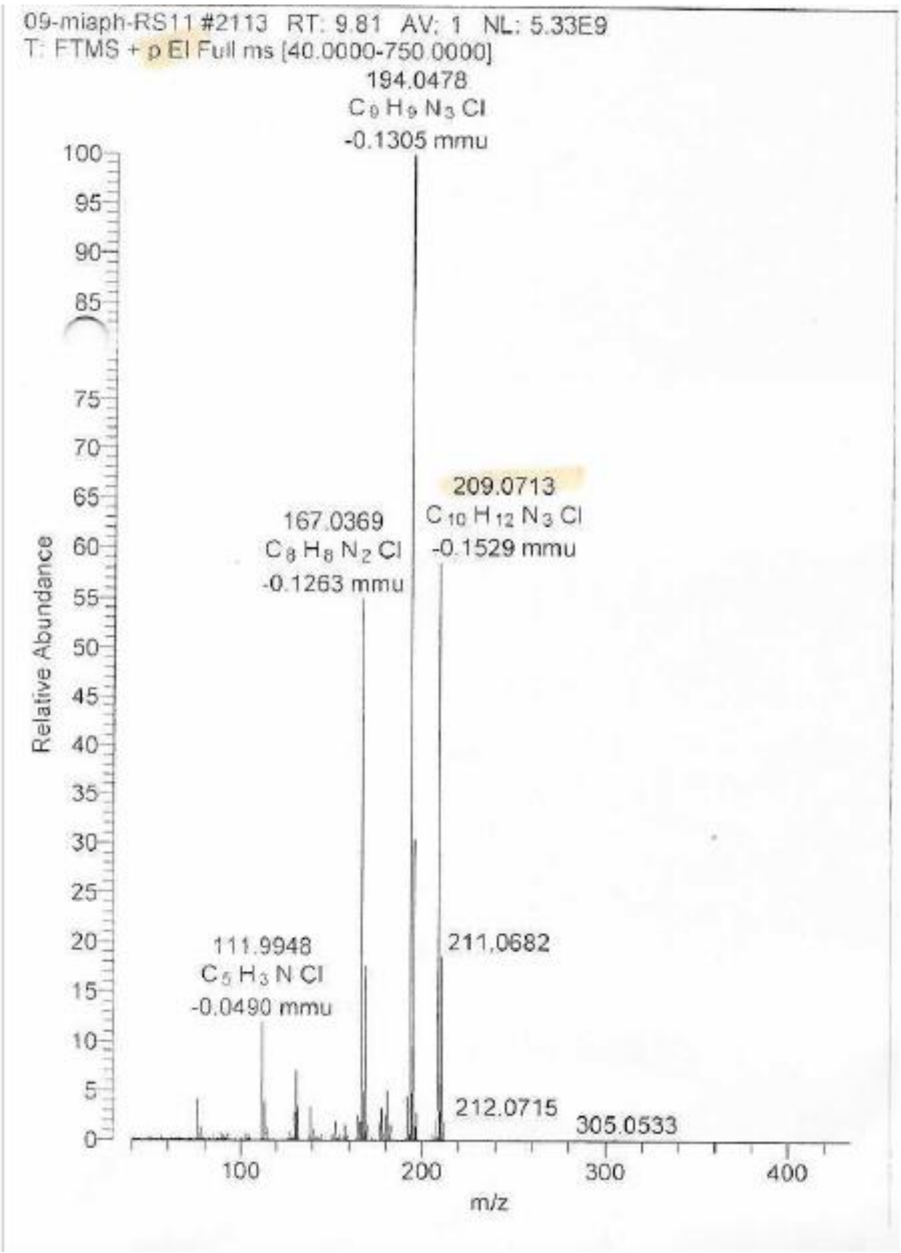

HRMS spectrum of compound **15:**

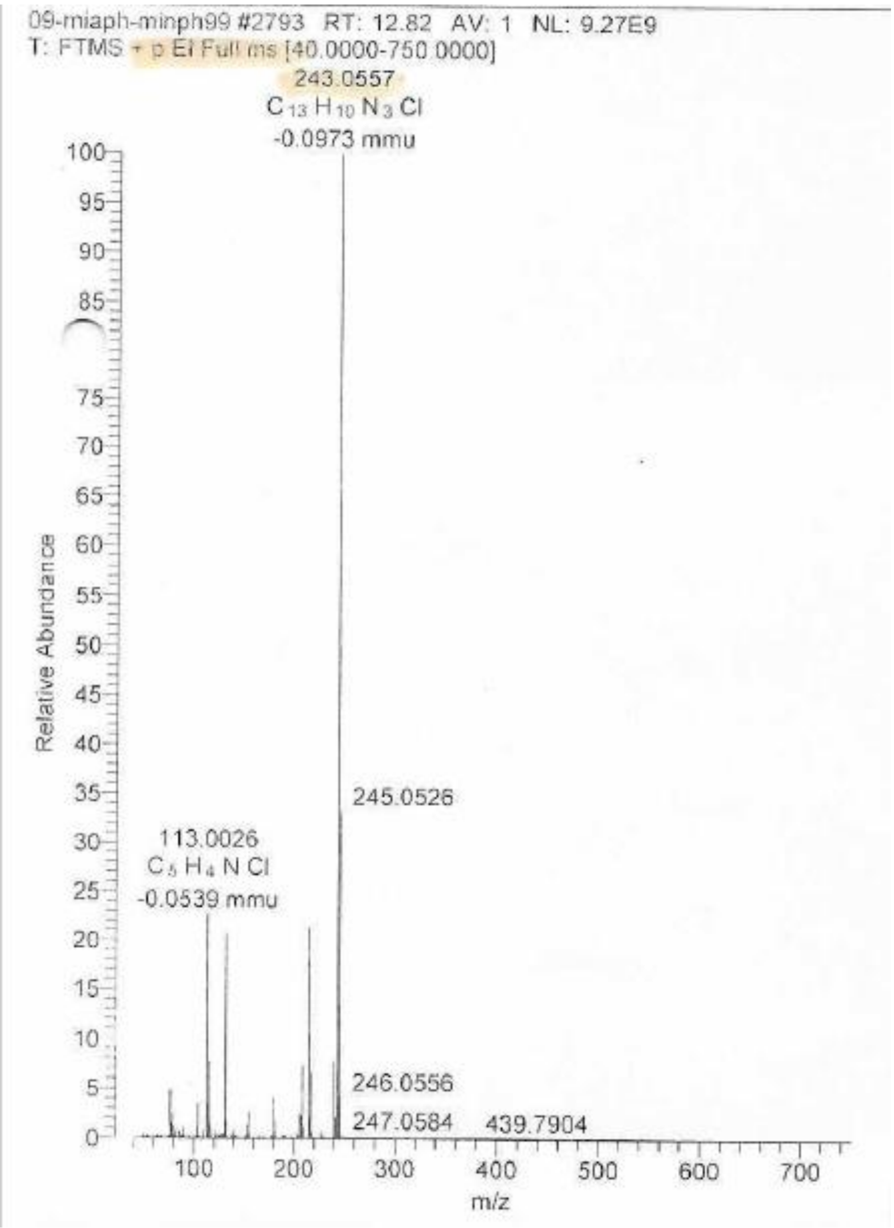

HRMS spectrum of compound **16:**

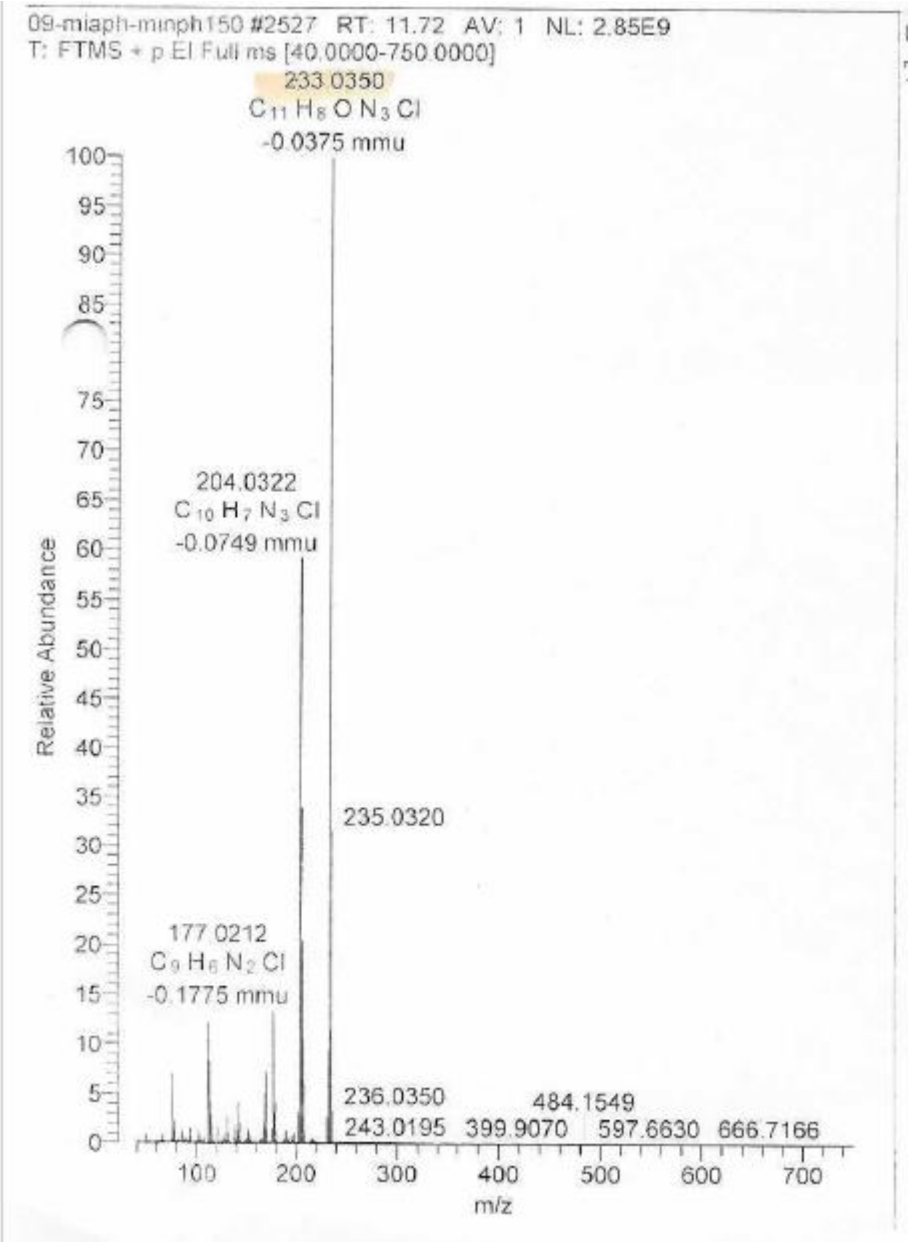

HRMS spectrum of compound **17:**

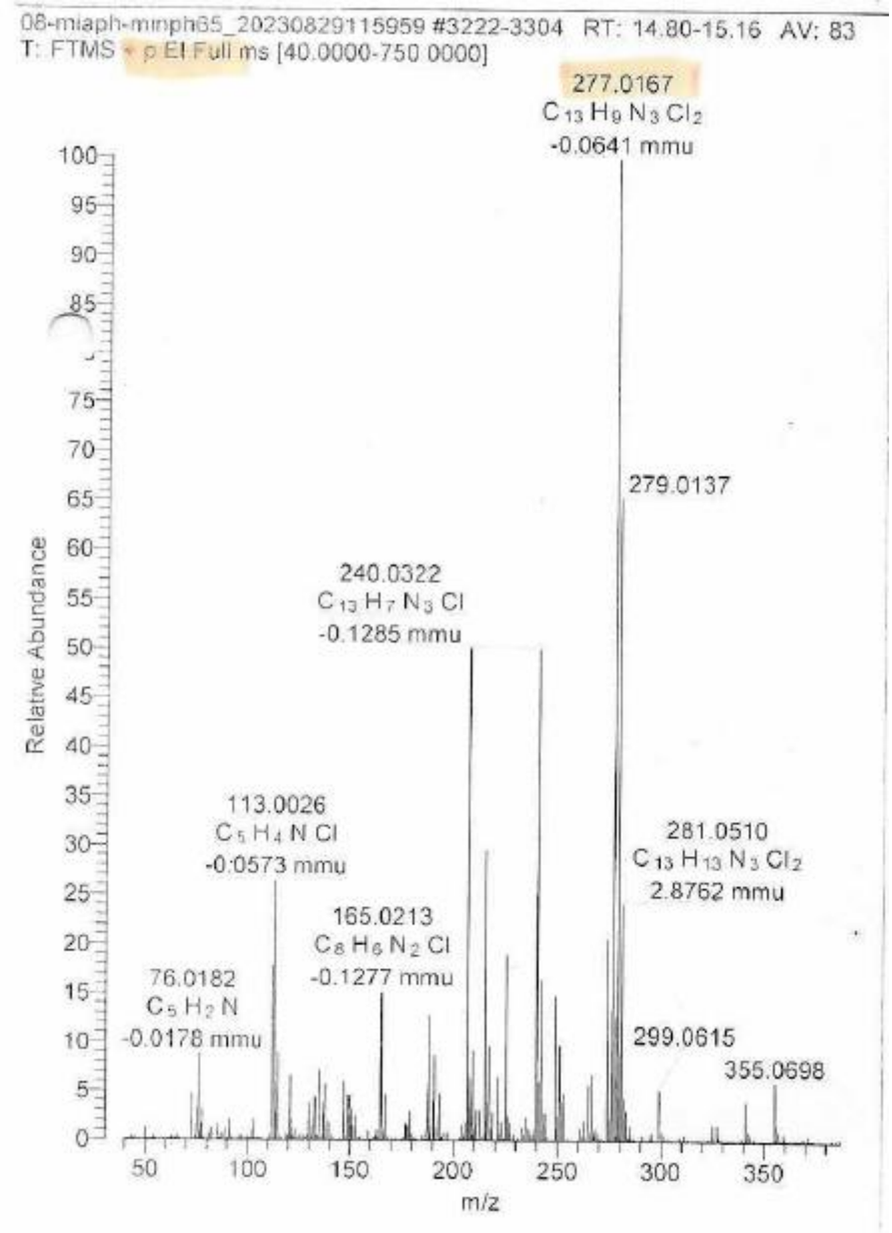

HRMS spectrum of compound **18:**

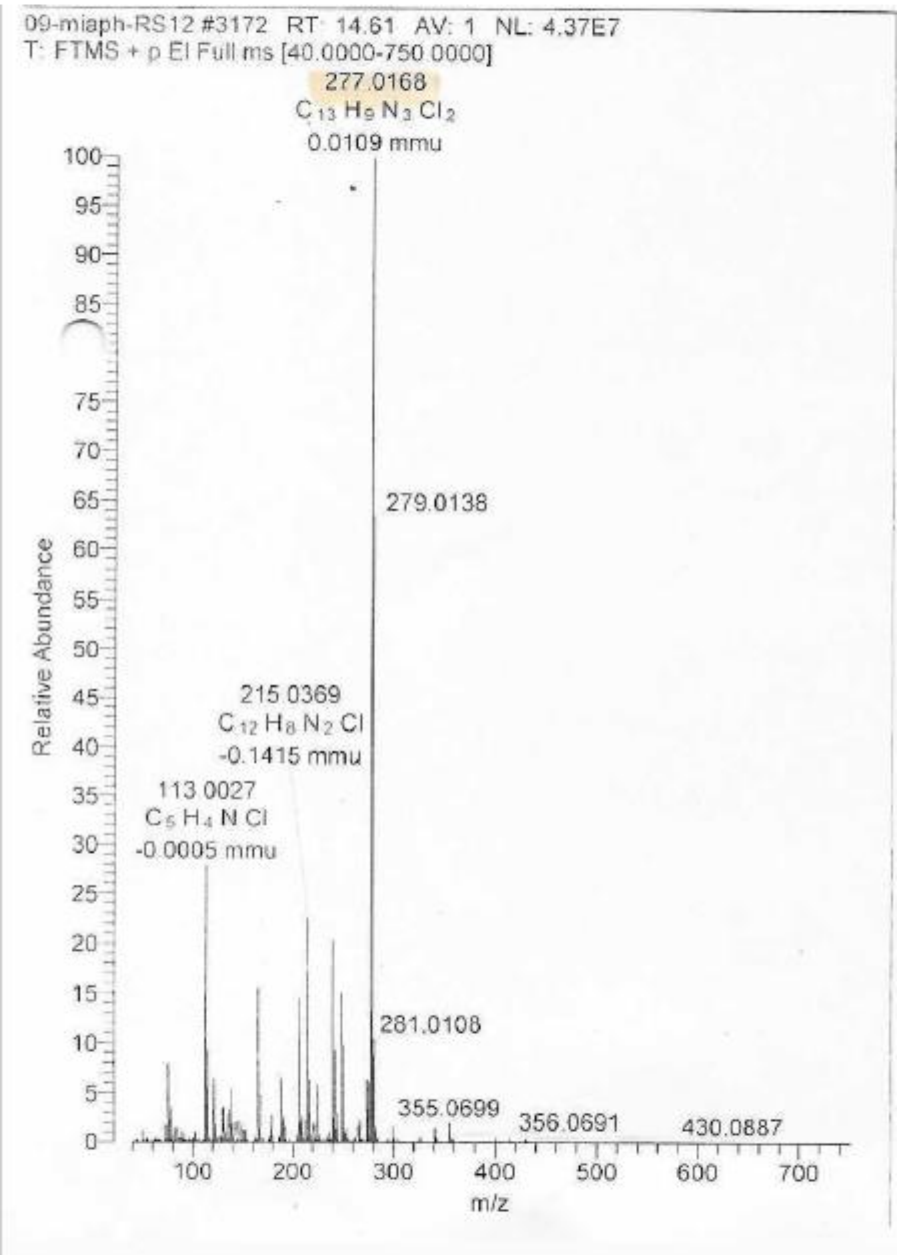

HRMS spectrum of compound **19:**

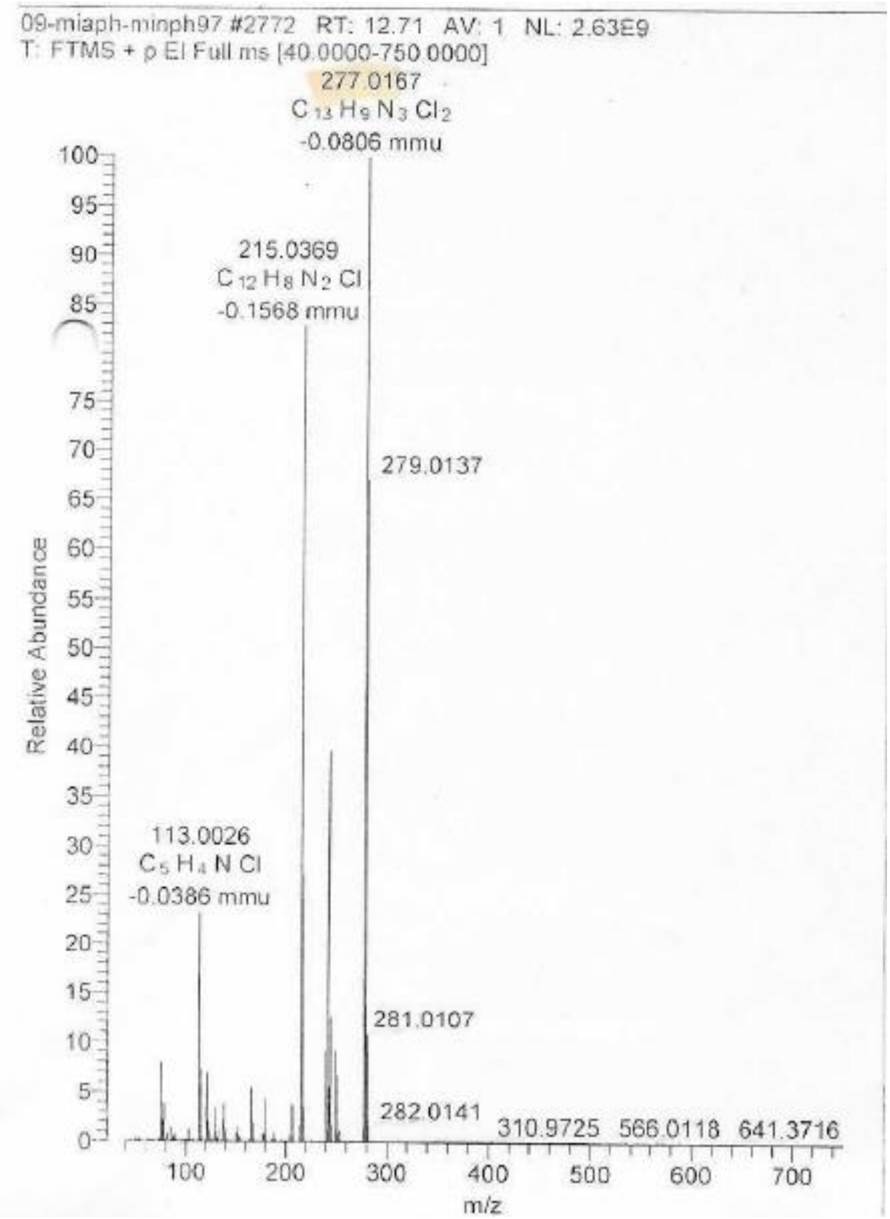

HRMS spectrum of compound **20:**

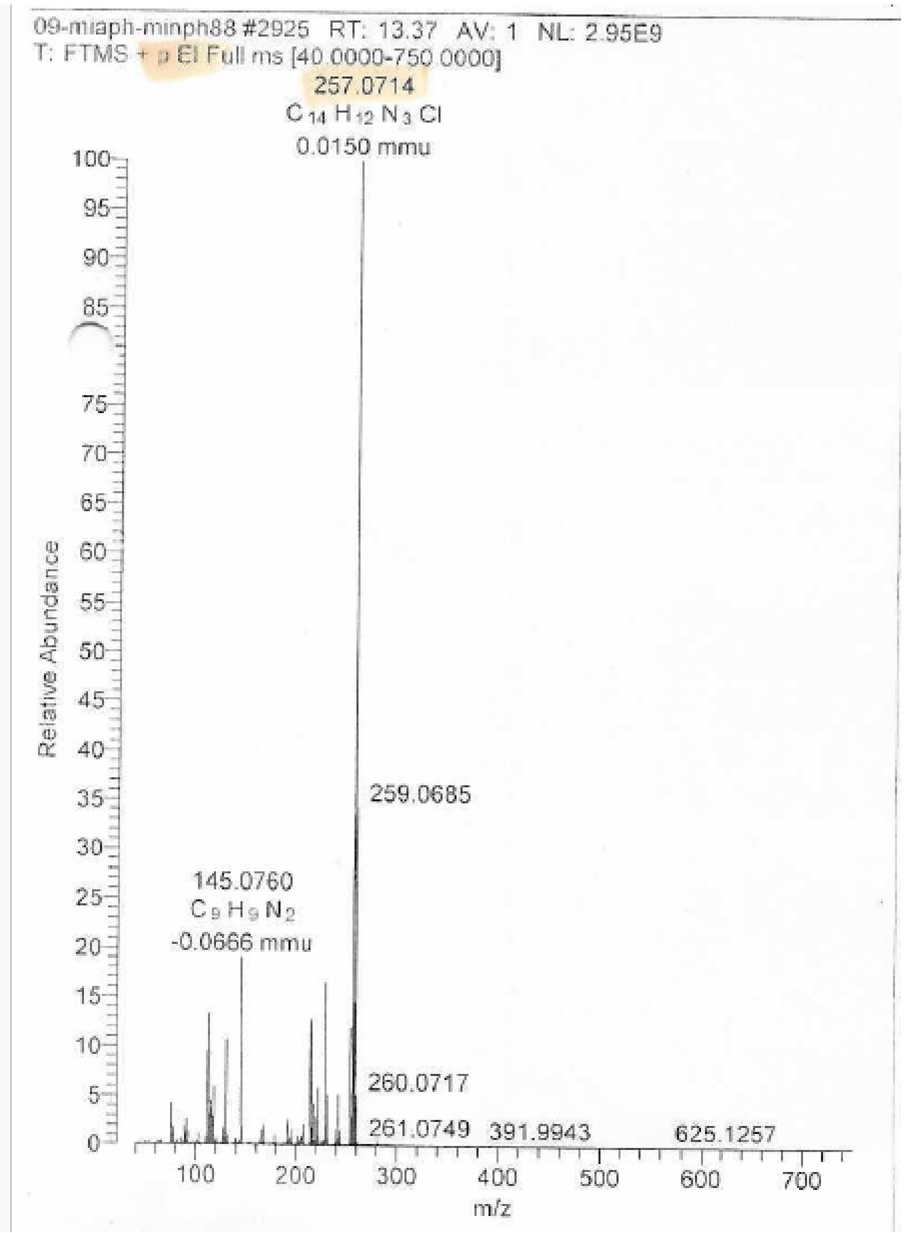

HRMS spectrum of compound **21:**

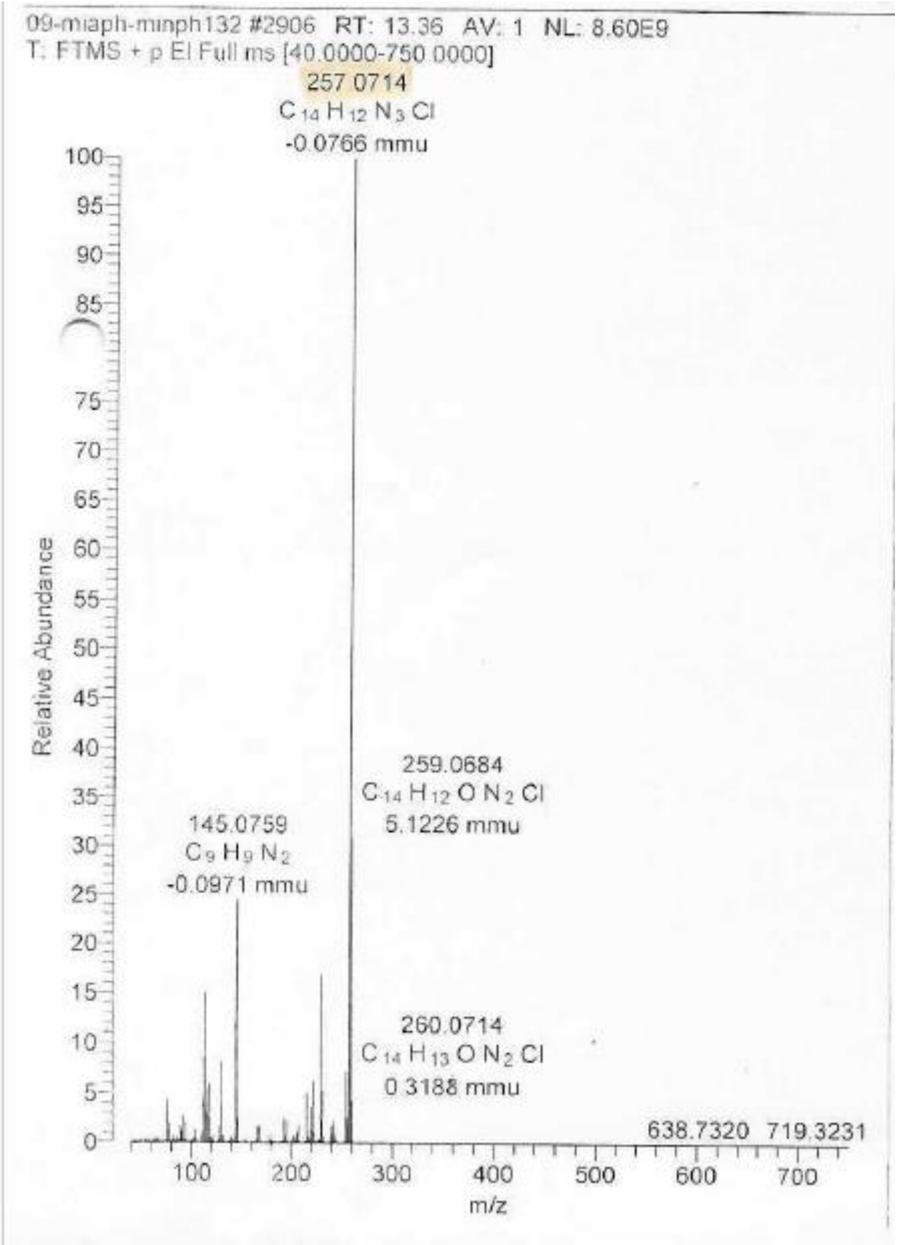

HRMS spectrum of compound **22:**

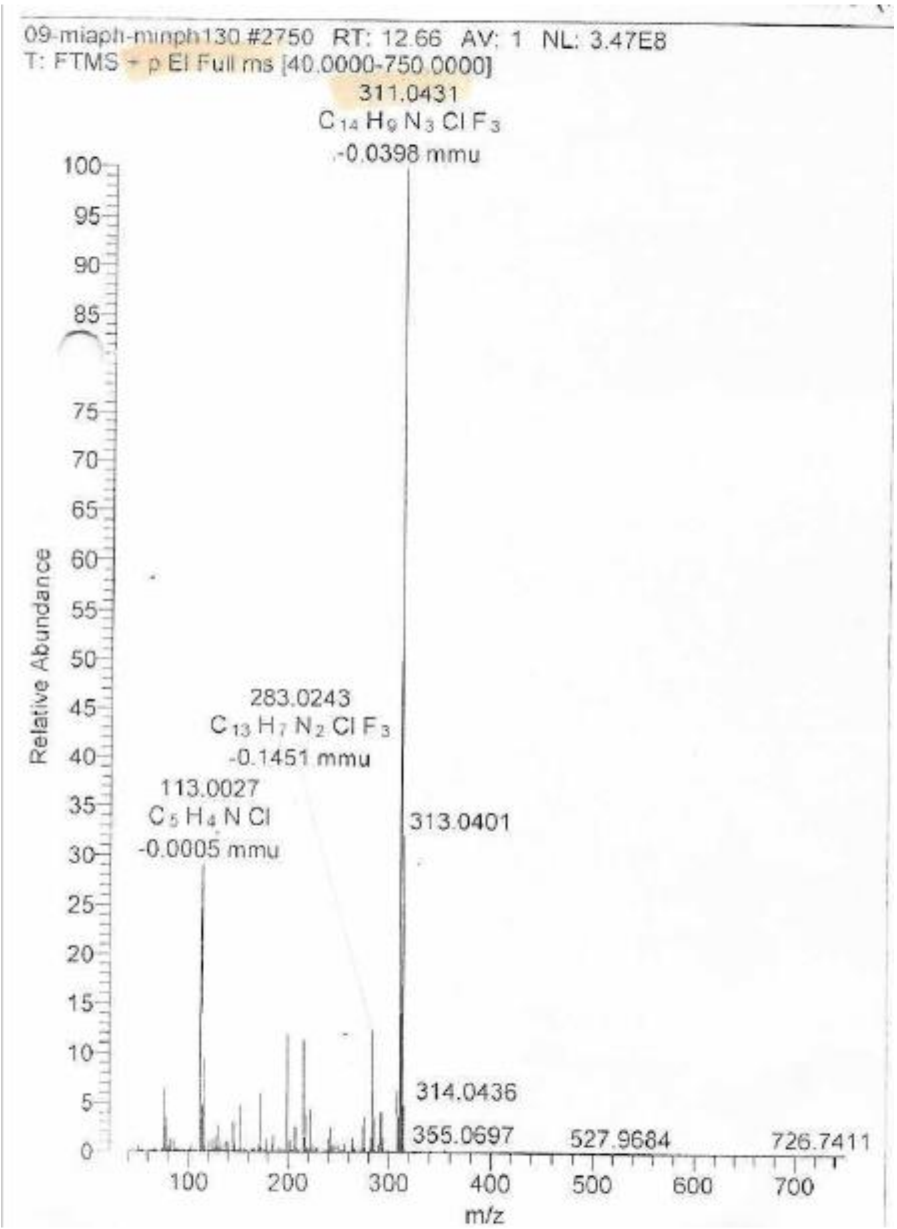

HRMS spectrum of compound **23:**

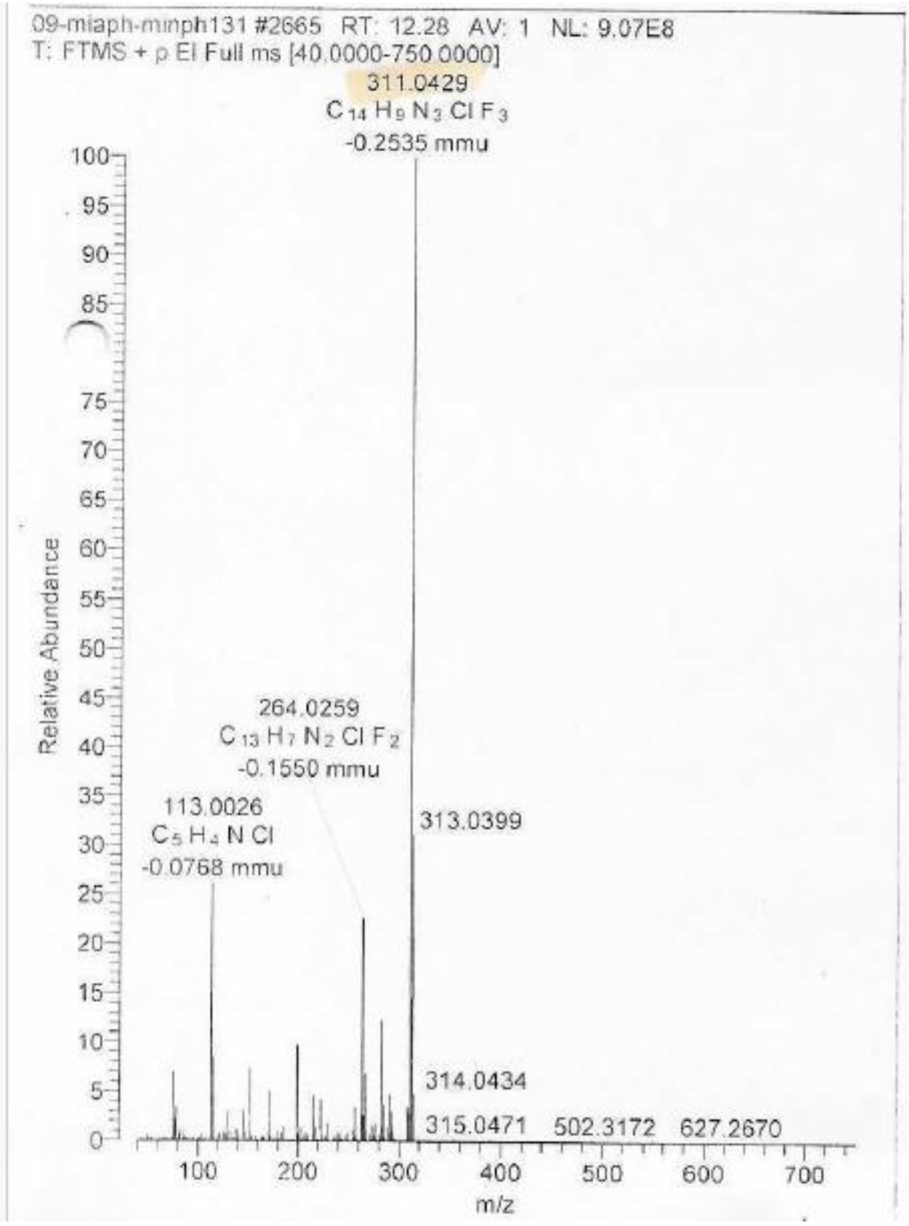

HRMS spectrum of compound **24:**

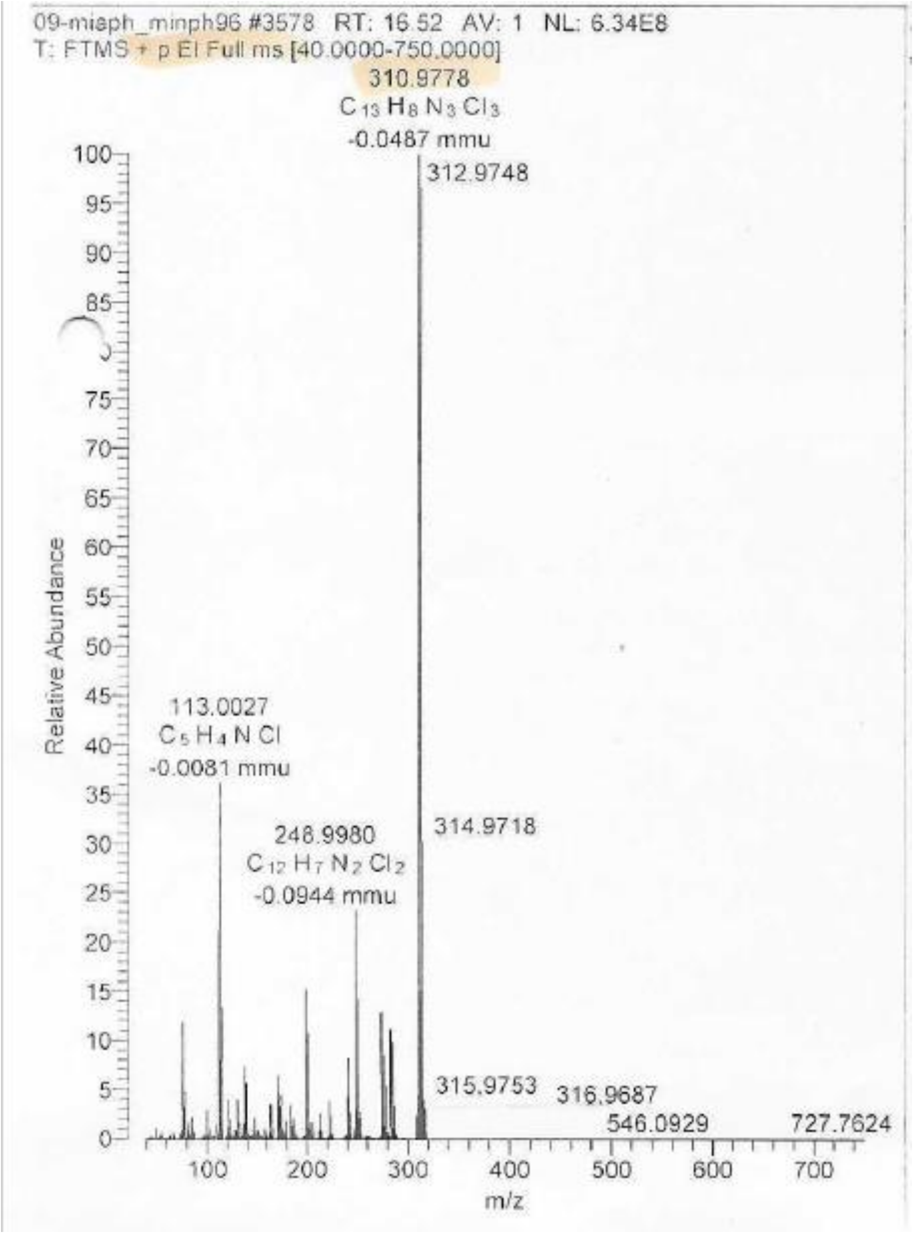

HRMS spectrum of compound **25:**

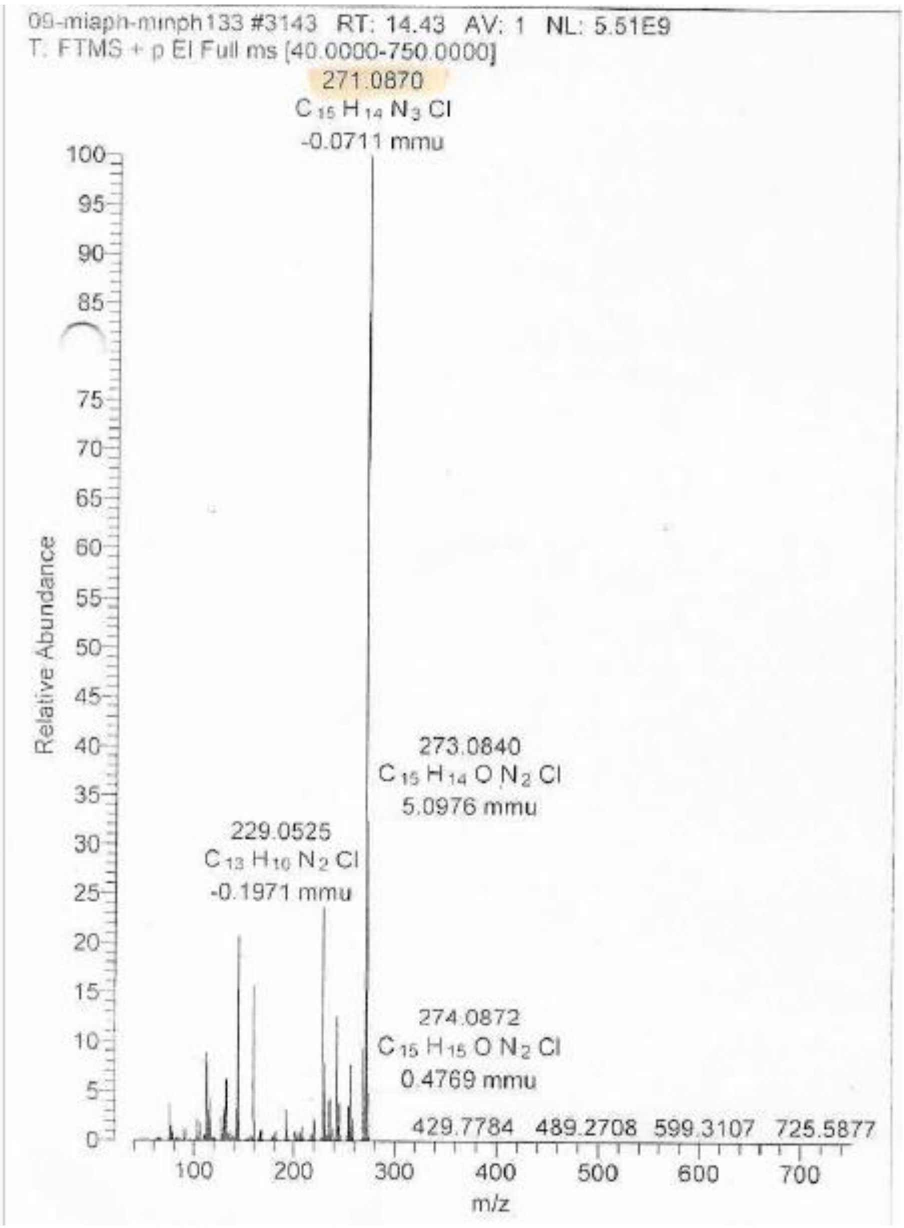

HRMS spectrum of compound **26:**

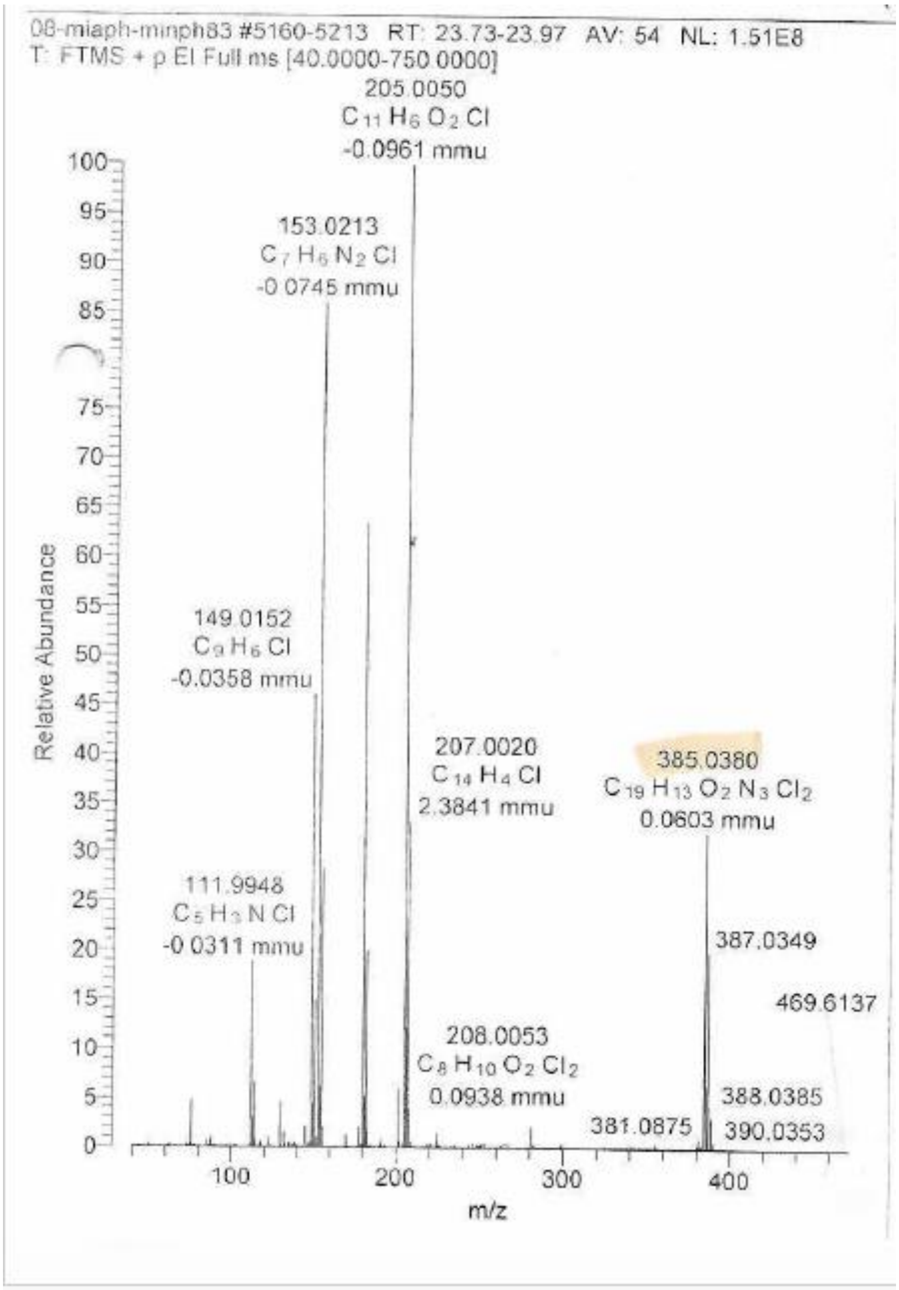

HRMS spectrum of compound **27:**

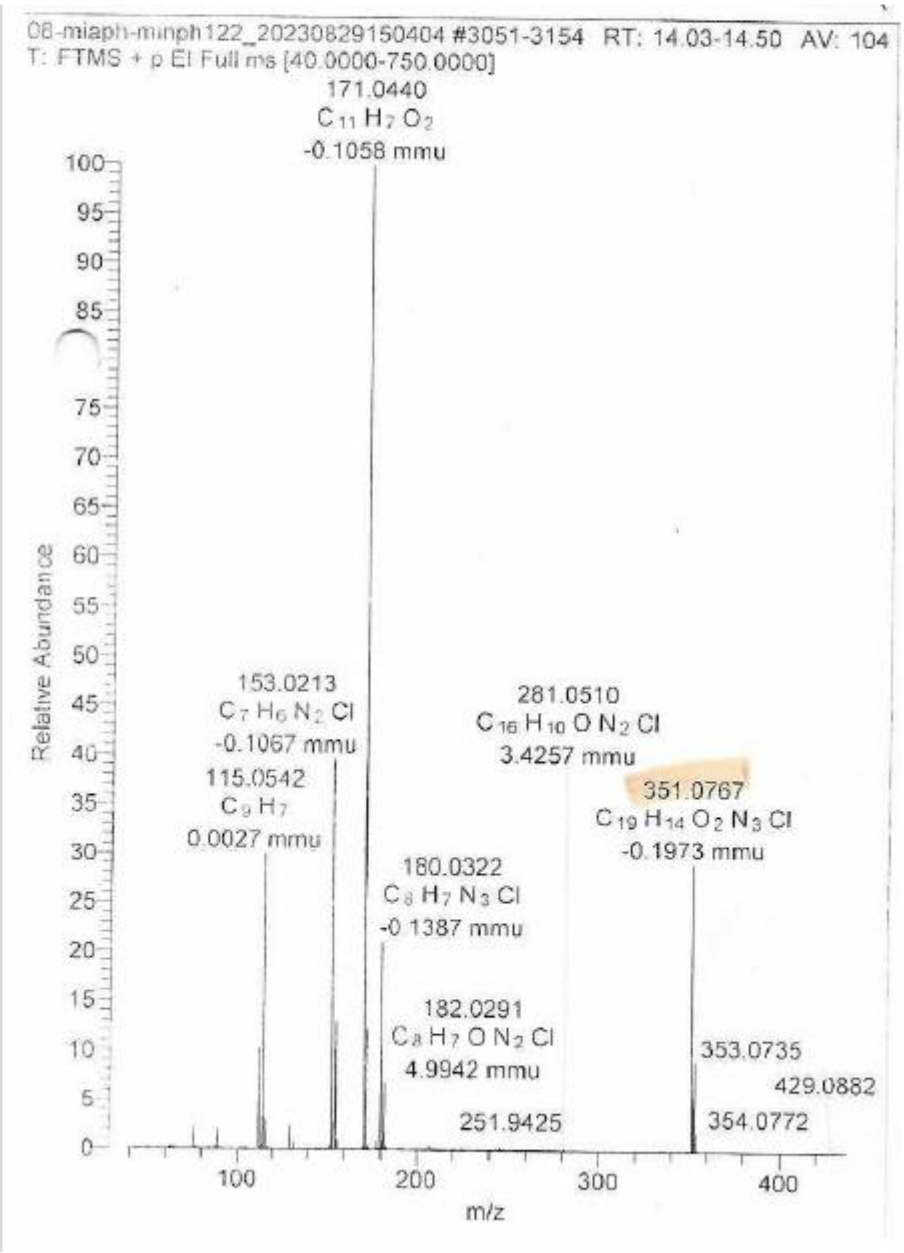

HRMS spectrum of compound **28:**

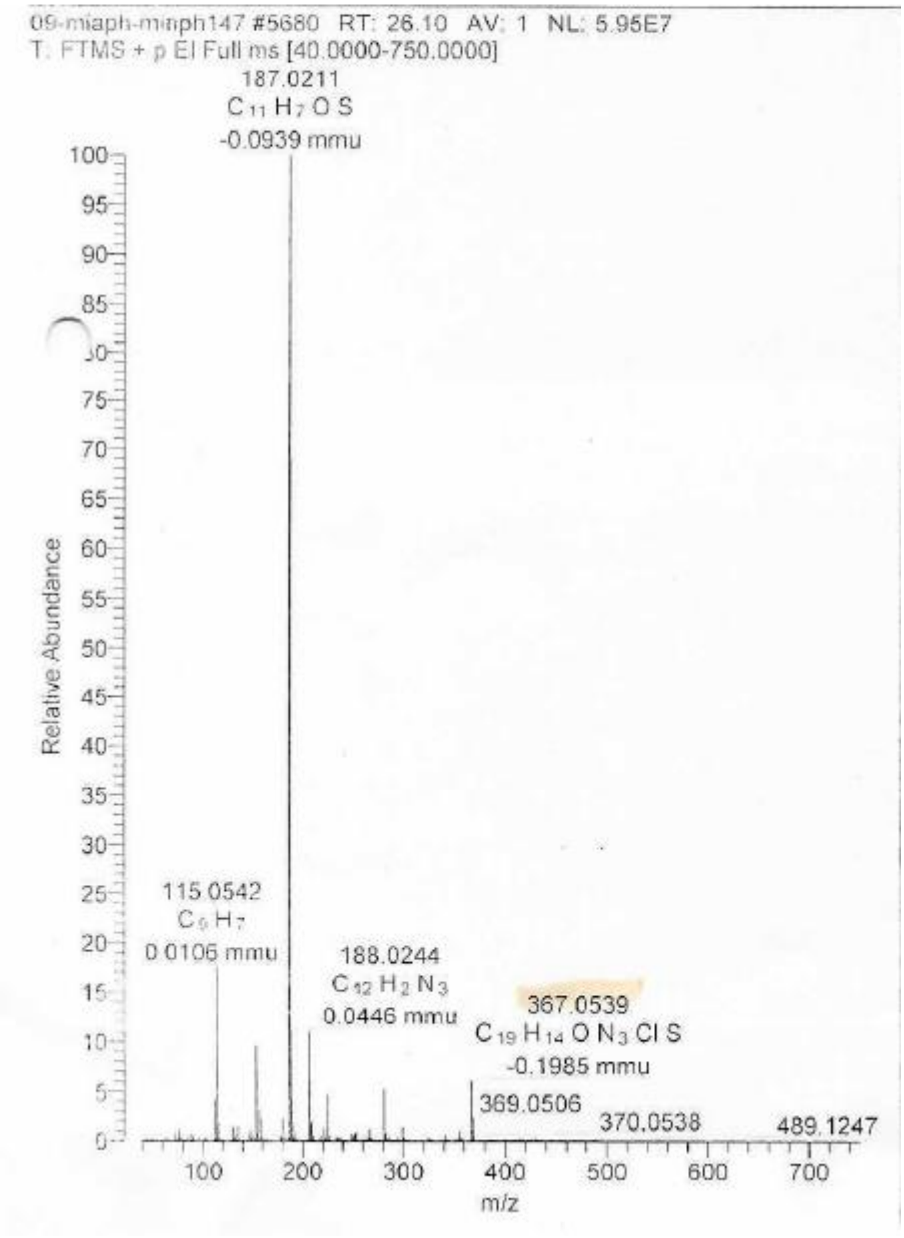

HRMS spectrum of compound **29:**

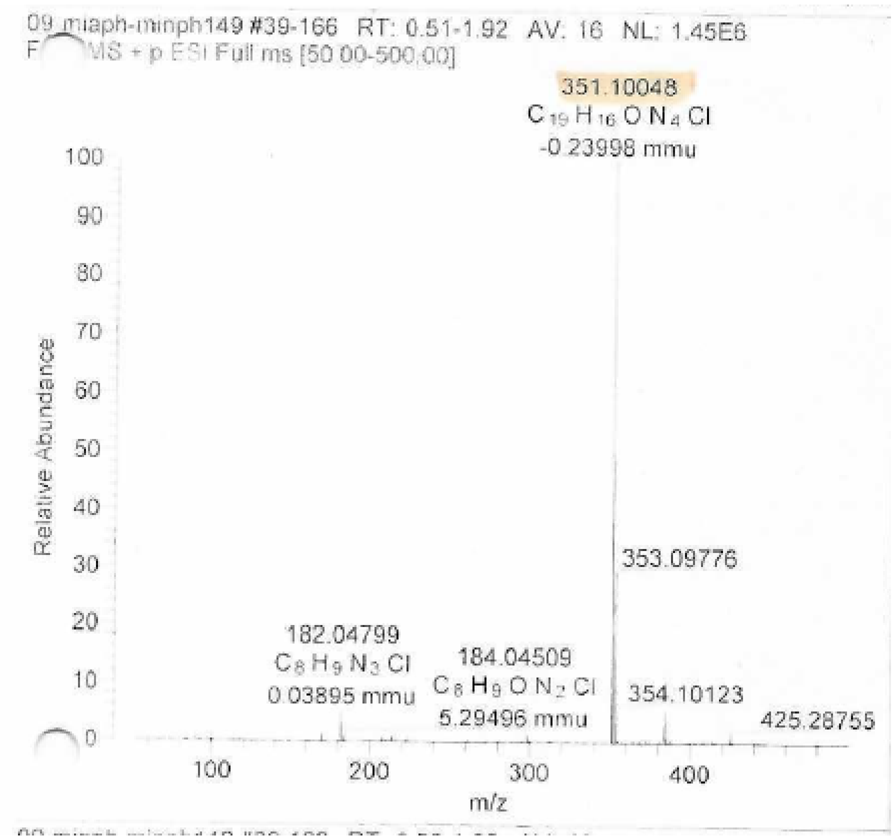

HRMS spectrum of compound **30:**

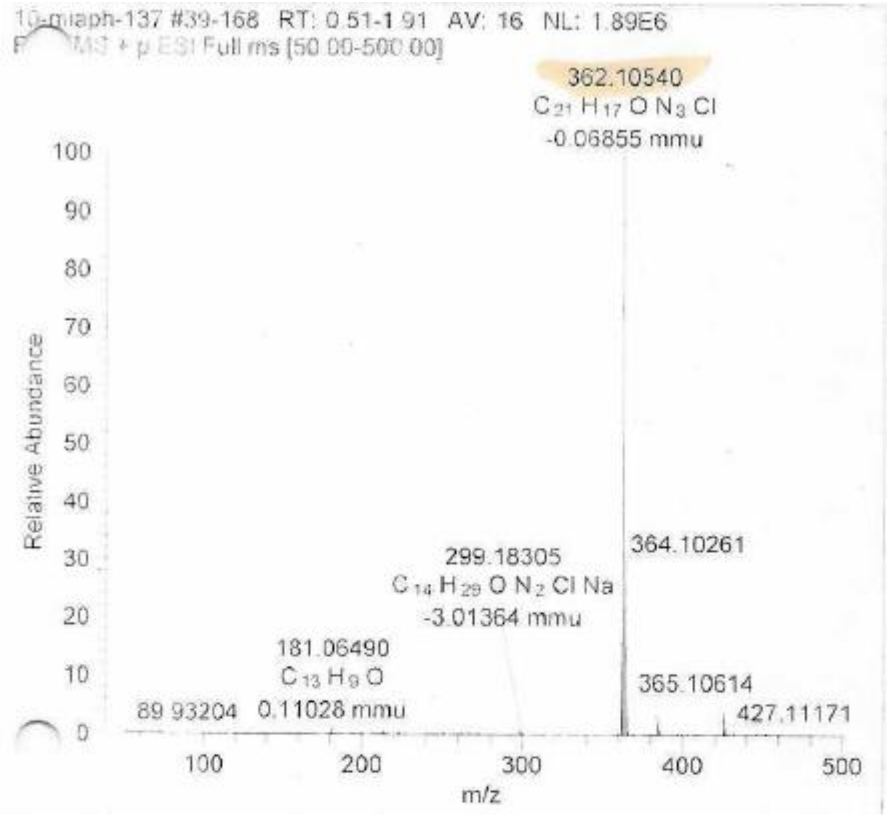

HRMS spectrum of compound **31:**

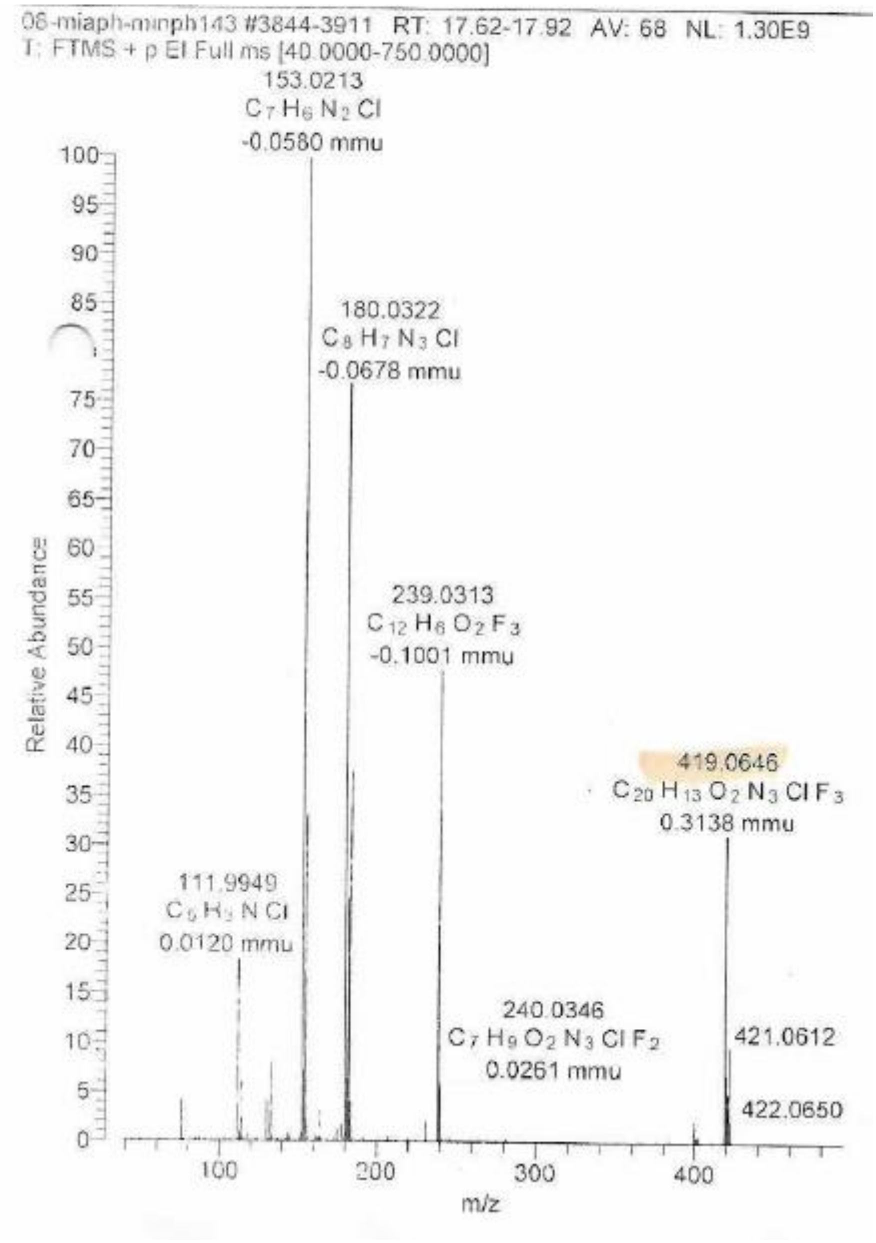

HRMS spectrum of compound **32:**

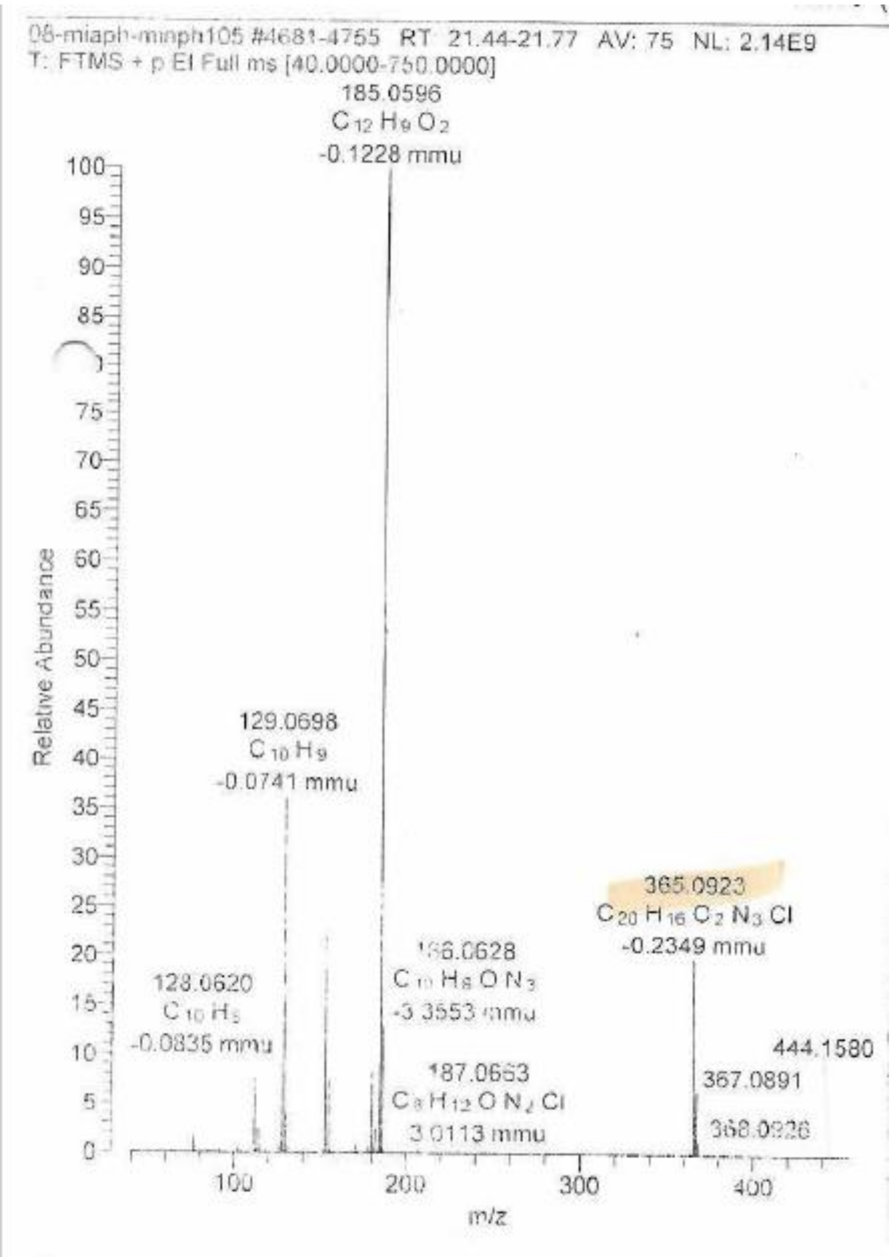

HRMS spectrum of compound **33:**

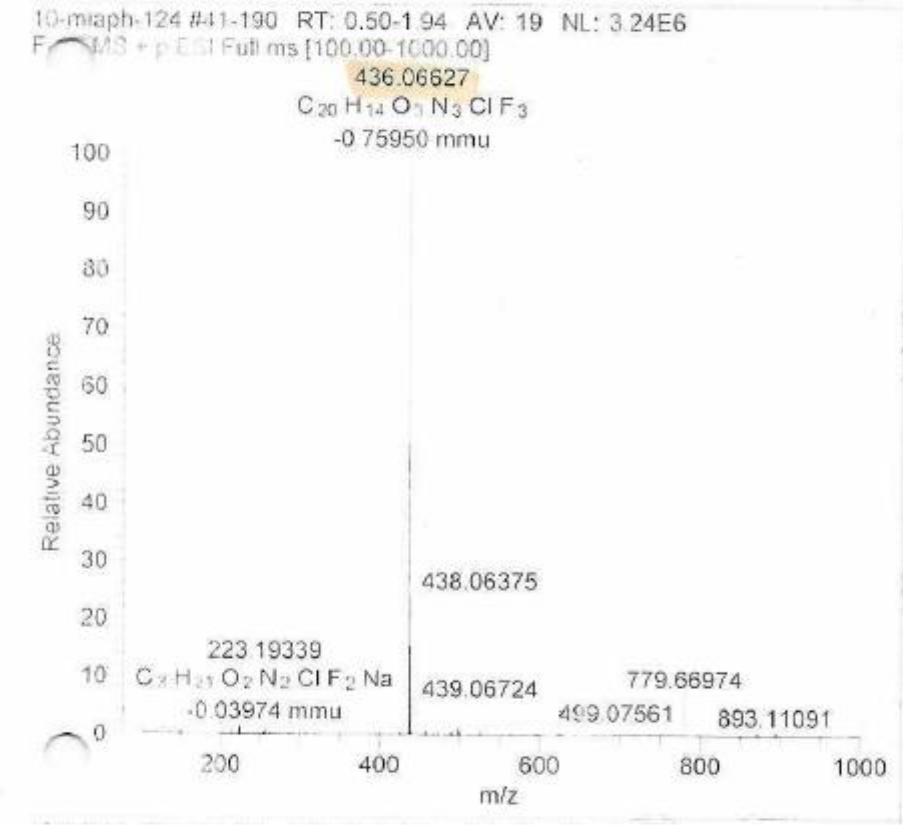

HRMS spectrum of compound **34:**

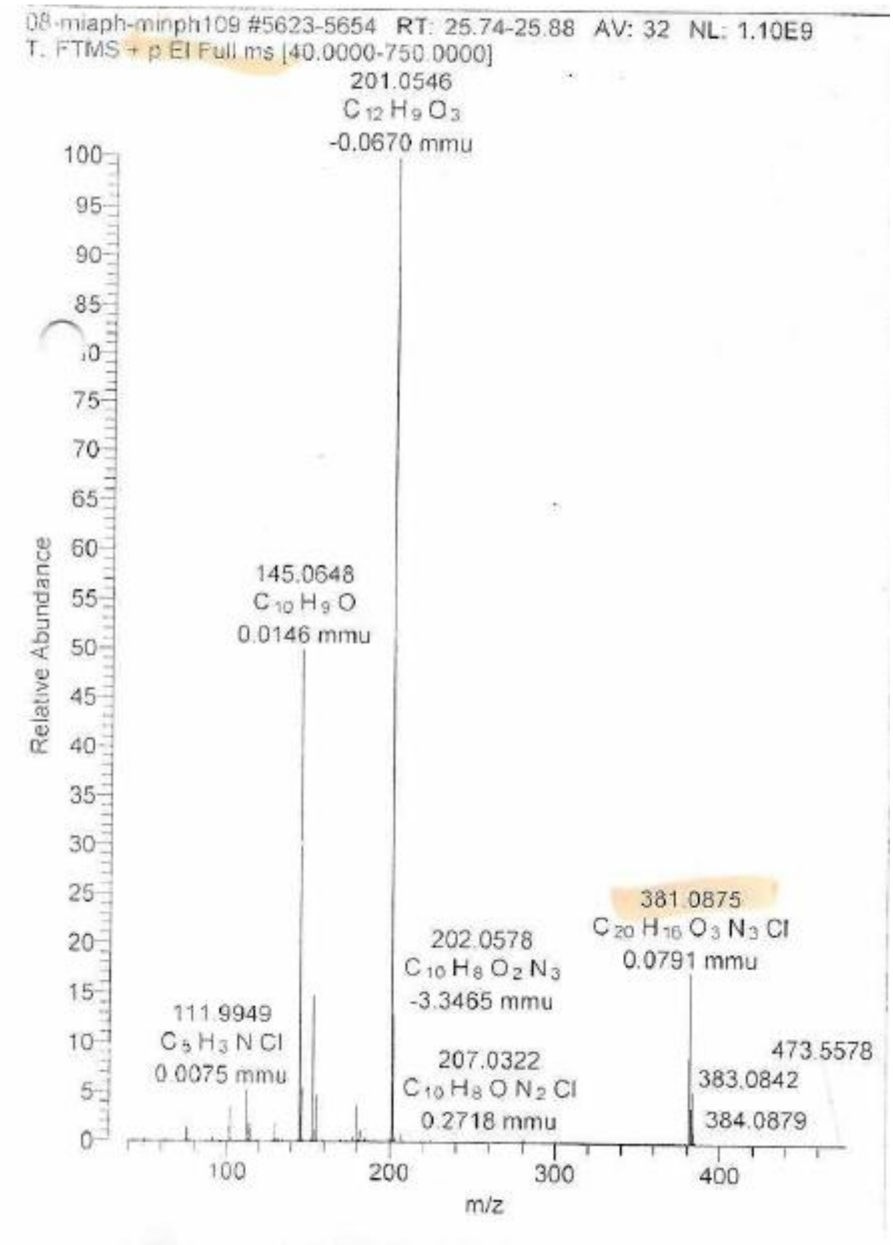

HRMS spectrum of compound **35:**

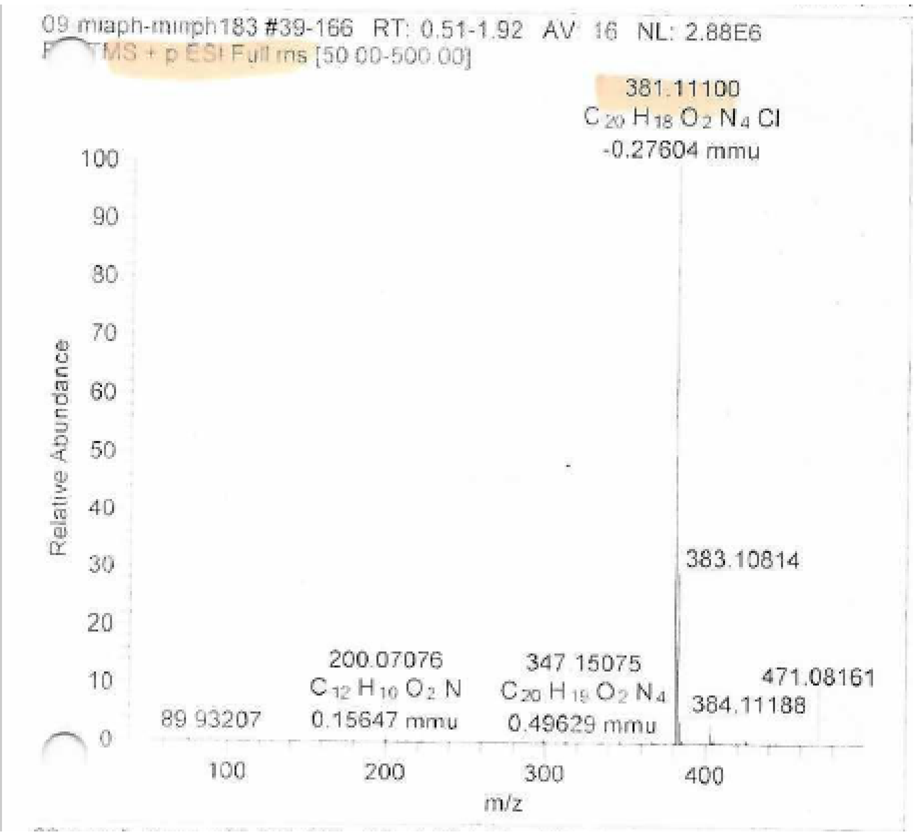

HRMS spectrum of compound **36:**

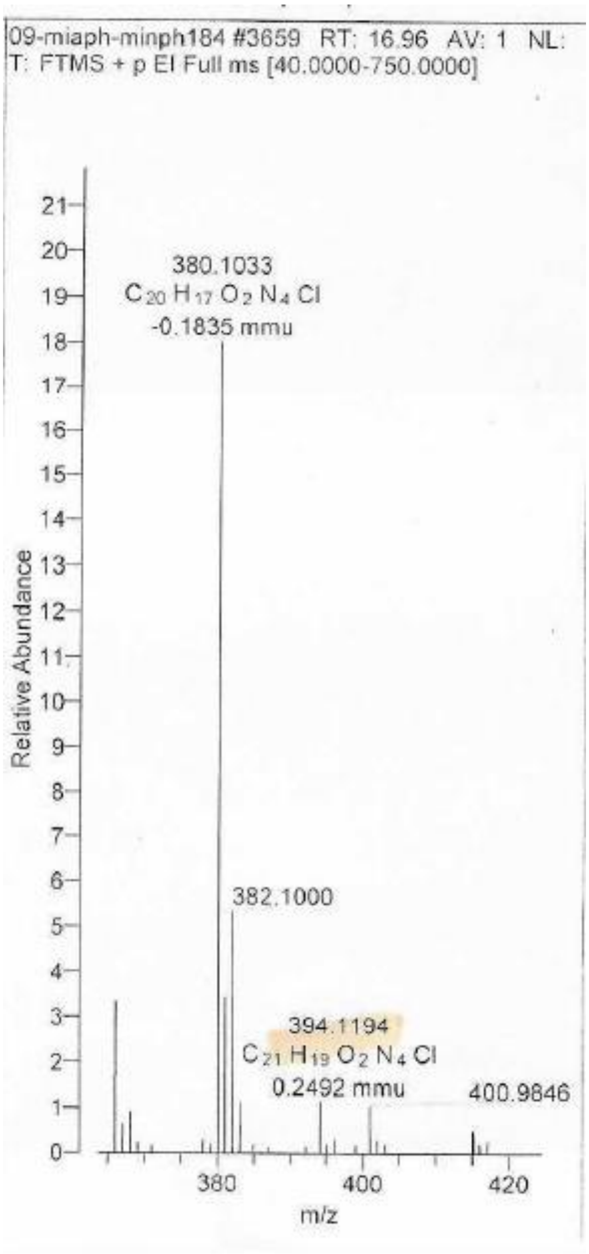

HRMS spectrum of compound **37:**

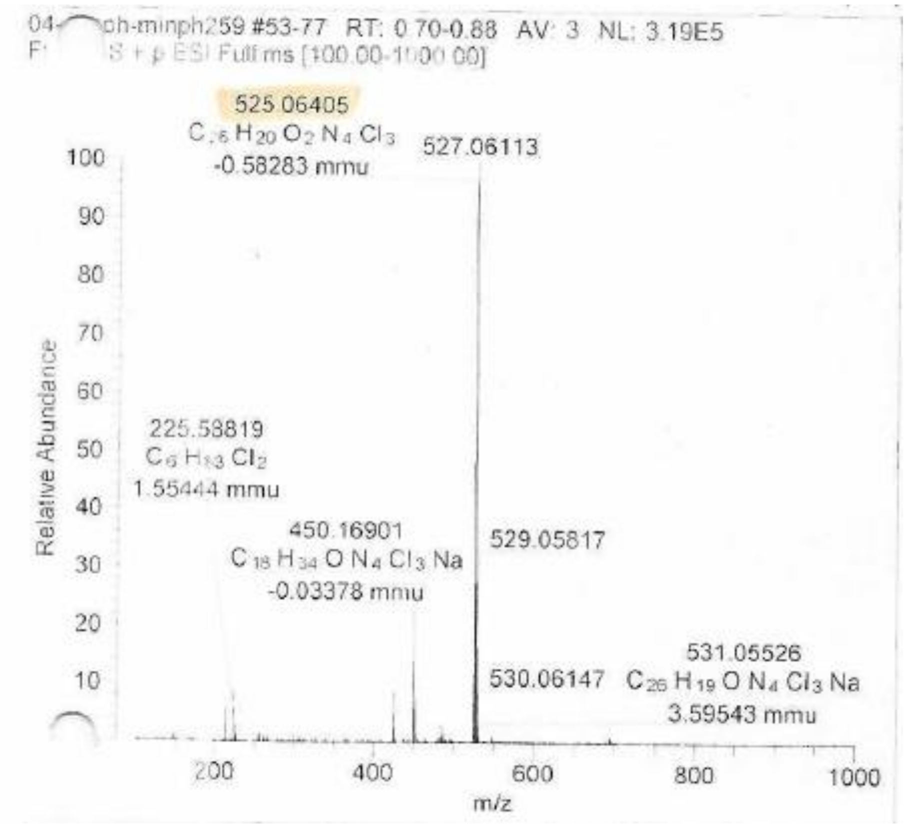

HRMS spectrum of compound **38:**

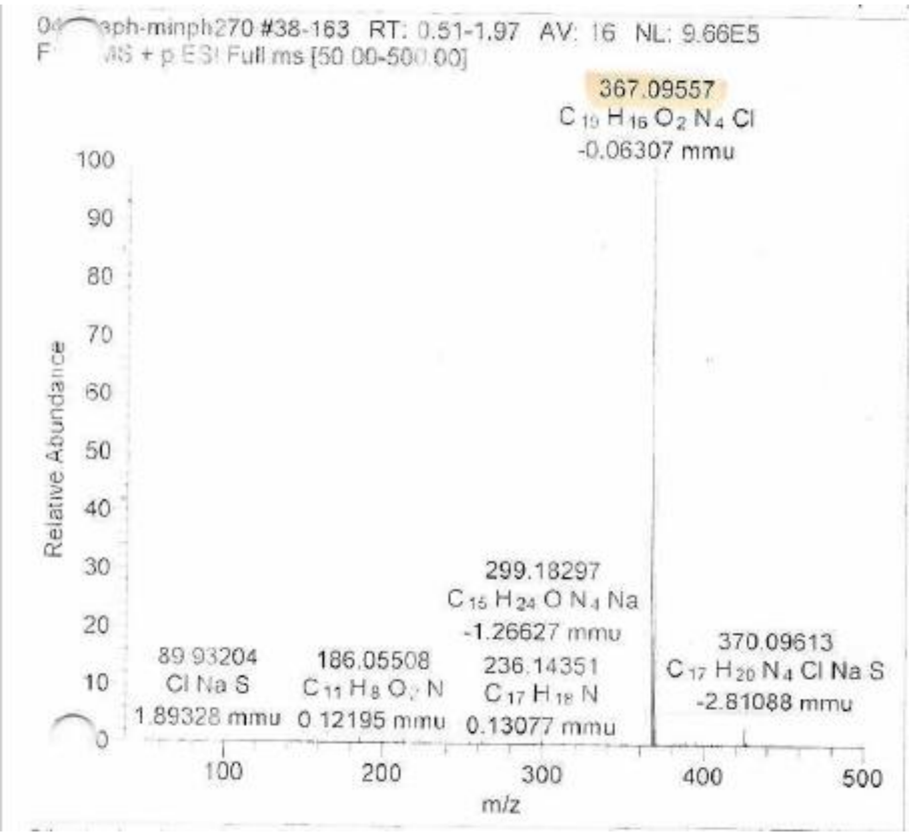

HRMS spectrum of compound **39:**

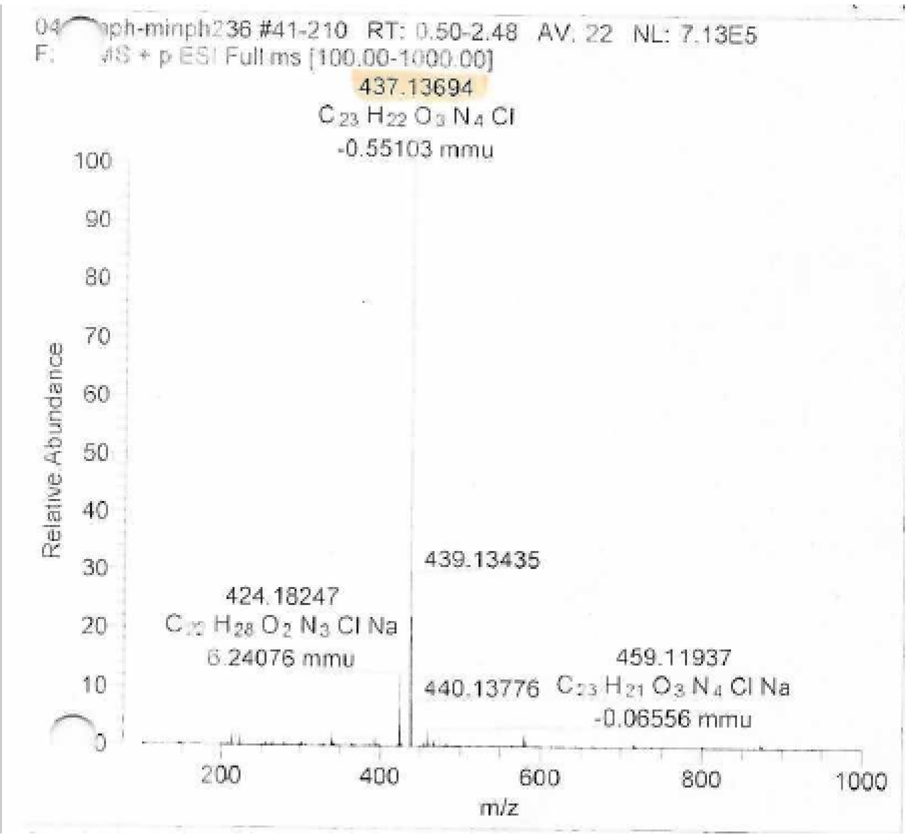

HRMS spectrum of compound **40:**

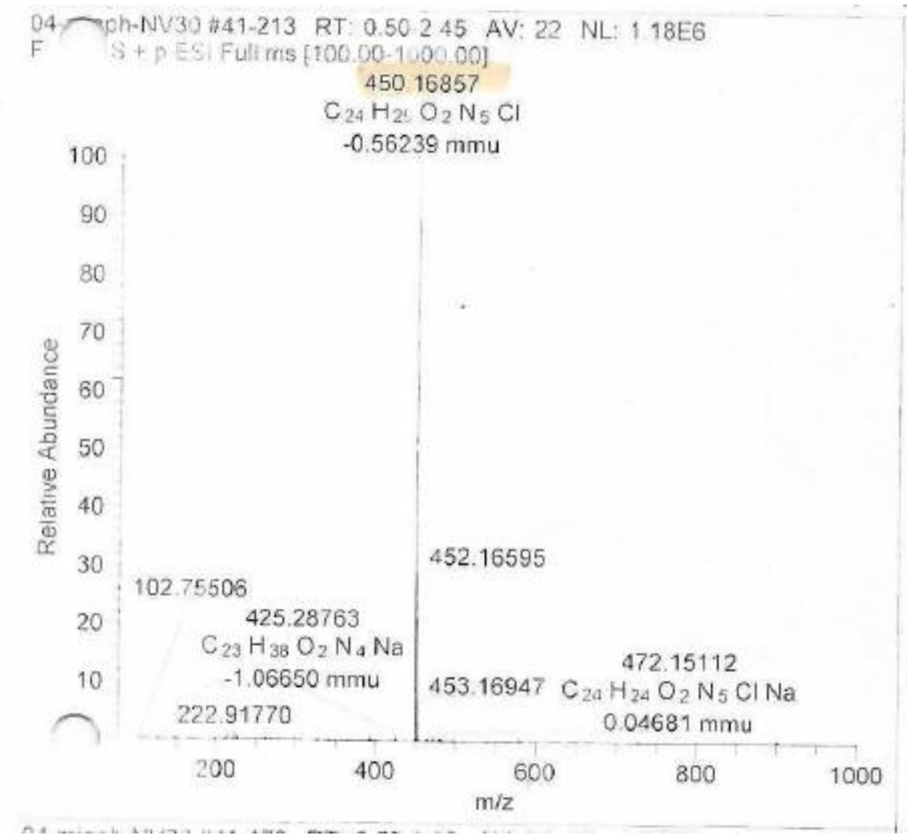

HRMS spectrum of compound **41:**

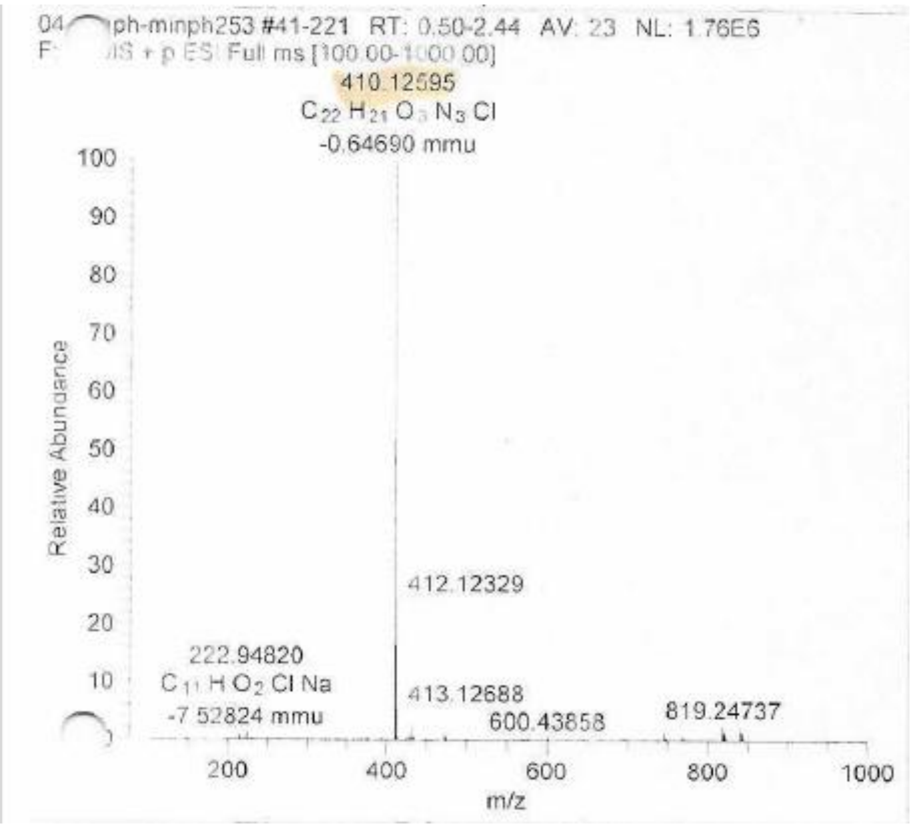

HRMS spectrum of compound **42:**

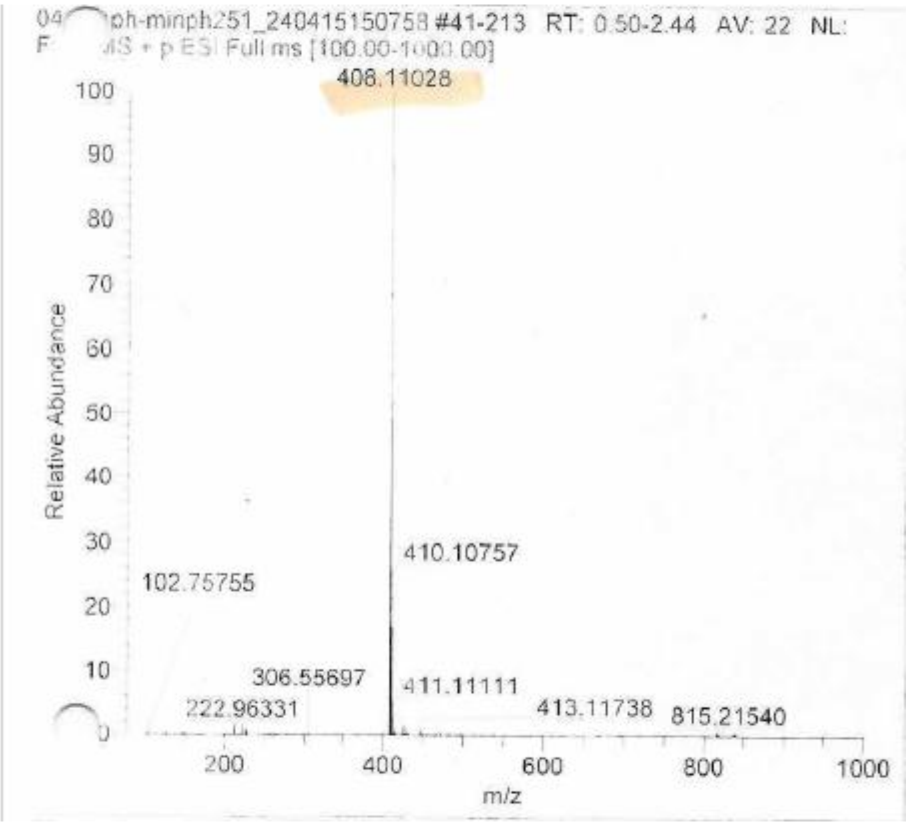

Supplement: Supplementary file 3 — Supplementary Data 1 [file 42004_2024_1224_MOESM3_ESM.pdf]
